# Spatial analyzes of HLA data in Rio Grande do Sul, south Brazil: genetic structure and possible correlation with autoimmune diseases

**DOI:** 10.1186/s12942-018-0154-8

**Published:** 2018-09-14

**Authors:** Juliano André Boquett, Marcelo Zagonel-Oliveira, Luis Fernando Jobim, Mariana Jobim, Luiz Gonzaga, Maurício Roberto Veronez, Nelson Jurandi Rosa Fagundes, Lavínia Schüler-Faccini

**Affiliations:** 1 Instituto Nacional de Genética Médica Populacional (INaGeMP), Porto Alegre, Brazil; 2 Post-Graduate Program in Genetics and Molecular Biology, Departamento de Genética, Universidade Federal do Rio Grande do Sul, Agencia Campus UFRGS, Caixa Postal 15053, Porto Alegre, RS CEP 91501-970 Brazil; 3 Advanced Visualization and Geoinformatics Laboratory (VIZLab), Applied Computing Graduate Program, Universidade do Vale do Rio dos Sinos, São Leopoldo, RS Brazil; 4 Department of Immunology, Hospital de Clínicas de Porto Alegre, Porto Alegre, Brazil

**Keywords:** HLA, Autoimmune diseases, Genetic structure, Correlation, Georeferencing

## Abstract

**Background:**

HLA genes are the most polymorphic of the human genome and have distinct allelic frequencies in populations of different geographical regions of the world, serving as genetic markers in ancestry studies. In addition, specific HLA alleles may be associated with various autoimmune and infectious diseases. The bone marrow donor registry in Brazil is the third largest in the world, and it counts with genetic typing of HLA-A, -B, and -DRB1. Since 1991 Brazil has maintained the DATASUS database, a system fed with epidemiological and health data from compulsory registration throughout the country.

**Methods:**

In this work, we perform spatial analysis and georeferencing of HLA genetic data from more than 86,000 bone marrow donors from Rio Grande do Sul (RS) and data of hospitalization for rheumatoid arthritis, multiple sclerosis and Crohn’s disease in RS, comprising the period from 1995 to 2016 obtained through the DATASUS system. The allele frequencies were georeferenced using Empirical Bayesian Kriging; the diseases prevalence were georeferenced using Inverse Distance Weighted and cluster analysis for both allele and disease were performed using Getis-Ord Gi* method. Spearman’s test was used to test the correlation between each allele and disease.

**Results:**

The results indicate a HLA genetic structure compatible with the history of RS colonization, where it is possible to observe differentiation between regions that underwent different colonization processes. Spatial analyzes of autoimmune disease hospitalization data were performed revealing clusters for different regions of the state for each disease analyzed. The correlation test between allelic frequency and the occurrence of autoimmune diseases indicated a significant correlation between the HLA-B*08 allele and rheumatoid arthritis.

**Conclusions:**

Genetic mapping of populations and the spatial analyzes such as those performed in this work have great economic relevance and can be very useful in the formulation of public health campaigns and policies, contributing to the planning and adjustment of clinical actions, as well as informing and educating professionals and the population.

**Electronic supplementary material:**

The online version of this article (10.1186/s12942-018-0154-8) contains supplementary material, which is available to authorized users.

## Background

Harboring more than 200 genes spread over a 3.6 Mb region, the Major Histocompatibility Complex (MHC) is the region of the human genome most enriched for open reading frames [1]. MHC genes, or HLA (Human Leukocyte Antigen) genes in humans, are the most polymorphic loci of the human genome [2], showing different allelic frequencies in populations from different geographic regions around the world [3–5]. Due to their high genetic variability and strong linkage disequilibrium, HLA genes have been used in studies of genetic ancestry and demography [6]. Due to their major role in immune response, the *loci* of the HLA system are the primary determinants of tolerance or rejection in organ and hematopoietic stem cell transplantation (HSCT) [7]. HSCT from bone marrow is clinically indicated for the treatment of disorders of the hematopoietic system or the immune system and in cases of malignant bone marrow diseases and disseminated solid tumors. Leukemia is the leading indication for allogeneic HSCT (72%), followed by lymphoproliferative diseases (15%), non-malignant diseases (12%) and solid tumors (0.6%) [8, 9].

In addition, specifc HLA alleles have already been associated with various autoimmune and infectious diseases [10, 11]. As a class, the overall cumulative prevalence for all autoimmune diseases (AD) is 5.0%, being 3.0% for males and 7.1% for females [12]. Rheumatoid arthritis (RA) is the most prevalent AD (0.5–1%) [13], being the HLA-DRB1 is the principal locus contributing to disease susceptibility, with an estimated contribution of 30–50% to overall susceptibility to RA [14, 15]. Other AD, such as Celiac Disease, Type 1 Diabetes Mellitus, Ankylosing spondylitis, Multiple sclerosis and Crohn’s disease also presented HLA genes associated with its susceptibility [see [16] and [17] for further review]. Thus, knowledge of HLA diversity at the population level is important to guide public health policies focused on AD and to improve bone marrow transplantation programs.

The use of the geographic information system (GIS)—a toolkit for capturing, storing, transforming, analyzing and presenting spatial data—has been a powerful tool in assessing and monitoring public health in different populations around the world [18, 19]. GIS-based data contributes to the improvement of health-related services for the population, since health data combined with geographic information allow researchers to analyze the spatial variation of diseases, mortality, morbidity, access to health care systems and social or environmental determinants for health outcomes [20, 21]. The transformation of detailed data into maps can facilitate communication of the geographical distribution of health challenges in different communities and identify areas for intervention [18, 22].

The Brazilian Bone Marrow Donor Registry (REDOME, in Portuguese) is the third largest bank of bone marrow donors in the world, with more than 4 million donors registered to date. The state of Rio Grande do Sul (RS), in southern Brazil, has the fourth largest number of registered donors in Brazil, with approximately 300,000 individuals. This register contains information of HLA-A, -B and -DRB1 genotypes, city of residence of the donor as well as ethnicity by self-declaration based on skin color. In this work, we used GIS tools to evaluate the spatial correlation between immune system alleles (from HLA-A, -B and -DRB1 *loci*) and occurrence of AD in Rio Grande do Sul, based on data information available from governmental health agencies.

## Methods

### Sample

**Fig. 1:**
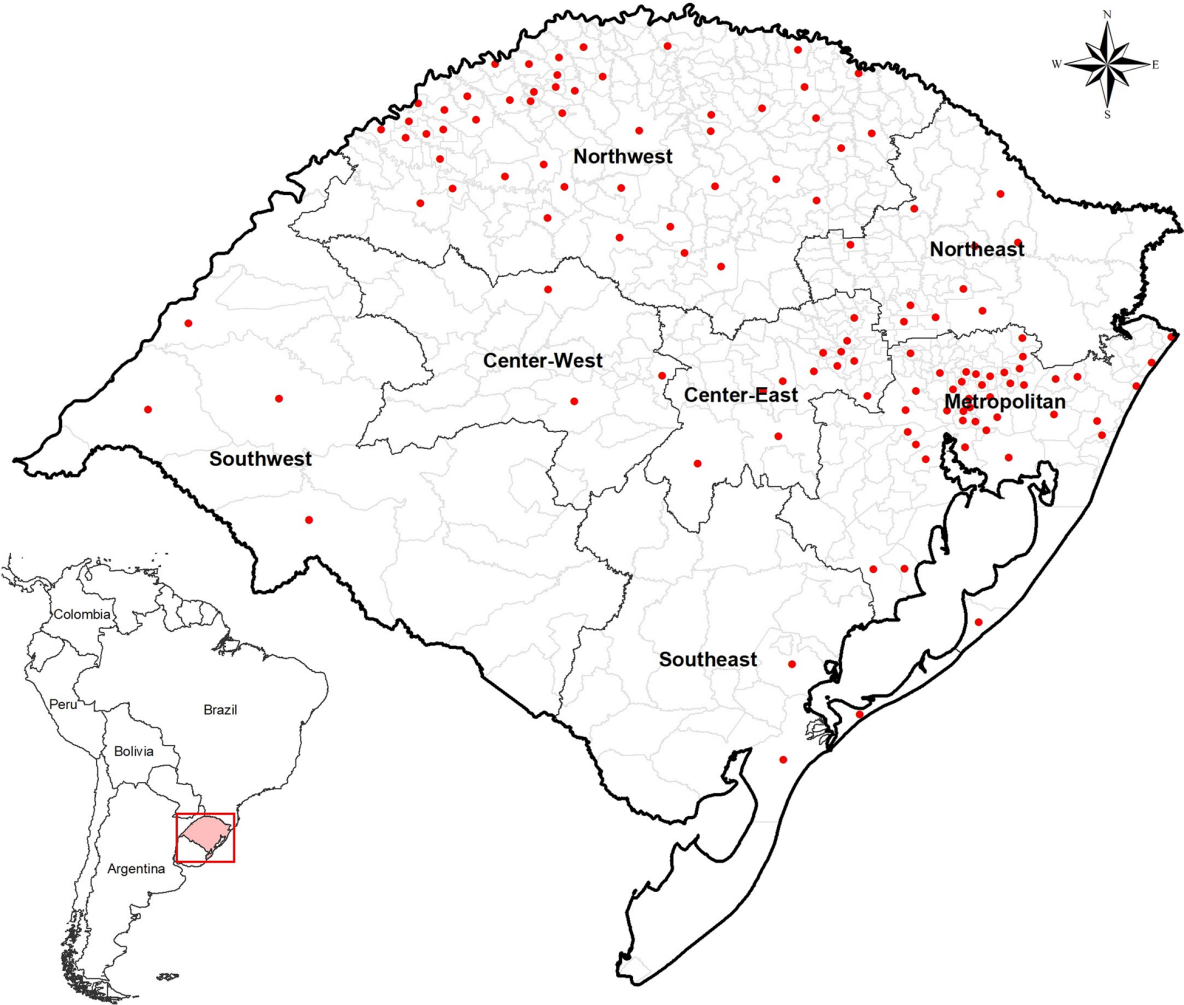
Rio Grande do Sul and its Meso-regions. Red dots indicate the cities included in the study for HLA data

We analyzed a dataset containing 97,292 potential bone marrow donors residing in the state of Rio Grande do Sul who voluntarily registered in REDOME between January 2008 and December 2012. Rio Grande do Sul, the southernmost state of Brazil, is the fourth largest state of the country with more than 11 million inhabitants distributed in 497 cities [23], 439 of which are represented in the dataset. At the time of registration in REDOME, the individuals declared their ethnicity by auto-perception based on skin color, following Brazilian Institute of Geography and Statistics (IBGE) standards. Only municipalities with 50 or more registered donors were included in the analysis. For self-reported white individuals (or Euro-descendants, EURD), 120 cities distributed across all regions of the state cities met the sample size criteria (Additional file 1), totaling 86,672 individuals (Fig. 1). On the other hand, only 19 cities had more than 50 self-reported black (or Afro-descendants, AFRD) individuals, scattered across the state and, therefore, excluded from further analyses.

Genotyping for HLA-A, -B and -DRB1 *loci* was performed at the Hospital das Clínicas de Porto Alegre (HCPA) (Luminex LABType SSO system; One Lambda, Inc., Canoga Park, CA). Due to the high polymorphism of HLA genes, complete identification of each allele is only possible through sequencing-based typing. Thus, Luminex genotyping identifies only “low resolution” allelic groups. Because they are closely located on the same chromosome, HLA alleles segregate in linkage blocks, known as haplotypes.

Information on AD were available in DATASUS (Department of Informatics of the Unified Health System, in Portuguese), a database established by the Brazilian Ministry of Health since 1991 that contains health information and statistics from all municipalities in Brazil and that is publicly available through online access (datasus.saude.gov.br/datasus). In general, the DATASUS database is fed by data sent by the municipal and state health secretariats to the Ministry of Health. Of the 497 cities of Rio Grande do Sul, 496 had information about AD hospitalizations in DATASUS database.

This study was approved by the Ethics Committee of the Research and Post-Graduation Group of the Hospital de Clínicas de Porto Alegre, under number 386.216.

### Statistical analyses

Allele and haplotype frequency estimations and tests of Hardy–Weinberg equilibrium (HWE) were performed using the GENE[RATE] tools as described elsewhere [7, 24–26]. Principal component analysis (PCA) was done for each *locus* using Rstudio (v0.98.1103) and the genetic structure was measured using the synthetic genetic structure (*SPC*) measure proposed by Xue et al. [27], as follows:

$$SPC = W_{1} \times PC_{1} + W_{2} \times PC_{2} + \cdots + W_{k} \times PC_{k}$$

where *PC* is the component score and *W* is the proportion (weight) of the component contribution. All components with an eigenvalue greater than 1 were included in the *SPC* calculation, following the Kaiser criterion [28]. Hospitalization data for RA, multiple sclerosis (MS), Crohn’s disease (CD) and leukemia for each city, comprising the period from January 1995 to December 2016, were obtained through the DATASUS system (tabnet.datasus.gov.br/). RA, MS and CD are the only AD recorded in DATASUS. The number of hospitalizations of each disease in each city was adjusted by the number of inhabitants and used as an indicator of disease prevalence (disease index, DI). Spearman’s correlation test between each allele and each disease was performed using IBM SPSS software, Version 20.0 (IBM Corp., Armonk, NY). The result obtained in the Spearman correlation test was submitted to the multiple comparison test FDR (false discovery rate) in Rstudio (v0.98.1103) using the stats (3.3.0) package.

Allele and haplotype frequency, *SPC* data as well as positive and statistically significant alleles × diseases (hereafter A*D) in the Spearman’s correlation test were spatially interpolated using the Empirical Bayesian Kriging method (EBK). For each interpolation, scatterplots were performed for the observed and predicted values and calculated their respective coefficients of determination (R^2^), Spea^r^man’s correlation coefficient (*ρ*), Spearman’s coefficient of determination (*ρ*^*2*^) and the root mean square error (RMSE). The P-values were adjusted by FDR for α = 0.05.

Cluster maps for A*D showing positive and statistically significant correlation in the Spearman test were generated through the Hot-Spot analysis using the Getis-Ord Gi* method [29, 30] based on the following formula:

$$\sqrt {\frac{DI - DImax}{DImax - DImin} \times \frac{AF - AFmax}{AFmax - AFmin}}$$

where DI is the disease index, DImax is the maximum disease index, DImin is the minimum disease index, AF is the allelic frequency, AFmax is the maximum allelic frequency and AFmin is the minimum allele frequency. All spatial analyses were performed in ArcGis v10.3.

## Results

**Fig. 2:**
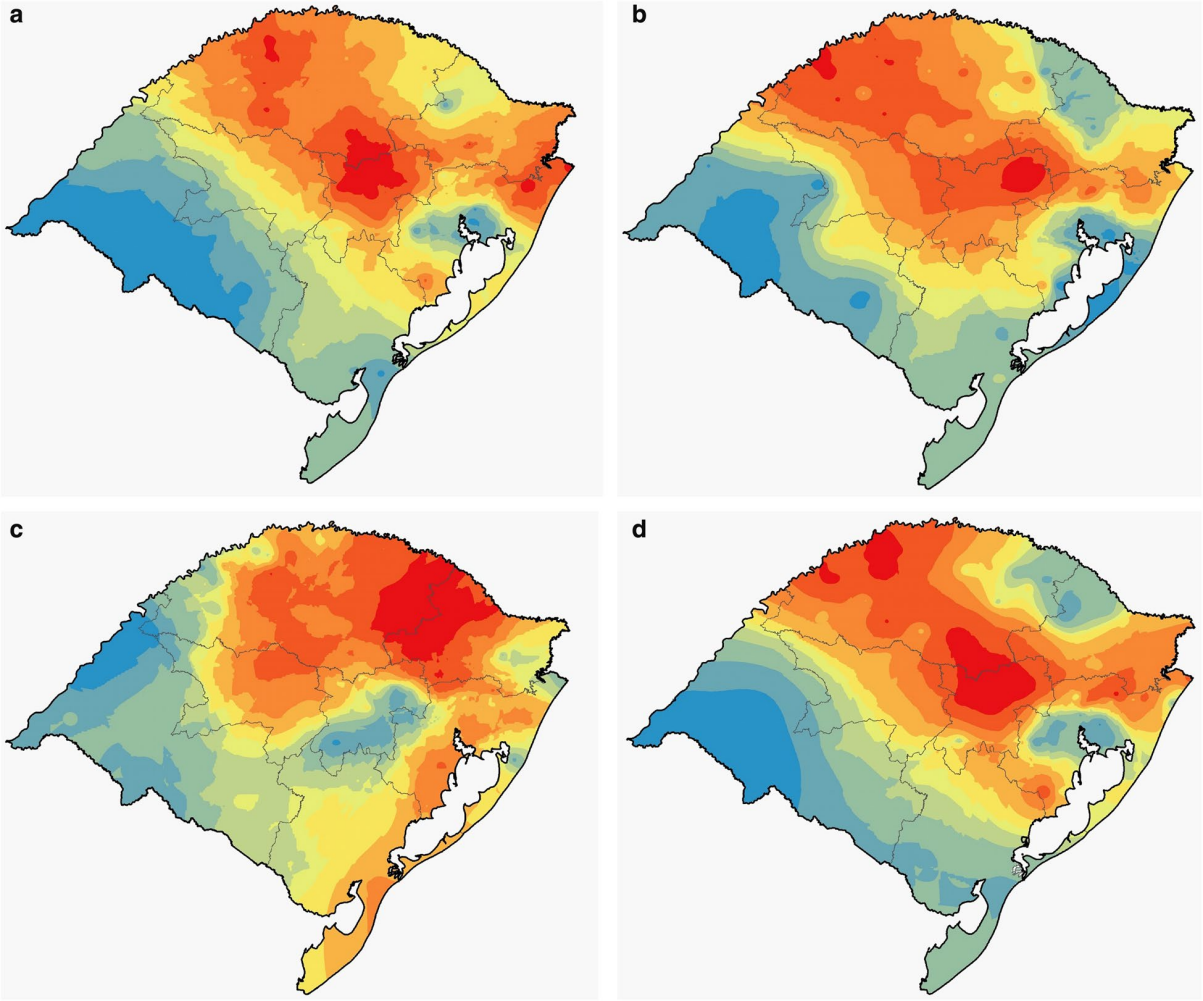
HLA heterogeneity and its genetic structure estimated by SPC and spatialized by EBK. **a** HLA-A *locus*. **b** HLA-B *locus*. **c** HLA-DRB1 *locus*. **d** HLA-A, -B and -DRB1 *loci* combined

For all cities, allele frequencies did not show deviations from the Hardy–Weinberg equilibrium (Additional file 2). Considering the whole state, the most frequent alleles for each *locus* were HLA-A*02 (27.6%), HLA-B*35 (12.4) and HLA-DRB1*07 (13.4%), with substantial allele frequency variation among cities (Additional files 2, 3). Five haplotypes reached frequencies above 2% in at least one city. Haplotype A*01 ~ B*08 ~ DRB1*03 presented the highest frequency considering the entire state (3.7%). Following allele frequencies, there was substantial variation in haplotype frequencies among cities (Additional files 4, 5). Figure 2 shows the spatial HLA genetic structure based on *SPC* in Rio Grande do Sul. HLA-A and HLA-B *loci* have a very similar structure, showing a higher differentiation between the Southwest and Metropolitan regions in relation to the Central and Northwest regions. The HLA-DRB1 *locus* presents a slightly different structure, with a higher differentiation in the Northeast. The combined data for the three *loci*, shows a very similar structure compared to HLA-A and HLA-B.

**Fig. 3:**
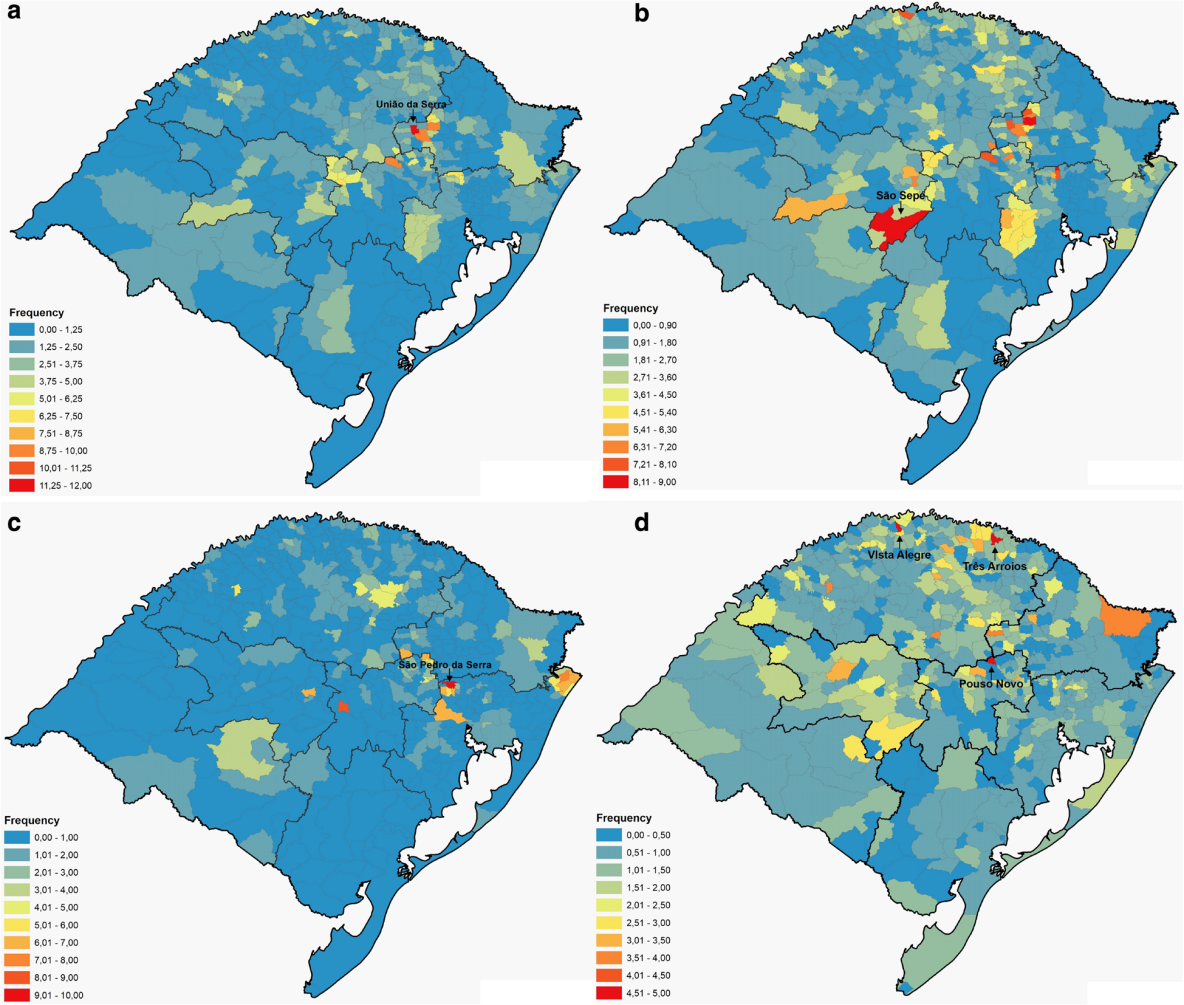
Maps of AD and leukemia prevalence in Rio Grande do Sul. **a** Rheumatoid arthritis. **b** Multiple sclerosis. **c** Crohn’s disease. **d** Leukemia. Data obtained from the DATASUS system comprising the period from January 1995 to June 2016 (tabnet.datasus.gov.br/)

**Fig. 4:**
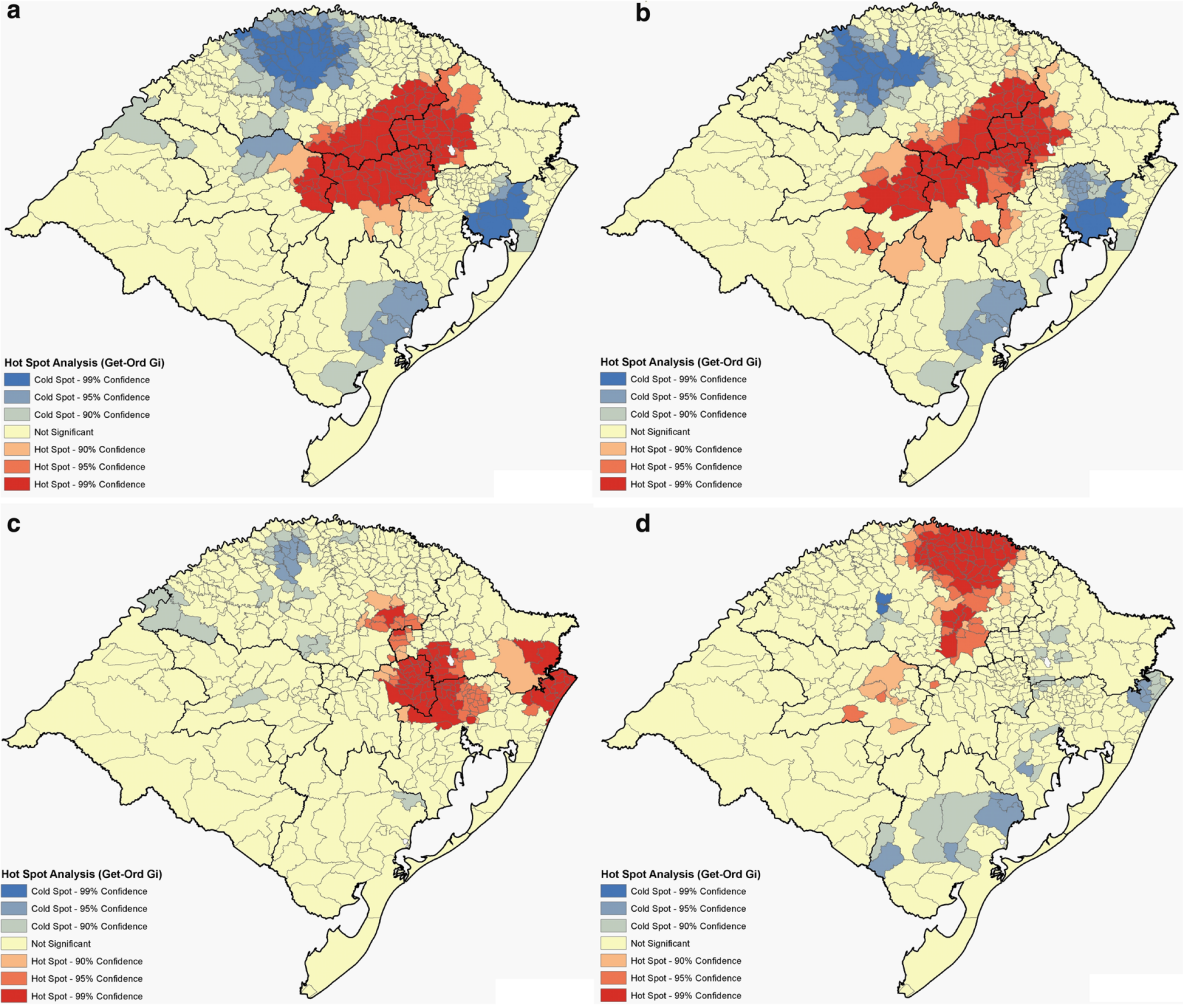
Cluster maps for AD and leukemia in Rio Grande do Sul. **a** Rheumatoid arthritis. **b** Multiple sclerosis. **c** Crohn’s disease. **d** Leukemia

Figure 3 shows the distribution of the DI, while disease prevalence is shown, for each city, in Additional file 6. The small town of União da Serra, located in the Northeast region of the state, has a population of approximately 1500 inhabitants, which is equivalent to 0.014% of the total population of the state of Rio Grande do Sul. However, this city responded to 0.162% of all hospitalizations for RA from January 1995 to December 2016 (62 hospitalizations events). Thus, when considering the number of hospitalizations by the number of inhabitants in relation to the totals for the state, União da Serra is the municipality with the highest prevalence of RA with a DI 11.6 × higher than expected. Similarly, the town of São Sepé, in the Center-East region of the state, was the city with the highest prevalence for MS, with a DI almost 9 × higher than expected. On its turn, the town of São Pedro da Serra, in the Metropolitan region, had the highest prevalence for CD, with a DI 9.5 × higher than expected. For leukemia, the towns of Pouso Novo, in the Center-East region, Vista Alegre and Três Arroios, both in the Northwest region, had a DI 4.5 × higher than the expected. For all diseases (CD, MS, RA, and leukemia) there was strong evidence for spatial clusters in DI (Fig. 4, Additional file 7, *P *< 3 × 10^−5^ in all cases). Different regions appeared as hot-spots for different diseases. The Center-East and Northeast regions behaved as hot-spots for RA and MS, while the Metropolitan and the Northwest regions were cold-spots. CD had a hot-spot cluster in the Metropolitan region and in a small area in the Northeast, while for leukemia there was a hot-spot in the extreme North of the state.

**Fig. 5:**
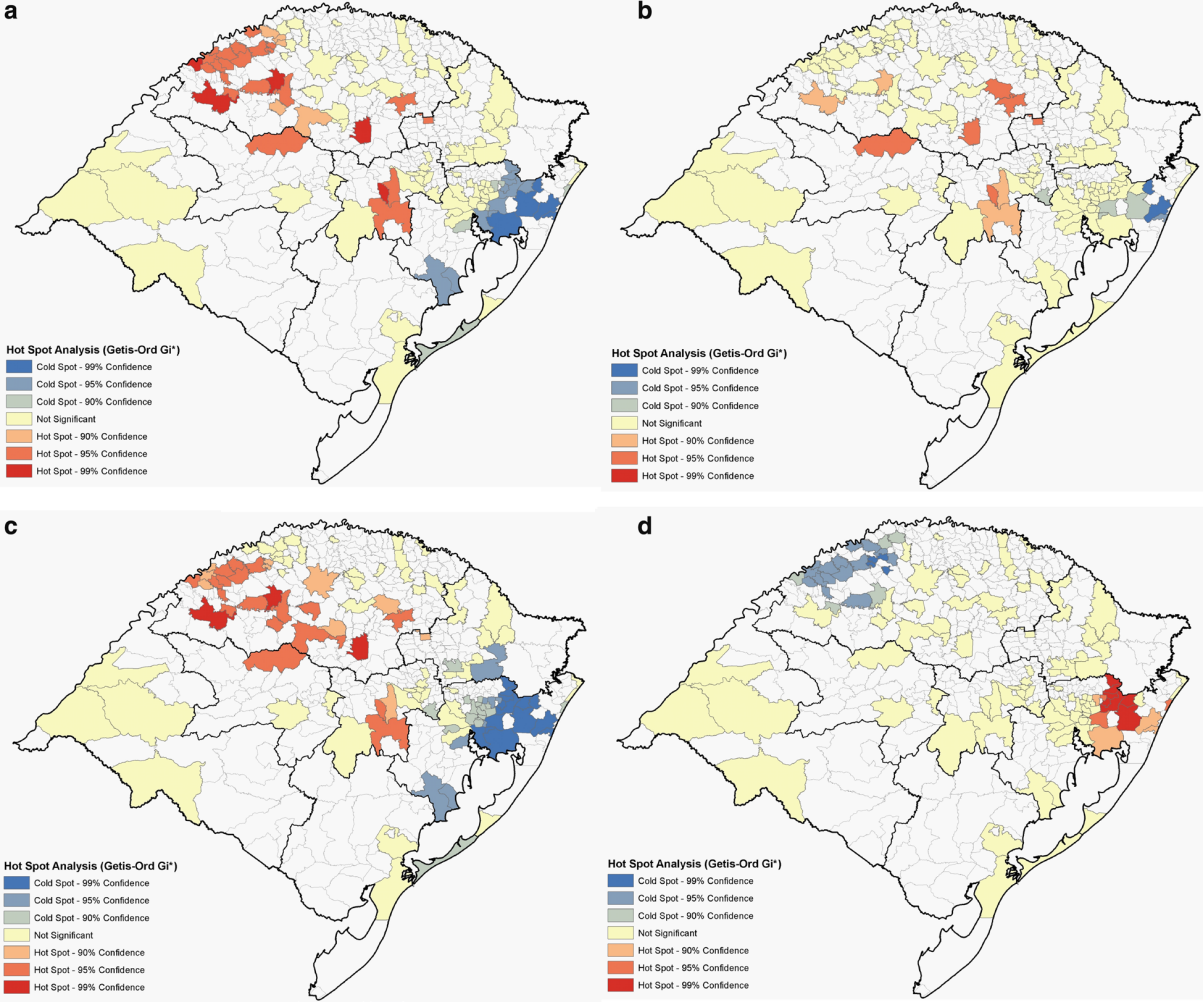
Cluster maps for allele × autoimmune disease interaction. **a** HLA-B*08 × RA. **b** HLA-DRB1*03 × RA. **c** HLA-B*08 × MS. **d** and HLA-A*29 × CD

Table 1 shows the Spearman correlation index (*ρ*) for each allele and each disease tested. Most of the statistically significant correlations found were negative. Alleles HLA-B*08 and -DRB1*03 showed a positive and significant correlation with RA; HLA-B*08 with MS; and HLA-A*29, HLA-B*38 and HLA-DRB1*01 with CD. Cluster analyses indicated a significant spatial component in A*D interaction for HLA-B*08 × RA, HLA-DRB1*03 × RA, HLA-B*08 × MS (*P *< 0.01), and HLA-A*29 × CD (*P *< 0.05), which is represented in Fig. 5 (and in Additional file 8). However, only the correlation between HLA-B*08 and RA remained significant after FDR correction. Interestingly, spatial hot-spots for A*D differ from DI hot-spots, indicating that adding genetic information on top of disease prevalence results in new insights of disease epidemiology.

**Table 1:** Spearman correlation (*ρ*) between alleles and diseases

HLA-A	RA	MS	CD	HLA-B	RA	MS	CD	HLA-DRB1	RA	MS	CD
A*01	0.124	0.072	− 0.198	B*07	0.146	0.135	− 0.079	DRB1*01	0.016	− 0.088	*0.187*
A*02	− 0.018	− 0.008	− 0.125	B*08	0.327*	*0.218*	− 0.005	DRB1*03	*0.210*	0.102	0.036
A*03	0.008	0.021	− 0.072	B*13	0.046	0.037	− 0.143	DRB1*04	0.054	0.162	− 0.249
A*11	− 0.108	− 0.118	− 0.089	B*14	− 0.077	− 0.049	0.100	DRB1*07	− 0.182	− 0.117	0.143
A*23	− 0.166	− 0.255	− 0.017	B*15	0.077	0.083	− 0.020	DRB1*08	− 0.220	− 0.168	0.066
A*24	− 0.059	− 0.084	0.036	B*18	0.169	0.056	0.027	DRB1*09	− 0.089	− 0.091	− 0.004
A*25	0.150	0.075	− 0.113	B*27	0.094	0.067	− 0.199	DRB1*10	− 0.059	− 0.008	0.101
A*26	0.045	0.002	0.149	B*35	0.029	− 0.013	0.125	DRB1*11	0.046	0.032	0.112
A*29	− 0.207	− 0.203	*0.197*	B*37	− 0.178	− 0.157	0.028	DRB1*12	− 0.121	− 0.150	− 0.180
A*30	− 0.131	− 0.055	0.172	B*38	− 0.038	− 0.111	*0.195*	DRB1*13	− 0.115	− 0.060	− 0.094
A*31	− 0.040	− 0.085	− 0.003	B*39	− 0.017	− 0.060	0.158	DRB1*14	− 0.066	− 0.077	− 0.005
A*32	0.093	0.133	− 0.119	B*40	− 0.034	0.001	− 0.248	DRB1*15	0.047	0.030	− 0.015
A*33	− 0.011	0.034	0.088	B*41	− 0.156	− 0.136	− 0.096	DRB1*16	− 0.230	− 0.265	− 0.084
A*34	− 0.313	− 0.229	0.065	B*42	− 0.111	− 0.047	0.167				
A*36	− 0.149	− 0.123	0.149	B*44	− 0.179	− 0.089	0.022				
A*43	0.020	0.073	− 0.054	B*45	− 0.211	− 0.178	0.024				
A*66	− 0.110	− 0.090	0.014	B*46	0.073	0.052	0.038				
A*68	0.056	0.043	0.149	B*47	− 0.155	− 0.168	0.028				
A*69	− 0.126	− 0.120	− 0.016	B*48	− 0.218	− 0.120	0.049				
A*74	− 0.197	− 0.185	0.089	B*49	− 0.123	− 0.148	0.004				
A*80	− 0.143	− 0.072	− 0.016	B*50	− 0.371	− 0.306	0.088				
				B*51	− 0.007	0.006	0.031				
				B*52	0.019	0.026	0.150				
				B*53	− 0.200	− 0.148	0.078				
				B*54	0.091	0.101	0.078				
				B*55	− 0.120	− 0.188	− 0.067				
				B*56	− 0.015	0.052	0.066				
				B*57	0.010	0.027	− 0.077				
				B*58	− 0.191	− 0.182	0.076				
				B*59	− 0.099	− 0.083	− 0.001				
				B*67	− 0.010	0.004	− 0.042				
				B*73	− 0.106	− 0.069	0.005				
				B*78	− 0.050	0.002	− 0.006				
				B*81	− 0.289	− 0.242	0.012				
				B*82	− 0.098	− 0.064	0.106				

*RA* Rheumatoid arthritis, *MS* multiple sclerosis, *CD* Crohn’s disease

Italic: Positive correlation; *P* ≤ 0.05

*Remained significant even after correction by FDR

The Spearman’s correlation coefficient (*ρ*), Spearman’s coefficient of determination (*ρ*^*2*^) and the root mean square error (RMSE) for all observed and interpolated values of each EBK map (allelic and haplotypic frequencies, SPC analysis and A*D correlation analysis) are presented in the Additional file 9. The lowest correlation coefficient was 0.1686 for allele HLA-B*56 and the highest was 0.9978 for HLA-A*30. The lowest RMSE found was < 0.0001 for allele HLA-B*27, while the highest was 0.1628 for SPC HLA-DRB1. Except for the HLA-A*68 and HLA-B*56 allele frequency maps, all interpolations were statistically significant, even after FDR correction. Scatterplots and their respective coefficient of determination (R^2^) for each interpolated map are presented in Additional file 10.

## Discussion

This is the first study to perform spatial analysis of HLA genetic structure, correlating HLA population genetics data with epidemiological data on AD. Figure 1 shows the HLA structure of the bone marrow donor population in Rio Grande do Sul based on the principal component analysis (PCA) of HLA allele frequencies. Visually, HLA-A, HLA-B and the combined data for the three *loci* showed a very similar structure, presenting a higher differentiation between the Southwest and Metropolitan regions in relation to the Central and Northwest regions.

PCA is a very useful tool in the investigation of population structure, but sampling strategy and the amount of data may impact its results [31]. In this study, only self-reported white individuals were included due to sample size limitations (only 19 cities had more than 50 self-reported black individuals, with little coverage in the state). It is unlikely that this had a major impact on the characterization of HLA genetic structure in Rio Grande do Sul as a whole, given that more than 80% of the population of Rio Grande do Sul is self-declared white [23], and more than 90% of REDOME donors in Rio Grande do Sul declare themselves white at the time of registration. However, given that there are differences in AD prevalence between black and white individuals [32, 33, see 34 for review], an important step forward would be characterizing geographic clusters of AD in the black population of this state and its relationship with the clusters identified in this study.

Among the classic HLA genes, HLA-A is more sensitive to demographic processes, such as genetic drift, because it is less affected by balancing selection [35, 36]. In this sense, the differentiation between the Southwest, Southeast and the Metropolitan regions, on one hand, compared to the Central and Northwest regions, on the other hand (Fig. 1), may mirror the colonization history of Rio Grande do Sul (Additional file 11). In these former regions, Portuguese and Spanish individuals were the major settlers since the early eighteenth century, with the later arrival of African slaves, mostly in the Pelotas (Southeast) region. On the other hand, Germans (1824), Poles (1871) and Italians (1875) were major ethnicities settling the Central and Northern regions [37, 38].

Specific spatialization and interpolation techniques may influence the geographic trends shown by the data. In this study, allele and haplotype frequencies as well as A*D positive and statistically significant correlations were spatialized by the EBK method (Additional files 3, 5). This method was chosen because we had only 120 points to represent the 496 municipalities of Rio Grande do Sul. Kriging is a probabilistic predictor, thus assuming a statistical model for the data, being able to quantify the uncertainty associated with the values predicted from the standard errors. This method uses a semivariogram—a function of distance and direction separating two locations—to quantify the spatial dependence on the data. EBK differs from classical kriging by using many semivariogram models rather than using only a single model. For each repetition, the semivariogram is used to simulate a new set of values at the input sites; then the simulated data are used to estimate a new semivariogram and its weight. Thus, predicted values and standard errors are inferred for the non-sampled regions using these weights [39].

All interpolated maps showed correlation between observed and interpolated values. The lowest correlation coefficients were typically observed in alleles with low frequencies, and where the sampling is consequently smaller. It is important to note that the alleles that showed a positive and significant correlation with AD (HLA-A*29, HLA-B*08, HLA-B*38, HLA-DRB1*01 e HLA-DRB1*03) presented a correlation coefficient for interpolation ranging from 0.513 to 0.982 and maximum RMSD of 0.017 (Additional file 9). These values indicate that the interpolation method and the analyses performed are consistent.

The Hot-Spot analysis (Getis-Ord Gi*) revealed geographic clusters of AD (RA, MS and CD) and leukemia in Rio Grande do Sul, indicating that neighbor regions should have similar disease prevalence (Additional file 7). On the other hand, our analysis also revealed spatial clusters of A*D, even though both spatial clusters had little overlap (Figs. 4, 5). A genetic cluster can be defined as a group of genetically divergent individuals that arises when gene flow is impeded by physical or cultural barriers [40]. Evolutionary forces such as the founder’s effect and low immigration may reinforce genetic backgrounds that pre-dispose to some genetic conditions. One interpretation for the little overlap between DI and A*D is that while DI spatial clustering is dominated by shared environmental and genetic (non-HLA) affecting disease status, A*D spatial clusters indicate a more important role for the common HLA genetic background (through specific “risk” alleles) for these diseases. As a result, cities having a high frequency of HLA-B*08, for example, will have a higher chance of having high DI for RA even if this city is distant from the DI spatial cluster disconsidering HLA information.

AD are heterogeneous in regard to prevalence, clinical manifestations, and pathogenesis, being caused by an immune response against constituents of the body’s own tissues. Specific HLA alleles can predispose to several AD [10, 11]. Indeed, some of the positive and significant correlations between HLA alleles and AD found in our study have already been described in case–control studies. Han et al. [41] established a relationship between HLA-B*08 and RA subtype anti-citrullinated-protein-autoantibody-negative (ACPA^−^ or seronegative) in a study involving 2406 ACPA^−^ case and 13,930 control individuals. Alsaied et al. [42] found an association between HLA-DRB1*03 and juvenile RA in Kuwaiti Arab children, and Manivel et al. [43] established an association between HLA-DRB1*03 and RA subtype anti-CII (anti fibrillar collagen type II) in the Swedish population. On the contrary, Lysandropoulos et al. [44] tested the relation between MS and HLA-B*08, but the result was inconclusive. Concerning CD, Goyette et al. [45] found a significant association with DRB1*01, but there are no other studies correlating CD and HLA-A*29 and HLA-B*38. Differently from our findings, Konda Mohan et al. [46] ] and Bizzari et al. [47] indicated a protective role for HLA-DRB1*03 for RA in Indian and Arabic populations, respectively. These results may indicate that some relationships between AD and HLA background may be population-specific, which highlights the potential of spatial analyses to identify small-scale A*D clusters in populations from a similar background.

Nonetheless, some limitations of this study should be taken into account: the bone marrow donor individuals are not the same reported in the DATASUS system for the mentioned diseases, in addition to the already mentioned limiting number of cities having enough sample size for allele frequency analysis. Besides, the data used in the DATASUS system refers to the number of hospitalizations for each disease and, because we use data of chronic disease, the same person may hospitalize more than once for the same condition. However, spatially studies can serve at least as preliminary models of genetic × disease interaction to guide further investigations and promote public health actions.

Understanding the demographic processes that affect the genetic diversity of human populations at a spatial scale can be useful in public health policies in the present. The study of the HLA diversity at the population level is invaluable in disease-association studies and in the effectiveness of bone marrow transplantation programs. Thus, the results presented in this study, such as the heterogeneous genetic structure and the A*D spatial correlations, demonstrate the importance of the integrated use of large databases with spatial-specific analysis approaches, and may indicate the need to implement space-specific interventions to guide policy planning and decision making in public health.

Despite all the potential use of GIS, this tool is still underutilized in public health centers around the world. Georeferencing is an essential first step in making it possible to analyze public health data geographically [48]. Through the georeferencing of public health data it is possible to perform a spatial analysis for public health systems [49]. The correct use of GIS can inform and educate professionals and the public, give more power to decision making at all levels, assist in planning and adjusting clinical and cost-effective actions, monitor and analyze changes in health levels and exposure to disease [50].

## Conclusions

In this study, we used GIS tools to evaluate the spatial correlation between HLA alleles and occurrence of AD in Rio Grande do Sul, based on data available from governmental health agencies. To the best of our knowledge, this is the first study that investigates the spatial correlation between genetic data and AD occurrence. The results presented in this study highlights the potential of spatial analyses to identify the interaction between alleles and diseases in populations from a similar background. The use of information from large databases such as REDOME and DATASUS together with georeferencing tools can help in the identification of useful markers in population genetics that may confer resistance or susceptibility to diseases. Genetic mapping of populations and the spatial analyzes such as those performed in this work have great economic relevance and can be very useful in the formulation of public health campaigns and policies, contributing to the planning and adjustment of clinical actions, as well as informing and educating professionals and the population.

## Additional files

**Additional file 1.** Sample size.

**Additional file 2.** Allelic frequencies, genetic diversity and Hardy–Weinberg equilibrium.

**Additional file 3.** Allelic frequency maps.

**Additional file 4.** Haplotype frequencies.

**Additional file 5.** Haplotype frequencies maps.

**Additional file 6.** Prevalence of hospitalizations for each disease in the cities of Rio Grande do Sul.

**Additional file 7.** Prevalence and cluster maps for each disease.

**Additional file 8.** Correlation maps between alleles and autoimmune diseases.

**Additional file 9.** Spearman’s determination coefficient, Spearman’s correlation coefficient and root mean square error for each interpolated map.

**Additional file 10.** Scatterplot and coefficient of determination (R^2^) for each interpolated map.

**Additional file 11.** Meso-regions of Rio Grande do Sul and its colonization regions.

## Abbreviations

MHC: Major histocompatibility complex
HLA: Human leukocyte antigen
HSCT: hematopoietic stem cell transplantation
AD: autoimmune diseases
RA: Rheumatoid arthritis
GIS: geographic information system
REDOME: Brazilian bone marrow donor registry
RS: Rio Grande do Sul
IBGE: Brazilian institute of geography and statistics
EURD: Euro-descendants
AFRD: Afro-descendants
HCPA: Hospital de Clínicas de Porto Alegre
DATASUS: Department of informatics of the unified health system
HWE: Hardy–Weinberg equilibrium
PCA: principal component analysis
SPC: synthetic genetic structure
MS: multiple sclerosis
CD: Crohn’s disease
DI: disease index
FDR: false discovery ratio
EBK: Empirical Bayesian kriging
