# Supplementary material for: Spatial analyzes of HLA data in Rio Grande do Sul, south Brazil: genetic structure and possible correlation with autoimmune diseases
Source: Int J Health Geogr. 2018 Sep 14;17:34. doi: 10.1186/s12942-018-0154-8 (PMC6137739; doi:10.1186/s12942-018-0154-8)
Supplement: Supplementary file 1 — Additional file 1. Sample size. [file 12942_2018_154_MOESM1_ESM.docx]

**Additional file 1 – Sample size**

| Town | Ethnicity (auto-perception based on skin color) | | | | | Total |
| --- | --- | --- | --- | --- | --- | --- |
|  | ASID | EURD | NAAM | MIXD | AFRD |  |
| Agua Santa | 0 | 4 | 0 | 0 | 0 | 4 |
| Agudo | 0 | **99** | 0 | 2 | 2 | 103 |
| Ajuricaba | 0 | 19 | 0 | 0 | 1 | 20 |
| Alecrim | 0 | 8 | 0 | 0 | 0 | 8 |
| Alegrete | 1 | **1259** | 0 | 11 | 24 | 1295 |
| Alegria | 0 | 47 | 0 | 3 | 1 | 51 |
| Alpestre | 0 | 1 | 0 | 0 | 0 | 1 |
| Alto Alegre | 0 | 26 | 0 | 0 | 0 | 26 |
| Alto Feliz | 0 | 10 | 0 | 0 | 0 | 10 |
| Alvorada | 3 | **841** | 2 | 22 | **153** | 1021 |
| Amaral Ferrador | 0 | 1 | 0 | 0 | 1 | 2 |
| Ametista do Sul | 0 | **62** | 0 | 0 | 13 | 75 |
| André da Rocha | 0 | 1 | 0 | 0 | 0 | 1 |
| Anta Gorda | 0 | 9 | 0 | 0 | 0 | 9 |
| Antônio Prado | 0 | 24 | 0 | 0 | 0 | 24 |
| Arambare | 0 | **60** | 1 | 2 | 6 | 69 |
| Ararica | 0 | 46 | 0 | 1 | 1 | 48 |
| Aratiba | 0 | **130** | 0 | 0 | 0 | 130 |
| Arroio do Meio | 0 | **86** | 0 | 1 | 1 | 88 |
| Arroio do Padre | 0 | 1 | 0 | 0 | 0 | 1 |
| Arroio do Sal | 1 | **507** | 0 | 0 | 43 | 551 |
| Arroio do Tigre | 0 | 5 | 0 | 0 | 0 | 5 |
| Arroio dos Ratos | 0 | 40 | 1 | 0 | 4 | 45 |
| Arroio Grande | 0 | 4 | 0 | 0 | 0 | 4 |
| Arvorezinha | 0 | 9 | 0 | 0 | 0 | 9 |
| Augusto Pestana | 0 | **65** | 0 | 0 | 5 | 70 |
| Aurea | 0 | 39 | 0 | 0 | 0 | 39 |
| Bagé | 0 | 40 | 0 | 1 | 6 | 47 |
| Balneário Pinhal | 0 | 21 | 0 | 1 | 1 | 23 |
| Barão | 0 | 10 | 0 | 0 | 0 | 10 |
| Barão de Cotegipe | 0 | 4 | 0 | 0 | 0 | 4 |
| Barão do Triunfo | 0 | 8 | 0 | 0 | 0 | 8 |
| Barra do Guarita | 0 | 11 | 0 | 0 | 0 | 11 |
| Barra o Quarai | 0 | 7 | 0 | 0 | 0 | 7 |
| Barra do Ribeiro | 0 | 43 | 0 | 1 | 1 | 45 |
| Barra do Rio Azul | 0 | 1 | 0 | 0 | 0 | 1 |
| Barra Funda | 0 | 7 | 0 | 0 | 1 | 8 |
| Barracão | 0 | 4 | 0 | 0 | 0 | 4 |
| Benjamin Constant do Sul | 0 | 2 | 0 | 0 | 0 | 2 |
| Bento Goncalves | 0 | **248** | 0 | 0 | 9 | 257 |
| Boa Vista das Missões | 0 | 2 | 0 | 0 | 0 | 2 |
| Town | Ethnicity (auto-perception based on skin color) | | | | | Total |
|  | ASID | EURD | NAAM | MIXD | AFRD |  |
| Boa Vista do Buricá | 1 | **173** | 0 | 0 | 3 | 177 |
| Boa Vista do Sul | 0 | 5 | 0 | 0 | 0 | 5 |
| Bom Jesus | 0 | 14 | 0 | 4 | 0 | 18 |
| Bom Princípio | 0 | 45 | 0 | 0 | 1 | 46 |
| Bom Progresso | 0 | 2 | 0 | 0 | 0 | 2 |
| Bom Retiro do Sul | 0 | 25 | 0 | 0 | 0 | 25 |
| Boqueirão do Leão | 0 | 2 | 0 | 0 | 0 | 2 |
| Bossoroca | 0 | 2 | 0 | 0 | 1 | 3 |
| Bozano | 0 | 3 | 0 | 0 | 0 | 3 |
| Braga | 0 | 8 | 0 | 0 | 0 | 8 |
| Brochier | 0 | 6 | 0 | 0 | 0 | 6 |
| Butiá | 1 | 26 | 0 | 0 | 4 | 31 |
| Caçapava do Sul | 0 | 10 | 0 | 0 | 1 | 11 |
| Cacequi | 0 | 2 | 0 | 0 | 0 | 2 |
| Cachoeira do Sul | 0 | **58** | 0 | 0 | 6 | 64 |
| Cachoeirinha | 0 | **1139** | 0 | 11 | **71** | 1221 |
| Cacique Doble | 0 | 3 | 0 | 0 | 0 | 3 |
| Caibaté | 0 | **88** | 0 | 0 | 0 | 88 |
| Caiçara | 0 | 4 | 0 | 0 | 0 | 4 |
| Camaquã | 0 | **114** | 0 | 3 | 9 | 126 |
| Camargo | 0 | 1 | 0 | 0 | 0 | 1 |
| Cambara do Sul | 0 | 17 | 0 | 0 | 0 | 17 |
| Campestre da Serra | 0 | **92** | 0 | 0 | 6 | 98 |
| Campina das Missões | 0 | **78** | 0 | 0 | 1 | 79 |
| Campinas do Sul | 0 | 22 | 0 | 0 | 0 | 22 |
| Campo Bom | 1 | **612** | 0 | 2 | 19 | 634 |
| Campo Novo | 0 | **177** | 0 | 1 | 15 | 193 |
| Campos Borges | 0 | 3 | 0 | 0 | 0 | 3 |
| Candelária | 0 | 28 | 0 | 0 | 2 | 30 |
| Candido Godói | 0 | **172** | 0 | 2 | 2 | 176 |
| Candiota | 0 | 3 | 0 | 0 | 0 | 3 |
| Canela | 0 | **51** | 0 | 0 | 5 | 56 |
| Canguçu | 0 | 30 | 0 | 1 | 2 | 33 |
| Canoas | 4 | **3820** | 0 | **51** | **268** | 4143 |
| Canudos do Vale | 0 | 4 | 0 | 0 | 0 | 4 |
| Capão da Canoa | 0 | **104** | 0 | 1 | 9 | 114 |
| Capão do Leão | 0 | 44 | 0 | 0 | 2 | 46 |
| Capela de Santana | 0 | 25 | 0 | 3 | 2 | 30 |
| Capitão | 0 | 4 | 0 | 0 | 0 | 4 |
| Capivari Do Sul | 0 | 11 | 0 | 0 | 0 | 11 |
| Caraá | 0 | 31 | 0 | 0 | 1 | 32 |
| Carazinho | 0 | **314** | 0 | 0 | 6 | 320 |
| Town | Ethnicity (auto-perception based on skin color) | | | | | Total |
|  | ASID | EURD | NAAM | MIXD | AFRD |  |
| Carlos Barbosa | 0 | 33 | 0 | 0 | 1 | 34 |
| Casca | 0 | 10 | 0 | 0 | 0 | 10 |
| Caseiros | 0 | 3 | 0 | 0 | 1 | 4 |
| Catuípe | 0 | **52** | 0 | 0 | 1 | 53 |
| Caxias do Sul | **68** | **4959** | 0 | **122** | **65** | 5214 |
| Centenário | 0 | 1 | 0 | 0 | 1 | 2 |
| Cerro Branco | 0 | 1 | 0 | 0 | 0 | 1 |
| Cerro Grande do Sul | 0 | 12 | 0 | 0 | 1 | 13 |
| Cerro Largo | 0 | **59** | 0 | 0 | 3 | 62 |
| Chapada | 0 | 12 | 0 | 0 | 1 | 13 |
| Charqueadas | 0 | **107** | 0 | 2 | 7 | 116 |
| Charrua | 0 | 8 | 0 | 0 | 0 | 8 |
| Chiapeta | 0 | 47 | 0 | 1 | 0 | 48 |
| Chuí | 0 | 4 | 0 | 0 | 0 | 4 |
| Chuvisca | 0 | 1 | 0 | 0 | 0 | 1 |
| Cidreira | 0 | 19 | 0 | 1 | 0 | 20 |
| Ciríaco | 0 | 2 | 0 | 0 | 0 | 2 |
| Colinas | 0 | 20 | 0 | 0 | 0 | 20 |
| Condor | 0 | 25 | 0 | 0 | 0 | 25 |
| Constantina | 0 | 4 | 0 | 0 | 0 | 4 |
| Coqueiro Baixo | 0 | 1 | 0 | 0 | 0 | 1 |
| Coqueiros Do Sul | 0 | 7 | 0 | 0 | 0 | 7 |
| Coronel Barros | 0 | 3 | 0 | 0 | 0 | 3 |
| Coronel Bicaco | 0 | 8 | 0 | 0 | 0 | 8 |
| Cotiporã | 0 | 7 | 0 | 0 | 0 | 7 |
| Coxilha | 0 | 41 | 1 | 0 | 8 | 50 |
| Crissiumal | 0 | **277** | 0 | 0 | 0 | 277 |
| Cristal | 0 | 6 | 0 | 0 | 0 | 6 |
| Cruz Alta | 4 | **1977** | 0 | 15 | **80** | 2076 |
| Cruzeiro do Sul | 0 | **79** | 0 | 0 | 3 | 82 |
| David Canabarro | 0 | 2 | 0 | 0 | 0 | 2 |
| Derrubadas | 0 | 12 | 0 | 0 | 0 | 12 |
| Dezesseis de Novembro | 0 | 2 | 0 | 0 | 0 | 2 |
| Dilermando de Aguiar | 0 | 1 | 0 | 0 | 0 | 1 |
| Dois Irmãos | 0 | **163** | 0 | 0 | 3 | 166 |
| Dois Irmãos das Missões | 0 | 1 | 0 | 0 | 0 | 1 |
| Dois Lajeados | 0 | 1 | 0 | 0 | 0 | 1 |
| Dom Feliciano | 0 | 5 | 0 | 0 | 0 | 5 |
| Dom Pedrito | 0 | 8 | 0 | 0 | 0 | 8 |
| Dom Pedro de Alcântara | 0 | 15 | 0 | 0 | 1 | 16 |
| Dona Francisca | 0 | 2 | 0 | 0 | 0 | 2 |
| Doutor Mauricio Cardoso | 0 | **172** | 0 | 0 | 5 | 177 |
| Town | Ethnicity (auto-perception based on skin color) | | | | | Total |
|  | ASID | EURD | NAAM | MIXD | AFRD |  |
| Doutor Ricardo | 0 | 1 | 0 | 0 | 0 | 1 |
| Eldorado do Sul | 1 | **175** | 0 | 5 | 10 | 191 |
| Encantado | 0 | **214** | 0 | 1 | 9 | 224 |
| Encruzilhada do Sul | 0 | 9 | 0 | 0 | 0 | 9 |
| Engenho Velho | 0 | 3 | 0 | 0 | 0 | 3 |
| Entre Ijuís | 0 | 15 | 0 | 0 | 0 | 15 |
| Entre Rios do Sul | 0 | 43 | 0 | 1 | 5 | 49 |
| Erebango | 0 | 1 | 0 | 0 | 0 | 1 |
| Erechim | 0 | **663** | 0 | 0 | **58** | 721 |
| Ernestina | 0 | 6 | 0 | 0 | 0 | 6 |
| Erval Seco | 0 | 5 | 0 | 0 | 0 | 5 |
| Esmeralda | 0 | 19 | 0 | 0 | 0 | 19 |
| Esperança do Sul | 1 | 18 | 0 | 0 | 0 | 19 |
| Espumoso | 0 | **107** | 0 | 1 | 6 | 114 |
| Estação | 0 | 13 | 0 | 0 | 0 | 13 |
| Estancia Velha | 3 | **883** | 0 | 9 | 17 | 912 |
| Esteio | 0 | **654** | 0 | 7 | 38 | 699 |
| Estrela | 2 | **799** | 1 | 10 | 25 | 837 |
| Estrela Velha | 0 | 2 | 0 | 0 | 0 | 2 |
| Eugenio de Castro | 0 | 2 | 0 | 0 | 0 | 2 |
| Fagundes Varela | 0 | 2 | 0 | 0 | 0 | 2 |
| Farroupilha | 4 | **2347** | 0 | 8 | **137** | 2496 |
| Faxinal do Soturno | 0 | 4 | 0 | 0 | 0 | 4 |
| Faxinalzinho | 0 | 1 | 0 | 0 | 0 | 1 |
| Fazenda Vilanova | 0 | 8 | 0 | 0 | 0 | 8 |
| Feliz | 0 | 22 | 0 | 0 | 0 | 22 |
| Flores da Cunha | 0 | **56** | 0 | 0 | 0 | 56 |
| Fontoura Xavier | 0 | 4 | 0 | 0 | 1 | 5 |
| Forquetinha | 0 | 6 | 0 | 0 | 0 | 6 |
| Fortaleza dos Valos | 0 | 45 | 0 | 0 | 3 | 48 |
| Frederico Westphalen | 0 | 30 | 0 | 0 | 0 | 30 |
| Garibaldi | 0 | **65** | 0 | 0 | 3 | 68 |
| Gaurama | 0 | 7 | 0 | 0 | 0 | 7 |
| General Câmara | 0 | 14 | 0 | 0 | 2 | 16 |
| Gentil | 0 | 3 | 0 | 0 | 0 | 3 |
| Getúlio Vargas | 0 | **90** | 0 | 0 | 7 | 97 |
| Giruá | 0 | 15 | 0 | 0 | 1 | 16 |
| Glorinha | 0 | 40 | 0 | 0 | 3 | 43 |
| Gramado | 0 | 34 | 0 | 1 | 1 | 36 |
| Gramado Dos Loureiros | 0 | 2 | 0 | 0 | 0 | 2 |
| Gramado Xavier | 0 | 2 | 0 | 0 | 0 | 2 |
| Gravataí | 8 | **3217** | 1 | 20 | **267** | 3513 |
| Town | Ethnicity (auto-perception based on skin color) | | | | | Total |
|  | ASID | EURD | NAAM | MIXD | AFRD |  |
| Guabiju | 0 | 2 | 0 | 0 | 0 | 2 |
| Guaíba | 0 | **469** | 0 | 5 | **58** | 532 |
| Guaporé | 0 | 36 | 0 | 1 | 1 | 38 |
| Guarani Das Missões | 0 | 4 | 0 | 0 | 0 | 4 |
| Harmonia | 0 | 14 | 0 | 0 | 0 | 14 |
| Herval | 0 | 2 | 0 | 0 | 0 | 2 |
| Herveiras | 0 | 1 | 0 | 0 | 0 | 1 |
| Horizontina | 0 | 27 | 0 | 0 | 1 | 28 |
| Hulha Negra | 0 | 2 | 0 | 0 | 0 | 2 |
| Humaitá | 0 | **58** | 0 | 0 | 2 | 60 |
| Ibarama | 0 | 2 | 0 | 0 | 0 | 2 |
| Ibiaçá | 0 | 19 | 0 | 0 | 0 | 19 |
| Ibiraiaras | 0 | 2 | 0 | 0 | 0 | 2 |
| Ibirapuita | 0 | 8 | 0 | 0 | 0 | 8 |
| Ibiruba | 0 | **93** | 0 | 1 | 0 | 94 |
| Igrejinha | 0 | **279** | 0 | 1 | 5 | 285 |
| Ijuí | 1 | **561** | 0 | 3 | 25 | 590 |
| Imbé | 0 | 36 | 0 | 1 | 1 | 38 |
| Imigrante | 0 | 14 | 0 | 0 | 0 | 14 |
| Independência | 0 | 14 | 0 | 0 | 0 | 14 |
| Inhacorá | 0 | 2 | 0 | 0 | 1 | 3 |
| Ipê | 0 | 8 | 0 | 0 | 2 | 10 |
| Ipiranga Do Sul | 0 | 5 | 0 | 0 | 0 | 5 |
| Irai | 0 | 1 | 0 | 0 | 0 | 1 |
| Itaara | 0 | 1 | 0 | 0 | 0 | 1 |
| Itapuca | 0 | 1 | 0 | 0 | 0 | 1 |
| Itaqui | 0 | **142** | 0 | 0 | 4 | 146 |
| Itati | 0 | 3 | 0 | 0 | 0 | 3 |
| Ivoti | 1 | **325** | 0 | 0 | 5 | 331 |
| Jaboticaba | 0 | 6 | 0 | 0 | 0 | 6 |
| Jacutinga | 0 | **154** | 0 | 0 | 5 | 159 |
| Jaguarão | 0 | 12 | 0 | 0 | 0 | 12 |
| Jaguari | 0 | 6 | 0 | 0 | 0 | 6 |
| Jóia | 0 | 3 | 0 | 0 | 0 | 3 |
| Júlio de Castilhos | 0 | 33 | 0 | 0 | 2 | 35 |
| Lagoa dos Três Cantos | 0 | 3 | 0 | 0 | 1 | 4 |
| Lagoa Vermelha | 0 | **77** | 0 | 0 | 0 | 77 |
| Lagoão | 0 | 1 | 0 | 0 | 0 | 1 |
| Lajeado | 0 | **1484** | 0 | 4 | 31 | 1519 |
| Lavras do Sul | 0 | 3 | 0 | 0 | 0 | 3 |
| Lindolfo Collor | 0 | 37 | 0 | 1 | 0 | 38 |
| Linha Nova | 0 | 1 | 0 | 0 | 0 | 1 |
| Town | Ethnicity (auto-perception based on skin color) | | | | | Total |
|  | ASID | EURD | NAAM | MIXD | AFRD |  |
| Machadinho | 0 | 7 | 0 | 0 | 0 | 7 |
| Manoel Viana | 0 | 11 | 0 | 0 | 1 | 12 |
| Maquine | 0 | 14 | 0 | 0 | 0 | 14 |
| Marata | 0 | 3 | 0 | 0 | 0 | 3 |
| Marau | 0 | **188** | 0 | 1 | 3 | 192 |
| Marcelino Ramos | 0 | **113** | 0 | 0 | 4 | 117 |
| Mariana Pimentel | 0 | 15 | 0 | 0 | 1 | 16 |
| Marques De Souza | 0 | 12 | 0 | 0 | 0 | 12 |
| Mato Castelhano | 0 | 2 | 0 | 0 | 0 | 2 |
| Mato Leitão | 0 | 3 | 0 | 0 | 1 | 4 |
| Maximiliano de Almeida | 0 | 3 | 0 | 0 | 0 | 3 |
| Minas do Leão | 0 | 10 | 0 | 0 | 1 | 11 |
| Miraguaí | 0 | 22 | 1 | 0 | 0 | 23 |
| Montauri | 0 | 1 | 0 | 0 | 0 | 1 |
| Monte Alegre dos Campos | 0 | **75** | 0 | 0 | 6 | 81 |
| Monte Belo do Sul | 0 | 3 | 0 | 0 | 0 | 3 |
| Montenegro | 1 | **504** | 0 | 5 | 46 | 556 |
| Mormaço | 0 | 3 | 0 | 0 | 0 | 3 |
| Morrinhos do Sul | 0 | 4 | 0 | 0 | 0 | 4 |
| Morro Redondo | 0 | 15 | 0 | 0 | 0 | 15 |
| Morro Reuter | 0 | 23 | 0 | 1 | 0 | 24 |
| Mostardas | 0 | 17 | 0 | 0 | 1 | 18 |
| Muçum | 0 | 18 | 0 | 0 | 0 | 18 |
| Muitos Capotes | 0 | 8 | 0 | 0 | 0 | 8 |
| Não-Me-Toque | 0 | 25 | 0 | 0 | 0 | 25 |
| Nicolau Vergueiro | 0 | 6 | 0 | 0 | 0 | 6 |
| Nonoai | 0 | 3 | 0 | 0 | 0 | 3 |
| Nova Alvorada | 0 | 3 | 0 | 0 | 0 | 3 |
| Nova Bassano | 0 | 3 | 0 | 0 | 0 | 3 |
| Nova Boa Vista | 0 | 3 | 0 | 0 | 0 | 3 |
| Nova Brescia | 0 | 4 | 0 | 0 | 0 | 4 |
| Nova Candelária | 0 | 22 | 0 | 0 | 0 | 22 |
| Nova Esperança Do Sul | 0 | 4 | 0 | 0 | 0 | 4 |
| Nova Hartz | 3 | **1566** | 0 | 9 | **97** | 1675 |
| Nova Palma | 0 | 1 | 0 | 0 | 0 | 1 |
| Nova Petrópolis | 0 | 33 | 0 | 0 | 1 | 34 |
| Nova Prata | 0 | 34 | 0 | 0 | 1 | 35 |
| Nova Ramada | 0 | 3 | 0 | 0 | 1 | 4 |
| Nova Roma Do Sul | 0 | 2 | 0 | 0 | 0 | 2 |
| Nova Santa Rita | 0 | **176** | 0 | 3 | 12 | 191 |
| Novo Barreiro | 0 | 1 | 0 | 0 | 0 | 1 |
| Novo Cabrais | 0 | 1 | 0 | 0 | 0 | 1 |
| Town | Ethnicity (auto-perception based on skin color) | | | | | Total |
|  | ASID | EURD | NAAM | MIXD | AFRD |  |
| Novo Hamburgo | 1 | **4997** | 0 | 8 | **142** | 5148 |
| Novo Machado | 0 | 11 | 0 | 0 | 0 | 11 |
| Novo Tiradentes | 0 | 1 | 0 | 0 | 0 | 1 |
| Novo Xingu | 0 | 1 | 0 | 0 | 0 | 1 |
| Osório | 0 | **89** | 0 | 1 | 2 | 92 |
| Paim Filho | 0 | 5 | 0 | 0 | 0 | 5 |
| Palmares Do Sul | 0 | 13 | 0 | 0 | 2 | 15 |
| Palmeira Das Missões | 6 | **1005** | 0 | 21 | 11 | 1043 |
| Palmitinho | 0 | 5 | 0 | 0 | 0 | 5 |
| Panambi | 0 | **353** | 0 | 2 | 17 | 372 |
| Pântano Grande | 0 | 10 | 0 | 0 | 0 | 10 |
| Parai | 0 | 10 | 0 | 0 | 1 | 11 |
| Paraiso Do Sul | 0 | 3 | 0 | 0 | 0 | 3 |
| Pareci Novo | 0 | 19 | 0 | 1 | 0 | 20 |
| Parobé | 0 | **394** | 0 | 3 | 19 | 416 |
| Passa Sete | 0 | 1 | 0 | 0 | 0 | 1 |
| Passo Do Sobrado | 0 | 3 | 0 | 0 | 0 | 3 |
| Passo Fundo | 5 | **2113** | 0 | 42 | 40 | 2200 |
| Paverama | 0 | 11 | 0 | 1 | 0 | 12 |
| Pedro Osorio | 0 | 3 | 0 | 0 | 0 | 3 |
| Pejuçara | 0 | 33 | 0 | 0 | 0 | 33 |
| Pelotas | 6 | **4143** | 0 | **64** | **236** | 4449 |
| Picada Café | 0 | 11 | 0 | 1 | 0 | 12 |
| Pinhal | 0 | 5 | 0 | 0 | 1 | 6 |
| Pinhal Grande | 0 | 4 | 0 | 0 | 0 | 4 |
| Pinheirinho do Vale | 0 | 2 | 0 | 0 | 1 | 3 |
| Pinheiro Machado | 0 | 3 | 0 | 0 | 2 | 5 |
| Piratini | 0 | 5 | 0 | 0 | 0 | 5 |
| Planalto | 0 | 1 | 0 | 0 | 1 | 2 |
| Poço das Antas | 0 | 9 | 0 | 0 | 0 | 9 |
| Pontão | 0 | 2 | 0 | 0 | 0 | 2 |
| Ponte Preta | 0 | 3 | 0 | 0 | 0 | 3 |
| Portão | 1 | **206** | 0 | 3 | 6 | 216 |
| Porto Alegre | **104** | **21392** | 6 | **843** | **2057** | 24402 |
| Porto Lucena | 1 | **192** | 0 | 1 | 5 | 199 |
| Porto Mauá | 0 | 7 | 0 | 0 | 0 | 7 |
| Porto Vera Cruz | 0 | **57** | 0 | 0 | 3 | 60 |
| Porto Xavier | 0 | **186** | 0 | 0 | 9 | 195 |
| Pouso Novo | 0 | 2 | 0 | 0 | 0 | 2 |
| Presidente Lucena | 0 | 9 | 0 | 0 | 0 | 9 |
| Progresso | 0 | 4 | 0 | 0 | 0 | 4 |
| Protasio Alves | 0 | 2 | 0 | 0 | 0 | 2 |
| Town | Ethnicity (auto-perception based on skin color) | | | | | Total |
|  | ASID | EURD | NAAM | MIXD | AFRD |  |
| Putinga | 0 | 2 | 0 | 0 | 0 | 2 |
| Quarai | 0 | 13 | 0 | 1 | 2 | 16 |
| Quinze de Novembro | 0 | **52** | 0 | 0 | 1 | 53 |
| Redentora | 0 | **106** | 0 | 0 | 3 | 109 |
| Relvado | 0 | 1 | 0 | 0 | 0 | 1 |
| Restinga Seca | 0 | 4 | 0 | 0 | 0 | 4 |
| Rio Grande | 1 | **383** | 0 | 1 | 18 | 403 |
| Rio Pardo | 0 | **67** | 0 | 0 | 0 | 67 |
| Riozinho | 0 | **53** | 0 | 1 | 1 | 55 |
| Roca Sales | 0 | 16 | 0 | 0 | 1 | 17 |
| Rodeio Bonito | 0 | 1 | 0 | 0 | 0 | 1 |
| Rolador | 0 | 3 | 0 | 0 | 0 | 3 |
| Rolante | 0 | **103** | 0 | 0 | 4 | 107 |
| Ronda Alta | 0 | 10 | 0 | 0 | 0 | 10 |
| Rondinha | 0 | **99** | 0 | 5 | 4 | 108 |
| Roque Gonzales | 0 | 41 | 0 | 1 | 2 | 44 |
| Rosário do Sul | 0 | 15 | 0 | 1 | 1 | 17 |
| Saldanha Marinho | 0 | 14 | 0 | 0 | 0 | 14 |
| Salto do Jacuí | 0 | 47 | 0 | 0 | 6 | 53 |
| Salvador das Missões | 0 | 3 | 0 | 0 | 0 | 3 |
| Salvador do Sul | 0 | **66** | 0 | 0 | 3 | 69 |
| Sananduva | 0 | **110** | 0 | 0 | 1 | 111 |
| Santa Barbara do Sul | 0 | 25 | 0 | 0 | 1 | 26 |
| Santa Clara do Sul | 1 | **57** | 0 | 0 | 0 | 58 |
| Santa Cruz do Sul | 3 | **959** | 0 | 11 | 21 | 994 |
| Santa Maria | 9 | **1740** | 0 | **67** | **74** | 1890 |
| Santa Maria do Herval | 0 | 11 | 0 | 0 | 0 | 11 |
| Santa Rosa | 8 | **1916** | 0 | 2 | 24 | 1950 |
| Santa Vitoria do Palmar | 0 | 8 | 0 | 0 | 0 | 8 |
| Santana da Boa Vista | 0 | 4 | 0 | 0 | 0 | 4 |
| Santana do Livramento | 0 | **500** | 0 | 14 | 48 | 562 |
| Santiago | 0 | 20 | 0 | 0 | 2 | 22 |
| Santo Ângelo | 1 | **468** | 0 | 2 | 14 | 485 |
| Santo Antônio da Patrulha | 0 | **337** | 0 | 1 | 5 | 343 |
| Santo Antônio das Missões | 0 | 24 | 0 | 0 | 3 | 27 |
| Santo Antônio do Palma | 0 | 1 | 0 | 0 | 0 | 1 |
| Santo Antônio do Planalto | 0 | 5 | 0 | 0 | 0 | 5 |
| Santo Augusto | 0 | 18 | 0 | 1 | 3 | 22 |
| Santo Cristo | 0 | **144** | 0 | 1 | 0 | 145 |
| Santo Expedito do Sul | 0 | 1 | 0 | 0 | 0 | 1 |
| São Borja | 0 | 31 | 0 | 2 | 1 | 34 |
| São Domingos do Sul | 0 | 1 | 0 | 0 | 1 | 2 |
| Town | Ethnicity (auto-perception based on skin color) | | | | | Total |
|  | ASID | EURD | NAAM | MIXD | AFRD |  |
| São Francisco de Assis | 0 | 1 | 0 | 0 | 1 | 2 |
| São Francisco de Paula | 0 | 16 | 0 | 0 | 0 | 16 |
| São Gabriel | 0 | 46 | 0 | 0 | 4 | 50 |
| São Jeronimo | 0 | 24 | 0 | 1 | 3 | 28 |
| São Joao do Polesine | 0 | 1 | 0 | 0 | 0 | 1 |
| São Jose das Missões | 0 | 2 | 0 | 0 | 0 | 2 |
| São Jose do Herval | 0 | 1 | 0 | 0 | 0 | 1 |
| São Jose do Hortencio | 0 | 13 | 0 | 0 | 0 | 13 |
| São Jose do Inhacorá | 0 | **147** | 0 | 0 | 2 | 149 |
| São Jose do Norte | 1 | **159** | 0 | 0 | 6 | 166 |
| São Jose do Ouro | 0 | 4 | 0 | 0 | 0 | 4 |
| São Jose do Sul | 0 | 8 | 0 | 0 | 0 | 8 |
| São Jose dos Ausentes | 0 | 3 | 0 | 0 | 0 | 3 |
| São Leopoldo | 1 | **1780** | 1 | 8 | **74** | 1864 |
| São Lourenco do Sul | 0 | 31 | 0 | 0 | 4 | 35 |
| São Luiz Gonzaga | 0 | **291** | 0 | 2 | 17 | 310 |
| São Marcos | 0 | 27 | 0 | 0 | 1 | 28 |
| São Martinho | 0 | 6 | 0 | 0 | 0 | 6 |
| São Martinho da Serra | 0 | 3 | 0 | 1 | 0 | 4 |
| São Miguel das Missões | 0 | 7 | 0 | 0 | 0 | 7 |
| São Nicolau | 0 | 2 | 0 | 0 | 1 | 3 |
| São Paulo das Missões | 0 | **144** | 0 | 0 | 4 | 148 |
| São Pedro da Serra | 0 | 36 | 0 | 0 | 0 | 36 |
| São Pedro do Sul | 0 | 5 | 0 | 0 | 0 | 5 |
| São Sebastiao do Cai | 0 | **79** | 0 | 0 | 2 | 81 |
| São Sepé | 0 | 1 | 0 | 0 | 0 | 1 |
| São Valentim | 0 | 2 | 0 | 0 | 0 | 2 |
| São Valentim do Sul | 0 | 2 | 0 | 0 | 0 | 2 |
| São Valério do Sul | 0 | **50** | 0 | 0 | 0 | 50 |
| São Vendelino | 0 | 2 | 0 | 0 | 0 | 2 |
| São Vicente do Sul | 0 | 3 | 0 | 0 | 0 | 3 |
| Sapiranga | 1 | **1289** | 1 | 28 | 37 | 1356 |
| Sapucaia do Sul | 2 | **955** | 0 | 19 | **54** | 1030 |
| Sarandi | 0 | **87** | 0 | 2 | 7 | 96 |
| Seberi | 0 | 7 | 0 | 0 | 0 | 7 |
| Sede Nova | 0 | **135** | 0 | 0 | 11 | 146 |
| Segredo | 0 | 1 | 0 | 0 | 0 | 1 |
| Selbach | 0 | 8 | 0 | 0 | 0 | 8 |
| Senador Salgado Filho | 0 | 6 | 0 | 0 | 0 | 6 |
| Sentinela Do Sul | 0 | 10 | 0 | 0 | 2 | 12 |
| Serafina Correa | 0 | **69** | 0 | 2 | 1 | 72 |
| Serio | 0 | 1 | 0 | 0 | 0 | 1 |
| Town | Ethnicity (auto-perception based on skin color) | | | | | Total |
|  | ASID | EURD | NAAM | MIXD | AFRD |  |
| Sertão | 0 | 12 | 0 | 0 | 0 | 12 |
| Sertão Santana | 1 | 23 | 0 | 0 | 0 | 24 |
| Severiano de Almeida | 0 | 1 | 0 | 0 | 0 | 1 |
| Sinimbu | 0 | 4 | 0 | 0 | 0 | 4 |
| Sobradinho | 0 | 13 | 0 | 1 | 0 | 14 |
| Soledade | 0 | 21 | 0 | 0 | 0 | 21 |
| Tabaí | 0 | 7 | 0 | 0 | 0 | 7 |
| Tapejara | 0 | **50** | 0 | 0 | 1 | 51 |
| Tapera | 0 | 24 | 0 | 0 | 0 | 24 |
| Tapes | 0 | 28 | 0 | 0 | 6 | 34 |
| Taquara | 0 | **375** | 0 | 2 | 16 | 393 |
| Taquari | 0 | **101** | 0 | 0 | 6 | 107 |
| Taquaruçu do Sul | 0 | 2 | 0 | 0 | 0 | 2 |
| Tavares | 0 | **66** | 0 | 0 | 4 | 70 |
| Tenente Portela | 0 | **356** | 0 | 0 | 12 | 368 |
| Terra De Areia | 0 | 13 | 0 | 0 | 0 | 13 |
| Teutônia | 0 | 48 | 0 | 1 | 1 | 50 |
| Tiradentes Do Sul | 0 | 2 | 0 | 0 | 0 | 2 |
| Torres | 0 | **85** | 0 | 0 | 2 | 87 |
| Tramandaí | 0 | **67** | 0 | 0 | 3 | 70 |
| Travesseiro | 0 | 2 | 0 | 0 | 0 | 2 |
| Três Arroios | 0 | 1 | 0 | 0 | 0 | 1 |
| Três Cachoeiras | 0 | 37 | 0 | 0 | 0 | 37 |
| Três Coroas | 0 | **56** | 0 | 0 | 2 | 58 |
| Três de Maio | 0 | **197** | 0 | 0 | 7 | 204 |
| Três Forquilhas | 0 | 4 | 0 | 0 | 0 | 4 |
| Três Palmeiras | 0 | 6 | 0 | 0 | 0 | 6 |
| Três Passos | 0 | **326** | 0 | 0 | 6 | 332 |
| Trindade Do Sul | 0 | 3 | 0 | 0 | 1 | 4 |
| Triunfo | 0 | **85** | 0 | 0 | 2 | 87 |
| Tucunduva | 0 | 12 | 0 | 0 | 0 | 12 |
| Tunas | 0 | 2 | 0 | 0 | 0 | 2 |
| Tupanci do Sul | 0 | 4 | 0 | 0 | 0 | 4 |
| Tupanciretã | 0 | **50** | 0 | 1 | 3 | 54 |
| Tupandi | 0 | 8 | 0 | 0 | 0 | 8 |
| Tuparendi | 0 | **243** | 0 | 0 | 4 | 247 |
| Turucu | 0 | 0 | 0 | 0 | 1 | 1 |
| Ubiretama | 0 | 3 | 0 | 0 | 0 | 3 |
| Uruguaiana | 0 | **1346** | 1 | **50** | **140** | 1537 |
| Vacaria | 0 | **614** | 0 | 12 | **53** | 679 |
| Vale Do Sol | 0 | 8 | 0 | 0 | 0 | 8 |
| Vale Real | 0 | 49 | 0 | 0 | 0 | 49 |
| Town | Ethnicity (auto-perception based on skin color) | | | | | Total |
|  | ASID | EURD | NAAM | MIXD | AFRD |  |
| Vale Verde | 0 | 1 | 0 | 0 | 0 | 1 |
| Vanini | 0 | 2 | 0 | 0 | 0 | 2 |
| Venâncio Aires | 0 | **71** | 0 | 0 | 2 | 73 |
| Vera Cruz | 0 | **57** | 0 | 0 | 1 | 58 |
| Veranópolis | 0 | 17 | 0 | 0 | 0 | 17 |
| Vespasiano Correa | 0 | 2 | 0 | 0 | 0 | 2 |
| Viadutos | 0 | 3 | 0 | 0 | 0 | 3 |
| Viamão | 0 | **1327** | 2 | 36 | **158** | 1523 |
| Vicente Dutra | 0 | 3 | 0 | 0 | 0 | 3 |
| Victor Graeff | 0 | 2 | 0 | 0 | 0 | 2 |
| Vila Flores | 0 | 1 | 0 | 0 | 1 | 2 |
| Vila Lângaro | 0 | 2 | 0 | 0 | 0 | 2 |
| Vila Maria | 0 | 6 | 0 | 0 | 0 | 6 |
| Vila Nova Do Sul | 0 | 1 | 0 | 0 | 0 | 1 |
| Vista Alegre | 0 | 1 | 0 | 0 | 1 | 2 |
| Vista Alegre Do Prata | 0 | 2 | 0 | 0 | 0 | 2 |
| Vista Gaúcha | 0 | 14 | 0 | 0 | 1 | 15 |
| Vitoria Das Missões | 0 | 5 | 0 | 0 | 4 | 9 |
| Westfália | 0 | 11 | 0 | 0 | 1 | 12 |
| Xangri-Lá | 0 | 42 | 0 | 0 | 2 | 44 |
| Total | 262 | 90105 | 19 | 1632 | 5274 | 97292 |

AFRD: African descent; ASID: Asian descent; EURD: European descent; MIXD: Mixed descent; NAAM: Native American. Populations in bold were included in the analyses.
