# Supplementary material for: Spatial analyzes of HLA data in Rio Grande do Sul, south Brazil: genetic structure and possible correlation with autoimmune diseases
Source: Int J Health Geogr. 2018 Sep 14;17:34. doi: 10.1186/s12942-018-0154-8 (PMC6137739; doi:10.1186/s12942-018-0154-8)
Supplement: Supplementary file 2 — Additional file 2. Allelic frequencies, genetic diversity and Hardy–Weinberg equilibrium. [file 12942_2018_154_MOESM2_ESM.docx]

**Additional file 2 – Allelic frequencies, genetic diversity and Hardy-Weinberg equilibrium**

HLA-A, -B and -DRB1 allelic frequencies (low resolution) for Rio Grande do Sul cities. Only cities with EURD self-declared population and with sample size greater or equal to 50 were included in the study. All cities tested are in Hardy-Weinberg equilibrium. Genetic diversity is given by the expected heterozygosity (Exp H).

**HLA-A**

| **Town** | **N** | **A*01** | **A*02** | **A*03** | **A*11** | **A*23** | **A*24** | **A*25** | **A*26** | **A*29** | **A*30** | **A*31** | **A*32** | **A*33** | **A*34** | **A*36** | **A*43** | **A*66** | **A*68** | **A*69** | **A*74** | **A*80** | **blank** | **Exp H** | **P-HWE** |
| --- | --- | --- | --- | --- | --- | --- | --- | --- | --- | --- | --- | --- | --- | --- | --- | --- | --- | --- | --- | --- | --- | --- | --- | --- | --- |
| Agudo | 99 | 0.086 | 0.288 | 0.111 | 0.091 | 0.015 | 0.081 | 0.015 | 0.045 | 0.025 | 0.020 | 0.061 | 0.025 | 0.025 | 0.000 | 0.020 | 0.000 | 0.000 | 0.081 | 0.010 | 0.000 | 0.000 | 0.000 | 0.867 | 1 |
| Alegrete | 1259 | 0.083 | 0.273 | 0.109 | 0.042 | 0.032 | 0.101 | 0.014 | 0.033 | 0.052 | 0.039 | 0.077 | 0.029 | 0.027 | 0.005 | 0.003 | 0.000 | 0.006 | 0.062 | 0.001 | 0.007 | 0.002 | 0.002 | 0.877 | 0.935 |
| Alvorada | 841 | 0.092 | 0.275 | 0.109 | 0.054 | 0.034 | 0.104 | 0.017 | 0.037 | 0.052 | 0.039 | 0.065 | 0.033 | 0.021 | 0.004 | 0.005 | 0.000 | 0.005 | 0.045 | 0.003 | 0.004 | 0.001 | 0.000 | 0.875 | 1 |
| Ametista Do Sul | 62 | 0.129 | 0.202 | 0.169 | 0.056 | 0.040 | 0.073 | 0.016 | 0.040 | 0.056 | 0.056 | 0.024 | 0.048 | 0.032 | 0.008 | 0.000 | 0.000 | 0.000 | 0.040 | 0.008 | 0.000 | 0.000 | 0.000 | 0.890 | 1 |
| Arambare | 60 | 0.100 | 0.300 | 0.058 | 0.033 | 0.083 | 0.133 | 0.017 | 0.025 | 0.058 | 0.008 | 0.058 | 0.017 | 0.050 | 0.000 | 0.000 | 0.000 | 0.000 | 0.050 | 0.000 | 0.008 | 0.000 | 0.000 | 0.858 | 1 |
| Aratiba | 130 | 0.119 | 0.285 | 0.077 | 0.042 | 0.019 | 0.150 | 0.031 | 0.042 | 0.038 | 0.038 | 0.035 | 0.042 | 0.027 | 0.000 | 0.000 | 0.000 | 0.008 | 0.042 | 0.000 | 0.000 | 0.004 | 0.000 | 0.863 | 1 |
| Arroio Do Meio | 86 | 0.150 | 0.243 | 0.132 | 0.047 | 0.006 | 0.115 | 0.017 | 0.097 | 0.017 | 0.023 | 0.017 | 0.052 | 0.012 | 0.000 | 0.000 | 0.000 | 0.000 | 0.058 | 0.000 | 0.006 | 0.000 | 0.007 | 0.868 | 0.934 |
| Arroio Do Sal | 507 | 0.102 | 0.300 | 0.108 | 0.059 | 0.028 | 0.106 | 0.013 | 0.031 | 0.064 | 0.031 | 0.047 | 0.031 | 0.025 | 0.001 | 0.001 | 0.000 | 0.003 | 0.050 | 0.001 | 0.001 | 0.000 | 0.000 | 0.860 | 1 |
| Augusto Pestana | 65 | 0.161 | 0.346 | 0.138 | 0.069 | 0.008 | 0.053 | 0.015 | 0.023 | 0.023 | 0.046 | 0.046 | 0.015 | 0.008 | 0.015 | 0.000 | 0.000 | 0.000 | 0.031 | 0.000 | 0.000 | 0.000 | 0.001 | 0.820 | 1 |
| Bento Goncalves | 248 | 0.143 | 0.252 | 0.106 | 0.040 | 0.020 | 0.121 | 0.022 | 0.056 | 0.026 | 0.028 | 0.054 | 0.050 | 0.030 | 0.000 | 0.002 | 0.000 | 0.002 | 0.042 | 0.000 | 0.002 | 0.000 | 0.003 | 0.875 | 0.987 |
| Boa Vista Do Burica | 173 | 0.147 | 0.260 | 0.159 | 0.064 | 0.023 | 0.090 | 0.029 | 0.029 | 0.052 | 0.026 | 0.029 | 0.020 | 0.014 | 0.000 | 0.009 | 0.000 | 0.003 | 0.040 | 0.000 | 0.006 | 0.000 | 0.000 | 0.864 | 1 |
| Cachoeira Do Sul | 58 | 0.103 | 0.250 | 0.121 | 0.069 | 0.026 | 0.095 | 0.017 | 0.000 | 0.060 | 0.017 | 0.086 | 0.069 | 0.009 | 0.000 | 0.000 | 0.000 | 0.009 | 0.060 | 0.000 | 0.000 | 0.009 | 0.000 | 0.878 | 1 |
| Cachoeirinha | 1139 | 0.087 | 0.275 | 0.116 | 0.051 | 0.034 | 0.109 | 0.015 | 0.030 | 0.056 | 0.037 | 0.051 | 0.035 | 0.019 | 0.003 | 0.004 | 0.000 | 0.010 | 0.050 | 0.002 | 0.006 | 0.000 | 0.008 | 0.875 | 0.696 |
| Caibate | 88 | 0.125 | 0.273 | 0.165 | 0.051 | 0.011 | 0.091 | 0.023 | 0.023 | 0.023 | 0.023 | 0.057 | 0.045 | 0.028 | 0.000 | 0.000 | 0.000 | 0.006 | 0.057 | 0.000 | 0.000 | 0.000 | 0.000 | 0.860 | 1 |
| Camaqua | 114 | 0.151 | 0.285 | 0.108 | 0.060 | 0.033 | 0.086 | 0.009 | 0.031 | 0.037 | 0.022 | 0.044 | 0.022 | 0.028 | 0.009 | 0.000 | 0.000 | 0.009 | 0.046 | 0.000 | 0.000 | 0.000 | 0.022 | 0.864 | 0.733 |
| Campestre Da Serra | 92 | 0.119 | 0.260 | 0.149 | 0.054 | 0.016 | 0.102 | 0.022 | 0.038 | 0.030 | 0.030 | 0.053 | 0.076 | 0.005 | 0.000 | 0.000 | 0.000 | 0.005 | 0.016 | 0.005 | 0.005 | 0.000 | 0.013 | 0.870 | 0.854 |
| Campina Das Missoes | 78 | 0.101 | 0.261 | 0.154 | 0.051 | 0.054 | 0.061 | 0.013 | 0.051 | 0.026 | 0.013 | 0.019 | 0.032 | 0.045 | 0.000 | 0.000 | 0.000 | 0.000 | 0.084 | 0.000 | 0.000 | 0.000 | 0.035 | 0.873 | 0.649 |
| Campo Bom | 612 | 0.105 | 0.291 | 0.109 | 0.048 | 0.040 | 0.105 | 0.012 | 0.037 | 0.047 | 0.032 | 0.053 | 0.039 | 0.021 | 0.002 | 0.002 | 0.000 | 0.007 | 0.045 | 0.002 | 0.002 | 0.000 | 0.000 | 0.866 | 1 |
| Campo Novo | 177 | 0.121 | 0.277 | 0.096 | 0.065 | 0.014 | 0.124 | 0.025 | 0.048 | 0.039 | 0.020 | 0.056 | 0.031 | 0.020 | 0.003 | 0.003 | 0.000 | 0.006 | 0.045 | 0.006 | 0.000 | 0.000 | 0.001 | 0.868 | 1 |
| Candido Godoi | 172 | 0.145 | 0.290 | 0.110 | 0.070 | 0.041 | 0.102 | 0.012 | 0.049 | 0.012 | 0.026 | 0.032 | 0.029 | 0.020 | 0.000 | 0.000 | 0.000 | 0.006 | 0.046 | 0.000 | 0.009 | 0.000 | 0.001 | 0.858 | 1 |
| Canela | 51 | 0.151 | 0.293 | 0.063 | 0.033 | 0.020 | 0.098 | 0.020 | 0.049 | 0.059 | 0.029 | 0.049 | 0.053 | 0.020 | 0.000 | 0.000 | 0.000 | 0.010 | 0.000 | 0.010 | 0.000 | 0.010 | 0.035 | 0.862 | 0.712 |
| Canoas | 3820 | 0.102 | 0.279 | 0.105 | 0.052 | 0.038 | 0.103 | 0.018 | 0.028 | 0.050 | 0.037 | 0.050 | 0.033 | 0.024 | 0.005 | 0.003 | 0.000 | 0.007 | 0.056 | 0.002 | 0.007 | 0.001 | 0.000 | 0.874 | 1 |
| Capao Da Canoa | 104 | 0.096 | 0.306 | 0.148 | 0.029 | 0.019 | 0.097 | 0.014 | 0.005 | 0.062 | 0.029 | 0.050 | 0.038 | 0.005 | 0.005 | 0.010 | 0.000 | 0.000 | 0.053 | 0.000 | 0.000 | 0.000 | 0.033 | 0.852 | 0.618 |
| Carazinho | 314 | 0.108 | 0.296 | 0.100 | 0.061 | 0.037 | 0.113 | 0.010 | 0.035 | 0.045 | 0.040 | 0.054 | 0.025 | 0.019 | 0.003 | 0.003 | 0.000 | 0.003 | 0.040 | 0.003 | 0.005 | 0.000 | 0.000 | 0.862 | 1 |
| Catuipe | 52 | 0.135 | 0.298 | 0.125 | 0.077 | 0.019 | 0.096 | 0.010 | 0.029 | 0.029 | 0.058 | 0.038 | 0.029 | 0.010 | 0.000 | 0.000 | 0.000 | 0.000 | 0.038 | 0.000 | 0.010 | 0.000 | 0.000 | 0.853 | 1 |
| Caxias Do Sul | 4959 | 0.114 | 0.273 | 0.116 | 0.053 | 0.035 | 0.112 | 0.016 | 0.035 | 0.041 | 0.031 | 0.048 | 0.040 | 0.023 | 0.003 | 0.002 | 0.000 | 0.007 | 0.044 | 0.002 | 0.004 | 0.001 | 0.001 | 0.872 | 0.949 |
| Cerro Largo | 59 | 0.136 | 0.290 | 0.134 | 0.042 | 0.051 | 0.093 | 0.025 | 0.025 | 0.042 | 0.017 | 0.038 | 0.017 | 0.025 | 0.000 | 0.000 | 0.000 | 0.000 | 0.034 | 0.000 | 0.008 | 0.000 | 0.021 | 0.859 | 0.816 |
| Charqueadas | 107 | 0.056 | 0.286 | 0.093 | 0.082 | 0.026 | 0.083 | 0.009 | 0.042 | 0.093 | 0.056 | 0.061 | 0.033 | 0.005 | 0.000 | 0.005 | 0.000 | 0.000 | 0.055 | 0.000 | 0.005 | 0.000 | 0.011 | 0.870 | 0.873 |
| Crissiumal | 277 | 0.132 | 0.293 | 0.137 | 0.066 | 0.032 | 0.059 | 0.020 | 0.027 | 0.042 | 0.031 | 0.027 | 0.049 | 0.016 | 0.000 | 0.000 | 0.000 | 0.000 | 0.066 | 0.000 | 0.000 | 0.000 | 0.004 | 0.858 | 0.955 |
| Cruz Alta | 1977 | 0.096 | 0.273 | 0.116 | 0.049 | 0.037 | 0.109 | 0.019 | 0.034 | 0.043 | 0.039 | 0.054 | 0.037 | 0.028 | 0.005 | 0.002 | 0.000 | 0.005 | 0.050 | 0.002 | 0.004 | 0.000 | 0.001 | 0.875 | 0.980 |
| **Town** | **N** | **A*01** | **A*02** | **A*03** | **A*11** | **A*23** | **A*24** | **A*25** | **A*26** | **A*29** | **A*30** | **A*31** | **A*32** | **A*33** | **A*34** | **A*36** | **A*43** | **A*66** | **A*68** | **A*69** | **A*74** | **A*80** | **blank** | **Exp H** | **P-HWE** |
| Cruzeiro Do Sul | 79 | 0.127 | 0.285 | 0.171 | 0.063 | 0.038 | 0.089 | 0.013 | 0.019 | 0.006 | 0.019 | 0.019 | 0.057 | 0.038 | 0.000 | 0.000 | 0.000 | 0.006 | 0.032 | 0.000 | 0.019 | 0.000 | 0.000 | 0.853 | 1 |
| Dois Irmaos | 163 | 0.122 | 0.297 | 0.141 | 0.049 | 0.028 | 0.113 | 0.006 | 0.027 | 0.037 | 0.031 | 0.040 | 0.025 | 0.015 | 0.000 | 0.000 | 0.000 | 0.009 | 0.052 | 0.003 | 0.003 | 0.000 | 0.001 | 0.853 | 1 |
| Doutor Mauricio Cardoso | 172 | 0.105 | 0.256 | 0.108 | 0.081 | 0.029 | 0.119 | 0.049 | 0.020 | 0.058 | 0.012 | 0.041 | 0.035 | 0.015 | 0.000 | 0.000 | 0.000 | 0.009 | 0.058 | 0.000 | 0.006 | 0.000 | 0.000 | 0.877 | 1 |
| Eldorado Do Sul | 175 | 0.114 | 0.291 | 0.117 | 0.043 | 0.049 | 0.091 | 0.017 | 0.034 | 0.031 | 0.034 | 0.060 | 0.026 | 0.031 | 0.003 | 0.006 | 0.003 | 0.000 | 0.043 | 0.000 | 0.006 | 0.000 | 0.000 | 0.865 | 1 |
| Encantado | 214 | 0.152 | 0.259 | 0.126 | 0.042 | 0.028 | 0.105 | 0.021 | 0.037 | 0.040 | 0.019 | 0.051 | 0.051 | 0.014 | 0.000 | 0.000 | 0.000 | 0.000 | 0.049 | 0.005 | 0.000 | 0.000 | 0.000 | 0.868 | 1 |
| Erechim | 663 | 0.116 | 0.280 | 0.112 | 0.068 | 0.026 | 0.115 | 0.022 | 0.051 | 0.043 | 0.027 | 0.039 | 0.032 | 0.020 | 0.001 | 0.003 | 0.000 | 0.005 | 0.036 | 0.002 | 0.002 | 0.000 | 0.001 | 0.867 | 1 |
| Espumoso | 107 | 0.124 | 0.311 | 0.084 | 0.051 | 0.051 | 0.096 | 0.033 | 0.047 | 0.021 | 0.014 | 0.063 | 0.037 | 0.009 | 0.000 | 0.000 | 0.000 | 0.005 | 0.042 | 0.000 | 0.000 | 0.000 | 0.010 | 0.855 | 0.884 |
| Estancia Velha | 883 | 0.123 | 0.270 | 0.130 | 0.054 | 0.033 | 0.097 | 0.013 | 0.032 | 0.049 | 0.026 | 0.033 | 0.045 | 0.028 | 0.001 | 0.001 | 0.000 | 0.006 | 0.055 | 0.001 | 0.001 | 0.000 | 0.000 | 0.870 | 1 |
| Esteio | 654 | 0.100 | 0.299 | 0.105 | 0.062 | 0.043 | 0.090 | 0.015 | 0.026 | 0.047 | 0.029 | 0.061 | 0.032 | 0.020 | 0.002 | 0.000 | 0.000 | 0.008 | 0.052 | 0.002 | 0.006 | 0.000 | 0.002 | 0.864 | 0.964 |
| Estrela | 799 | 0.125 | 0.280 | 0.157 | 0.046 | 0.032 | 0.086 | 0.020 | 0.037 | 0.028 | 0.019 | 0.037 | 0.042 | 0.025 | 0.003 | 0.001 | 0.000 | 0.006 | 0.048 | 0.001 | 0.003 | 0.000 | 0.003 | 0.862 | 0.938 |
| Farroupilha | 2347 | 0.115 | 0.271 | 0.121 | 0.055 | 0.031 | 0.112 | 0.021 | 0.035 | 0.038 | 0.034 | 0.041 | 0.041 | 0.024 | 0.001 | 0.003 | 0.000 | 0.003 | 0.049 | 0.001 | 0.001 | 0.000 | 0.000 | 0.871 | 1 |
| Flores Da Cunha | 56 | 0.152 | 0.313 | 0.089 | 0.063 | 0.018 | 0.116 | 0.036 | 0.063 | 0.018 | 0.018 | 0.018 | 0.036 | 0.009 | 0.000 | 0.000 | 0.000 | 0.009 | 0.045 | 0.000 | 0.000 | 0.000 | 0.000 | 0.844 | 1 |
| Garibaldi | 65 | 0.169 | 0.211 | 0.131 | 0.092 | 0.046 | 0.042 | 0.015 | 0.090 | 0.038 | 0.008 | 0.066 | 0.026 | 0.000 | 0.000 | 0.000 | 0.000 | 0.008 | 0.034 | 0.000 | 0.000 | 0.000 | 0.024 | 0.881 | 0.783 |
| Getulio Vargas | 90 | 0.083 | 0.261 | 0.111 | 0.100 | 0.011 | 0.111 | 0.011 | 0.022 | 0.078 | 0.061 | 0.028 | 0.028 | 0.017 | 0.000 | 0.000 | 0.000 | 0.006 | 0.072 | 0.000 | 0.000 | 0.000 | 0.000 | 0.873 | 1 |
| Gravatai | 3217 | 0.101 | 0.278 | 0.111 | 0.054 | 0.036 | 0.102 | 0.015 | 0.030 | 0.055 | 0.034 | 0.047 | 0.038 | 0.024 | 0.002 | 0.003 | 0.000 | 0.007 | 0.055 | 0.002 | 0.006 | 0.000 | 0.000 | 0.873 | 1 |
| Guaiba | 469 | 0.095 | 0.301 | 0.098 | 0.042 | 0.044 | 0.098 | 0.013 | 0.031 | 0.037 | 0.033 | 0.062 | 0.037 | 0.035 | 0.010 | 0.004 | 0.000 | 0.007 | 0.045 | 0.000 | 0.007 | 0.001 | 0.000 | 0.865 | 1 |
| Humaita | 58 | 0.135 | 0.351 | 0.154 | 0.095 | 0.034 | 0.086 | 0.009 | 0.017 | 0.026 | 0.009 | 0.040 | 0.000 | 0.000 | 0.009 | 0.000 | 0.000 | 0.009 | 0.009 | 0.009 | 0.000 | 0.000 | 0.010 | 0.814 | 0.921 |
| Ibiruba | 93 | 0.096 | 0.280 | 0.086 | 0.051 | 0.048 | 0.153 | 0.005 | 0.016 | 0.043 | 0.027 | 0.048 | 0.027 | 0.023 | 0.000 | 0.000 | 0.000 | 0.005 | 0.051 | 0.005 | 0.005 | 0.000 | 0.029 | 0.867 | 0.680 |
| Igrejinha | 279 | 0.125 | 0.319 | 0.149 | 0.045 | 0.032 | 0.084 | 0.014 | 0.029 | 0.038 | 0.020 | 0.047 | 0.041 | 0.014 | 0.002 | 0.000 | 0.000 | 0.000 | 0.038 | 0.002 | 0.002 | 0.000 | 0.000 | 0.842 | 1 |
| Ijui | 561 | 0.120 | 0.270 | 0.120 | 0.064 | 0.032 | 0.096 | 0.015 | 0.035 | 0.042 | 0.038 | 0.046 | 0.035 | 0.027 | 0.001 | 0.002 | 0.000 | 0.003 | 0.048 | 0.000 | 0.004 | 0.002 | 0.000 | 0.873 | 1 |
| Itaqui | 142 | 0.116 | 0.236 | 0.106 | 0.035 | 0.018 | 0.130 | 0.011 | 0.039 | 0.042 | 0.028 | 0.123 | 0.042 | 0.011 | 0.011 | 0.000 | 0.000 | 0.014 | 0.039 | 0.000 | 0.000 | 0.000 | 0.000 | 0.878 | 1 |
| Ivoti | 325 | 0.126 | 0.242 | 0.145 | 0.058 | 0.035 | 0.098 | 0.020 | 0.031 | 0.040 | 0.022 | 0.057 | 0.046 | 0.014 | 0.002 | 0.003 | 0.000 | 0.006 | 0.052 | 0.002 | 0.002 | 0.000 | 0.000 | 0.879 | 1 |
| Jacutinga | 154 | 0.136 | 0.258 | 0.115 | 0.070 | 0.019 | 0.102 | 0.010 | 0.051 | 0.036 | 0.021 | 0.042 | 0.045 | 0.045 | 0.000 | 0.000 | 0.000 | 0.000 | 0.036 | 0.003 | 0.000 | 0.000 | 0.010 | 0.874 | 0.859 |
| Lagoa Vermelha | 77 | 0.104 | 0.234 | 0.091 | 0.039 | 0.032 | 0.156 | 0.000 | 0.045 | 0.019 | 0.026 | 0.019 | 0.052 | 0.039 | 0.000 | 0.006 | 0.000 | 0.019 | 0.091 | 0.006 | 0.013 | 0.006 | 0.000 | 0.883 | 1 |
| Lajeado | 1484 | 0.136 | 0.275 | 0.146 | 0.057 | 0.032 | 0.091 | 0.015 | 0.033 | 0.034 | 0.026 | 0.033 | 0.041 | 0.019 | 0.000 | 0.001 | 0.000 | 0.005 | 0.047 | 0.002 | 0.002 | 0.001 | 0.002 | 0.863 | 0.938 |
| Marau | 188 | 0.125 | 0.250 | 0.120 | 0.040 | 0.035 | 0.106 | 0.016 | 0.037 | 0.040 | 0.040 | 0.061 | 0.040 | 0.035 | 0.000 | 0.000 | 0.000 | 0.000 | 0.048 | 0.003 | 0.005 | 0.000 | 0.000 | 0.880 | 1 |
| Marcelino Ramos | 113 | 0.122 | 0.248 | 0.096 | 0.047 | 0.013 | 0.133 | 0.013 | 0.029 | 0.033 | 0.035 | 0.058 | 0.071 | 0.022 | 0.000 | 0.000 | 0.000 | 0.004 | 0.062 | 0.000 | 0.000 | 0.000 | 0.013 | 0.878 | 0.839 |
| Monte Alegre Dos Campos | 75 | 0.191 | 0.169 | 0.133 | 0.036 | 0.029 | 0.105 | 0.013 | 0.022 | 0.027 | 0.073 | 0.105 | 0.020 | 0.000 | 0.000 | 0.000 | 0.000 | 0.007 | 0.047 | 0.000 | 0.000 | 0.000 | 0.024 | 0.883 | 0.767 |
| Montenegro | 504 | 0.108 | 0.283 | 0.108 | 0.073 | 0.046 | 0.085 | 0.019 | 0.033 | 0.041 | 0.028 | 0.049 | 0.042 | 0.022 | 0.003 | 0.001 | 0.000 | 0.009 | 0.043 | 0.001 | 0.007 | 0.001 | 0.000 | 0.872 | 1 |
| Nova Hartz | 1566 | 0.110 | 0.289 | 0.118 | 0.051 | 0.044 | 0.099 | 0.014 | 0.036 | 0.041 | 0.027 | 0.045 | 0.032 | 0.018 | 0.004 | 0.003 | 0.000 | 0.008 | 0.054 | 0.001 | 0.003 | 0.001 | 0.001 | 0.866 | 0.969 |
| Nova Santa Rita | 176 | 0.091 | 0.258 | 0.096 | 0.068 | 0.048 | 0.116 | 0.026 | 0.026 | 0.037 | 0.042 | 0.043 | 0.051 | 0.034 | 0.006 | 0.003 | 0.000 | 0.003 | 0.051 | 0.000 | 0.000 | 0.000 | 0.002 | 0.883 | 0.967 |
| Novo Hamburgo | 4997 | 0.115 | 0.284 | 0.118 | 0.050 | 0.039 | 0.103 | 0.015 | 0.031 | 0.041 | 0.032 | 0.050 | 0.034 | 0.022 | 0.003 | 0.001 | 0.000 | 0.007 | 0.045 | 0.001 | 0.005 | 0.001 | 0.003 | 0.867 | 0.737 |
| Osorio | 89 | 0.134 | 0.289 | 0.070 | 0.062 | 0.022 | 0.090 | 0.017 | 0.017 | 0.045 | 0.067 | 0.060 | 0.028 | 0.045 | 0.000 | 0.000 | 0.000 | 0.006 | 0.031 | 0.000 | 0.000 | 0.006 | 0.010 | 0.867 | 0.893 |
| **Town** | **N** | **A*01** | **A*02** | **A*03** | **A*11** | **A*23** | **A*24** | **A*25** | **A*26** | **A*29** | **A*30** | **A*31** | **A*32** | **A*33** | **A*34** | **A*36** | **A*43** | **A*66** | **A*68** | **A*69** | **A*74** | **A*80** | **blank** | **Exp H** | **P-HWE** |
| Palmeira Das Missoes | 1005 | 0.124 | 0.262 | 0.110 | 0.056 | 0.028 | 0.121 | 0.016 | 0.050 | 0.039 | 0.031 | 0.043 | 0.039 | 0.017 | 0.001 | 0.002 | 0.000 | 0.005 | 0.051 | 0.000 | 0.002 | 0.000 | 0.003 | 0.874 | 0.927 |
| Panambi | 353 | 0.116 | 0.272 | 0.140 | 0.054 | 0.044 | 0.099 | 0.017 | 0.030 | 0.031 | 0.024 | 0.055 | 0.037 | 0.020 | 0.000 | 0.003 | 0.000 | 0.001 | 0.052 | 0.001 | 0.003 | 0.000 | 0.000 | 0.868 | 1 |
| Parobe | 394 | 0.098 | 0.284 | 0.109 | 0.052 | 0.053 | 0.100 | 0.011 | 0.027 | 0.044 | 0.022 | 0.058 | 0.035 | 0.019 | 0.003 | 0.006 | 0.000 | 0.003 | 0.055 | 0.006 | 0.001 | 0.000 | 0.015 | 0.871 | 0.673 |
| Passo Fundo | 2113 | 0.110 | 0.262 | 0.109 | 0.050 | 0.035 | 0.120 | 0.020 | 0.033 | 0.045 | 0.031 | 0.061 | 0.037 | 0.020 | 0.003 | 0.001 | 0.000 | 0.005 | 0.052 | 0.001 | 0.003 | 0.001 | 0.001 | 0.876 | 0.964 |
| Pelotas | 4143 | 0.108 | 0.273 | 0.104 | 0.054 | 0.041 | 0.095 | 0.018 | 0.036 | 0.048 | 0.036 | 0.044 | 0.037 | 0.025 | 0.004 | 0.002 | 0.000 | 0.008 | 0.056 | 0.002 | 0.005 | 0.003 | 0.002 | 0.877 | 0.892 |
| Portao | 206 | 0.104 | 0.289 | 0.095 | 0.039 | 0.053 | 0.095 | 0.017 | 0.044 | 0.039 | 0.032 | 0.061 | 0.039 | 0.036 | 0.002 | 0.000 | 0.000 | 0.007 | 0.041 | 0.002 | 0.005 | 0.000 | 0.000 | 0.870 | 1 |
| Porto Alegre | 21392 | 0.102 | 0.274 | 0.104 | 0.054 | 0.039 | 0.108 | 0.015 | 0.033 | 0.048 | 0.034 | 0.049 | 0.036 | 0.026 | 0.004 | 0.003 | 0.000 | 0.007 | 0.054 | 0.002 | 0.004 | 0.001 | 0.003 | NC | 0.587 |
| Porto Lucena | 192 | 0.145 | 0.280 | 0.117 | 0.060 | 0.013 | 0.120 | 0.010 | 0.034 | 0.039 | 0.036 | 0.026 | 0.042 | 0.016 | 0.005 | 0.000 | 0.000 | 0.008 | 0.042 | 0.000 | 0.003 | 0.003 | 0.002 | 0.860 | 0.968 |
| Porto Vera Cruz | 57 | 0.132 | 0.246 | 0.123 | 0.044 | 0.044 | 0.096 | 0.026 | 0.053 | 0.044 | 0.026 | 0.018 | 0.035 | 0.026 | 0.000 | 0.000 | 0.000 | 0.000 | 0.088 | 0.000 | 0.000 | 0.000 | 0.000 | 0.878 | 1 |
| Porto Xavier | 186 | 0.127 | 0.262 | 0.160 | 0.084 | 0.013 | 0.113 | 0.008 | 0.013 | 0.036 | 0.022 | 0.043 | 0.034 | 0.017 | 0.000 | 0.003 | 0.000 | 0.003 | 0.040 | 0.000 | 0.003 | 0.000 | 0.019 | 0.862 | 0.712 |
| Quinze De Novembro | 52 | 0.087 | 0.327 | 0.135 | 0.029 | 0.029 | 0.096 | 0.019 | 0.067 | 0.038 | 0.019 | 0.029 | 0.048 | 0.010 | 0.010 | 0.000 | 0.000 | 0.000 | 0.058 | 0.000 | 0.000 | 0.000 | 0.000 | 0.843 | 1 |
| Redentora | 106 | 0.113 | 0.302 | 0.151 | 0.042 | 0.028 | 0.099 | 0.005 | 0.024 | 0.028 | 0.042 | 0.052 | 0.038 | 0.014 | 0.000 | 0.000 | 0.000 | 0.000 | 0.061 | 0.000 | 0.000 | 0.000 | 0.000 | 0.850 | 1 |
| Rio Grande | 383 | 0.117 | 0.264 | 0.092 | 0.048 | 0.040 | 0.100 | 0.008 | 0.030 | 0.045 | 0.042 | 0.054 | 0.033 | 0.030 | 0.000 | 0.004 | 0.000 | 0.005 | 0.070 | 0.003 | 0.008 | 0.003 | 0.004 | 0.880 | 0.912 |
| Rio Pardo | 67 | 0.119 | 0.313 | 0.134 | 0.045 | 0.045 | 0.127 | 0.022 | 0.037 | 0.052 | 0.015 | 0.022 | 0.007 | 0.015 | 0.000 | 0.000 | 0.000 | 0.000 | 0.037 | 0.007 | 0.000 | 0.000 | 0.000 | 0.842 | 1 |
| Riozinho | 53 | 0.132 | 0.349 | 0.123 | 0.019 | 0.057 | 0.085 | 0.019 | 0.047 | 0.019 | 0.028 | 0.057 | 0.009 | 0.019 | 0.000 | 0.000 | 0.000 | 0.000 | 0.038 | 0.000 | 0.000 | 0.000 | 0.000 | 0.826 | 1 |
| Rolante | 103 | 0.086 | 0.276 | 0.121 | 0.058 | 0.049 | 0.073 | 0.024 | 0.019 | 0.028 | 0.048 | 0.058 | 0.044 | 0.034 | 0.000 | 0.000 | 0.000 | 0.019 | 0.044 | 0.005 | 0.000 | 0.010 | 0.005 | 0.878 | 0.980 |
| Rondinha | 99 | 0.076 | 0.283 | 0.091 | 0.081 | 0.045 | 0.146 | 0.030 | 0.045 | 0.035 | 0.035 | 0.030 | 0.040 | 0.000 | 0.000 | 0.005 | 0.000 | 0.010 | 0.045 | 0.000 | 0.000 | 0.000 | 0.000 | 0.866 | 1 |
| Salvador Do Sul | 66 | 0.097 | 0.210 | 0.225 | 0.066 | 0.008 | 0.105 | 0.008 | 0.015 | 0.043 | 0.000 | 0.030 | 0.083 | 0.015 | 0.008 | 0.008 | 0.000 | 0.008 | 0.061 | 0.000 | 0.000 | 0.000 | 0.013 | 0.867 | 0.882 |
| Sananduva | 110 | 0.082 | 0.318 | 0.100 | 0.045 | 0.041 | 0.109 | 0.045 | 0.064 | 0.005 | 0.032 | 0.009 | 0.041 | 0.045 | 0.000 | 0.000 | 0.000 | 0.014 | 0.050 | 0.000 | 0.000 | 0.000 | 0.000 | 0.853 | 1 |
| Santa Clara Do Sul | 57 | 0.149 | 0.316 | 0.193 | 0.044 | 0.000 | 0.096 | 0.000 | 0.026 | 0.000 | 0.000 | 0.000 | 0.088 | 0.000 | 0.000 | 0.000 | 0.000 | 0.018 | 0.070 | 0.000 | 0.000 | 0.000 | 0.000 | 0.816 | 1 |
| Santa Cruz Do Sul | 959 | 0.129 | 0.272 | 0.119 | 0.064 | 0.039 | 0.098 | 0.019 | 0.037 | 0.042 | 0.028 | 0.034 | 0.031 | 0.019 | 0.004 | 0.001 | 0.000 | 0.005 | 0.049 | 0.003 | 0.003 | 0.000 | 0.004 | 0.871 | 0.911 |
| Santa Maria | 1740 | 0.110 | 0.283 | 0.107 | 0.052 | 0.038 | 0.115 | 0.014 | 0.031 | 0.037 | 0.031 | 0.047 | 0.044 | 0.022 | 0.005 | 0.003 | 0.000 | 0.007 | 0.045 | 0.001 | 0.002 | 0.002 | 0.005 | 0.869 | 0.792 |
| Santa Rosa | 1916 | 0.126 | 0.271 | 0.134 | 0.055 | 0.031 | 0.090 | 0.018 | 0.038 | 0.037 | 0.026 | 0.049 | 0.035 | 0.017 | 0.001 | 0.002 | 0.000 | 0.007 | 0.051 | 0.001 | 0.003 | 0.000 | 0.007 | 0.870 | 0.679 |
| Santana Do Livramento | 500 | 0.093 | 0.281 | 0.102 | 0.044 | 0.029 | 0.113 | 0.021 | 0.029 | 0.043 | 0.031 | 0.064 | 0.032 | 0.036 | 0.002 | 0.010 | 0.000 | 0.007 | 0.058 | 0.000 | 0.004 | 0.000 | 0.003 | 0.872 | 0.949 |
| Santo Angelo | 468 | 0.117 | 0.220 | 0.117 | 0.062 | 0.029 | 0.113 | 0.019 | 0.026 | 0.050 | 0.029 | 0.063 | 0.034 | 0.018 | 0.004 | 0.005 | 0.000 | 0.012 | 0.072 | 0.002 | 0.003 | 0.000 | 0.003 | 0.891 | 0.941 |
| Santo Antonio Da Patrulha | 337 | 0.114 | 0.317 | 0.100 | 0.037 | 0.040 | 0.084 | 0.021 | 0.040 | 0.049 | 0.033 | 0.040 | 0.035 | 0.021 | 0.006 | 0.001 | 0.000 | 0.003 | 0.048 | 0.001 | 0.004 | 0.000 | 0.006 | 0.855 | 0.890 |
| Santo Cristo | 144 | 0.118 | 0.250 | 0.170 | 0.056 | 0.024 | 0.069 | 0.021 | 0.042 | 0.052 | 0.031 | 0.038 | 0.045 | 0.010 | 0.000 | 0.003 | 0.000 | 0.007 | 0.063 | 0.000 | 0.000 | 0.000 | 0.000 | 0.873 | 1 |
| Sao Jose Do Inhacora | 147 | 0.160 | 0.248 | 0.133 | 0.034 | 0.014 | 0.088 | 0.027 | 0.034 | 0.034 | 0.037 | 0.037 | 0.068 | 0.007 | 0.000 | 0.000 | 0.000 | 0.000 | 0.068 | 0.003 | 0.007 | 0.000 | 0.000 | 0.871 | 1 |
| Sao Jose Do Norte | 159 | 0.074 | 0.241 | 0.165 | 0.031 | 0.024 | 0.084 | 0.013 | 0.028 | 0.060 | 0.038 | 0.028 | 0.025 | 0.053 | 0.003 | 0.000 | 0.000 | 0.028 | 0.093 | 0.000 | 0.003 | 0.000 | 0.007 | 0.881 | 0.910 |
| Sao Leopoldo | 1780 | 0.109 | 0.286 | 0.105 | 0.053 | 0.032 | 0.100 | 0.017 | 0.034 | 0.051 | 0.030 | 0.049 | 0.035 | 0.025 | 0.005 | 0.002 | 0.000 | 0.006 | 0.051 | 0.002 | 0.004 | 0.001 | 0.003 | 0.870 | 0.894 |
| Sao Luiz Gonzaga | 291 | 0.113 | 0.256 | 0.127 | 0.045 | 0.027 | 0.113 | 0.010 | 0.027 | 0.058 | 0.024 | 0.076 | 0.041 | 0.024 | 0.003 | 0.002 | 0.000 | 0.002 | 0.050 | 0.000 | 0.000 | 0.000 | 0.000 | 0.874 | 1 |
| Sao Paulo Das Missoes | 144 | 0.128 | 0.212 | 0.160 | 0.049 | 0.049 | 0.111 | 0.021 | 0.028 | 0.028 | 0.014 | 0.052 | 0.056 | 0.028 | 0.000 | 0.007 | 0.000 | 0.003 | 0.052 | 0.003 | 0.000 | 0.000 | 0.000 | 0.885 | 1 |
| Sao Sebastiao Do Cai | 79 | 0.108 | 0.304 | 0.133 | 0.063 | 0.019 | 0.139 | 0.000 | 0.025 | 0.032 | 0.013 | 0.057 | 0.032 | 0.019 | 0.013 | 0.006 | 0.000 | 0.013 | 0.025 | 0.000 | 0.000 | 0.000 | 0.000 | 0.847 | 1 |
| **Town** | **N** | **A*01** | **A*02** | **A*03** | **A*11** | **A*23** | **A*24** | **A*25** | **A*26** | **A*29** | **A*30** | **A*31** | **A*32** | **A*33** | **A*34** | **A*36** | **A*43** | **A*66** | **A*68** | **A*69** | **A*74** | **A*80** | **blank** | **Exp H** | **P-HWE** |
| Sao Valerio Do Sul | 50 | 0.150 | 0.290 | 0.060 | 0.100 | 0.050 | 0.100 | 0.030 | 0.040 | 0.030 | 0.010 | 0.080 | 0.020 | 0.000 | 0.000 | 0.000 | 0.000 | 0.000 | 0.040 | 0.000 | 0.000 | 0.000 | 0.000 | 0.855 | 1 |
| Sapiranga | 1289 | 0.118 | 0.287 | 0.101 | 0.051 | 0.040 | 0.098 | 0.011 | 0.037 | 0.040 | 0.033 | 0.048 | 0.038 | 0.021 | 0.005 | 0.002 | 0.000 | 0.011 | 0.052 | 0.001 | 0.003 | 0.000 | 0.003 | 0.868 | 0.875 |
| Sapucaia Do Sul | 955 | 0.104 | 0.287 | 0.115 | 0.057 | 0.040 | 0.107 | 0.012 | 0.027 | 0.045 | 0.034 | 0.050 | 0.034 | 0.020 | 0.006 | 0.000 | 0.000 | 0.007 | 0.048 | 0.002 | 0.006 | 0.001 | 0.000 | 0.867 | 1 |
| Sarandi | 87 | 0.132 | 0.252 | 0.122 | 0.054 | 0.023 | 0.101 | 0.034 | 0.048 | 0.017 | 0.023 | 0.031 | 0.060 | 0.040 | 0.000 | 0.000 | 0.000 | 0.000 | 0.029 | 0.000 | 0.000 | 0.000 | 0.032 | 0.878 | 0.660 |
| Sede Nova | 135 | 0.163 | 0.263 | 0.178 | 0.041 | 0.044 | 0.081 | 0.004 | 0.033 | 0.019 | 0.026 | 0.052 | 0.037 | 0.004 | 0.000 | 0.000 | 0.000 | 0.004 | 0.033 | 0.000 | 0.019 | 0.000 | 0.000 | 0.855 | 1 |
| Serafina Correa | 69 | 0.145 | 0.239 | 0.123 | 0.014 | 0.029 | 0.138 | 0.029 | 0.065 | 0.043 | 0.029 | 0.058 | 0.014 | 0.022 | 0.007 | 0.000 | 0.000 | 0.000 | 0.043 | 0.000 | 0.000 | 0.000 | 0.000 | 0.873 | 1 |
| Tapejara | 50 | 0.120 | 0.233 | 0.078 | 0.050 | 0.020 | 0.127 | 0.000 | 0.060 | 0.040 | 0.060 | 0.080 | 0.030 | 0.030 | 0.000 | 0.000 | 0.000 | 0.010 | 0.050 | 0.000 | 0.000 | 0.000 | 0.013 | 0.887 | 0.899 |
| Taquara | 375 | 0.102 | 0.329 | 0.136 | 0.036 | 0.028 | 0.099 | 0.015 | 0.025 | 0.037 | 0.029 | 0.033 | 0.038 | 0.017 | 0.003 | 0.001 | 0.000 | 0.007 | 0.052 | 0.003 | 0.003 | 0.000 | 0.007 | 0.842 | 0.852 |
| Taquari | 101 | 0.089 | 0.287 | 0.069 | 0.054 | 0.069 | 0.074 | 0.015 | 0.020 | 0.074 | 0.059 | 0.050 | 0.025 | 0.010 | 0.015 | 0.005 | 0.000 | 0.010 | 0.074 | 0.000 | 0.000 | 0.000 | 0.000 | 0.873 | 1 |
| Tavares | 66 | 0.076 | 0.242 | 0.144 | 0.030 | 0.076 | 0.076 | 0.008 | 0.030 | 0.114 | 0.045 | 0.030 | 0.030 | 0.045 | 0.000 | 0.000 | 0.000 | 0.008 | 0.038 | 0.000 | 0.008 | 0.000 | 0.000 | 0.881 | 1 |
| Tenente Portela | 356 | 0.117 | 0.290 | 0.105 | 0.038 | 0.024 | 0.114 | 0.017 | 0.044 | 0.024 | 0.024 | 0.064 | 0.042 | 0.022 | 0.001 | 0.003 | 0.000 | 0.003 | 0.056 | 0.001 | 0.001 | 0.003 | 0.008 | 0.863 | 0.840 |
| Torres | 85 | 0.171 | 0.329 | 0.076 | 0.041 | 0.035 | 0.082 | 0.012 | 0.029 | 0.041 | 0.041 | 0.029 | 0.024 | 0.029 | 0.000 | 0.000 | 0.000 | 0.000 | 0.059 | 0.000 | 0.000 | 0.000 | 0.000 | 0.837 | 1 |
| Tramandai | 67 | 0.067 | 0.321 | 0.104 | 0.082 | 0.052 | 0.060 | 0.015 | 0.030 | 0.045 | 0.052 | 0.045 | 0.037 | 0.022 | 0.000 | 0.000 | 0.000 | 0.007 | 0.045 | 0.000 | 0.015 | 0.000 | 0.000 | 0.857 | 1 |
| Tres Coroas | 56 | 0.131 | 0.292 | 0.114 | 0.069 | 0.018 | 0.131 | 0.000 | 0.027 | 0.045 | 0.018 | 0.036 | 0.018 | 0.018 | 0.000 | 0.000 | 0.000 | 0.000 | 0.054 | 0.009 | 0.009 | 0.000 | 0.013 | 0.854 | 0.894 |
| Tres De Maio | 197 | 0.121 | 0.271 | 0.101 | 0.063 | 0.036 | 0.132 | 0.036 | 0.033 | 0.033 | 0.023 | 0.048 | 0.033 | 0.013 | 0.003 | 0.003 | 0.000 | 0.003 | 0.043 | 0.000 | 0.003 | 0.003 | 0.003 | 0.870 | 0.962 |
| Tres Passos | 326 | 0.140 | 0.294 | 0.141 | 0.043 | 0.026 | 0.092 | 0.023 | 0.034 | 0.032 | 0.014 | 0.038 | 0.043 | 0.017 | 0.000 | 0.002 | 0.000 | 0.011 | 0.044 | 0.002 | 0.003 | 0.002 | 0.000 | 0.854 | 1 |
| Triunfo | 85 | 0.111 | 0.249 | 0.129 | 0.063 | 0.024 | 0.124 | 0.012 | 0.018 | 0.075 | 0.035 | 0.029 | 0.018 | 0.041 | 0.006 | 0.000 | 0.000 | 0.012 | 0.036 | 0.000 | 0.006 | 0.000 | 0.014 | 0.877 | 0.858 |
| Tupancireta | 50 | 0.050 | 0.370 | 0.110 | 0.080 | 0.030 | 0.080 | 0.000 | 0.020 | 0.060 | 0.020 | 0.080 | 0.030 | 0.020 | 0.000 | 0.020 | 0.000 | 0.000 | 0.030 | 0.000 | 0.000 | 0.000 | 0.000 | 0.821 | 1 |
| Tuparendi | 243 | 0.140 | 0.263 | 0.103 | 0.060 | 0.035 | 0.132 | 0.029 | 0.049 | 0.037 | 0.025 | 0.037 | 0.039 | 0.014 | 0.000 | 0.002 | 0.000 | 0.004 | 0.029 | 0.000 | 0.002 | 0.000 | 0.000 | 0.869 | 1 |
| Uruguaiana | 1346 | 0.093 | 0.251 | 0.085 | 0.049 | 0.038 | 0.115 | 0.016 | 0.030 | 0.053 | 0.043 | 0.086 | 0.034 | 0.026 | 0.005 | 0.004 | 0.000 | 0.007 | 0.056 | 0.001 | 0.005 | 0.002 | 0.001 | 0.886 | 0.960 |
| Vacaria | 614 | 0.106 | 0.261 | 0.116 | 0.050 | 0.037 | 0.104 | 0.013 | 0.031 | 0.050 | 0.041 | 0.062 | 0.042 | 0.023 | 0.002 | 0.002 | 0.000 | 0.002 | 0.050 | 0.002 | 0.005 | 0.002 | 0.000 | 0.879 | 1 |
| Venancio Aires | 71 | 0.148 | 0.275 | 0.120 | 0.085 | 0.042 | 0.077 | 0.021 | 0.021 | 0.028 | 0.028 | 0.014 | 0.056 | 0.007 | 0.000 | 0.000 | 0.000 | 0.007 | 0.070 | 0.000 | 0.000 | 0.000 | 0.000 | 0.863 | 1 |
| Vera Cruz | 57 | 0.219 | 0.289 | 0.105 | 0.061 | 0.035 | 0.079 | 0.018 | 0.009 | 0.035 | 0.009 | 0.018 | 0.061 | 0.009 | 0.000 | 0.000 | 0.000 | 0.000 | 0.053 | 0.000 | 0.000 | 0.000 | 0.000 | 0.837 | 1 |
| Viamao | 1327 | 0.105 | 0.253 | 0.104 | 0.057 | 0.038 | 0.109 | 0.009 | 0.035 | 0.053 | 0.035 | 0.053 | 0.033 | 0.031 | 0.005 | 0.003 | 0.000 | 0.006 | 0.059 | 0.003 | 0.007 | 0.001 | 0.000 | 0.884 | 1 |

**HLA-B**

| **Town** | **N** | **B*07** | **B*08** | **B*13** | **B*14** | **B*15** | **B*18** | **B*27** | **B*35** | **B*37** | **B*38** | **B*39** | **B*40** | **B*41** | **B*42** | **B*44** | **B*45** | **B*46** | **B*47** | **B*48** | **B*49** | **B*50** | **B*51** | **B*52** | **B*53** | **B*54** | **B*55** | **B*56** | **B*57** | **B*58** | **B*59** | **B*67** | **B*73** | **B*78** | **B*81** | **B*82** | **blank** | **Exp H** | **P-HWE** |
| --- | --- | --- | --- | --- | --- | --- | --- | --- | --- | --- | --- | --- | --- | --- | --- | --- | --- | --- | --- | --- | --- | --- | --- | --- | --- | --- | --- | --- | --- | --- | --- | --- | --- | --- | --- | --- | --- | --- | --- |
| Agudo | 99 | 0.061 | 0.076 | 0.015 | 0.040 | 0.076 | 0.040 | 0.040 | 0.136 | 0.000 | 0.015 | 0.030 | 0.081 | 0.005 | 0.000 | 0.157 | 0.015 | 0.000 | 0.005 | 0.005 | 0.010 | 0.010 | 0.076 | 0.015 | 0.005 | 0.000 | 0.010 | 0.010 | 0.035 | 0.030 | 0.000 | 0.000 | 0.000 | 0.000 | 0.000 | 0.000 | 0.000 | 0.920 | 1 |
| Alegrete | 1259 | 0.070 | 0.050 | 0.011 | 0.071 | 0.093 | 0.050 | 0.021 | 0.127 | 0.007 | 0.027 | 0.042 | 0.063 | 0.010 | 0.009 | 0.098 | 0.014 | 0.000 | 0.002 | 0.018 | 0.017 | 0.020 | 0.083 | 0.015 | 0.016 | 0.000 | 0.007 | 0.004 | 0.023 | 0.028 | 0.000 | 0.000 | 0.000 | 0.000 | 0.004 | 0.000 | 0.002 | 0.933 | 0.935 |
| Alvorada | 841 | 0.066 | 0.057 | 0.013 | 0.063 | 0.073 | 0.041 | 0.023 | 0.126 | 0.008 | 0.029 | 0.042 | 0.046 | 0.012 | 0.004 | 0.125 | 0.021 | 0.000 | 0.001 | 0.008 | 0.022 | 0.028 | 0.083 | 0.018 | 0.013 | 0.000 | 0.008 | 0.004 | 0.030 | 0.027 | 0.000 | 0.000 | 0.000 | 0.000 | 0.001 | 0.000 | 0.006 | 0.933 | 0.814 |
| Ametista Do Sul | 62 | 0.106 | 0.053 | 0.024 | 0.073 | 0.081 | 0.048 | 0.024 | 0.125 | 0.008 | 0.024 | 0.024 | 0.053 | 0.024 | 0.000 | 0.121 | 0.032 | 0.000 | 0.000 | 0.008 | 0.024 | 0.008 | 0.070 | 0.008 | 0.000 | 0.000 | 0.000 | 0.000 | 0.024 | 0.016 | 0.000 | 0.000 | 0.000 | 0.000 | 0.000 | 0.000 | 0.021 | 0.928 | 0.807 |
| Arambare | 60 | 0.067 | 0.045 | 0.008 | 0.058 | 0.083 | 0.042 | 0.025 | 0.108 | 0.000 | 0.045 | 0.050 | 0.045 | 0.027 | 0.017 | 0.088 | 0.042 | 0.000 | 0.000 | 0.000 | 0.017 | 0.025 | 0.067 | 0.008 | 0.017 | 0.000 | 0.017 | 0.000 | 0.033 | 0.017 | 0.000 | 0.000 | 0.000 | 0.000 | 0.017 | 0.000 | 0.032 | 0.943 | 0.716 |
| Aratiba | 130 | 0.092 | 0.068 | 0.053 | 0.052 | 0.058 | 0.095 | 0.012 | 0.083 | 0.019 | 0.023 | 0.046 | 0.050 | 0.019 | 0.000 | 0.084 | 0.004 | 0.000 | 0.000 | 0.012 | 0.012 | 0.019 | 0.087 | 0.012 | 0.012 | 0.000 | 0.012 | 0.004 | 0.057 | 0.004 | 0.000 | 0.004 | 0.000 | 0.000 | 0.000 | 0.000 | 0.010 | 0.937 | 0.953 |
| Arroio Do Meio | 86 | 0.110 | 0.116 | 0.047 | 0.064 | 0.041 | 0.047 | 0.041 | 0.128 | 0.023 | 0.058 | 0.017 | 0.035 | 0.006 | 0.006 | 0.076 | 0.012 | 0.000 | 0.000 | 0.006 | 0.023 | 0.006 | 0.081 | 0.012 | 0.000 | 0.000 | 0.006 | 0.006 | 0.023 | 0.012 | 0.000 | 0.000 | 0.000 | 0.000 | 0.000 | 0.000 | 0.000 | 0.927 | 1 |
| Arroio Do Sal | 507 | 0.067 | 0.058 | 0.016 | 0.064 | 0.076 | 0.036 | 0.036 | 0.135 | 0.009 | 0.031 | 0.033 | 0.039 | 0.004 | 0.002 | 0.135 | 0.013 | 0.000 | 0.001 | 0.011 | 0.019 | 0.031 | 0.095 | 0.007 | 0.004 | 0.000 | 0.015 | 0.008 | 0.028 | 0.026 | 0.000 | 0.000 | 0.000 | 0.000 | 0.003 | 0.000 | 0.000 | 0.927 | 1 |
| Augusto Pestana | 65 | 0.108 | 0.146 | 0.038 | 0.008 | 0.046 | 0.023 | 0.023 | 0.069 | 0.038 | 0.008 | 0.031 | 0.077 | 0.008 | 0.015 | 0.108 | 0.008 | 0.000 | 0.008 | 0.008 | 0.008 | 0.015 | 0.131 | 0.000 | 0.000 | 0.000 | 0.000 | 0.008 | 0.046 | 0.008 | 0.000 | 0.000 | 0.015 | 0.000 | 0.000 | 0.000 | 0.000 | 0.917 | 1 |
| Bento Goncalves | 248 | 0.050 | 0.093 | 0.022 | 0.036 | 0.056 | 0.060 | 0.028 | 0.127 | 0.008 | 0.026 | 0.046 | 0.058 | 0.012 | 0.002 | 0.117 | 0.006 | 0.000 | 0.004 | 0.002 | 0.028 | 0.004 | 0.109 | 0.024 | 0.002 | 0.000 | 0.016 | 0.002 | 0.038 | 0.016 | 0.000 | 0.000 | 0.004 | 0.000 | 0.000 | 0.000 | 0.000 | 0.928 | 1 |
| Boa Vista Do Burica | 173 | 0.116 | 0.092 | 0.009 | 0.020 | 0.092 | 0.058 | 0.029 | 0.124 | 0.009 | 0.026 | 0.032 | 0.061 | 0.006 | 0.000 | 0.136 | 0.006 | 0.000 | 0.006 | 0.003 | 0.012 | 0.003 | 0.095 | 0.012 | 0.006 | 0.000 | 0.014 | 0.009 | 0.020 | 0.006 | 0.000 | 0.000 | 0.000 | 0.000 | 0.000 | 0.000 | 0.000 | 0.915 | 1 |
| Cachoeira Do Sul | 58 | 0.060 | 0.026 | 0.009 | 0.060 | 0.060 | 0.043 | 0.034 | 0.155 | 0.009 | 0.026 | 0.026 | 0.078 | 0.034 | 0.000 | 0.103 | 0.009 | 0.000 | 0.000 | 0.000 | 0.017 | 0.017 | 0.121 | 0.000 | 0.009 | 0.000 | 0.034 | 0.009 | 0.026 | 0.034 | 0.000 | 0.000 | 0.000 | 0.000 | 0.000 | 0.000 | 0.000 | 0.923 | 1 |
| Cachoeirinha | 1139 | 0.076 | 0.049 | 0.011 | 0.053 | 0.069 | 0.047 | 0.025 | 0.140 | 0.012 | 0.025 | 0.033 | 0.047 | 0.015 | 0.003 | 0.130 | 0.016 | 0.000 | 0.001 | 0.008 | 0.019 | 0.025 | 0.085 | 0.015 | 0.018 | 0.000 | 0.015 | 0.008 | 0.028 | 0.020 | 0.000 | 0.000 | 0.000 | 0.000 | 0.001 | 0.000 | 0.005 | 0.930 | 0.834 |
| Caibate | 88 | 0.114 | 0.085 | 0.017 | 0.085 | 0.131 | 0.034 | 0.045 | 0.091 | 0.006 | 0.040 | 0.006 | 0.074 | 0.006 | 0.000 | 0.080 | 0.017 | 0.000 | 0.000 | 0.000 | 0.011 | 0.006 | 0.097 | 0.000 | 0.000 | 0.000 | 0.017 | 0.000 | 0.023 | 0.017 | 0.000 | 0.000 | 0.000 | 0.000 | 0.000 | 0.000 | 0.000 | 0.919 | 1 |
| Camaqua | 114 | 0.100 | 0.109 | 0.004 | 0.044 | 0.059 | 0.032 | 0.041 | 0.096 | 0.009 | 0.031 | 0.019 | 0.066 | 0.026 | 0.000 | 0.135 | 0.013 | 0.000 | 0.000 | 0.004 | 0.018 | 0.018 | 0.070 | 0.013 | 0.004 | 0.000 | 0.013 | 0.013 | 0.026 | 0.004 | 0.000 | 0.000 | 0.000 | 0.000 | 0.000 | 0.000 | 0.032 | 0.928 | 0.612 |
| Campestre Da Serra | 92 | 0.062 | 0.043 | 0.047 | 0.016 | 0.080 | 0.064 | 0.022 | 0.156 | 0.016 | 0.038 | 0.054 | 0.022 | 0.038 | 0.005 | 0.058 | 0.005 | 0.000 | 0.005 | 0.005 | 0.027 | 0.016 | 0.108 | 0.027 | 0.005 | 0.000 | 0.005 | 0.000 | 0.049 | 0.011 | 0.000 | 0.000 | 0.000 | 0.000 | 0.000 | 0.000 | 0.010 | 0.930 | 0.890 |
| Campina Das Missoes | 78 | 0.122 | 0.109 | 0.026 | 0.064 | 0.051 | 0.045 | 0.000 | 0.096 | 0.013 | 0.045 | 0.019 | 0.077 | 0.000 | 0.000 | 0.122 | 0.000 | 0.000 | 0.000 | 0.000 | 0.026 | 0.013 | 0.090 | 0.006 | 0.000 | 0.000 | 0.013 | 0.006 | 0.038 | 0.019 | 0.000 | 0.000 | 0.000 | 0.000 | 0.000 | 0.000 | 0.000 | 0.920 | 1 |
| Campo Bom | 612 | 0.085 | 0.064 | 0.016 | 0.056 | 0.089 | 0.045 | 0.023 | 0.120 | 0.008 | 0.020 | 0.036 | 0.053 | 0.011 | 0.007 | 0.115 | 0.020 | 0.000 | 0.002 | 0.007 | 0.027 | 0.018 | 0.081 | 0.014 | 0.011 | 0.000 | 0.008 | 0.008 | 0.033 | 0.016 | 0.000 | 0.000 | 0.000 | 0.000 | 0.002 | 0.000 | 0.005 | 0.932 | 0.877 |
| Campo Novo | 177 | 0.087 | 0.068 | 0.023 | 0.048 | 0.059 | 0.059 | 0.011 | 0.118 | 0.011 | 0.037 | 0.025 | 0.067 | 0.011 | 0.003 | 0.099 | 0.006 | 0.000 | 0.000 | 0.006 | 0.017 | 0.014 | 0.079 | 0.025 | 0.003 | 0.000 | 0.036 | 0.008 | 0.045 | 0.031 | 0.000 | 0.000 | 0.000 | 0.000 | 0.000 | 0.000 | 0.002 | 0.935 | 0.996 |
| Candido Godoi | 172 | 0.128 | 0.087 | 0.052 | 0.026 | 0.073 | 0.041 | 0.041 | 0.076 | 0.009 | 0.029 | 0.023 | 0.049 | 0.009 | 0.003 | 0.110 | 0.003 | 0.000 | 0.000 | 0.000 | 0.044 | 0.006 | 0.105 | 0.006 | 0.020 | 0.000 | 0.006 | 0.006 | 0.044 | 0.006 | 0.000 | 0.000 | 0.000 | 0.000 | 0.000 | 0.000 | 0.000 | 0.927 | 1 |
| Canela | 51 | 0.039 | 0.075 | 0.039 | 0.054 | 0.098 | 0.049 | 0.029 | 0.075 | 0.020 | 0.039 | 0.010 | 0.078 | 0.010 | 0.000 | 0.122 | 0.010 | 0.000 | 0.000 | 0.000 | 0.029 | 0.000 | 0.108 | 0.000 | 0.010 | 0.000 | 0.020 | 0.010 | 0.034 | 0.020 | 0.000 | 0.000 | 0.000 | 0.000 | 0.000 | 0.000 | 0.023 | 0.932 | 0.812 |
| Canoas | 3820 | 0.080 | 0.062 | 0.014 | 0.057 | 0.084 | 0.050 | 0.025 | 0.121 | 0.011 | 0.023 | 0.037 | 0.046 | 0.013 | 0.005 | 0.120 | 0.012 | 0.000 | 0.003 | 0.007 | 0.025 | 0.021 | 0.088 | 0.015 | 0.014 | 0.000 | 0.011 | 0.005 | 0.030 | 0.018 | 0.000 | 0.001 | 0.000 | 0.000 | 0.002 | 0.000 | 0.001 | 0.932 | 0.945 |
| Capao Da Canoa | 104 | 0.082 | 0.067 | 0.010 | 0.063 | 0.072 | 0.043 | 0.010 | 0.149 | 0.014 | 0.019 | 0.029 | 0.063 | 0.014 | 0.010 | 0.077 | 0.014 | 0.000 | 0.005 | 0.014 | 0.000 | 0.038 | 0.101 | 0.005 | 0.029 | 0.000 | 0.024 | 0.005 | 0.019 | 0.024 | 0.000 | 0.000 | 0.000 | 0.000 | 0.000 | 0.000 | 0.000 | 0.929 | 1 |
| Carazinho | 314 | 0.084 | 0.085 | 0.016 | 0.034 | 0.088 | 0.051 | 0.029 | 0.137 | 0.008 | 0.029 | 0.038 | 0.049 | 0.008 | 0.005 | 0.123 | 0.013 | 0.000 | 0.000 | 0.006 | 0.016 | 0.011 | 0.088 | 0.013 | 0.005 | 0.000 | 0.013 | 0.006 | 0.021 | 0.016 | 0.000 | 0.000 | 0.000 | 0.000 | 0.000 | 0.000 | 0.009 | 0.925 | 0.824 |
| Catuipe | 52 | 0.077 | 0.096 | 0.029 | 0.019 | 0.087 | 0.087 | 0.038 | 0.115 | 0.010 | 0.058 | 0.019 | 0.038 | 0.010 | 0.000 | 0.067 | 0.010 | 0.000 | 0.010 | 0.010 | 0.048 | 0.010 | 0.096 | 0.000 | 0.010 | 0.000 | 0.010 | 0.000 | 0.029 | 0.019 | 0.000 | 0.000 | 0.000 | 0.000 | 0.000 | 0.000 | 0.000 | 0.931 | 1 |
| Caxias Do Sul | 4959 | 0.074 | 0.064 | 0.023 | 0.042 | 0.080 | 0.054 | 0.025 | 0.133 | 0.011 | 0.027 | 0.035 | 0.051 | 0.013 | 0.004 | 0.108 | 0.013 | 0.000 | 0.003 | 0.005 | 0.022 | 0.018 | 0.100 | 0.015 | 0.011 | 0.000 | 0.017 | 0.006 | 0.029 | 0.014 | 0.000 | 0.000 | 0.001 | 0.000 | 0.001 | 0.000 | 0.000 | 0.931 | 0.977 |
| Cerro Largo | 59 | 0.126 | 0.075 | 0.034 | 0.042 | 0.076 | 0.066 | 0.048 | 0.144 | 0.008 | 0.017 | 0.048 | 0.042 | 0.017 | 0.000 | 0.059 | 0.008 | 0.000 | 0.000 | 0.008 | 0.034 | 0.008 | 0.051 | 0.000 | 0.000 | 0.000 | 0.025 | 0.000 | 0.034 | 0.017 | 0.000 | 0.000 | 0.000 | 0.000 | 0.000 | 0.000 | 0.010 | 0.928 | 0.928 |
| Charqueadas | 107 | 0.103 | 0.033 | 0.019 | 0.051 | 0.079 | 0.056 | 0.023 | 0.168 | 0.005 | 0.014 | 0.009 | 0.042 | 0.000 | 0.009 | 0.103 | 0.028 | 0.000 | 0.005 | 0.009 | 0.009 | 0.023 | 0.103 | 0.019 | 0.014 | 0.000 | 0.009 | 0.009 | 0.023 | 0.028 | 0.000 | 0.000 | 0.000 | 0.000 | 0.005 | 0.000 | 0.000 | 0.920 | 1 |
| Crissiumal | 277 | 0.101 | 0.078 | 0.034 | 0.031 | 0.072 | 0.056 | 0.022 | 0.112 | 0.018 | 0.018 | 0.032 | 0.103 | 0.009 | 0.000 | 0.125 | 0.007 | 0.000 | 0.002 | 0.000 | 0.027 | 0.016 | 0.069 | 0.014 | 0.005 | 0.000 | 0.018 | 0.005 | 0.022 | 0.004 | 0.000 | 0.000 | 0.000 | 0.000 | 0.000 | 0.000 | 0.000 | 0.926 | 1 |
| Cruz Alta | 1977 | 0.082 | 0.058 | 0.020 | 0.056 | 0.080 | 0.051 | 0.028 | 0.130 | 0.012 | 0.025 | 0.034 | 0.049 | 0.014 | 0.003 | 0.115 | 0.013 | 0.000 | 0.001 | 0.009 | 0.019 | 0.020 | 0.083 | 0.016 | 0.009 | 0.000 | 0.014 | 0.005 | 0.032 | 0.019 | 0.000 | 0.000 | 0.002 | 0.000 | 0.003 | 0.001 | 0.000 | 0.932 | 1 |
| **Town** | **N** | **B*07** | **B*08** | **B*13** | **B*14** | **B*15** | **B*18** | **B*27** | **B*35** | **B*37** | **B*38** | **B*39** | **B*40** | **B*41** | **B*42** | **B*44** | **B*45** | **B*46** | **B*47** | **B*48** | **B*49** | **B*50** | **B*51** | **B*52** | **B*53** | **B*54** | **B*55** | **B*56** | **B*57** | **B*58** | **B*59** | **B*67** | **B*73** | **B*78** | **B*81** | **B*82** | **blank** | **Exp H** | **P-HWE** |
| Cruzeiro Do Sul | 79 | 0.127 | 0.076 | 0.032 | 0.076 | 0.057 | 0.032 | 0.038 | 0.146 | 0.006 | 0.025 | 0.051 | 0.089 | 0.025 | 0.000 | 0.082 | 0.000 | 0.000 | 0.000 | 0.000 | 0.013 | 0.006 | 0.063 | 0.013 | 0.006 | 0.000 | 0.000 | 0.006 | 0.019 | 0.013 | 0.000 | 0.000 | 0.000 | 0.000 | 0.000 | 0.000 | 0.000 | 0.921 | 1 |
| Dois Irmaos | 163 | 0.129 | 0.101 | 0.043 | 0.025 | 0.046 | 0.046 | 0.043 | 0.129 | 0.009 | 0.021 | 0.025 | 0.046 | 0.006 | 0.000 | 0.083 | 0.018 | 0.000 | 0.000 | 0.015 | 0.021 | 0.015 | 0.067 | 0.012 | 0.000 | 0.000 | 0.018 | 0.006 | 0.058 | 0.012 | 0.000 | 0.000 | 0.000 | 0.000 | 0.003 | 0.000 | 0.000 | 0.928 | 1 |
| Doutor Mauricio Cardoso | 172 | 0.113 | 0.067 | 0.012 | 0.049 | 0.087 | 0.081 | 0.029 | 0.087 | 0.009 | 0.020 | 0.023 | 0.049 | 0.020 | 0.006 | 0.145 | 0.009 | 0.000 | 0.000 | 0.000 | 0.015 | 0.009 | 0.078 | 0.009 | 0.012 | 0.000 | 0.015 | 0.000 | 0.049 | 0.006 | 0.000 | 0.000 | 0.000 | 0.000 | 0.000 | 0.000 | 0.003 | 0.923 | 0.964 |
| Eldorado Do Sul | 175 | 0.066 | 0.071 | 0.029 | 0.054 | 0.089 | 0.046 | 0.017 | 0.109 | 0.003 | 0.029 | 0.046 | 0.066 | 0.009 | 0.006 | 0.106 | 0.020 | 0.000 | 0.009 | 0.020 | 0.020 | 0.029 | 0.060 | 0.017 | 0.011 | 0.000 | 0.014 | 0.009 | 0.037 | 0.011 | 0.000 | 0.000 | 0.000 | 0.000 | 0.000 | 0.000 | 0.000 | 0.938 | 1 |
| Encantado | 214 | 0.076 | 0.095 | 0.027 | 0.030 | 0.063 | 0.051 | 0.035 | 0.116 | 0.012 | 0.028 | 0.037 | 0.049 | 0.002 | 0.005 | 0.111 | 0.005 | 0.000 | 0.002 | 0.007 | 0.021 | 0.016 | 0.116 | 0.014 | 0.002 | 0.000 | 0.026 | 0.005 | 0.021 | 0.021 | 0.000 | 0.000 | 0.000 | 0.000 | 0.000 | 0.000 | 0.006 | 0.929 | 0.900 |
| Erechim | 663 | 0.075 | 0.068 | 0.029 | 0.037 | 0.068 | 0.069 | 0.026 | 0.150 | 0.016 | 0.030 | 0.029 | 0.042 | 0.008 | 0.003 | 0.111 | 0.012 | 0.000 | 0.003 | 0.002 | 0.021 | 0.011 | 0.094 | 0.014 | 0.008 | 0.000 | 0.021 | 0.005 | 0.030 | 0.011 | 0.000 | 0.000 | 0.003 | 0.000 | 0.000 | 0.000 | 0.002 | 0.928 | 0.938 |
| Espumoso | 107 | 0.051 | 0.083 | 0.023 | 0.028 | 0.095 | 0.065 | 0.037 | 0.081 | 0.019 | 0.005 | 0.037 | 0.037 | 0.019 | 0.000 | 0.152 | 0.000 | 0.000 | 0.005 | 0.023 | 0.009 | 0.014 | 0.117 | 0.023 | 0.009 | 0.000 | 0.023 | 0.000 | 0.026 | 0.005 | 0.000 | 0.000 | 0.000 | 0.000 | 0.000 | 0.000 | 0.011 | 0.925 | 0.867 |
| Estancia Velha | 883 | 0.092 | 0.084 | 0.021 | 0.054 | 0.076 | 0.039 | 0.037 | 0.113 | 0.014 | 0.020 | 0.028 | 0.070 | 0.007 | 0.001 | 0.125 | 0.010 | 0.000 | 0.002 | 0.005 | 0.023 | 0.017 | 0.079 | 0.013 | 0.012 | 0.000 | 0.015 | 0.008 | 0.024 | 0.012 | 0.000 | 0.000 | 0.001 | 0.000 | 0.001 | 0.000 | 0.000 | 0.930 | 1 |
| Esteio | 654 | 0.054 | 0.054 | 0.019 | 0.048 | 0.069 | 0.048 | 0.029 | 0.132 | 0.007 | 0.028 | 0.037 | 0.056 | 0.011 | 0.002 | 0.132 | 0.014 | 0.000 | 0.002 | 0.005 | 0.032 | 0.023 | 0.088 | 0.017 | 0.009 | 0.000 | 0.018 | 0.003 | 0.029 | 0.023 | 0.000 | 0.001 | 0.001 | 0.002 | 0.001 | 0.000 | 0.005 | 0.932 | 0.850 |
| Estrela | 799 | 0.117 | 0.071 | 0.023 | 0.038 | 0.073 | 0.047 | 0.034 | 0.118 | 0.015 | 0.027 | 0.030 | 0.072 | 0.008 | 0.001 | 0.102 | 0.003 | 0.000 | 0.002 | 0.002 | 0.022 | 0.025 | 0.074 | 0.014 | 0.005 | 0.000 | 0.011 | 0.006 | 0.037 | 0.016 | 0.000 | 0.000 | 0.001 | 0.000 | 0.002 | 0.000 | 0.005 | 0.931 | 0.849 |
| Farroupilha | 2347 | 0.072 | 0.070 | 0.027 | 0.039 | 0.076 | 0.069 | 0.027 | 0.135 | 0.013 | 0.026 | 0.034 | 0.047 | 0.010 | 0.002 | 0.100 | 0.006 | 0.000 | 0.003 | 0.004 | 0.019 | 0.016 | 0.115 | 0.013 | 0.009 | 0.000 | 0.017 | 0.006 | 0.026 | 0.014 | 0.000 | 0.000 | 0.001 | 0.000 | 0.000 | 0.000 | 0.004 | 0.929 | 0.783 |
| Flores Da Cunha | 56 | 0.063 | 0.063 | 0.036 | 0.036 | 0.087 | 0.054 | 0.009 | 0.098 | 0.000 | 0.018 | 0.018 | 0.054 | 0.009 | 0.000 | 0.169 | 0.000 | 0.000 | 0.000 | 0.009 | 0.027 | 0.018 | 0.124 | 0.000 | 0.000 | 0.000 | 0.018 | 0.000 | 0.051 | 0.022 | 0.000 | 0.000 | 0.009 | 0.000 | 0.000 | 0.000 | 0.012 | 0.917 | 0.903 |
| Garibaldi | 65 | 0.085 | 0.131 | 0.031 | 0.023 | 0.046 | 0.085 | 0.023 | 0.146 | 0.015 | 0.031 | 0.031 | 0.054 | 0.008 | 0.000 | 0.077 | 0.015 | 0.000 | 0.000 | 0.000 | 0.000 | 0.015 | 0.138 | 0.000 | 0.000 | 0.000 | 0.015 | 0.015 | 0.008 | 0.008 | 0.000 | 0.000 | 0.000 | 0.000 | 0.000 | 0.000 | 0.000 | 0.912 | 1 |
| Getulio Vargas | 90 | 0.061 | 0.044 | 0.033 | 0.039 | 0.072 | 0.044 | 0.017 | 0.161 | 0.006 | 0.022 | 0.039 | 0.039 | 0.011 | 0.011 | 0.083 | 0.006 | 0.000 | 0.000 | 0.006 | 0.017 | 0.022 | 0.178 | 0.022 | 0.006 | 0.000 | 0.022 | 0.006 | 0.017 | 0.011 | 0.000 | 0.000 | 0.000 | 0.000 | 0.006 | 0.000 | 0.000 | 0.914 | 1 |
| Gravatai | 3217 | 0.069 | 0.062 | 0.018 | 0.055 | 0.075 | 0.042 | 0.030 | 0.129 | 0.014 | 0.022 | 0.032 | 0.048 | 0.012 | 0.004 | 0.122 | 0.012 | 0.000 | 0.002 | 0.009 | 0.027 | 0.022 | 0.087 | 0.018 | 0.015 | 0.000 | 0.013 | 0.007 | 0.031 | 0.021 | 0.000 | 0.000 | 0.000 | 0.000 | 0.001 | 0.001 | 0.000 | 0.933 | 1 |
| Guaiba | 469 | 0.060 | 0.059 | 0.014 | 0.061 | 0.081 | 0.043 | 0.023 | 0.114 | 0.004 | 0.017 | 0.041 | 0.053 | 0.013 | 0.001 | 0.141 | 0.009 | 0.000 | 0.002 | 0.014 | 0.021 | 0.030 | 0.096 | 0.012 | 0.018 | 0.000 | 0.011 | 0.011 | 0.028 | 0.013 | 0.000 | 0.000 | 0.000 | 0.001 | 0.001 | 0.000 | 0.008 | 0.930 | 0.809 |
| Humaita | 58 | 0.103 | 0.052 | 0.000 | 0.017 | 0.085 | 0.009 | 0.034 | 0.095 | 0.017 | 0.026 | 0.017 | 0.060 | 0.017 | 0.009 | 0.155 | 0.009 | 0.000 | 0.000 | 0.009 | 0.026 | 0.026 | 0.121 | 0.000 | 0.000 | 0.000 | 0.026 | 0.034 | 0.026 | 0.009 | 0.000 | 0.000 | 0.000 | 0.000 | 0.013 | 0.000 | 0.006 | 0.921 | 0.982 |
| Ibiruba | 93 | 0.102 | 0.097 | 0.016 | 0.048 | 0.075 | 0.043 | 0.016 | 0.086 | 0.005 | 0.005 | 0.027 | 0.070 | 0.000 | 0.000 | 0.156 | 0.011 | 0.000 | 0.000 | 0.000 | 0.027 | 0.011 | 0.124 | 0.016 | 0.000 | 0.000 | 0.000 | 0.027 | 0.027 | 0.011 | 0.000 | 0.000 | 0.000 | 0.000 | 0.000 | 0.000 | 0.000 | 0.914 | 1 |
| Igrejinha | 279 | 0.091 | 0.075 | 0.009 | 0.023 | 0.076 | 0.043 | 0.039 | 0.115 | 0.014 | 0.017 | 0.027 | 0.073 | 0.013 | 0.000 | 0.127 | 0.011 | 0.000 | 0.000 | 0.007 | 0.028 | 0.013 | 0.070 | 0.022 | 0.009 | 0.000 | 0.013 | 0.016 | 0.043 | 0.009 | 0.000 | 0.000 | 0.000 | 0.000 | 0.004 | 0.000 | 0.013 | 0.931 | 0.747 |
| Ijui | 561 | 0.098 | 0.073 | 0.029 | 0.043 | 0.077 | 0.045 | 0.029 | 0.118 | 0.018 | 0.022 | 0.034 | 0.062 | 0.012 | 0.005 | 0.102 | 0.012 | 0.000 | 0.002 | 0.008 | 0.018 | 0.014 | 0.088 | 0.011 | 0.004 | 0.000 | 0.022 | 0.006 | 0.020 | 0.012 | 0.000 | 0.000 | 0.000 | 0.000 | 0.001 | 0.001 | 0.013 | 0.933 | 0.651 |
| Itaqui | 142 | 0.060 | 0.032 | 0.014 | 0.056 | 0.102 | 0.053 | 0.039 | 0.106 | 0.018 | 0.018 | 0.063 | 0.053 | 0.028 | 0.007 | 0.123 | 0.007 | 0.000 | 0.000 | 0.021 | 0.011 | 0.018 | 0.070 | 0.025 | 0.011 | 0.000 | 0.021 | 0.000 | 0.011 | 0.028 | 0.000 | 0.000 | 0.000 | 0.000 | 0.007 | 0.000 | 0.000 | 0.935 | 1 |
| Ivoti | 325 | 0.102 | 0.072 | 0.014 | 0.043 | 0.082 | 0.068 | 0.034 | 0.131 | 0.017 | 0.028 | 0.034 | 0.062 | 0.014 | 0.006 | 0.102 | 0.003 | 0.000 | 0.005 | 0.000 | 0.026 | 0.005 | 0.074 | 0.012 | 0.005 | 0.000 | 0.015 | 0.003 | 0.025 | 0.022 | 0.000 | 0.000 | 0.000 | 0.000 | 0.000 | 0.000 | 0.000 | 0.929 | 1 |
| Jacutinga | 154 | 0.097 | 0.078 | 0.019 | 0.065 | 0.065 | 0.078 | 0.029 | 0.114 | 0.026 | 0.016 | 0.052 | 0.036 | 0.006 | 0.003 | 0.071 | 0.000 | 0.000 | 0.000 | 0.003 | 0.016 | 0.003 | 0.127 | 0.013 | 0.013 | 0.000 | 0.013 | 0.003 | 0.036 | 0.010 | 0.000 | 0.000 | 0.006 | 0.000 | 0.000 | 0.000 | 0.000 | 0.927 | 1 |
| Lagoa Vermelha | 77 | 0.071 | 0.058 | 0.006 | 0.065 | 0.110 | 0.045 | 0.006 | 0.084 | 0.006 | 0.052 | 0.013 | 0.039 | 0.006 | 0.006 | 0.084 | 0.019 | 0.000 | 0.000 | 0.013 | 0.006 | 0.032 | 0.091 | 0.032 | 0.019 | 0.000 | 0.006 | 0.039 | 0.052 | 0.032 | 0.000 | 0.000 | 0.000 | 0.000 | 0.000 | 0.000 | 0.000 | 0.938 | 1 |
| Lajeado | 1484 | 0.112 | 0.082 | 0.028 | 0.037 | 0.074 | 0.053 | 0.040 | 0.125 | 0.010 | 0.026 | 0.032 | 0.054 | 0.009 | 0.001 | 0.105 | 0.006 | 0.000 | 0.002 | 0.002 | 0.020 | 0.014 | 0.088 | 0.011 | 0.005 | 0.000 | 0.014 | 0.005 | 0.030 | 0.012 | 0.000 | 0.000 | 0.000 | 0.000 | 0.001 | 0.000 | 0.000 | 0.927 | 1 |
| Marau | 188 | 0.058 | 0.071 | 0.031 | 0.044 | 0.084 | 0.071 | 0.029 | 0.157 | 0.011 | 0.016 | 0.042 | 0.040 | 0.013 | 0.003 | 0.101 | 0.005 | 0.003 | 0.005 | 0.008 | 0.035 | 0.020 | 0.085 | 0.005 | 0.008 | 0.000 | 0.011 | 0.003 | 0.024 | 0.005 | 0.000 | 0.000 | 0.000 | 0.000 | 0.003 | 0.000 | 0.010 | 0.927 | 0.848 |
| Marcelino Ramos | 113 | 0.058 | 0.066 | 0.049 | 0.027 | 0.106 | 0.053 | 0.022 | 0.106 | 0.018 | 0.018 | 0.049 | 0.066 | 0.009 | 0.004 | 0.133 | 0.000 | 0.000 | 0.000 | 0.004 | 0.013 | 0.013 | 0.071 | 0.009 | 0.022 | 0.000 | 0.018 | 0.004 | 0.040 | 0.018 | 0.000 | 0.000 | 0.004 | 0.000 | 0.000 | 0.000 | 0.000 | 0.930 | 1 |
| Monte Alegre Dos Campos | 75 | 0.040 | 0.133 | 0.027 | 0.013 | 0.080 | 0.053 | 0.020 | 0.093 | 0.000 | 0.033 | 0.040 | 0.073 | 0.007 | 0.007 | 0.100 | 0.000 | 0.000 | 0.007 | 0.000 | 0.007 | 0.033 | 0.133 | 0.000 | 0.000 | 0.000 | 0.020 | 0.000 | 0.033 | 0.047 | 0.000 | 0.000 | 0.000 | 0.000 | 0.000 | 0.000 | 0.000 | 0.921 | 1 |
| Montenegro | 504 | 0.088 | 0.061 | 0.024 | 0.059 | 0.066 | 0.035 | 0.030 | 0.126 | 0.009 | 0.021 | 0.029 | 0.060 | 0.013 | 0.004 | 0.113 | 0.010 | 0.000 | 0.001 | 0.009 | 0.035 | 0.019 | 0.082 | 0.017 | 0.012 | 0.000 | 0.013 | 0.010 | 0.027 | 0.026 | 0.000 | 0.000 | 0.000 | 0.000 | 0.001 | 0.000 | 0.004 | 0.934 | 0.900 |
| Nova Hartz | 1566 | 0.088 | 0.070 | 0.014 | 0.046 | 0.074 | 0.043 | 0.028 | 0.112 | 0.011 | 0.023 | 0.033 | 0.054 | 0.011 | 0.003 | 0.139 | 0.011 | 0.000 | 0.001 | 0.007 | 0.019 | 0.018 | 0.079 | 0.017 | 0.015 | 0.000 | 0.024 | 0.003 | 0.038 | 0.015 | 0.000 | 0.000 | 0.000 | 0.000 | 0.003 | 0.000 | 0.003 | 0.931 | 0.879 |
| Nova Santa Rita | 176 | 0.051 | 0.048 | 0.020 | 0.062 | 0.074 | 0.065 | 0.020 | 0.122 | 0.011 | 0.020 | 0.034 | 0.054 | 0.006 | 0.000 | 0.133 | 0.011 | 0.000 | 0.003 | 0.000 | 0.023 | 0.023 | 0.096 | 0.011 | 0.023 | 0.000 | 0.014 | 0.006 | 0.023 | 0.045 | 0.000 | 0.000 | 0.000 | 0.000 | 0.000 | 0.000 | 0.001 | 0.929 | 1 |
| Novo Hamburgo | 4997 | 0.086 | 0.068 | 0.018 | 0.046 | 0.078 | 0.049 | 0.029 | 0.127 | 0.010 | 0.025 | 0.034 | 0.055 | 0.008 | 0.003 | 0.116 | 0.010 | 0.000 | 0.002 | 0.006 | 0.024 | 0.022 | 0.082 | 0.015 | 0.010 | 0.000 | 0.014 | 0.006 | 0.032 | 0.019 | 0.000 | 0.000 | 0.000 | 0.000 | 0.001 | 0.000 | 0.002 | 0.932 | 0.822 |
| Osorio | 89 | 0.106 | 0.084 | 0.017 | 0.090 | 0.056 | 0.061 | 0.011 | 0.117 | 0.011 | 0.028 | 0.017 | 0.051 | 0.006 | 0.011 | 0.077 | 0.006 | 0.000 | 0.000 | 0.006 | 0.045 | 0.022 | 0.078 | 0.011 | 0.006 | 0.000 | 0.000 | 0.000 | 0.056 | 0.022 | 0.000 | 0.000 | 0.000 | 0.000 | 0.000 | 0.000 | 0.005 | 0.930 | 0.947 |
| **Town** | **N** | **B*07** | **B*08** | **B*13** | **B*14** | **B*15** | **B*18** | **B*27** | **B*35** | **B*37** | **B*38** | **B*39** | **B*40** | **B*41** | **B*42** | **B*44** | **B*45** | **B*46** | **B*47** | **B*48** | **B*49** | **B*50** | **B*51** | **B*52** | **B*53** | **B*54** | **B*55** | **B*56** | **B*57** | **B*58** | **B*59** | **B*67** | **B*73** | **B*78** | **B*81** | **B*82** | **blank** | **Exp H** | **P-HWE** |
| Palmeira Das Missoes | 1005 | 0.069 | 0.067 | 0.021 | 0.035 | 0.080 | 0.065 | 0.035 | 0.115 | 0.015 | 0.025 | 0.039 | 0.048 | 0.013 | 0.002 | 0.109 | 0.005 | 0.000 | 0.001 | 0.006 | 0.025 | 0.011 | 0.106 | 0.017 | 0.009 | 0.000 | 0.018 | 0.007 | 0.034 | 0.013 | 0.000 | 0.000 | 0.001 | 0.000 | 0.000 | 0.000 | 0.008 | 0.933 | 0.720 |
| Panambi | 353 | 0.108 | 0.071 | 0.020 | 0.051 | 0.095 | 0.057 | 0.025 | 0.099 | 0.017 | 0.020 | 0.035 | 0.064 | 0.008 | 0.001 | 0.122 | 0.011 | 0.000 | 0.003 | 0.003 | 0.025 | 0.008 | 0.076 | 0.008 | 0.006 | 0.000 | 0.018 | 0.006 | 0.031 | 0.008 | 0.000 | 0.000 | 0.000 | 0.000 | 0.001 | 0.000 | 0.000 | 0.929 | 1 |
| Parobe | 394 | 0.063 | 0.048 | 0.020 | 0.048 | 0.090 | 0.032 | 0.033 | 0.114 | 0.013 | 0.024 | 0.051 | 0.046 | 0.006 | 0.008 | 0.119 | 0.014 | 0.000 | 0.003 | 0.013 | 0.027 | 0.027 | 0.076 | 0.016 | 0.015 | 0.000 | 0.019 | 0.010 | 0.040 | 0.023 | 0.000 | 0.000 | 0.000 | 0.000 | 0.001 | 0.000 | 0.002 | 0.937 | 0.968 |
| Passo Fundo | 2113 | 0.074 | 0.059 | 0.021 | 0.042 | 0.083 | 0.058 | 0.022 | 0.118 | 0.014 | 0.029 | 0.045 | 0.047 | 0.010 | 0.002 | 0.110 | 0.012 | 0.000 | 0.002 | 0.008 | 0.030 | 0.020 | 0.102 | 0.015 | 0.008 | 0.000 | 0.012 | 0.004 | 0.029 | 0.017 | 0.000 | 0.000 | 0.001 | 0.000 | 0.001 | 0.000 | 0.003 | 0.933 | 0.926 |
| Pelotas | 4143 | 0.077 | 0.060 | 0.015 | 0.059 | 0.081 | 0.046 | 0.030 | 0.115 | 0.010 | 0.020 | 0.027 | 0.050 | 0.012 | 0.005 | 0.130 | 0.015 | 0.000 | 0.002 | 0.006 | 0.027 | 0.026 | 0.082 | 0.011 | 0.014 | 0.000 | 0.011 | 0.004 | 0.032 | 0.022 | 0.000 | 0.000 | 0.001 | 0.001 | 0.001 | 0.000 | 0.005 | 0.932 | 0.645 |
| Portao | 206 | 0.085 | 0.056 | 0.027 | 0.063 | 0.068 | 0.049 | 0.036 | 0.129 | 0.015 | 0.017 | 0.027 | 0.063 | 0.012 | 0.000 | 0.114 | 0.010 | 0.000 | 0.005 | 0.000 | 0.034 | 0.012 | 0.063 | 0.022 | 0.012 | 0.000 | 0.015 | 0.007 | 0.041 | 0.017 | 0.000 | 0.000 | 0.000 | 0.002 | 0.000 | 0.000 | 0.000 | 0.933 | 1 |
| Porto Alegre | 21392 | 0.075 | 0.060 | 0.020 | 0.055 | 0.079 | 0.047 | 0.026 | 0.126 | 0.011 | 0.023 | 0.033 | 0.049 | 0.012 | 0.004 | 0.122 | 0.014 | 0.000 | 0.002 | 0.008 | 0.025 | 0.023 | 0.087 | 0.015 | 0.014 | 0.000 | 0.013 | 0.005 | 0.029 | 0.018 | 0.000 | 0.000 | 0.001 | 0.000 | 0.001 | 0.000 | 0.001 | NC | 0.815 |
| Porto Lucena | 192 | 0.096 | 0.122 | 0.039 | 0.023 | 0.078 | 0.065 | 0.031 | 0.112 | 0.016 | 0.016 | 0.036 | 0.060 | 0.010 | 0.000 | 0.117 | 0.005 | 0.000 | 0.003 | 0.000 | 0.026 | 0.018 | 0.055 | 0.010 | 0.008 | 0.000 | 0.023 | 0.003 | 0.016 | 0.010 | 0.000 | 0.000 | 0.000 | 0.000 | 0.000 | 0.000 | 0.000 | 0.925 | 1 |
| Porto Vera Cruz | 57 | 0.123 | 0.079 | 0.026 | 0.044 | 0.079 | 0.044 | 0.053 | 0.140 | 0.009 | 0.035 | 0.035 | 0.061 | 0.009 | 0.000 | 0.096 | 0.000 | 0.000 | 0.000 | 0.000 | 0.018 | 0.018 | 0.026 | 0.018 | 0.000 | 0.000 | 0.035 | 0.018 | 0.035 | 0.000 | 0.000 | 0.000 | 0.000 | 0.000 | 0.000 | 0.000 | 0.000 | 0.925 | 1 |
| Porto Xavier | 186 | 0.094 | 0.091 | 0.019 | 0.051 | 0.118 | 0.043 | 0.027 | 0.110 | 0.013 | 0.013 | 0.038 | 0.078 | 0.003 | 0.003 | 0.075 | 0.005 | 0.000 | 0.000 | 0.008 | 0.016 | 0.011 | 0.056 | 0.016 | 0.013 | 0.000 | 0.022 | 0.005 | 0.046 | 0.022 | 0.000 | 0.003 | 0.000 | 0.000 | 0.000 | 0.000 | 0.000 | 0.930 | 1 |
| Quinze De Novembro | 52 | 0.096 | 0.115 | 0.010 | 0.010 | 0.077 | 0.086 | 0.019 | 0.096 | 0.000 | 0.019 | 0.019 | 0.019 | 0.000 | 0.000 | 0.201 | 0.010 | 0.000 | 0.000 | 0.000 | 0.029 | 0.010 | 0.029 | 0.019 | 0.010 | 0.000 | 0.038 | 0.037 | 0.029 | 0.019 | 0.000 | 0.000 | 0.000 | 0.000 | 0.000 | 0.000 | 0.003 | 0.906 | 0.996 |
| Redentora | 106 | 0.104 | 0.075 | 0.019 | 0.038 | 0.071 | 0.066 | 0.033 | 0.123 | 0.024 | 0.028 | 0.042 | 0.047 | 0.009 | 0.005 | 0.108 | 0.005 | 0.000 | 0.009 | 0.014 | 0.019 | 0.019 | 0.061 | 0.033 | 0.000 | 0.000 | 0.009 | 0.000 | 0.033 | 0.005 | 0.000 | 0.000 | 0.000 | 0.000 | 0.000 | 0.000 | 0.000 | 0.932 | 1 |
| Rio Grande | 383 | 0.080 | 0.063 | 0.012 | 0.070 | 0.070 | 0.038 | 0.034 | 0.133 | 0.008 | 0.027 | 0.017 | 0.042 | 0.017 | 0.001 | 0.117 | 0.014 | 0.000 | 0.000 | 0.005 | 0.025 | 0.026 | 0.091 | 0.007 | 0.020 | 0.000 | 0.010 | 0.007 | 0.034 | 0.026 | 0.000 | 0.001 | 0.000 | 0.000 | 0.004 | 0.000 | 0.000 | 0.930 | 1 |
| Rio Pardo | 67 | 0.090 | 0.060 | 0.007 | 0.045 | 0.119 | 0.052 | 0.022 | 0.119 | 0.015 | 0.045 | 0.022 | 0.037 | 0.030 | 0.000 | 0.119 | 0.007 | 0.000 | 0.007 | 0.007 | 0.022 | 0.037 | 0.060 | 0.015 | 0.007 | 0.000 | 0.000 | 0.000 | 0.045 | 0.007 | 0.000 | 0.000 | 0.000 | 0.000 | 0.000 | 0.000 | 0.000 | 0.927 | 1 |
| Riozinho | 53 | 0.094 | 0.057 | 0.009 | 0.009 | 0.085 | 0.047 | 0.038 | 0.094 | 0.019 | 0.028 | 0.028 | 0.075 | 0.000 | 0.009 | 0.104 | 0.000 | 0.000 | 0.000 | 0.000 | 0.028 | 0.057 | 0.113 | 0.019 | 0.000 | 0.000 | 0.019 | 0.019 | 0.028 | 0.019 | 0.000 | 0.000 | 0.000 | 0.000 | 0.000 | 0.000 | 0.000 | 0.930 | 1 |
| Rolante | 103 | 0.097 | 0.063 | 0.024 | 0.058 | 0.049 | 0.015 | 0.029 | 0.136 | 0.005 | 0.029 | 0.034 | 0.078 | 0.010 | 0.000 | 0.092 | 0.005 | 0.000 | 0.005 | 0.010 | 0.049 | 0.019 | 0.078 | 0.015 | 0.015 | 0.000 | 0.005 | 0.005 | 0.058 | 0.019 | 0.000 | 0.000 | 0.000 | 0.000 | 0.000 | 0.000 | 0.000 | 0.931 | 1 |
| Rondinha | 99 | 0.049 | 0.051 | 0.015 | 0.025 | 0.040 | 0.091 | 0.029 | 0.222 | 0.000 | 0.030 | 0.020 | 0.030 | 0.020 | 0.010 | 0.091 | 0.000 | 0.000 | 0.000 | 0.000 | 0.030 | 0.005 | 0.166 | 0.010 | 0.000 | 0.000 | 0.015 | 0.020 | 0.010 | 0.015 | 0.000 | 0.000 | 0.000 | 0.000 | 0.000 | 0.000 | 0.003 | 0.894 | 0.990 |
| Salvador Do Sul | 66 | 0.129 | 0.136 | 0.015 | 0.068 | 0.076 | 0.023 | 0.023 | 0.076 | 0.008 | 0.023 | 0.030 | 0.053 | 0.015 | 0.000 | 0.129 | 0.008 | 0.000 | 0.000 | 0.008 | 0.030 | 0.000 | 0.053 | 0.008 | 0.015 | 0.000 | 0.008 | 0.015 | 0.030 | 0.023 | 0.000 | 0.000 | 0.000 | 0.000 | 0.000 | 0.000 | 0.000 | 0.920 | 1 |
| Sananduva | 110 | 0.059 | 0.045 | 0.009 | 0.045 | 0.077 | 0.050 | 0.040 | 0.182 | 0.006 | 0.027 | 0.064 | 0.040 | 0.018 | 0.005 | 0.077 | 0.005 | 0.000 | 0.005 | 0.005 | 0.005 | 0.005 | 0.127 | 0.014 | 0.018 | 0.000 | 0.045 | 0.005 | 0.000 | 0.018 | 0.000 | 0.000 | 0.000 | 0.000 | 0.000 | 0.000 | 0.006 | 0.917 | 0.943 |
| Santa Clara Do Sul | 57 | 0.193 | 0.114 | 0.035 | 0.000 | 0.070 | 0.026 | 0.035 | 0.158 | 0.000 | 0.009 | 0.000 | 0.096 | 0.026 | 0.000 | 0.132 | 0.000 | 0.000 | 0.000 | 0.000 | 0.000 | 0.000 | 0.079 | 0.009 | 0.000 | 0.000 | 0.009 | 0.009 | 0.000 | 0.000 | 0.000 | 0.000 | 0.000 | 0.000 | 0.000 | 0.000 | 0.000 | 0.883 | 1 |
| Santa Cruz Do Sul | 959 | 0.095 | 0.071 | 0.029 | 0.045 | 0.059 | 0.054 | 0.031 | 0.116 | 0.010 | 0.020 | 0.020 | 0.063 | 0.012 | 0.002 | 0.129 | 0.009 | 0.000 | 0.003 | 0.004 | 0.029 | 0.015 | 0.090 | 0.009 | 0.009 | 0.000 | 0.023 | 0.005 | 0.033 | 0.013 | 0.000 | 0.000 | 0.001 | 0.000 | 0.000 | 0.000 | 0.000 | 0.929 | 1 |
| Santa Maria | 1740 | 0.084 | 0.070 | 0.018 | 0.056 | 0.075 | 0.042 | 0.030 | 0.124 | 0.012 | 0.022 | 0.031 | 0.063 | 0.016 | 0.002 | 0.111 | 0.009 | 0.000 | 0.001 | 0.006 | 0.026 | 0.022 | 0.092 | 0.012 | 0.007 | 0.000 | 0.010 | 0.007 | 0.027 | 0.020 | 0.000 | 0.000 | 0.000 | 0.001 | 0.003 | 0.000 | 0.001 | 0.932 | 0.990 |
| Santa Rosa | 1916 | 0.089 | 0.079 | 0.027 | 0.036 | 0.080 | 0.051 | 0.035 | 0.122 | 0.009 | 0.027 | 0.031 | 0.060 | 0.011 | 0.003 | 0.119 | 0.008 | 0.000 | 0.002 | 0.007 | 0.023 | 0.013 | 0.083 | 0.015 | 0.008 | 0.000 | 0.011 | 0.006 | 0.031 | 0.014 | 0.000 | 0.000 | 0.002 | 0.000 | 0.000 | 0.000 | 0.000 | 0.930 | 1 |
| Santana Do Livramento | 500 | 0.070 | 0.050 | 0.013 | 0.070 | 0.091 | 0.042 | 0.025 | 0.103 | 0.009 | 0.029 | 0.049 | 0.052 | 0.010 | 0.002 | 0.110 | 0.027 | 0.000 | 0.001 | 0.008 | 0.023 | 0.027 | 0.067 | 0.021 | 0.023 | 0.000 | 0.019 | 0.001 | 0.020 | 0.033 | 0.000 | 0.000 | 0.000 | 0.000 | 0.005 | 0.000 | 0.000 | 0.939 | 1 |
| Santo Angelo | 468 | 0.085 | 0.075 | 0.016 | 0.038 | 0.077 | 0.051 | 0.024 | 0.136 | 0.010 | 0.027 | 0.043 | 0.053 | 0.009 | 0.002 | 0.115 | 0.012 | 0.000 | 0.001 | 0.004 | 0.020 | 0.007 | 0.094 | 0.016 | 0.011 | 0.000 | 0.011 | 0.003 | 0.030 | 0.016 | 0.000 | 0.001 | 0.002 | 0.001 | 0.000 | 0.000 | 0.010 | 0.928 | 0.766 |
| Santo Antonio Da Patrulha | 337 | 0.104 | 0.067 | 0.012 | 0.049 | 0.080 | 0.055 | 0.027 | 0.126 | 0.016 | 0.028 | 0.016 | 0.028 | 0.013 | 0.001 | 0.119 | 0.015 | 0.000 | 0.003 | 0.001 | 0.021 | 0.025 | 0.082 | 0.013 | 0.009 | 0.000 | 0.013 | 0.009 | 0.043 | 0.024 | 0.000 | 0.000 | 0.000 | 0.000 | 0.000 | 0.000 | 0.000 | 0.929 | 1 |
| Santo Cristo | 144 | 0.162 | 0.063 | 0.044 | 0.052 | 0.065 | 0.044 | 0.027 | 0.103 | 0.007 | 0.017 | 0.038 | 0.056 | 0.007 | 0.000 | 0.100 | 0.000 | 0.000 | 0.000 | 0.003 | 0.017 | 0.014 | 0.083 | 0.017 | 0.003 | 0.000 | 0.007 | 0.003 | 0.042 | 0.017 | 0.000 | 0.000 | 0.000 | 0.000 | 0.000 | 0.000 | 0.006 | 0.923 | 0.922 |
| Sao Jose Do Inhacora | 147 | 0.112 | 0.110 | 0.031 | 0.022 | 0.101 | 0.079 | 0.017 | 0.114 | 0.020 | 0.020 | 0.014 | 0.075 | 0.010 | 0.000 | 0.096 | 0.020 | 0.000 | 0.000 | 0.000 | 0.007 | 0.007 | 0.074 | 0.007 | 0.003 | 0.003 | 0.003 | 0.007 | 0.034 | 0.000 | 0.000 | 0.000 | 0.000 | 0.000 | 0.000 | 0.000 | 0.012 | 0.921 | 0.838 |
| Sao Jose Do Norte | 159 | 0.069 | 0.041 | 0.006 | 0.079 | 0.041 | 0.035 | 0.031 | 0.142 | 0.006 | 0.035 | 0.019 | 0.041 | 0.000 | 0.006 | 0.160 | 0.006 | 0.000 | 0.003 | 0.000 | 0.041 | 0.044 | 0.082 | 0.003 | 0.044 | 0.000 | 0.009 | 0.016 | 0.016 | 0.016 | 0.000 | 0.000 | 0.003 | 0.006 | 0.000 | 0.000 | 0.000 | 0.921 | 1 |
| Sao Leopoldo | 1780 | 0.078 | 0.061 | 0.017 | 0.051 | 0.079 | 0.041 | 0.027 | 0.121 | 0.007 | 0.023 | 0.035 | 0.062 | 0.012 | 0.004 | 0.119 | 0.015 | 0.000 | 0.002 | 0.006 | 0.024 | 0.020 | 0.088 | 0.016 | 0.014 | 0.000 | 0.014 | 0.006 | 0.033 | 0.022 | 0.000 | 0.000 | 0.000 | 0.000 | 0.001 | 0.000 | 0.000 | 0.933 | 1 |
| Sao Luiz Gonzaga | 291 | 0.072 | 0.076 | 0.014 | 0.046 | 0.065 | 0.043 | 0.029 | 0.153 | 0.009 | 0.015 | 0.040 | 0.058 | 0.014 | 0.007 | 0.108 | 0.024 | 0.000 | 0.000 | 0.015 | 0.031 | 0.015 | 0.086 | 0.017 | 0.009 | 0.000 | 0.010 | 0.000 | 0.027 | 0.012 | 0.000 | 0.000 | 0.000 | 0.000 | 0.003 | 0.000 | 0.000 | 0.928 | 1 |
| Sao Paulo Das Missoes | 144 | 0.114 | 0.093 | 0.024 | 0.048 | 0.097 | 0.079 | 0.031 | 0.121 | 0.003 | 0.010 | 0.017 | 0.042 | 0.007 | 0.000 | 0.097 | 0.003 | 0.000 | 0.003 | 0.000 | 0.010 | 0.010 | 0.094 | 0.000 | 0.010 | 0.000 | 0.014 | 0.000 | 0.063 | 0.003 | 0.000 | 0.000 | 0.000 | 0.000 | 0.000 | 0.000 | 0.003 | 0.919 | 0.959 |
| Sao Sebastiao Do Cai | 79 | 0.114 | 0.051 | 0.025 | 0.051 | 0.057 | 0.038 | 0.019 | 0.127 | 0.000 | 0.000 | 0.070 | 0.057 | 0.013 | 0.006 | 0.089 | 0.013 | 0.000 | 0.006 | 0.006 | 0.013 | 0.019 | 0.070 | 0.032 | 0.032 | 0.000 | 0.032 | 0.006 | 0.044 | 0.006 | 0.006 | 0.000 | 0.000 | 0.000 | 0.000 | 0.000 | 0.000 | 0.933 | 1 |
| **Town** | **N** | **B*07** | **B*08** | **B*13** | **B*14** | **B*15** | **B*18** | **B*27** | **B*35** | **B*37** | **B*38** | **B*39** | **B*40** | **B*41** | **B*42** | **B*44** | **B*45** | **B*46** | **B*47** | **B*48** | **B*49** | **B*50** | **B*51** | **B*52** | **B*53** | **B*54** | **B*55** | **B*56** | **B*57** | **B*58** | **B*59** | **B*67** | **B*73** | **B*78** | **B*81** | **B*82** | **blank** | **Exp H** | **P-HWE** |
| Sao Valerio Do Sul | 50 | 0.050 | 0.120 | 0.010 | 0.040 | 0.070 | 0.020 | 0.040 | 0.120 | 0.020 | 0.010 | 0.020 | 0.080 | 0.010 | 0.000 | 0.100 | 0.020 | 0.000 | 0.000 | 0.030 | 0.030 | 0.000 | 0.120 | 0.020 | 0.000 | 0.000 | 0.040 | 0.000 | 0.010 | 0.020 | 0.000 | 0.000 | 0.000 | 0.000 | 0.000 | 0.000 | 0.000 | 0.924 | 1 |
| Sapiranga | 1289 | 0.088 | 0.070 | 0.020 | 0.046 | 0.082 | 0.042 | 0.036 | 0.105 | 0.010 | 0.023 | 0.033 | 0.050 | 0.009 | 0.004 | 0.125 | 0.013 | 0.000 | 0.000 | 0.004 | 0.025 | 0.017 | 0.085 | 0.021 | 0.019 | 0.000 | 0.016 | 0.007 | 0.027 | 0.017 | 0.000 | 0.000 | 0.000 | 0.000 | 0.001 | 0.000 | 0.003 | 0.934 | 0.917 |
| Sapucaia Do Sul | 955 | 0.071 | 0.058 | 0.017 | 0.061 | 0.080 | 0.045 | 0.023 | 0.135 | 0.015 | 0.025 | 0.039 | 0.053 | 0.016 | 0.002 | 0.108 | 0.012 | 0.000 | 0.001 | 0.007 | 0.023 | 0.024 | 0.086 | 0.015 | 0.007 | 0.000 | 0.015 | 0.004 | 0.033 | 0.021 | 0.000 | 0.000 | 0.000 | 0.002 | 0.002 | 0.000 | 0.001 | 0.933 | 0.955 |
| Sarandi | 87 | 0.092 | 0.063 | 0.029 | 0.069 | 0.052 | 0.046 | 0.046 | 0.155 | 0.023 | 0.029 | 0.017 | 0.029 | 0.006 | 0.000 | 0.080 | 0.011 | 0.000 | 0.006 | 0.006 | 0.023 | 0.006 | 0.115 | 0.011 | 0.006 | 0.000 | 0.000 | 0.011 | 0.052 | 0.011 | 0.000 | 0.000 | 0.006 | 0.000 | 0.000 | 0.000 | 0.000 | 0.925 | 1 |
| Sede Nova | 135 | 0.130 | 0.056 | 0.022 | 0.004 | 0.126 | 0.037 | 0.026 | 0.107 | 0.033 | 0.022 | 0.030 | 0.044 | 0.011 | 0.000 | 0.122 | 0.019 | 0.000 | 0.000 | 0.007 | 0.022 | 0.011 | 0.074 | 0.026 | 0.004 | 0.000 | 0.026 | 0.004 | 0.026 | 0.011 | 0.000 | 0.000 | 0.000 | 0.000 | 0.000 | 0.000 | 0.000 | 0.922 | 1 |
| Serafina Correa | 69 | 0.094 | 0.116 | 0.022 | 0.058 | 0.080 | 0.087 | 0.014 | 0.101 | 0.022 | 0.043 | 0.022 | 0.036 | 0.000 | 0.000 | 0.116 | 0.000 | 0.000 | 0.007 | 0.000 | 0.043 | 0.000 | 0.080 | 0.014 | 0.007 | 0.000 | 0.000 | 0.000 | 0.022 | 0.007 | 0.000 | 0.000 | 0.007 | 0.000 | 0.000 | 0.000 | 0.000 | 0.923 | 1 |
| Tapejara | 50 | 0.060 | 0.090 | 0.030 | 0.060 | 0.070 | 0.100 | 0.060 | 0.160 | 0.010 | 0.030 | 0.010 | 0.030 | 0.000 | 0.010 | 0.110 | 0.000 | 0.000 | 0.000 | 0.000 | 0.010 | 0.010 | 0.060 | 0.020 | 0.000 | 0.000 | 0.030 | 0.010 | 0.030 | 0.000 | 0.000 | 0.000 | 0.000 | 0.000 | 0.000 | 0.000 | 0.000 | 0.919 | 1 |
| Taquara | 375 | 0.081 | 0.063 | 0.009 | 0.051 | 0.097 | 0.044 | 0.028 | 0.113 | 0.005 | 0.016 | 0.032 | 0.056 | 0.009 | 0.007 | 0.132 | 0.008 | 0.000 | 0.000 | 0.003 | 0.021 | 0.021 | 0.081 | 0.013 | 0.017 | 0.000 | 0.013 | 0.007 | 0.047 | 0.021 | 0.000 | 0.000 | 0.000 | 0.001 | 0.001 | 0.000 | 0.000 | 0.929 | 1 |
| Taquari | 101 | 0.054 | 0.059 | 0.015 | 0.069 | 0.050 | 0.025 | 0.040 | 0.139 | 0.005 | 0.020 | 0.035 | 0.059 | 0.010 | 0.010 | 0.124 | 0.020 | 0.000 | 0.000 | 0.005 | 0.025 | 0.010 | 0.094 | 0.030 | 0.025 | 0.000 | 0.005 | 0.000 | 0.054 | 0.015 | 0.000 | 0.000 | 0.000 | 0.005 | 0.000 | 0.000 | 0.000 | 0.929 | 1 |
| Tavares | 66 | 0.068 | 0.030 | 0.008 | 0.098 | 0.098 | 0.015 | 0.015 | 0.076 | 0.008 | 0.030 | 0.008 | 0.038 | 0.053 | 0.008 | 0.174 | 0.008 | 0.000 | 0.000 | 0.008 | 0.091 | 0.038 | 0.083 | 0.000 | 0.015 | 0.000 | 0.000 | 0.000 | 0.023 | 0.008 | 0.000 | 0.000 | 0.000 | 0.000 | 0.000 | 0.000 | 0.000 | 0.916 | 1 |
| Tenente Portela | 356 | 0.096 | 0.079 | 0.021 | 0.041 | 0.096 | 0.067 | 0.024 | 0.128 | 0.007 | 0.017 | 0.056 | 0.048 | 0.007 | 0.006 | 0.086 | 0.006 | 0.000 | 0.001 | 0.006 | 0.017 | 0.013 | 0.100 | 0.015 | 0.006 | 0.000 | 0.020 | 0.006 | 0.024 | 0.008 | 0.000 | 0.000 | 0.000 | 0.003 | 0.000 | 0.000 | 0.000 | 0.927 | 1 |
| Torres | 85 | 0.088 | 0.076 | 0.006 | 0.064 | 0.082 | 0.041 | 0.006 | 0.100 | 0.034 | 0.018 | 0.035 | 0.029 | 0.000 | 0.006 | 0.170 | 0.006 | 0.000 | 0.000 | 0.006 | 0.035 | 0.024 | 0.105 | 0.018 | 0.006 | 0.000 | 0.006 | 0.000 | 0.012 | 0.018 | 0.000 | 0.000 | 0.000 | 0.006 | 0.000 | 0.000 | 0.006 | 0.918 | 0.935 |
| Tramandai | 67 | 0.088 | 0.030 | 0.015 | 0.045 | 0.073 | 0.082 | 0.030 | 0.103 | 0.022 | 0.022 | 0.022 | 0.052 | 0.007 | 0.007 | 0.103 | 0.015 | 0.000 | 0.000 | 0.007 | 0.030 | 0.037 | 0.119 | 0.010 | 0.022 | 0.000 | 0.022 | 0.000 | 0.000 | 0.022 | 0.000 | 0.000 | 0.000 | 0.000 | 0.000 | 0.000 | 0.011 | 0.932 | 0.903 |
| Tres Coroas | 56 | 0.141 | 0.071 | 0.009 | 0.045 | 0.063 | 0.018 | 0.009 | 0.097 | 0.009 | 0.027 | 0.054 | 0.036 | 0.018 | 0.009 | 0.152 | 0.000 | 0.000 | 0.000 | 0.000 | 0.027 | 0.000 | 0.071 | 0.009 | 0.018 | 0.000 | 0.018 | 0.013 | 0.063 | 0.018 | 0.000 | 0.000 | 0.000 | 0.000 | 0.000 | 0.000 | 0.008 | 0.920 | 0.936 |
| Tres De Maio | 197 | 0.112 | 0.091 | 0.033 | 0.033 | 0.078 | 0.061 | 0.023 | 0.116 | 0.008 | 0.015 | 0.033 | 0.056 | 0.008 | 0.000 | 0.106 | 0.010 | 0.000 | 0.000 | 0.005 | 0.018 | 0.003 | 0.099 | 0.013 | 0.005 | 0.000 | 0.010 | 0.005 | 0.033 | 0.020 | 0.000 | 0.000 | 0.005 | 0.000 | 0.000 | 0.000 | 0.002 | 0.925 | 1 |
| Tres Passos | 326 | 0.118 | 0.086 | 0.009 | 0.041 | 0.080 | 0.070 | 0.038 | 0.112 | 0.005 | 0.009 | 0.031 | 0.060 | 0.009 | 0.000 | 0.121 | 0.006 | 0.000 | 0.003 | 0.005 | 0.020 | 0.015 | 0.075 | 0.015 | 0.005 | 0.000 | 0.021 | 0.000 | 0.032 | 0.009 | 0.000 | 0.000 | 0.000 | 0.000 | 0.002 | 0.000 | 0.003 | 0.924 | 0.963 |
| Triunfo | 85 | 0.129 | 0.059 | 0.012 | 0.041 | 0.082 | 0.047 | 0.018 | 0.112 | 0.006 | 0.018 | 0.041 | 0.059 | 0.006 | 0.000 | 0.094 | 0.000 | 0.000 | 0.000 | 0.018 | 0.035 | 0.006 | 0.076 | 0.006 | 0.018 | 0.000 | 0.012 | 0.000 | 0.035 | 0.065 | 0.000 | 0.000 | 0.006 | 0.000 | 0.000 | 0.000 | 0.000 | 0.928 | 1 |
| Tupancireta | 50 | 0.120 | 0.070 | 0.010 | 0.050 | 0.110 | 0.050 | 0.010 | 0.140 | 0.010 | 0.020 | 0.040 | 0.070 | 0.010 | 0.000 | 0.110 | 0.020 | 0.000 | 0.010 | 0.010 | 0.010 | 0.010 | 0.070 | 0.020 | 0.000 | 0.000 | 0.000 | 0.000 | 0.000 | 0.030 | 0.000 | 0.000 | 0.000 | 0.000 | 0.000 | 0.000 | 0.000 | 0.918 | 1 |
| Tuparendi | 243 | 0.073 | 0.090 | 0.029 | 0.029 | 0.069 | 0.065 | 0.060 | 0.092 | 0.010 | 0.023 | 0.029 | 0.065 | 0.002 | 0.000 | 0.123 | 0.008 | 0.000 | 0.002 | 0.002 | 0.014 | 0.023 | 0.078 | 0.021 | 0.008 | 0.002 | 0.010 | 0.004 | 0.041 | 0.016 | 0.000 | 0.000 | 0.006 | 0.000 | 0.000 | 0.000 | 0.006 | 0.934 | 0.889 |
| Uruguaiana | 1346 | 0.061 | 0.055 | 0.014 | 0.065 | 0.086 | 0.046 | 0.024 | 0.118 | 0.017 | 0.023 | 0.043 | 0.057 | 0.010 | 0.006 | 0.110 | 0.018 | 0.000 | 0.002 | 0.020 | 0.028 | 0.020 | 0.085 | 0.018 | 0.012 | 0.000 | 0.009 | 0.004 | 0.026 | 0.019 | 0.000 | 0.001 | 0.000 | 0.001 | 0.002 | 0.000 | 0.000 | 0.936 | 1 |
| Vacaria | 614 | 0.067 | 0.053 | 0.029 | 0.042 | 0.080 | 0.065 | 0.017 | 0.132 | 0.013 | 0.024 | 0.036 | 0.047 | 0.008 | 0.002 | 0.098 | 0.015 | 0.000 | 0.007 | 0.007 | 0.022 | 0.014 | 0.103 | 0.021 | 0.015 | 0.000 | 0.015 | 0.007 | 0.041 | 0.012 | 0.000 | 0.000 | 0.001 | 0.000 | 0.001 | 0.000 | 0.004 | 0.933 | 0.905 |
| Venancio Aires | 71 | 0.085 | 0.070 | 0.042 | 0.042 | 0.063 | 0.021 | 0.007 | 0.134 | 0.021 | 0.028 | 0.021 | 0.049 | 0.007 | 0.000 | 0.141 | 0.000 | 0.000 | 0.007 | 0.000 | 0.063 | 0.035 | 0.070 | 0.014 | 0.014 | 0.000 | 0.021 | 0.007 | 0.021 | 0.014 | 0.000 | 0.000 | 0.000 | 0.000 | 0.000 | 0.000 | 0.000 | 0.926 | 1 |
| Vera Cruz | 57 | 0.079 | 0.157 | 0.009 | 0.018 | 0.086 | 0.070 | 0.059 | 0.130 | 0.009 | 0.000 | 0.035 | 0.044 | 0.035 | 0.000 | 0.121 | 0.009 | 0.000 | 0.000 | 0.000 | 0.000 | 0.000 | 0.026 | 0.026 | 0.000 | 0.000 | 0.026 | 0.009 | 0.041 | 0.000 | 0.000 | 0.000 | 0.000 | 0.000 | 0.000 | 0.000 | 0.011 | 0.913 | 0.914 |
| Viamao | 1327 | 0.072 | 0.058 | 0.012 | 0.062 | 0.084 | 0.042 | 0.023 | 0.125 | 0.011 | 0.026 | 0.034 | 0.053 | 0.015 | 0.008 | 0.112 | 0.018 | 0.000 | 0.001 | 0.013 | 0.024 | 0.013 | 0.081 | 0.012 | 0.016 | 0.000 | 0.013 | 0.006 | 0.027 | 0.024 | 0.000 | 0.000 | 0.000 | 0.000 | 0.005 | 0.000 | 0.009 | 0.935 | 0.623 |

**HLA-DRB1**

| **Town** | **N** | **DRB1*01** | **DRB1*03** | **DRB1*04** | **DRB1*07** | **DRB1*08** | **DRB1*09** | **DRB1*10** | **DRB1*11** | **DRB1*12** | **DRB1*13** | **DRB1*14** | **DRB1*15** | **DRB1*16** | **blank** | **Exp H** | **P-HWE** |
| --- | --- | --- | --- | --- | --- | --- | --- | --- | --- | --- | --- | --- | --- | --- | --- | --- | --- |
| Agudo | 99 | 0.081 | 0.106 | 0.152 | 0.162 | 0.040 | 0.010 | 0.005 | 0.167 | 0.035 | 0.106 | 0.051 | 0.051 | 0.035 | 0.000 | 0.885 | 1 |
| Alegrete | 1259 | 0.094 | 0.092 | 0.140 | 0.124 | 0.074 | 0.019 | 0.012 | 0.101 | 0.019 | 0.126 | 0.059 | 0.090 | 0.046 | 0.002 | 0.901 | 0.913 |
| Alvorada | 841 | 0.108 | 0.092 | 0.128 | 0.130 | 0.071 | 0.023 | 0.018 | 0.112 | 0.018 | 0.138 | 0.049 | 0.073 | 0.040 | 0.000 | 0.899 | 1 |
| Ametista Do Sul | 62 | 0.077 | 0.052 | 0.183 | 0.151 | 0.056 | 0.016 | 0.016 | 0.113 | 0.008 | 0.105 | 0.032 | 0.091 | 0.073 | 0.026 | 0.892 | 0.765 |
| Arambare | 60 | 0.083 | 0.111 | 0.050 | 0.120 | 0.097 | 0.008 | 0.000 | 0.092 | 0.033 | 0.241 | 0.042 | 0.054 | 0.042 | 0.027 | 0.880 | 0.759 |
| Aratiba | 130 | 0.092 | 0.104 | 0.131 | 0.177 | 0.038 | 0.012 | 0.015 | 0.112 | 0.008 | 0.146 | 0.027 | 0.108 | 0.031 | 0.000 | 0.883 | 1 |
| Arroio Do Meio | 86 | 0.105 | 0.145 | 0.128 | 0.105 | 0.035 | 0.000 | 0.017 | 0.093 | 0.017 | 0.128 | 0.035 | 0.174 | 0.017 | 0.000 | 0.882 | 1 |
| Arroio Do Sal | 507 | 0.116 | 0.088 | 0.144 | 0.134 | 0.069 | 0.017 | 0.032 | 0.099 | 0.012 | 0.124 | 0.047 | 0.086 | 0.029 | 0.005 | 0.898 | 0.892 |
| Augusto Pestana | 65 | 0.046 | 0.108 | 0.184 | 0.154 | 0.084 | 0.023 | 0.008 | 0.100 | 0.008 | 0.146 | 0.031 | 0.099 | 0.008 | 0.002 | 0.879 | 1 |
| Bento Goncalves | 248 | 0.099 | 0.123 | 0.117 | 0.133 | 0.044 | 0.016 | 0.014 | 0.147 | 0.024 | 0.145 | 0.032 | 0.073 | 0.032 | 0.000 | 0.891 | 1 |
| Boa Vista Do Burica | 173 | 0.115 | 0.099 | 0.171 | 0.124 | 0.029 | 0.000 | 0.003 | 0.119 | 0.009 | 0.106 | 0.035 | 0.133 | 0.029 | 0.029 | 0.885 | 0.569 |
| Cachoeira Do Sul | 58 | 0.164 | 0.034 | 0.189 | 0.129 | 0.069 | 0.009 | 0.026 | 0.095 | 0.034 | 0.112 | 0.026 | 0.103 | 0.009 | 0.000 | 0.880 | 1 |
| Cachoeirinha | 1139 | 0.104 | 0.097 | 0.132 | 0.143 | 0.061 | 0.018 | 0.014 | 0.122 | 0.014 | 0.136 | 0.039 | 0.083 | 0.040 | 0.000 | 0.895 | 1 |
| Caibate | 88 | 0.153 | 0.091 | 0.176 | 0.091 | 0.023 | 0.034 | 0.017 | 0.097 | 0.011 | 0.131 | 0.017 | 0.091 | 0.068 | 0.000 | 0.887 | 1 |
| Camaqua | 114 | 0.083 | 0.127 | 0.132 | 0.123 | 0.075 | 0.022 | 0.004 | 0.096 | 0.044 | 0.149 | 0.018 | 0.088 | 0.039 | 0.000 | 0.895 | 1 |
| Campestre Da Serra | 92 | 0.109 | 0.087 | 0.114 | 0.092 | 0.016 | 0.005 | 0.033 | 0.196 | 0.022 | 0.174 | 0.049 | 0.087 | 0.016 | 0.000 | 0.878 | 1 |
| Campina Das Missoes | 78 | 0.147 | 0.122 | 0.109 | 0.154 | 0.038 | 0.006 | 0.013 | 0.115 | 0.026 | 0.109 | 0.019 | 0.135 | 0.006 | 0.000 | 0.882 | 1 |
| Campo Bom | 612 | 0.109 | 0.097 | 0.134 | 0.141 | 0.059 | 0.016 | 0.020 | 0.138 | 0.016 | 0.115 | 0.035 | 0.089 | 0.027 | 0.004 | 0.894 | 0.885 |
| Campo Novo | 177 | 0.097 | 0.094 | 0.132 | 0.119 | 0.038 | 0.006 | 0.006 | 0.123 | 0.000 | 0.115 | 0.064 | 0.139 | 0.053 | 0.014 | 0.894 | 0.782 |
| Candido Godoi | 172 | 0.105 | 0.093 | 0.145 | 0.122 | 0.029 | 0.003 | 0.006 | 0.134 | 0.020 | 0.140 | 0.026 | 0.148 | 0.029 | 0.000 | 0.882 | 1 |
| Canela | 51 | 0.108 | 0.118 | 0.157 | 0.127 | 0.039 | 0.000 | 0.020 | 0.088 | 0.010 | 0.196 | 0.059 | 0.049 | 0.029 | 0.000 | 0.879 | 1 |
| Canoas | 3820 | 0.102 | 0.102 | 0.132 | 0.130 | 0.054 | 0.016 | 0.012 | 0.119 | 0.018 | 0.135 | 0.045 | 0.093 | 0.040 | 0.000 | 0.896 | 1 |
| Capao Da Canoa | 104 | 0.115 | 0.091 | 0.106 | 0.110 | 0.063 | 0.019 | 0.019 | 0.167 | 0.010 | 0.148 | 0.033 | 0.077 | 0.038 | 0.005 | 0.892 | 0.951 |
| Carazinho | 314 | 0.107 | 0.116 | 0.113 | 0.132 | 0.053 | 0.013 | 0.016 | 0.158 | 0.013 | 0.108 | 0.053 | 0.089 | 0.030 | 0.000 | 0.893 | 1 |
| Catuipe | 52 | 0.106 | 0.144 | 0.096 | 0.096 | 0.067 | 0.029 | 0.000 | 0.115 | 0.019 | 0.183 | 0.048 | 0.067 | 0.029 | 0.000 | 0.889 | 1 |
| Caxias Do Sul | 4959 | 0.096 | 0.097 | 0.121 | 0.128 | 0.059 | 0.012 | 0.017 | 0.151 | 0.015 | 0.127 | 0.050 | 0.085 | 0.040 | 0.001 | NC | 0.935 |
| Cerro Largo | 59 | 0.144 | 0.110 | 0.102 | 0.119 | 0.085 | 0.017 | 0.008 | 0.102 | 0.017 | 0.110 | 0.000 | 0.153 | 0.034 | 0.000 | 0.888 | 1 |
| Charqueadas | 107 | 0.121 | 0.107 | 0.136 | 0.112 | 0.042 | 0.014 | 0.023 | 0.117 | 0.009 | 0.145 | 0.075 | 0.084 | 0.014 | 0.000 | 0.893 | 1 |
| Crissiumal | 277 | 0.108 | 0.087 | 0.137 | 0.121 | 0.036 | 0.011 | 0.014 | 0.134 | 0.020 | 0.155 | 0.036 | 0.108 | 0.032 | 0.000 | 0.889 | 1 |
| Cruz Alta | 1977 | 0.107 | 0.092 | 0.118 | 0.137 | 0.070 | 0.020 | 0.016 | 0.121 | 0.014 | 0.130 | 0.047 | 0.092 | 0.037 | 0.000 | 0.898 | 1 |
| **Town** | **N** | **DRB1*01** | **DRB1*03** | **DRB1*04** | **DRB1*07** | **DRB1*08** | **DRB1*09** | **DRB1*10** | **DRB1*11** | **DRB1*12** | **DRB1*13** | **DRB1*14** | **DRB1*15** | **DRB1*16** | **blank** | **Exp H** | **P-HWE** |
| Cruzeiro Do Sul | 79 | 0.130 | 0.070 | 0.161 | 0.117 | 0.054 | 0.013 | 0.019 | 0.130 | 0.006 | 0.091 | 0.025 | 0.139 | 0.025 | 0.018 | 0.889 | 0.814 |
| Dois Irmaos | 163 | 0.077 | 0.129 | 0.123 | 0.156 | 0.046 | 0.009 | 0.009 | 0.117 | 0.006 | 0.123 | 0.052 | 0.129 | 0.025 | 0.000 | 0.887 | 1 |
| Doutor Mauricio Cardoso | 172 | 0.106 | 0.081 | 0.112 | 0.162 | 0.035 | 0.003 | 0.009 | 0.161 | 0.006 | 0.123 | 0.039 | 0.124 | 0.026 | 0.014 | 0.883 | 0.788 |
| Eldorado Do Sul | 175 | 0.091 | 0.109 | 0.151 | 0.129 | 0.077 | 0.029 | 0.020 | 0.083 | 0.026 | 0.120 | 0.049 | 0.097 | 0.020 | 0.000 | 0.899 | 1 |
| Encantado | 214 | 0.091 | 0.117 | 0.093 | 0.126 | 0.028 | 0.016 | 0.028 | 0.196 | 0.019 | 0.110 | 0.047 | 0.077 | 0.051 | 0.000 | 0.890 | 1 |
| Erechim | 663 | 0.115 | 0.091 | 0.110 | 0.133 | 0.044 | 0.016 | 0.013 | 0.167 | 0.014 | 0.118 | 0.037 | 0.094 | 0.048 | 0.000 | 0.892 | 1 |
| Espumoso | 107 | 0.097 | 0.088 | 0.120 | 0.101 | 0.037 | 0.014 | 0.011 | 0.120 | 0.005 | 0.185 | 0.065 | 0.089 | 0.056 | 0.011 | 0.892 | 0.875 |
| Estancia Velha | 883 | 0.097 | 0.098 | 0.125 | 0.146 | 0.054 | 0.018 | 0.016 | 0.116 | 0.012 | 0.129 | 0.037 | 0.113 | 0.037 | 0.003 | 0.895 | 0.927 |
| Esteio | 654 | 0.113 | 0.094 | 0.122 | 0.139 | 0.062 | 0.021 | 0.011 | 0.103 | 0.022 | 0.149 | 0.042 | 0.076 | 0.044 | 0.000 | 0.897 | 1 |
| Estrela | 799 | 0.122 | 0.086 | 0.128 | 0.138 | 0.050 | 0.014 | 0.014 | 0.109 | 0.013 | 0.135 | 0.039 | 0.123 | 0.027 | 0.002 | 0.892 | 0.959 |
| Farroupilha | 2347 | 0.095 | 0.095 | 0.112 | 0.128 | 0.048 | 0.010 | 0.016 | 0.177 | 0.013 | 0.130 | 0.043 | 0.092 | 0.042 | 0.000 | 0.890 | 1 |
| Flores Da Cunha | 56 | 0.063 | 0.080 | 0.134 | 0.134 | 0.045 | 0.018 | 0.009 | 0.134 | 0.036 | 0.125 | 0.071 | 0.071 | 0.080 | 0.000 | 0.900 | 1 |
| Garibaldi | 65 | 0.123 | 0.138 | 0.100 | 0.131 | 0.023 | 0.008 | 0.000 | 0.108 | 0.015 | 0.085 | 0.069 | 0.108 | 0.092 | 0.000 | 0.894 | 1 |
| Getulio Vargas | 90 | 0.050 | 0.072 | 0.139 | 0.122 | 0.044 | 0.028 | 0.000 | 0.200 | 0.017 | 0.111 | 0.072 | 0.111 | 0.033 | 0.000 | 0.884 | 1 |
| Gravatai | 3217 | 0.105 | 0.094 | 0.123 | 0.138 | 0.057 | 0.025 | 0.015 | 0.107 | 0.020 | 0.141 | 0.049 | 0.093 | 0.033 | 0.000 | 0.898 | 1 |
| Guaiba | 469 | 0.107 | 0.095 | 0.126 | 0.136 | 0.076 | 0.019 | 0.013 | 0.105 | 0.023 | 0.129 | 0.044 | 0.084 | 0.039 | 0.004 | 0.900 | 0.897 |
| Humaita | 58 | 0.095 | 0.078 | 0.192 | 0.065 | 0.060 | 0.000 | 0.017 | 0.161 | 0.029 | 0.069 | 0.052 | 0.100 | 0.052 | 0.031 | 0.892 | 0.732 |
| Ibiruba | 93 | 0.086 | 0.113 | 0.156 | 0.177 | 0.081 | 0.000 | 0.011 | 0.075 | 0.022 | 0.124 | 0.038 | 0.091 | 0.027 | 0.000 | 0.886 | 1 |
| Igrejinha | 279 | 0.098 | 0.096 | 0.150 | 0.123 | 0.057 | 0.013 | 0.016 | 0.121 | 0.011 | 0.124 | 0.041 | 0.119 | 0.025 | 0.005 | 0.893 | 0.895 |
| Ijui | 561 | 0.100 | 0.108 | 0.107 | 0.135 | 0.057 | 0.015 | 0.009 | 0.135 | 0.019 | 0.122 | 0.045 | 0.104 | 0.045 | 0.000 | 0.897 | 1 |
| Itaqui | 142 | 0.074 | 0.077 | 0.218 | 0.099 | 0.039 | 0.011 | 0.018 | 0.116 | 0.011 | 0.162 | 0.028 | 0.109 | 0.039 | 0.000 | 0.875 | 1 |
| Ivoti | 325 | 0.106 | 0.115 | 0.138 | 0.118 | 0.037 | 0.011 | 0.012 | 0.127 | 0.016 | 0.136 | 0.029 | 0.118 | 0.032 | 0.004 | 0.890 | 0.918 |
| Jacutinga | 154 | 0.146 | 0.084 | 0.088 | 0.107 | 0.036 | 0.013 | 0.013 | 0.185 | 0.013 | 0.107 | 0.049 | 0.094 | 0.065 | 0.000 | 0.889 | 1 |
| Lagoa Vermelha | 77 | 0.123 | 0.097 | 0.078 | 0.097 | 0.084 | 0.013 | 0.013 | 0.175 | 0.000 | 0.123 | 0.071 | 0.097 | 0.026 | 0.000 | 0.891 | 1 |
| Lajeado | 1484 | 0.102 | 0.101 | 0.130 | 0.127 | 0.047 | 0.011 | 0.008 | 0.133 | 0.017 | 0.130 | 0.041 | 0.116 | 0.028 | 0.009 | 0.893 | 0.629 |
| Marau | 188 | 0.125 | 0.112 | 0.120 | 0.117 | 0.053 | 0.011 | 0.011 | 0.170 | 0.013 | 0.120 | 0.048 | 0.061 | 0.040 | 0.000 | 0.890 | 1 |
| Marcelino Ramos | 113 | 0.079 | 0.110 | 0.119 | 0.114 | 0.061 | 0.009 | 0.022 | 0.190 | 0.013 | 0.137 | 0.031 | 0.084 | 0.027 | 0.005 | 0.887 | 0.953 |
| Monte Alegre Dos Campos | 75 | 0.112 | 0.120 | 0.133 | 0.159 | 0.066 | 0.000 | 0.007 | 0.146 | 0.020 | 0.067 | 0.027 | 0.093 | 0.047 | 0.004 | 0.888 | 0.990 |
| Montenegro | 504 | 0.106 | 0.093 | 0.144 | 0.160 | 0.050 | 0.021 | 0.010 | 0.117 | 0.017 | 0.116 | 0.031 | 0.109 | 0.027 | 0.000 | 0.890 | 1 |
| Nova Hartz | 1566 | 0.105 | 0.102 | 0.142 | 0.142 | 0.043 | 0.020 | 0.014 | 0.115 | 0.019 | 0.114 | 0.042 | 0.107 | 0.034 | 0.000 | 0.895 | 1 |
| Nova Santa Rita | 176 | 0.084 | 0.100 | 0.106 | 0.158 | 0.051 | 0.017 | 0.017 | 0.114 | 0.017 | 0.135 | 0.051 | 0.106 | 0.031 | 0.011 | 0.897 | 0.826 |
| Novo Hamburgo | 4997 | 0.099 | 0.101 | 0.135 | 0.128 | 0.050 | 0.015 | 0.012 | 0.119 | 0.020 | 0.136 | 0.046 | 0.100 | 0.036 | 0.002 | NC | 0.831 |
| Osorio | 89 | 0.152 | 0.112 | 0.090 | 0.124 | 0.039 | 0.022 | 0.006 | 0.118 | 0.045 | 0.101 | 0.062 | 0.096 | 0.034 | 0.000 | 0.899 | 1 |
| **Town** | **N** | **DRB1*01** | **DRB1*03** | **DRB1*04** | **DRB1*07** | **DRB1*08** | **DRB1*09** | **DRB1*10** | **DRB1*11** | **DRB1*12** | **DRB1*13** | **DRB1*14** | **DRB1*15** | **DRB1*16** | **blank** | **Exp H** | **P-HWE** |
| Palmeira Das Missoes | 1005 | 0.107 | 0.095 | 0.114 | 0.117 | 0.048 | 0.012 | 0.012 | 0.173 | 0.016 | 0.140 | 0.031 | 0.098 | 0.037 | 0.000 | 0.889 | 1 |
| Panambi | 353 | 0.091 | 0.115 | 0.139 | 0.108 | 0.059 | 0.014 | 0.013 | 0.124 | 0.018 | 0.138 | 0.034 | 0.122 | 0.018 | 0.006 | 0.892 | 0.873 |
| Parobe | 394 | 0.092 | 0.081 | 0.116 | 0.139 | 0.086 | 0.030 | 0.015 | 0.119 | 0.018 | 0.137 | 0.049 | 0.086 | 0.028 | 0.004 | 0.900 | 0.912 |
| Passo Fundo | 2113 | 0.090 | 0.091 | 0.124 | 0.127 | 0.057 | 0.013 | 0.019 | 0.147 | 0.012 | 0.142 | 0.053 | 0.088 | 0.037 | 0.000 | 0.894 | 1 |
| Pelotas | 4143 | 0.108 | 0.103 | 0.133 | 0.138 | 0.053 | 0.017 | 0.018 | 0.113 | 0.015 | 0.141 | 0.040 | 0.090 | 0.031 | 0.003 | NC | 0.815 |
| Portao | 206 | 0.125 | 0.079 | 0.108 | 0.154 | 0.054 | 0.017 | 0.012 | 0.127 | 0.005 | 0.098 | 0.044 | 0.102 | 0.044 | 0.029 | 0.898 | 0.532 |
| Porto Alegre | 21392 | 0.105 | 0.094 | 0.131 | 0.141 | 0.055 | 0.017 | 0.015 | 0.121 | 0.017 | 0.132 | 0.045 | 0.091 | 0.036 | 0.001 | NC | 0.868 |
| Porto Lucena | 192 | 0.099 | 0.104 | 0.138 | 0.133 | 0.049 | 0.005 | 0.026 | 0.151 | 0.008 | 0.086 | 0.042 | 0.122 | 0.036 | 0.000 | 0.891 | 1 |
| Porto Vera Cruz | 57 | 0.140 | 0.079 | 0.132 | 0.079 | 0.035 | 0.009 | 0.000 | 0.123 | 0.035 | 0.202 | 0.009 | 0.132 | 0.026 | 0.000 | 0.874 | 1 |
| Porto Xavier | 186 | 0.065 | 0.113 | 0.167 | 0.113 | 0.051 | 0.011 | 0.011 | 0.097 | 0.005 | 0.167 | 0.065 | 0.105 | 0.032 | 0.000 | 0.886 | 1 |
| Quinze De Novembro | 52 | 0.067 | 0.125 | 0.115 | 0.144 | 0.048 | 0.000 | 0.000 | 0.087 | 0.010 | 0.125 | 0.067 | 0.202 | 0.010 | 0.000 | 0.875 | 1 |
| Redentora | 106 | 0.090 | 0.113 | 0.094 | 0.127 | 0.057 | 0.033 | 0.009 | 0.175 | 0.014 | 0.094 | 0.066 | 0.094 | 0.033 | 0.000 | 0.896 | 1 |
| Rio Grande | 383 | 0.097 | 0.103 | 0.098 | 0.144 | 0.057 | 0.012 | 0.017 | 0.125 | 0.020 | 0.146 | 0.045 | 0.088 | 0.038 | 0.009 | 0.897 | 0.800 |
| Rio Pardo | 67 | 0.075 | 0.060 | 0.142 | 0.090 | 0.045 | 0.007 | 0.007 | 0.149 | 0.022 | 0.201 | 0.060 | 0.082 | 0.060 | 0.000 | 0.883 | 1 |
| Riozinho | 53 | 0.094 | 0.094 | 0.151 | 0.132 | 0.085 | 0.000 | 0.019 | 0.151 | 0.019 | 0.075 | 0.047 | 0.085 | 0.047 | 0.000 | 0.894 | 1 |
| Rolante | 103 | 0.102 | 0.078 | 0.136 | 0.155 | 0.063 | 0.024 | 0.019 | 0.097 | 0.015 | 0.117 | 0.053 | 0.117 | 0.024 | 0.000 | 0.896 | 1 |
| Rondinha | 99 | 0.126 | 0.086 | 0.076 | 0.071 | 0.075 | 0.005 | 0.015 | 0.287 | 0.010 | 0.141 | 0.035 | 0.035 | 0.035 | 0.002 | 0.854 | 1 |
| Salvador Do Sul | 66 | 0.151 | 0.181 | 0.083 | 0.159 | 0.061 | 0.008 | 0.015 | 0.061 | 0.000 | 0.129 | 0.008 | 0.106 | 0.038 | 0.001 | 0.875 | 1 |
| Sananduva | 110 | 0.100 | 0.091 | 0.086 | 0.091 | 0.050 | 0.005 | 0.032 | 0.214 | 0.009 | 0.132 | 0.082 | 0.073 | 0.036 | 0.000 | 0.886 | 1 |
| Santa Clara Do Sul | 57 | 0.132 | 0.088 | 0.167 | 0.079 | 0.009 | 0.026 | 0.000 | 0.193 | 0.018 | 0.105 | 0.035 | 0.132 | 0.018 | 0.000 | 0.873 | 1 |
| Santa Cruz Do Sul | 959 | 0.105 | 0.096 | 0.136 | 0.125 | 0.052 | 0.010 | 0.011 | 0.113 | 0.023 | 0.138 | 0.033 | 0.118 | 0.035 | 0.003 | 0.894 | 0.900 |
| Santa Maria | 1740 | 0.104 | 0.097 | 0.125 | 0.133 | 0.063 | 0.014 | 0.016 | 0.132 | 0.015 | 0.132 | 0.047 | 0.084 | 0.037 | 0.000 | 0.896 | 1 |
| Santa Rosa | 1916 | 0.095 | 0.097 | 0.138 | 0.126 | 0.052 | 0.013 | 0.013 | 0.134 | 0.013 | 0.131 | 0.043 | 0.104 | 0.041 | 0.000 | 0.894 | 1 |
| Santana Do Livramento | 500 | 0.105 | 0.075 | 0.162 | 0.117 | 0.067 | 0.018 | 0.023 | 0.093 | 0.017 | 0.139 | 0.060 | 0.081 | 0.040 | 0.004 | 0.898 | 0.904 |
| Santo Angelo | 468 | 0.092 | 0.109 | 0.124 | 0.108 | 0.062 | 0.020 | 0.010 | 0.127 | 0.016 | 0.136 | 0.047 | 0.091 | 0.048 | 0.011 | 0.901 | 0.735 |
| Santo Antonio Da Patrulha | 337 | 0.105 | 0.107 | 0.105 | 0.138 | 0.031 | 0.037 | 0.015 | 0.132 | 0.015 | 0.144 | 0.031 | 0.119 | 0.021 | 0.000 | 0.891 | 1 |
| Santo Cristo | 144 | 0.108 | 0.076 | 0.132 | 0.174 | 0.045 | 0.007 | 0.000 | 0.101 | 0.035 | 0.118 | 0.028 | 0.153 | 0.024 | 0.001 | 0.883 | 1 |
| Sao Jose Do Inhacora | 147 | 0.095 | 0.121 | 0.136 | 0.135 | 0.067 | 0.000 | 0.000 | 0.118 | 0.020 | 0.121 | 0.007 | 0.137 | 0.031 | 0.012 | 0.886 | 0.829 |
| Sao Jose Do Norte | 159 | 0.135 | 0.063 | 0.123 | 0.157 | 0.053 | 0.003 | 0.003 | 0.123 | 0.013 | 0.204 | 0.044 | 0.053 | 0.025 | 0.000 | 0.873 | 1 |
| Sao Leopoldo | 1780 | 0.105 | 0.082 | 0.128 | 0.143 | 0.056 | 0.016 | 0.015 | 0.127 | 0.023 | 0.142 | 0.044 | 0.088 | 0.031 | 0.000 | 0.894 | 1 |
| Sao Luiz Gonzaga | 291 | 0.105 | 0.103 | 0.137 | 0.137 | 0.052 | 0.018 | 0.018 | 0.109 | 0.024 | 0.120 | 0.039 | 0.086 | 0.048 | 0.003 | 0.899 | 0.952 |
| Sao Paulo Das Missoes | 144 | 0.076 | 0.122 | 0.177 | 0.118 | 0.031 | 0.010 | 0.000 | 0.128 | 0.024 | 0.118 | 0.024 | 0.132 | 0.038 | 0.000 | 0.883 | 1 |
| Sao Sebastiao Do Cai | 79 | 0.114 | 0.070 | 0.146 | 0.146 | 0.070 | 0.006 | 0.000 | 0.101 | 0.032 | 0.133 | 0.044 | 0.120 | 0.019 | 0.000 | 0.889 | 1 |
| **Town** | **N** | **DRB1*01** | **DRB1*03** | **DRB1*04** | **DRB1*07** | **DRB1*08** | **DRB1*09** | **DRB1*10** | **DRB1*11** | **DRB1*12** | **DRB1*13** | **DRB1*14** | **DRB1*15** | **DRB1*16** | **blank** | **Exp H** | **P-HWE** |
| Sao Valerio Do Sul | 50 | 0.070 | 0.150 | 0.110 | 0.080 | 0.060 | 0.020 | 0.010 | 0.210 | 0.010 | 0.080 | 0.070 | 0.090 | 0.040 | 0.000 | 0.778 | 1 |
| Sapiranga | 1289 | 0.100 | 0.099 | 0.131 | 0.133 | 0.069 | 0.017 | 0.018 | 0.099 | 0.013 | 0.134 | 0.049 | 0.101 | 0.037 | 0.000 | 0.898 | 1 |
| Sapucaia Do Sul | 955 | 0.101 | 0.088 | 0.134 | 0.120 | 0.058 | 0.017 | 0.017 | 0.111 | 0.014 | 0.147 | 0.052 | 0.091 | 0.045 | 0.003 | 0.898 | 0.959 |
| Sarandi | 87 | 0.125 | 0.063 | 0.096 | 0.117 | 0.052 | 0.011 | 0.017 | 0.167 | 0.011 | 0.154 | 0.017 | 0.084 | 0.061 | 0.023 | 0.891 | 0.749 |
| Sede Nova | 135 | 0.096 | 0.099 | 0.155 | 0.096 | 0.048 | 0.007 | 0.007 | 0.125 | 0.017 | 0.111 | 0.037 | 0.137 | 0.059 | 0.005 | 0.893 | 0.939 |
| Serafina Correa | 69 | 0.094 | 0.123 | 0.094 | 0.130 | 0.051 | 0.007 | 0.029 | 0.181 | 0.014 | 0.109 | 0.022 | 0.094 | 0.051 | 0.000 | 0.890 | 1 |
| Tapejara | 50 | 0.130 | 0.160 | 0.110 | 0.130 | 0.060 | 0.000 | 0.000 | 0.270 | 0.010 | 0.060 | 0.010 | 0.030 | 0.030 | 0.000 | 0.846 | 1 |
| Taquara | 375 | 0.083 | 0.087 | 0.124 | 0.133 | 0.056 | 0.011 | 0.013 | 0.113 | 0.024 | 0.151 | 0.047 | 0.121 | 0.037 | 0.000 | 0.895 | 1 |
| Taquari | 101 | 0.099 | 0.084 | 0.173 | 0.178 | 0.050 | 0.030 | 0.015 | 0.124 | 0.010 | 0.114 | 0.015 | 0.079 | 0.030 | 0.000 | 0.882 | 1 |
| Tavares | 66 | 0.091 | 0.068 | 0.106 | 0.174 | 0.061 | 0.000 | 0.030 | 0.038 | 0.008 | 0.182 | 0.023 | 0.174 | 0.045 | 0.000 | 0.873 | 1 |
| Tenente Portela | 356 | 0.105 | 0.108 | 0.102 | 0.114 | 0.052 | 0.017 | 0.010 | 0.106 | 0.015 | 0.118 | 0.073 | 0.106 | 0.061 | 0.014 | 0.905 | 0.705 |
| Torres | 85 | 0.071 | 0.098 | 0.136 | 0.136 | 0.035 | 0.012 | 0.018 | 0.108 | 0.012 | 0.221 | 0.035 | 0.080 | 0.018 | 0.021 | 0.878 | 0.774 |
| Tramandai | 67 | 0.148 | 0.060 | 0.116 | 0.141 | 0.075 | 0.007 | 0.007 | 0.073 | 0.030 | 0.125 | 0.037 | 0.118 | 0.052 | 0.009 | 0.895 | 0.918 |
| Tres Coroas | 56 | 0.125 | 0.125 | 0.098 | 0.143 | 0.036 | 0.027 | 0.000 | 0.071 | 0.009 | 0.134 | 0.054 | 0.125 | 0.054 | 0.000 | 0.892 | 1 |
| Tres De Maio | 197 | 0.091 | 0.112 | 0.122 | 0.140 | 0.048 | 0.015 | 0.003 | 0.119 | 0.005 | 0.117 | 0.036 | 0.145 | 0.048 | 0.000 | 0.890 | 1 |
| Tres Passos | 326 | 0.089 | 0.101 | 0.155 | 0.126 | 0.051 | 0.023 | 0.009 | 0.117 | 0.014 | 0.115 | 0.043 | 0.132 | 0.026 | 0.000 | 0.892 | 1 |
| Triunfo | 85 | 0.085 | 0.088 | 0.145 | 0.110 | 0.047 | 0.041 | 0.006 | 0.150 | 0.000 | 0.094 | 0.051 | 0.142 | 0.024 | 0.018 | 0.893 | 0.812 |
| Tupancireta | 50 | 0.060 | 0.100 | 0.120 | 0.110 | 0.050 | 0.030 | 0.010 | 0.110 | 0.010 | 0.160 | 0.060 | 0.100 | 0.080 | 0.000 | 0.899 | 1 |
| Tuparendi | 243 | 0.086 | 0.113 | 0.101 | 0.126 | 0.064 | 0.002 | 0.012 | 0.128 | 0.006 | 0.134 | 0.058 | 0.126 | 0.045 | 0.000 | 0.894 | 1 |
| Uruguaiana | 1346 | 0.107 | 0.092 | 0.158 | 0.123 | 0.076 | 0.025 | 0.016 | 0.113 | 0.010 | 0.116 | 0.052 | 0.072 | 0.040 | 0.000 | 0.897 | 1 |
| Vacaria | 614 | 0.115 | 0.084 | 0.110 | 0.157 | 0.077 | 0.013 | 0.015 | 0.141 | 0.015 | 0.110 | 0.069 | 0.064 | 0.031 | 0.000 | 0.895 | 1 |
| Venancio Aires | 71 | 0.113 | 0.099 | 0.176 | 0.127 | 0.049 | 0.000 | 0.007 | 0.113 | 0.007 | 0.141 | 0.021 | 0.127 | 0.021 | 0.000 | 0.878 | 1 |
| Vera Cruz | 57 | 0.123 | 0.132 | 0.079 | 0.096 | 0.026 | 0.018 | 0.009 | 0.149 | 0.035 | 0.149 | 0.053 | 0.105 | 0.026 | 0.000 | 0.891 | 1 |
| Viamao | 1327 | 0.108 | 0.095 | 0.133 | 0.133 | 0.054 | 0.023 | 0.018 | 0.120 | 0.015 | 0.133 | 0.044 | 0.086 | 0.038 | 0.000 | 0.897 | 1 |
