# Supplementary material for: Spatial analyzes of HLA data in Rio Grande do Sul, south Brazil: genetic structure and possible correlation with autoimmune diseases
Source: Int J Health Geogr. 2018 Sep 14;17:34. doi: 10.1186/s12942-018-0154-8 (PMC6137739; doi:10.1186/s12942-018-0154-8)
Supplement: Supplementary file 3 — Additional file 3. Allelic frequency maps. [file 12942_2018_154_MOESM3_ESM.docx]

**Additional file 3 – Allelic frequency maps**

**HLA-A**


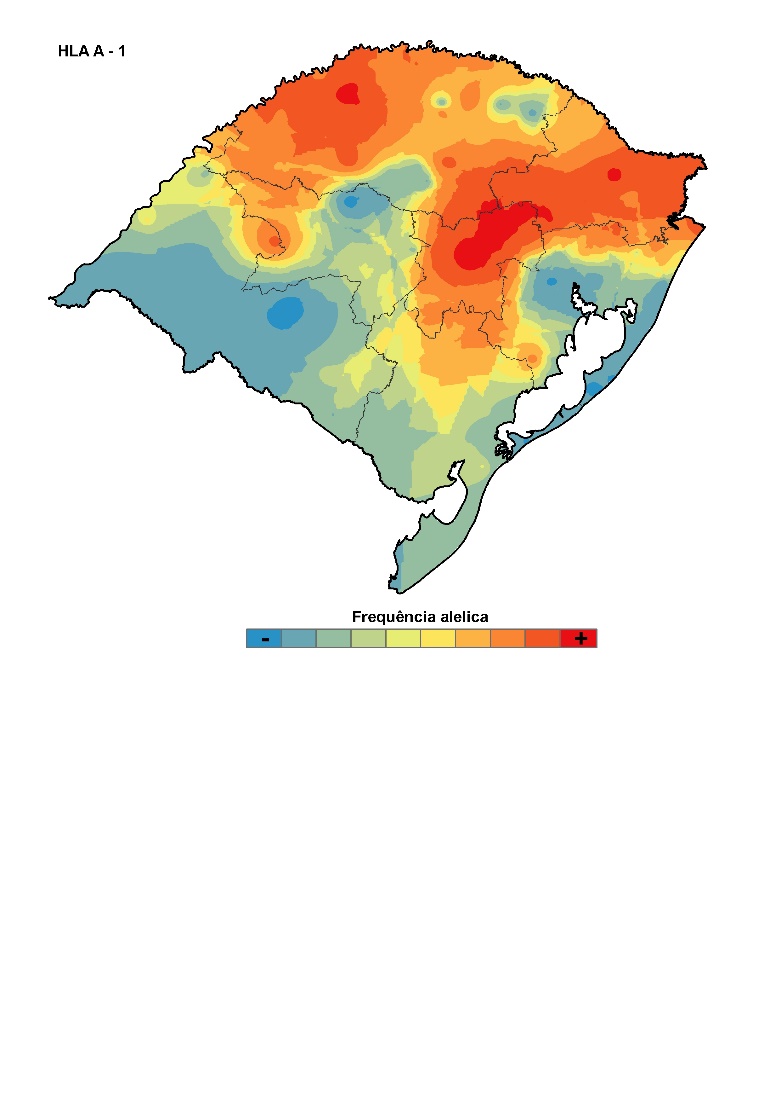


HLA-A*01


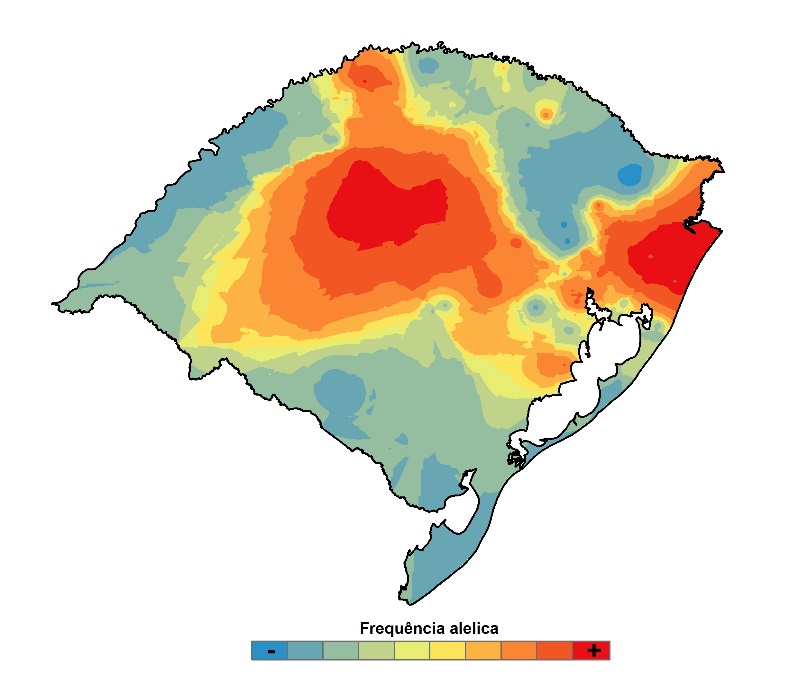


HLA-A*02


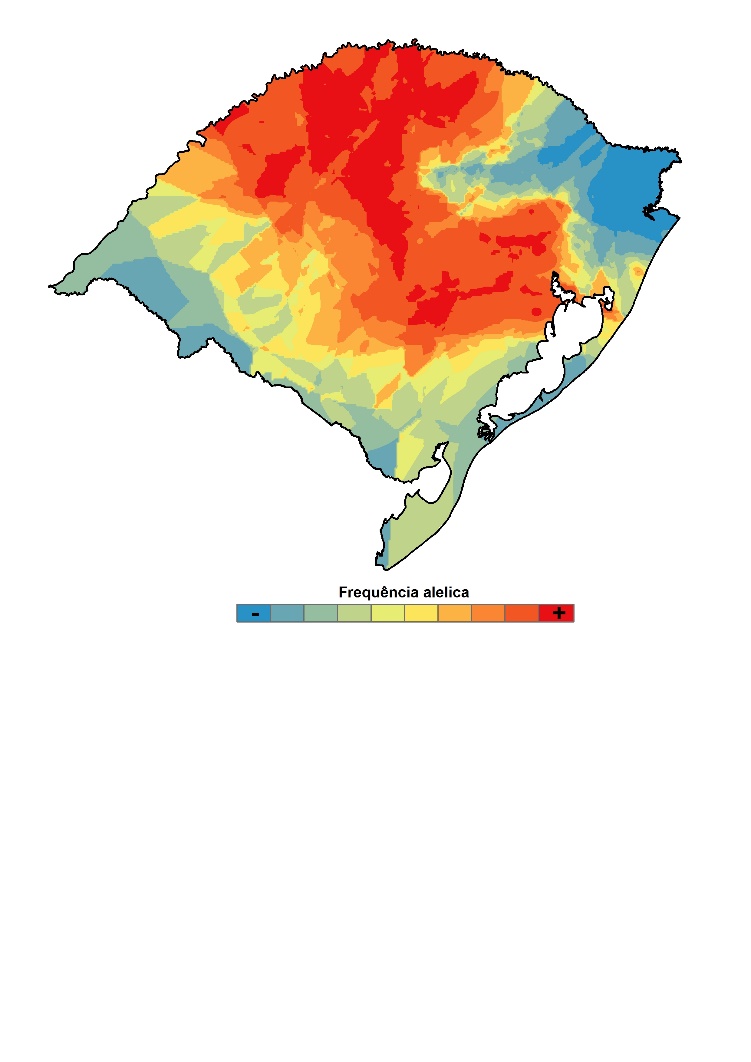


HLA-A*11


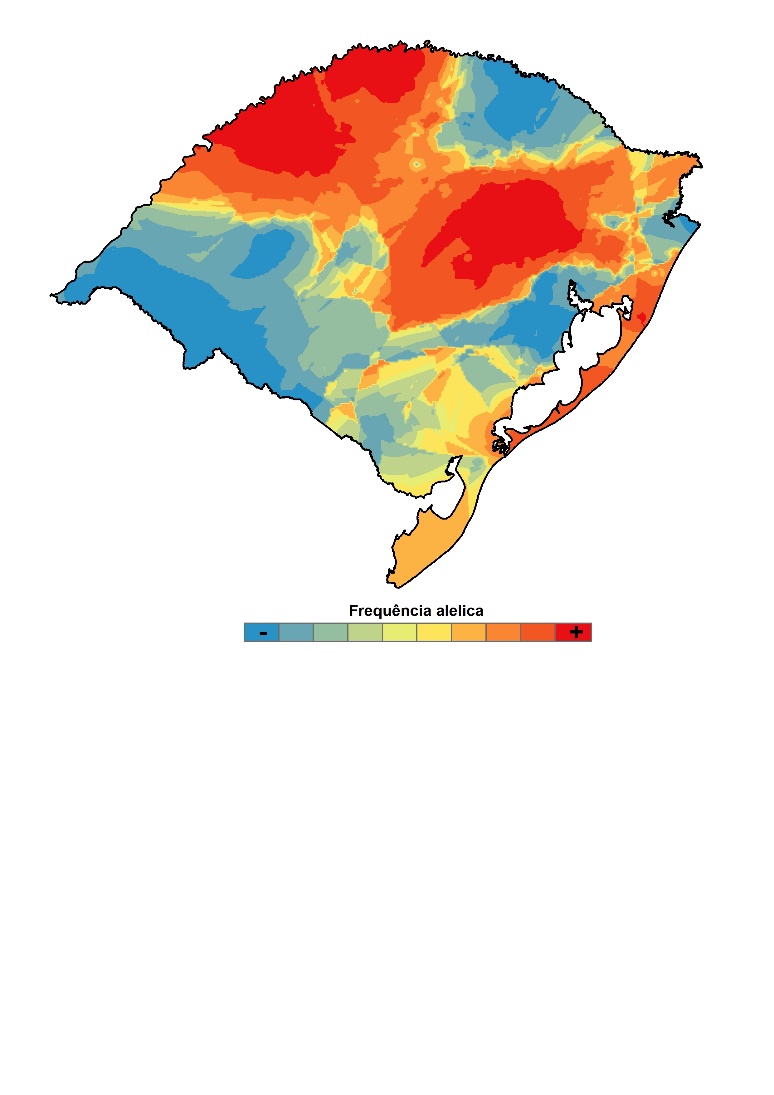


HLA-A*03


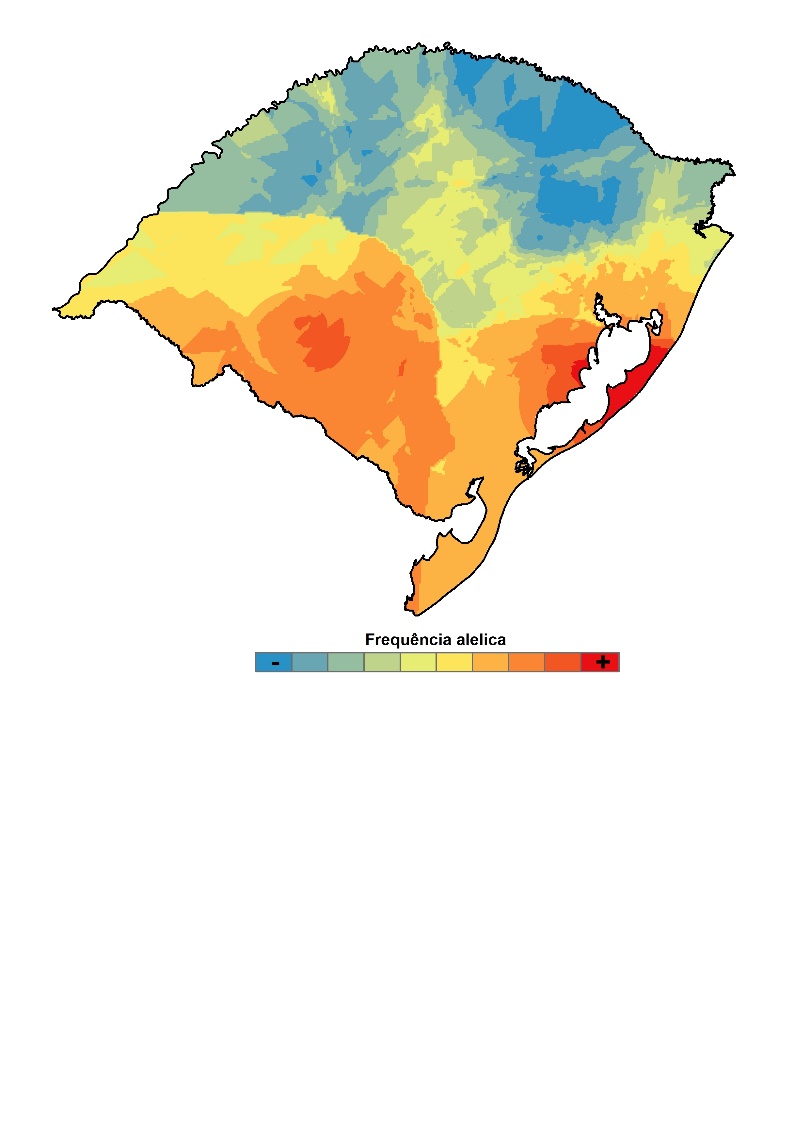


HLA-A*23


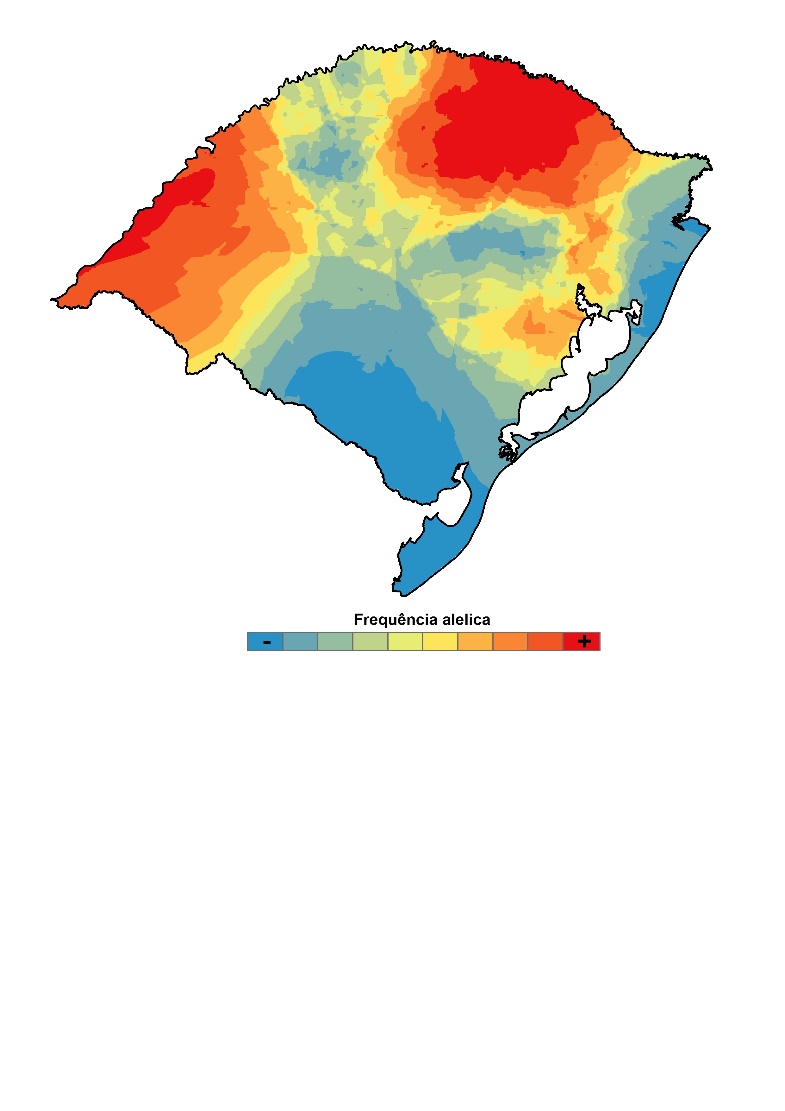


HLA-A*24


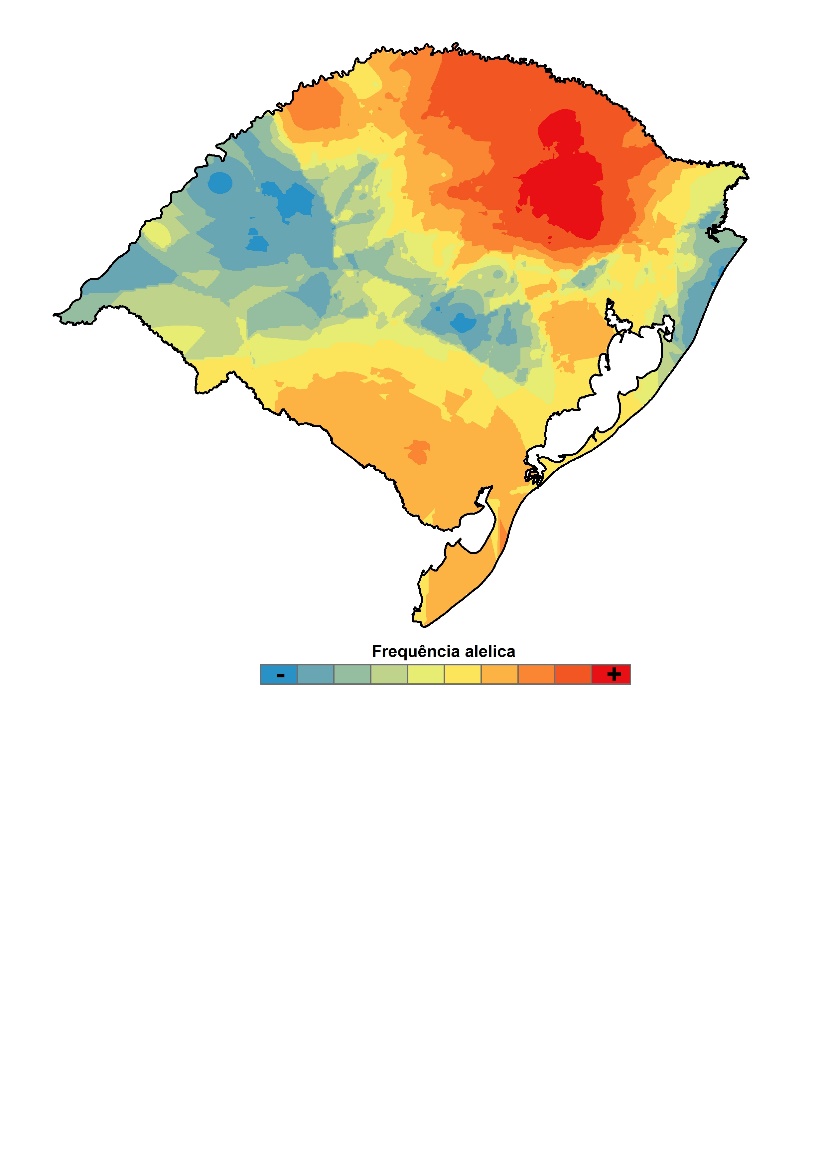


HLA-A*26


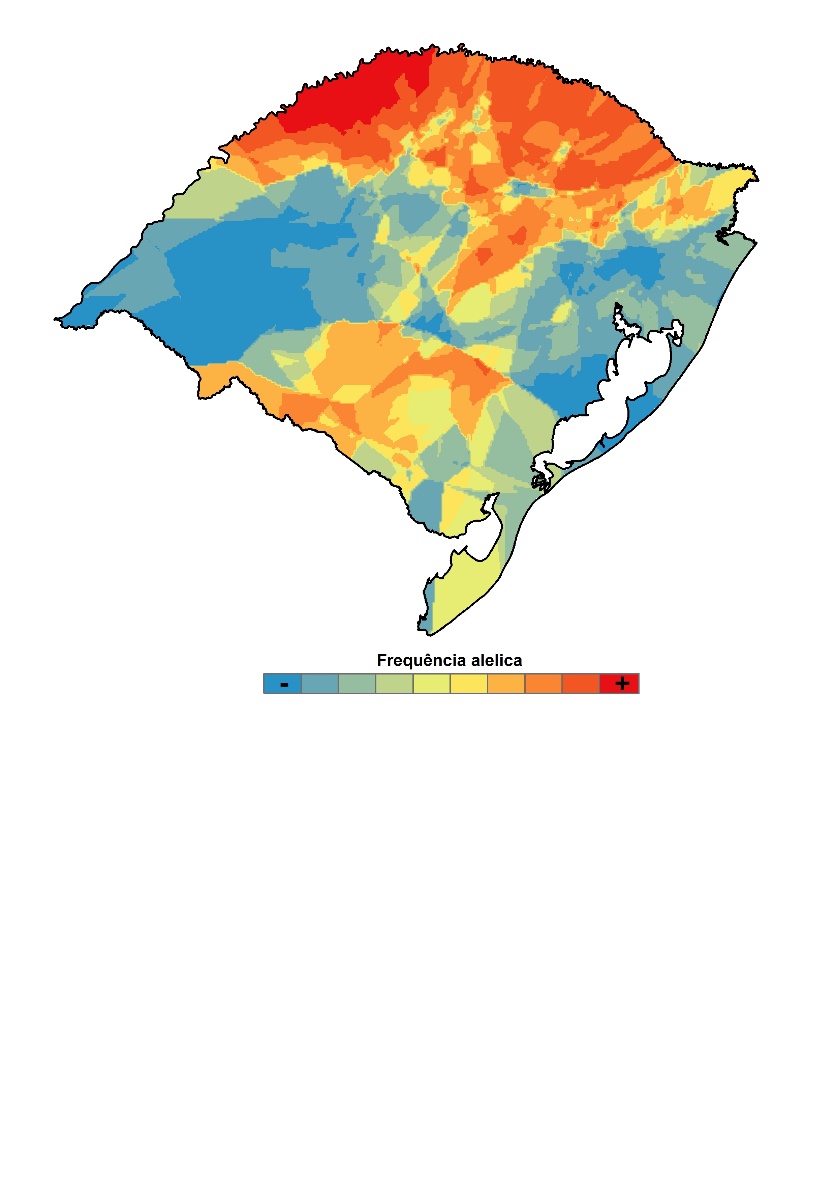


HLA-A*25


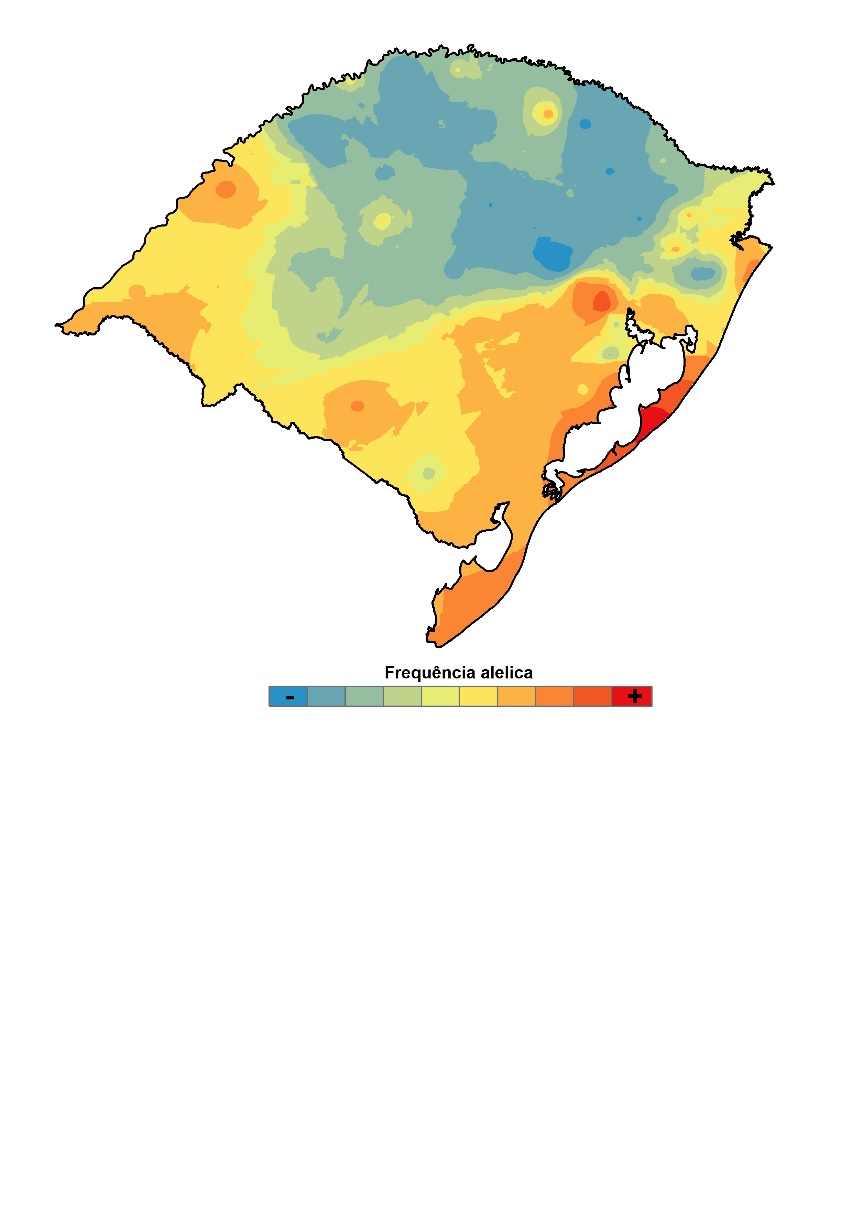


HLA-A*29


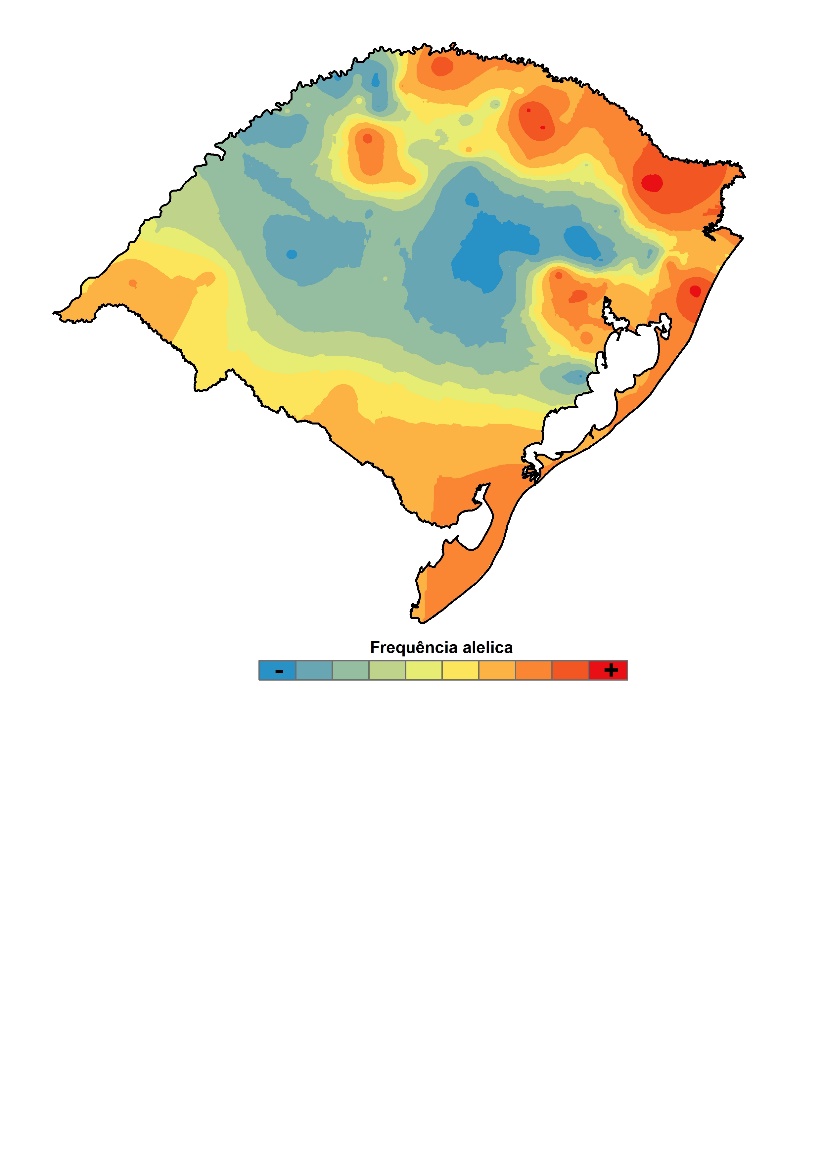


HLA-A*30


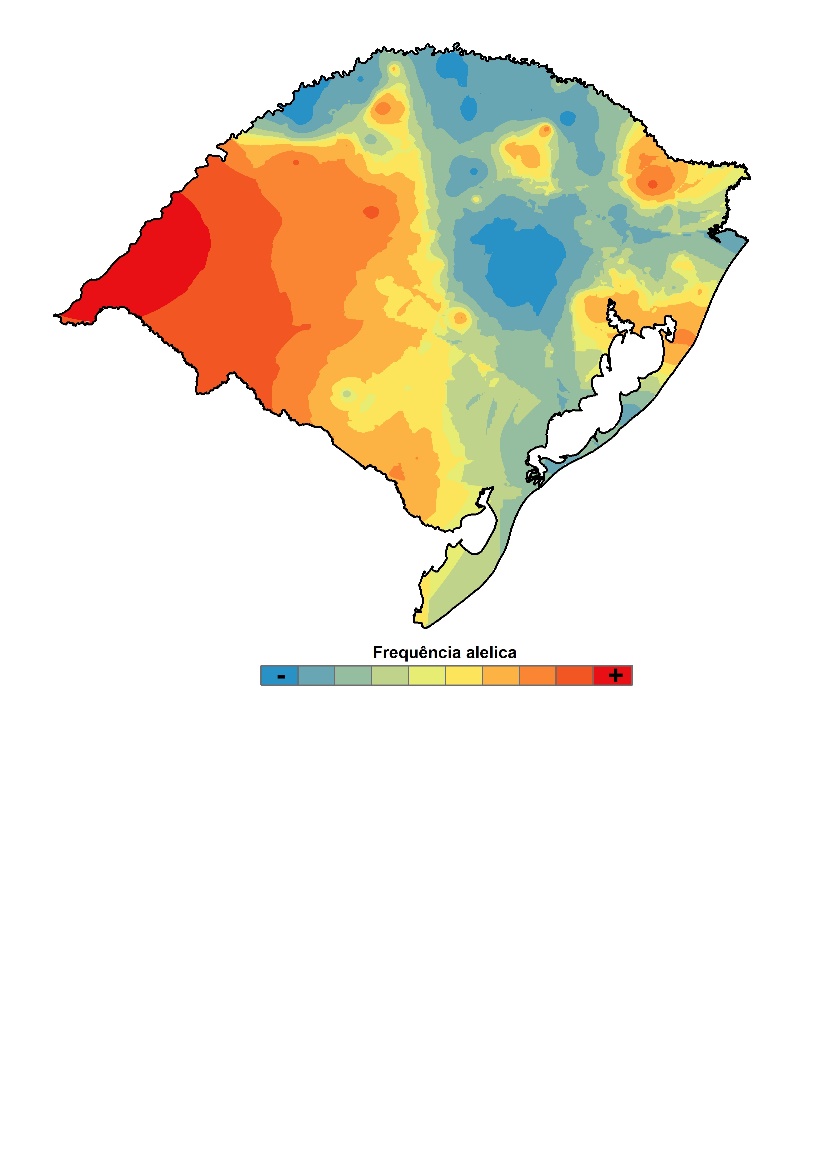


HLA-A*31


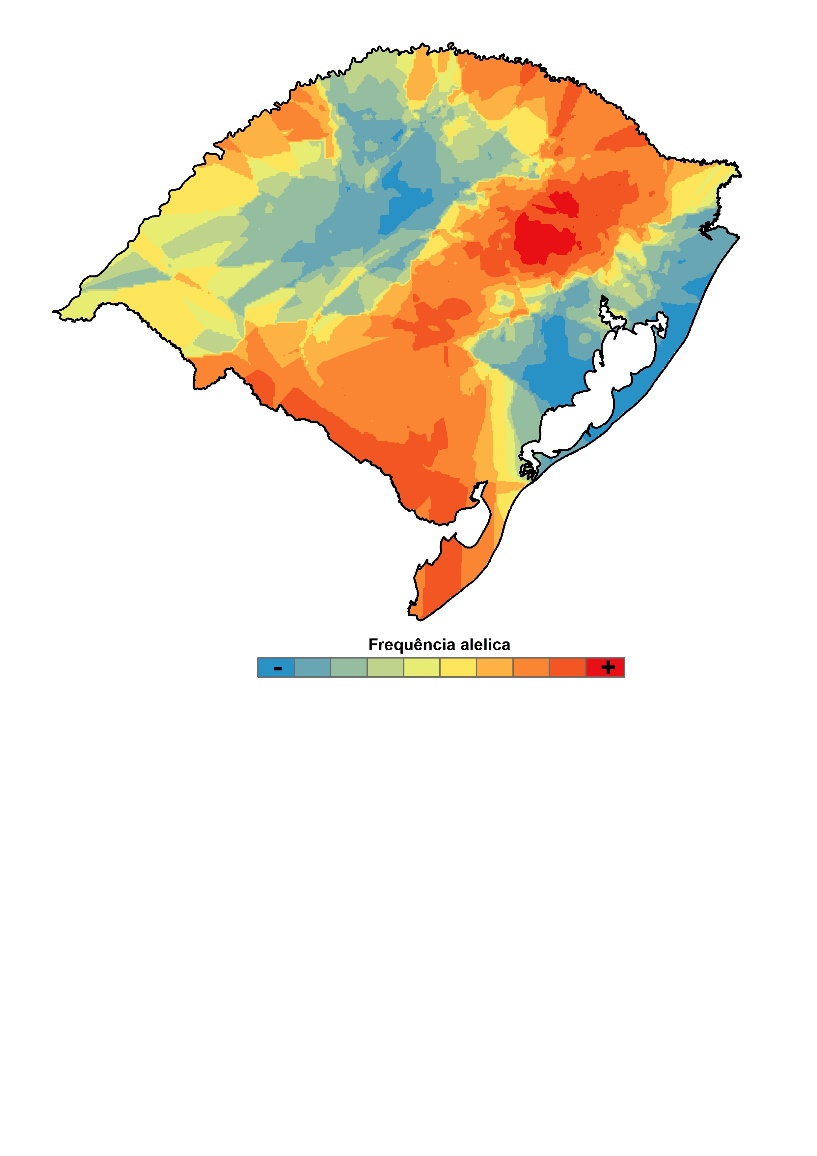


HLA-A*32


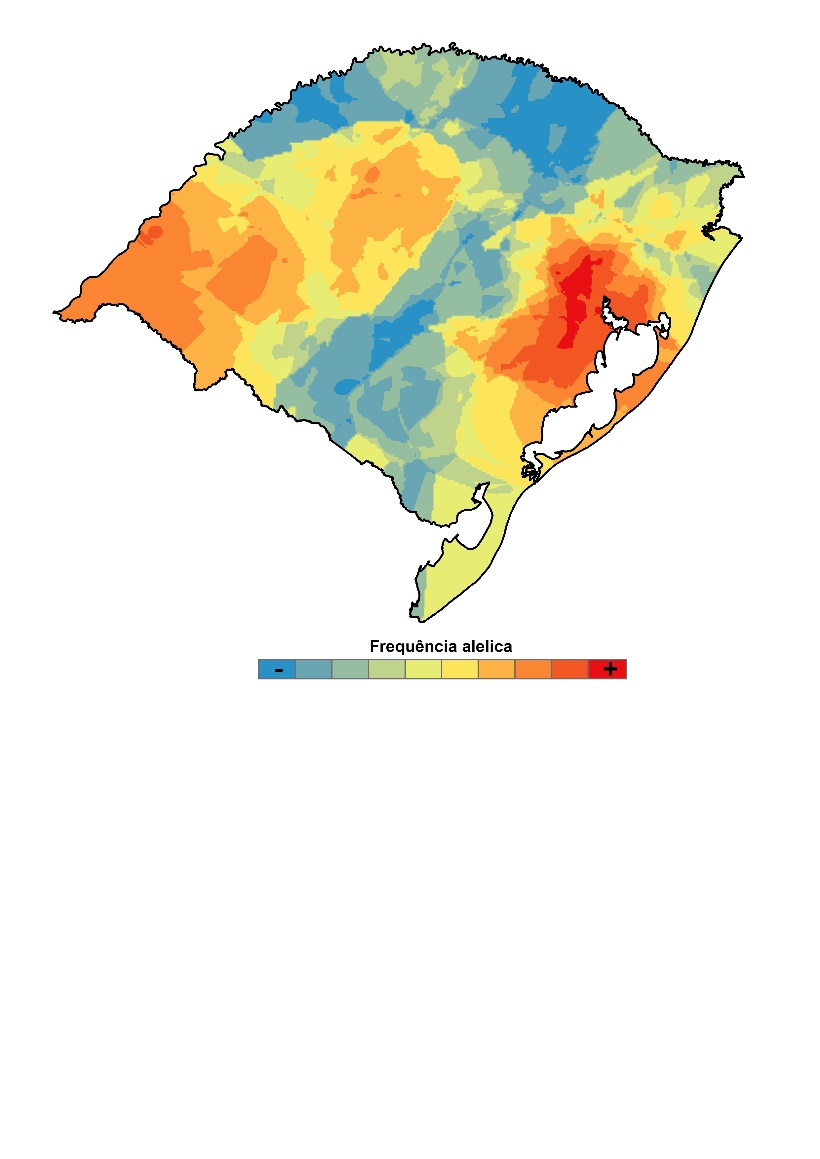


HLA-A*34


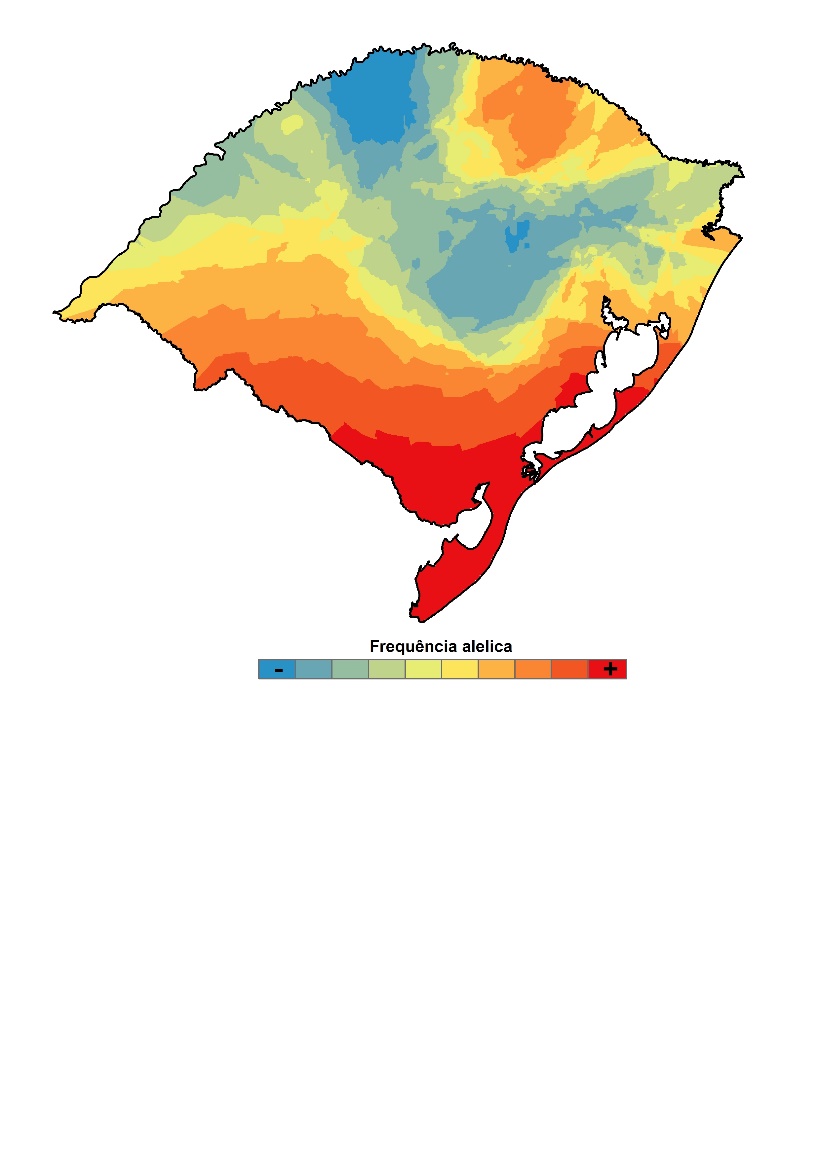


HLA-A*33


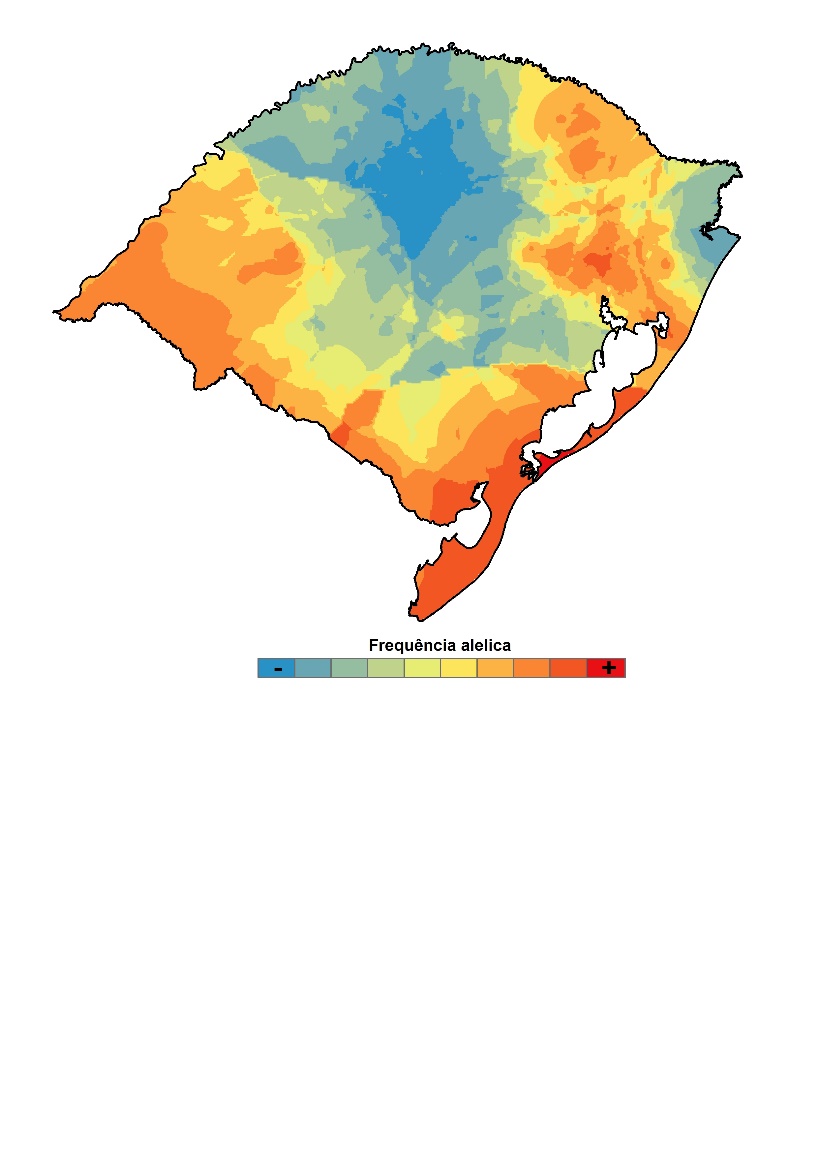


HLA-A*66


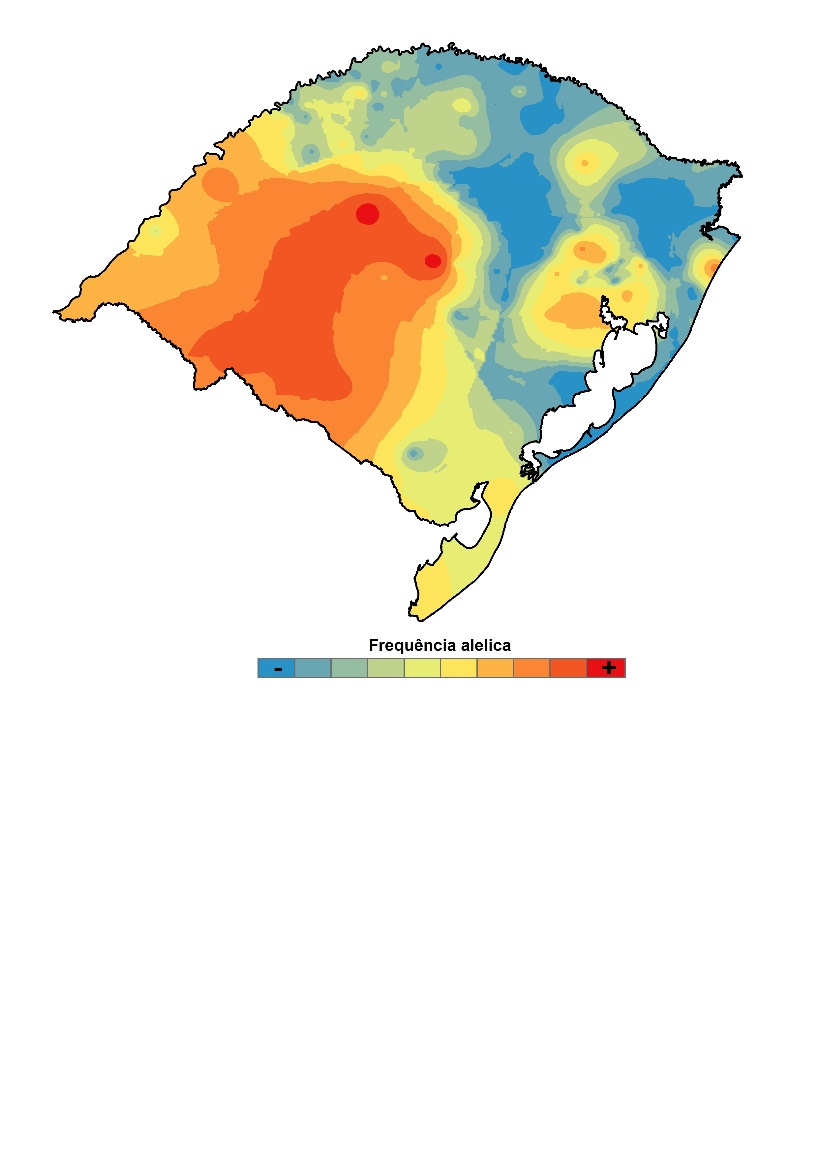


HLA-A*36


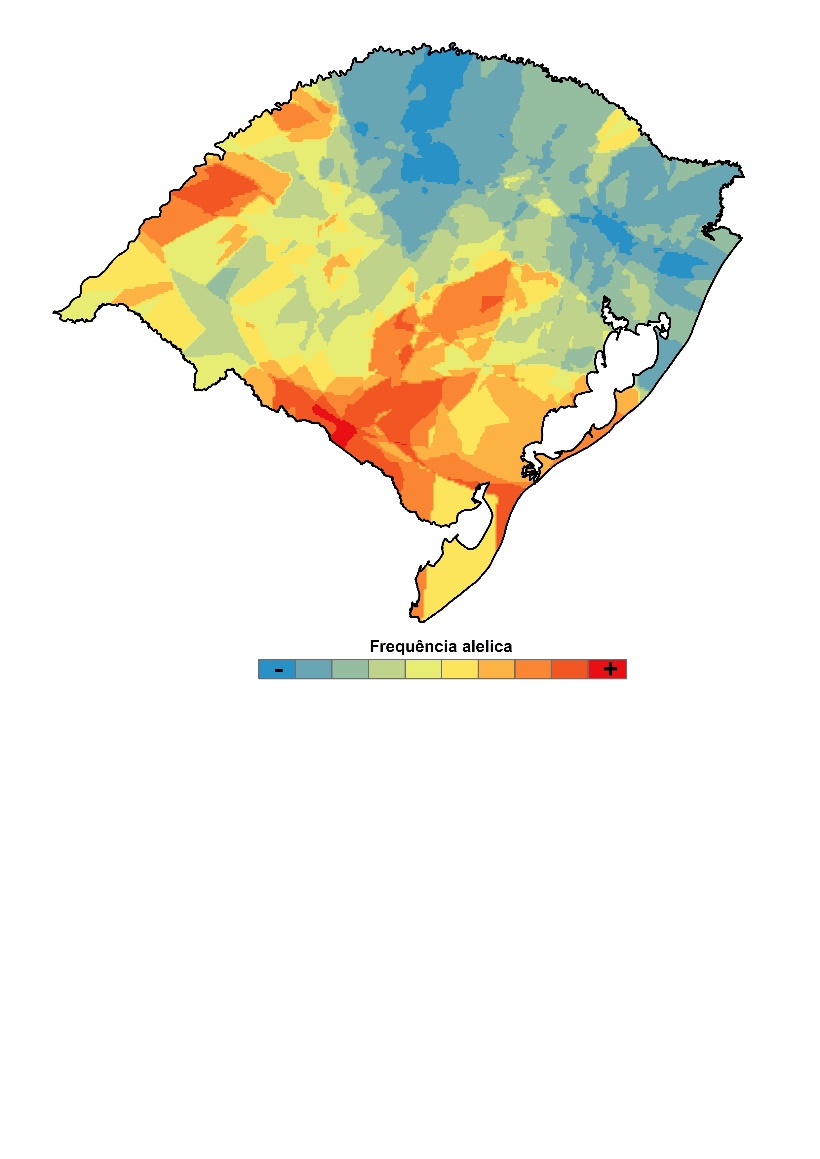


HLA-A*68


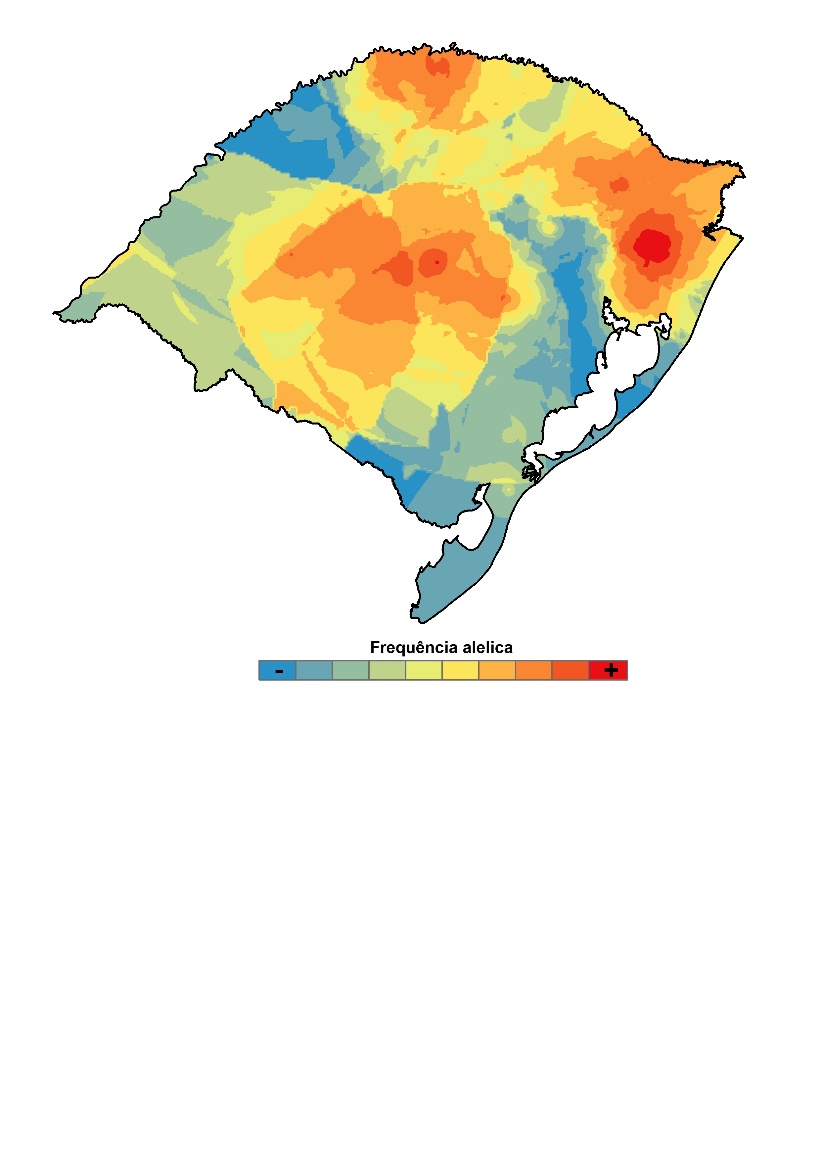


HLA-A*69


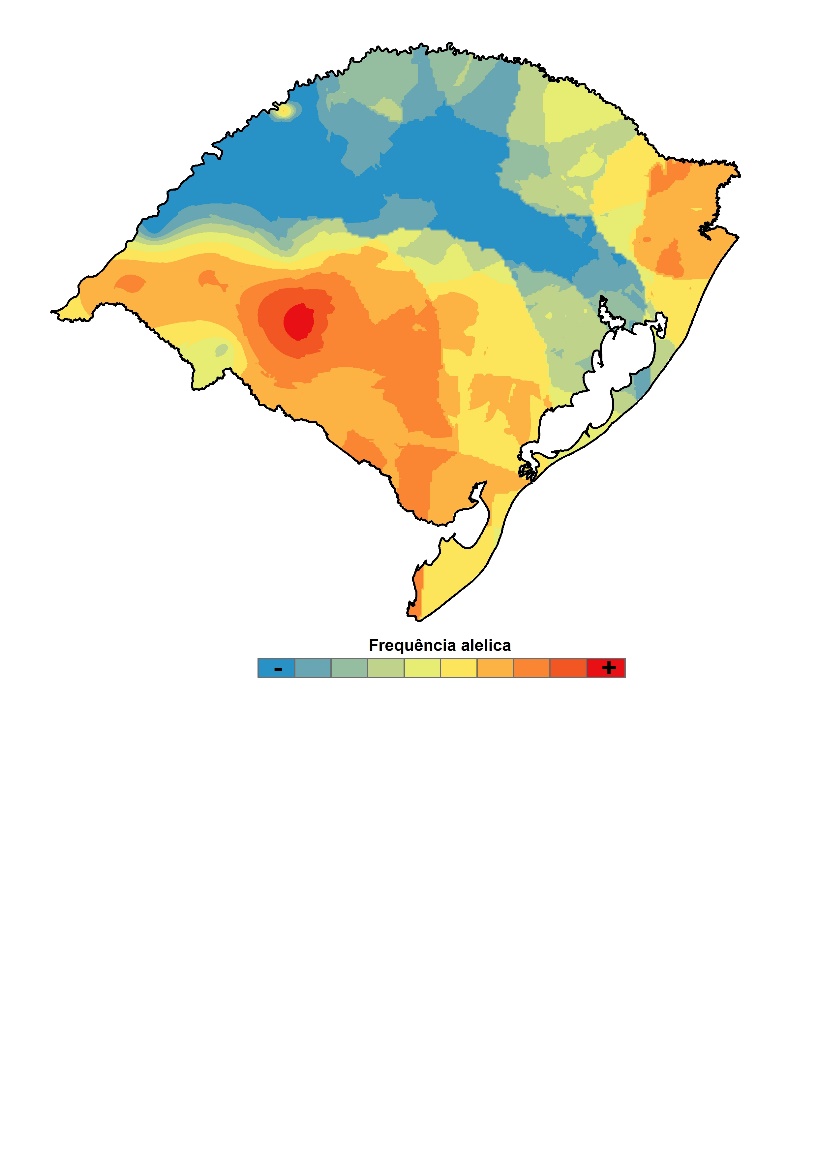


HLA-A*80


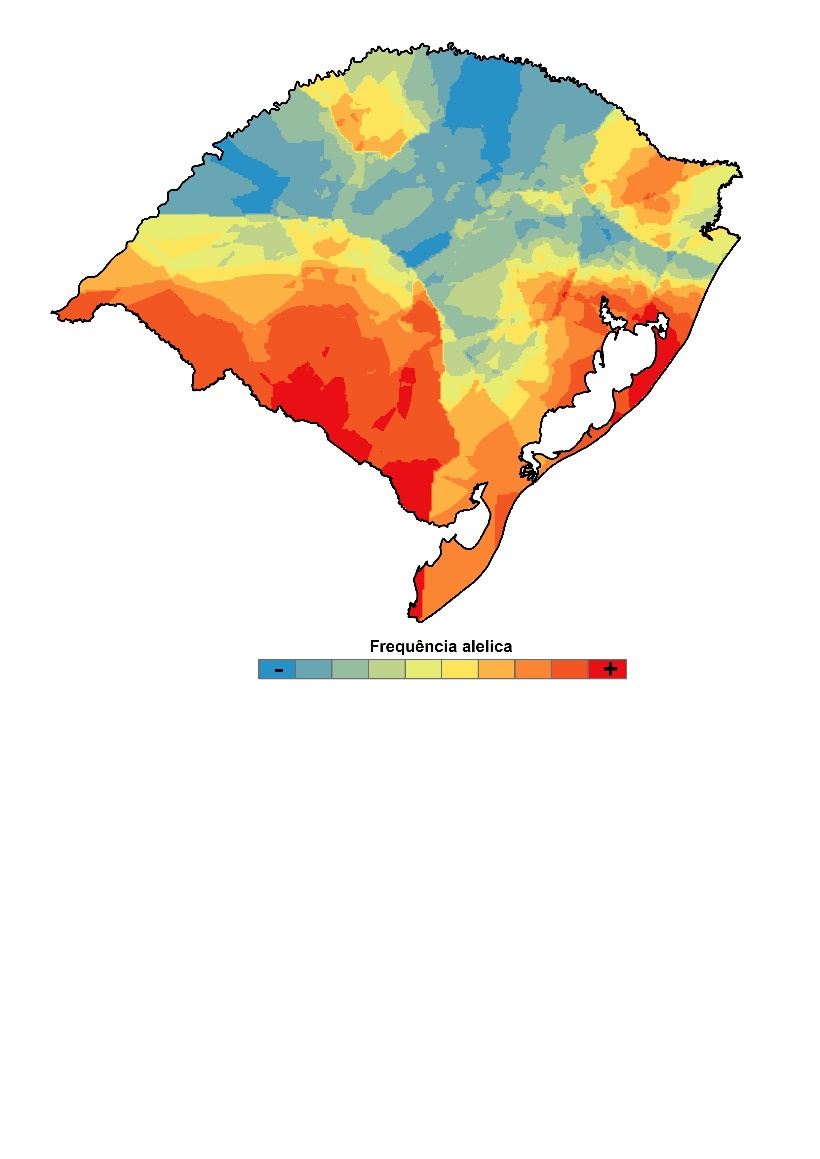


HLA-A*74


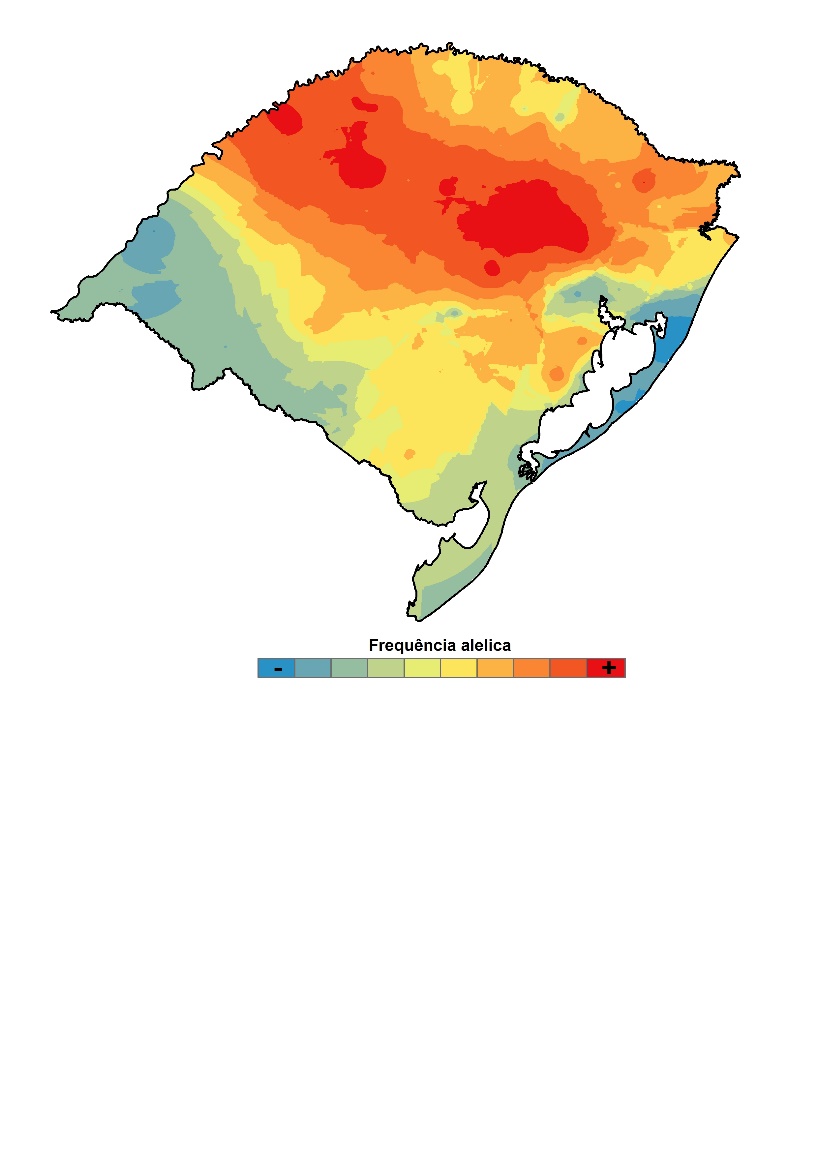
**HLA-B**


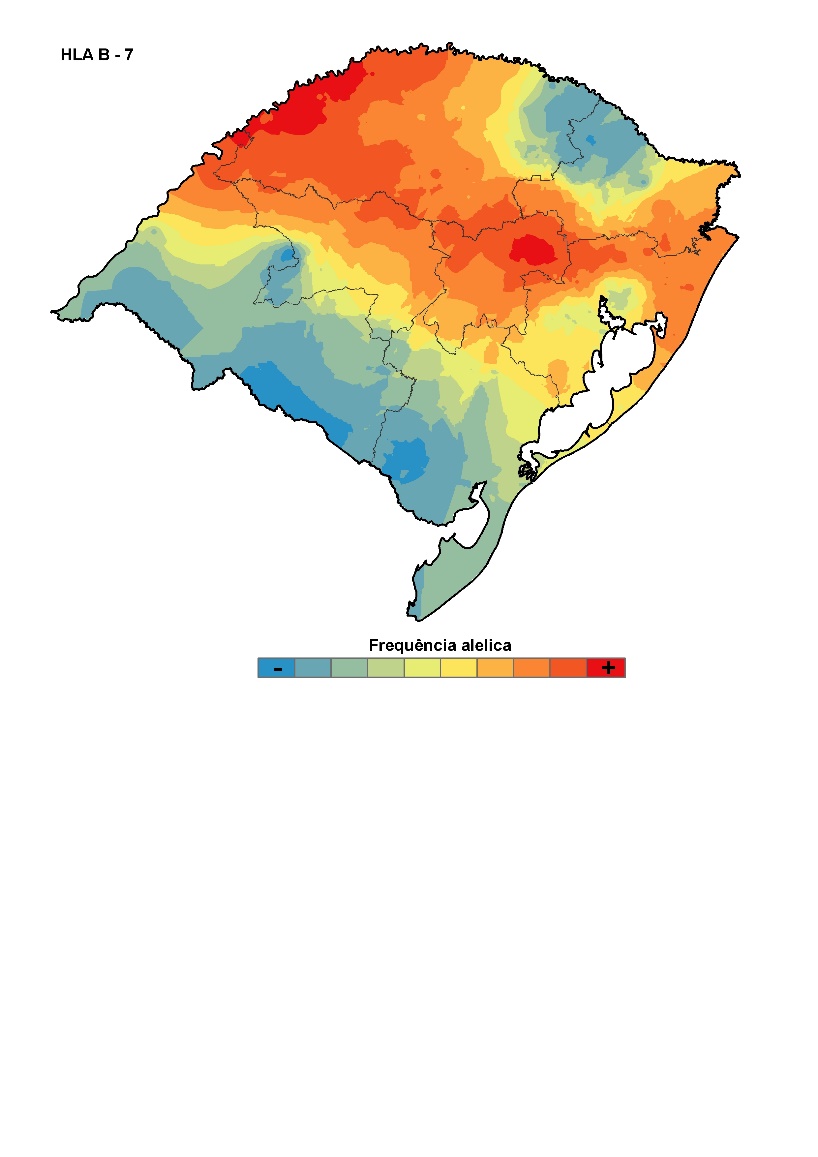


HLA-B*07

HLA-B*08


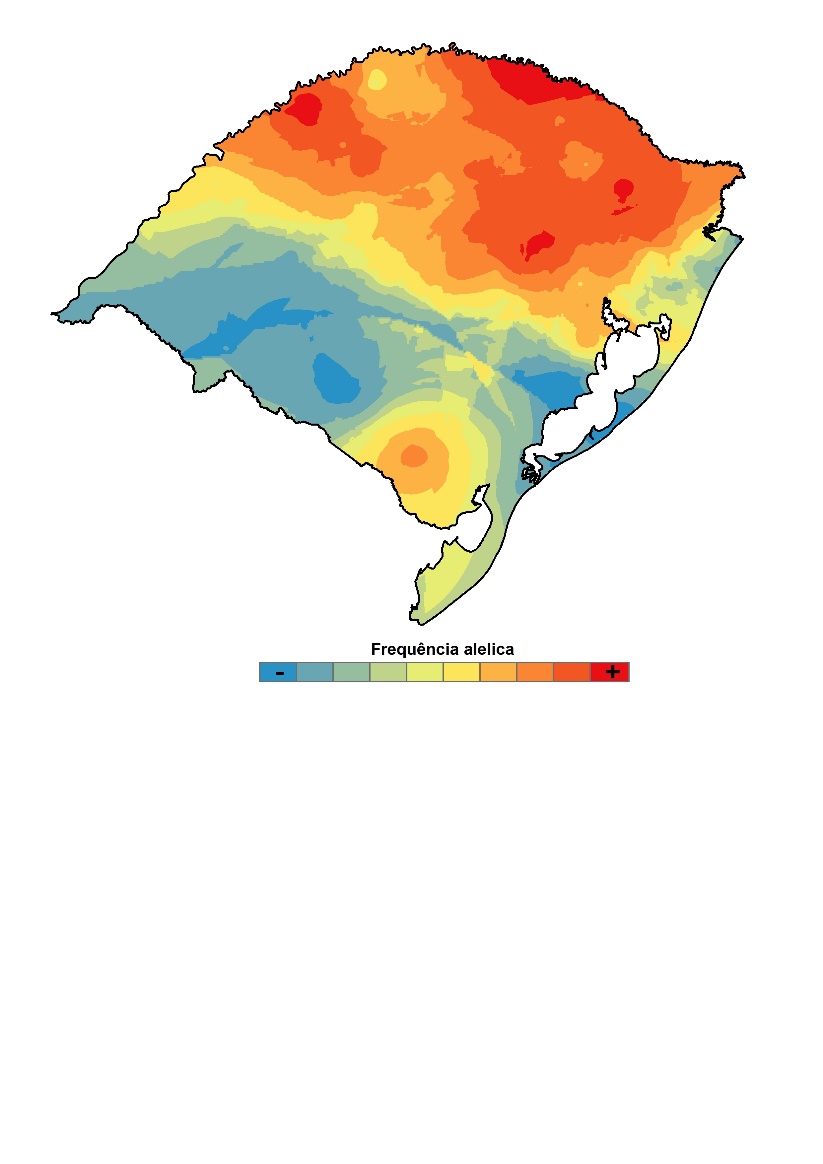


HLA-B*13


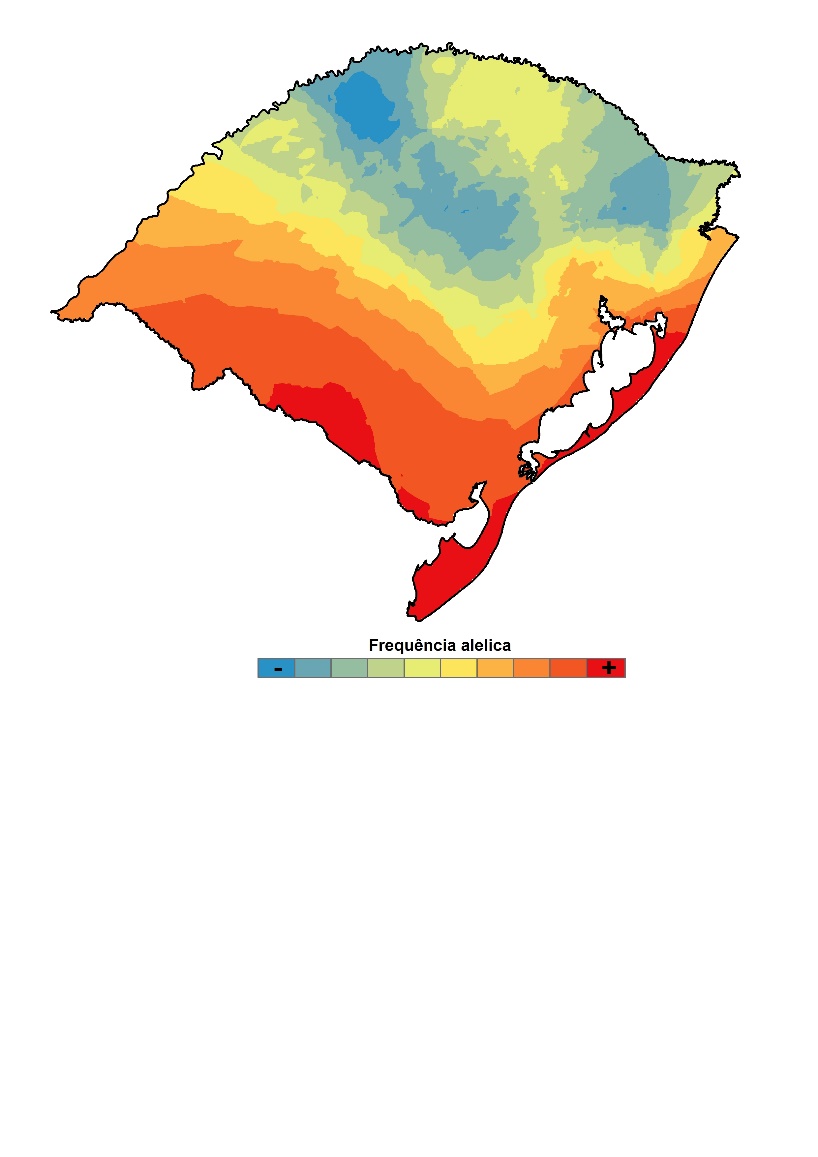


HLA-B*14


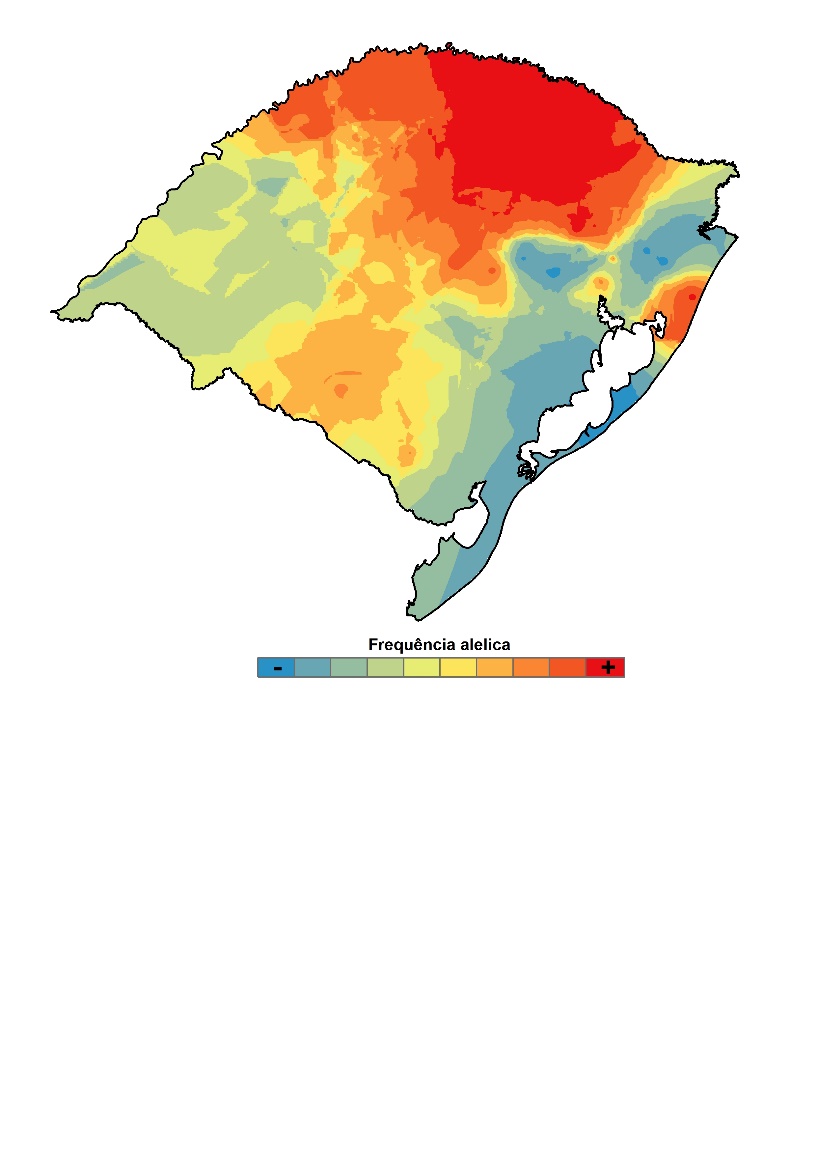


HLA-B*18


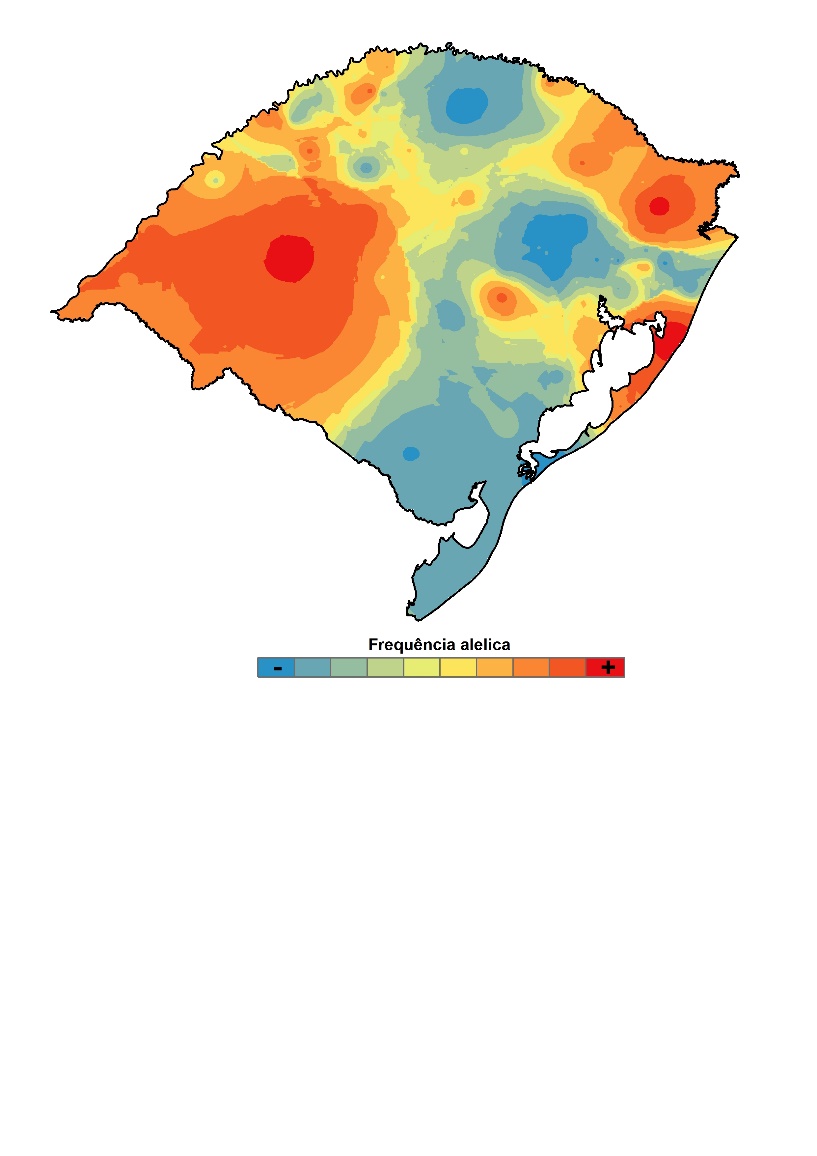


HLA-B*15


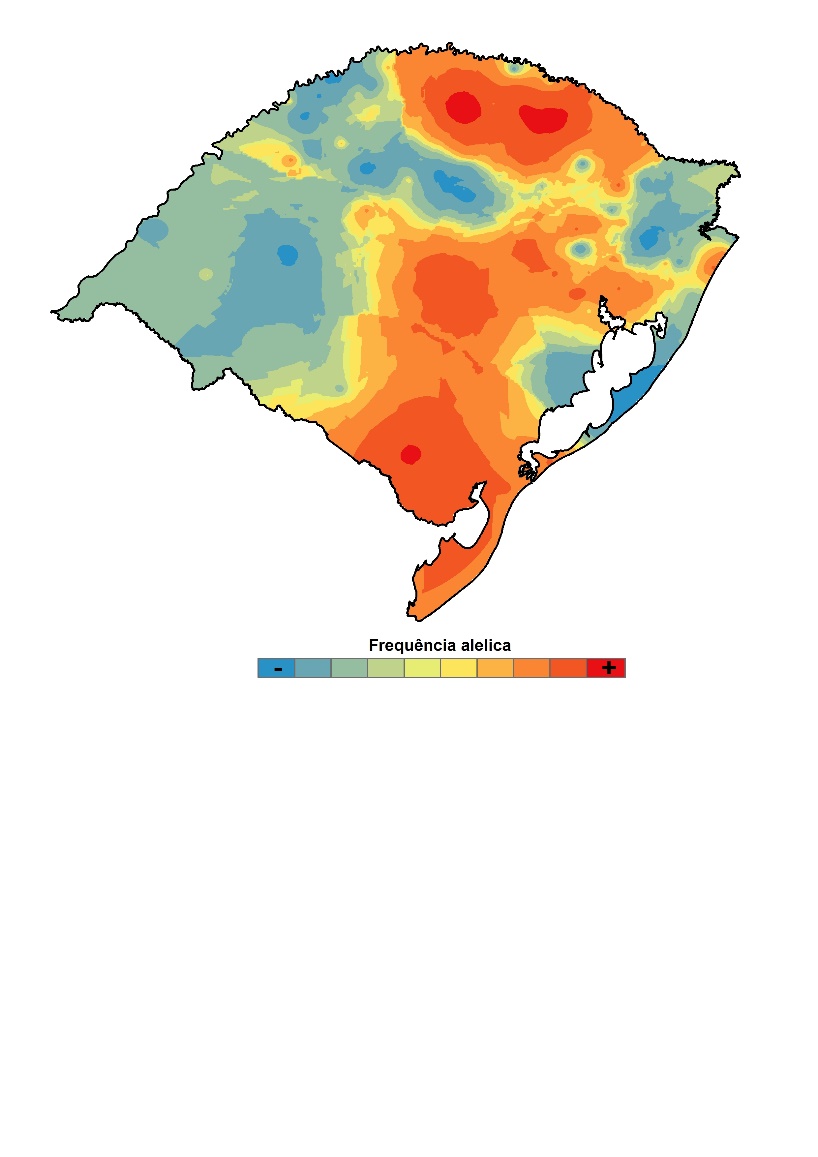


HLA-B*35


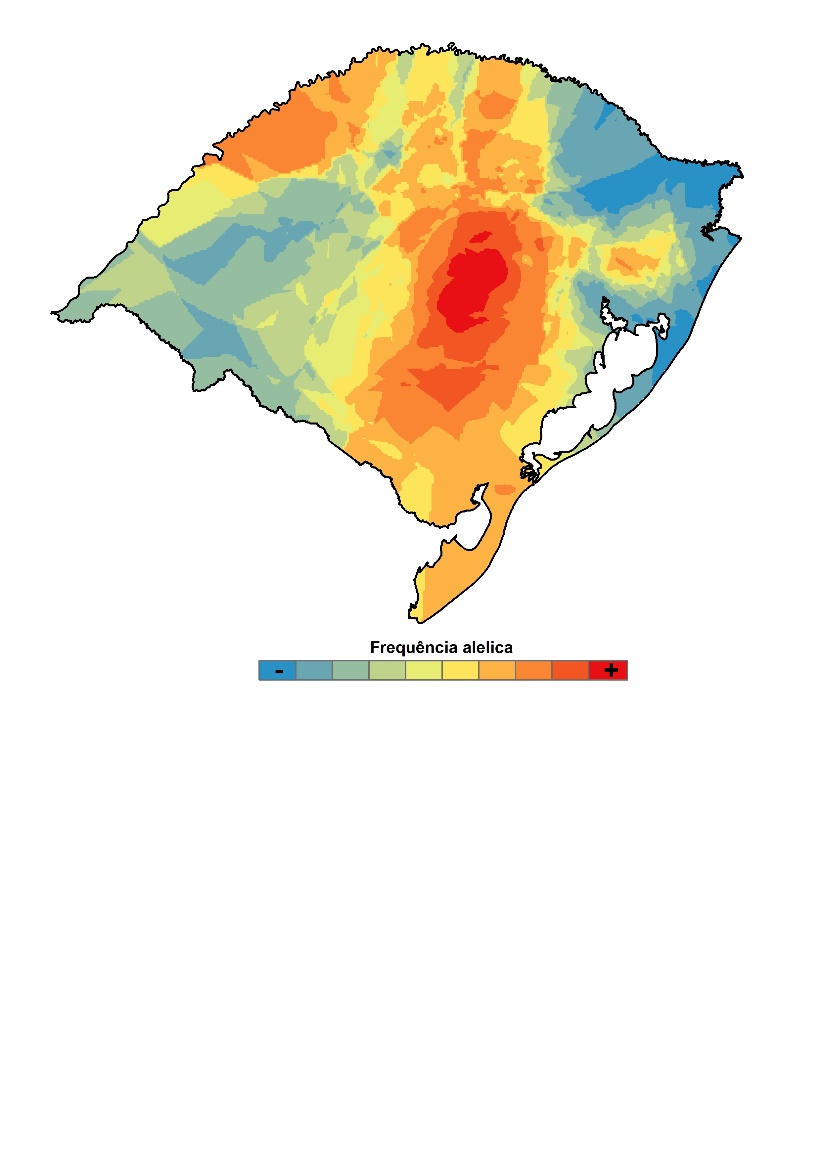


HLA-B*27


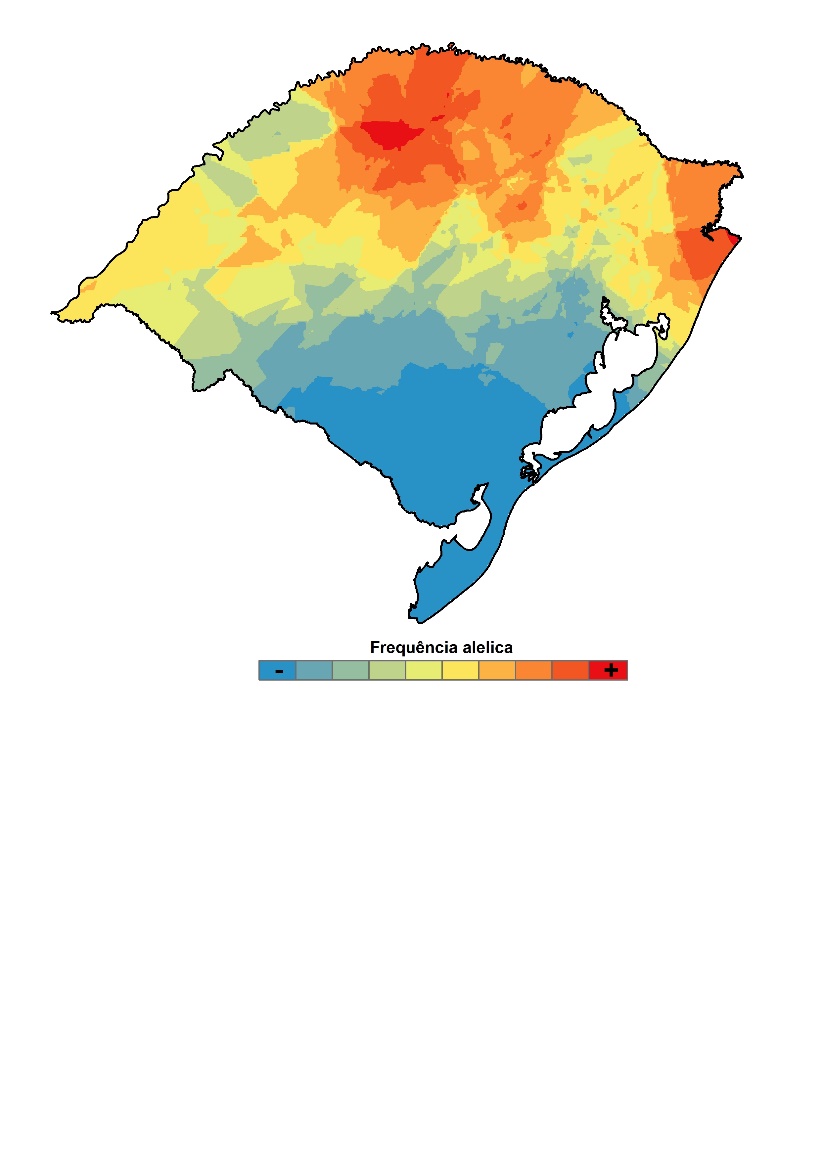


HLA-B*37


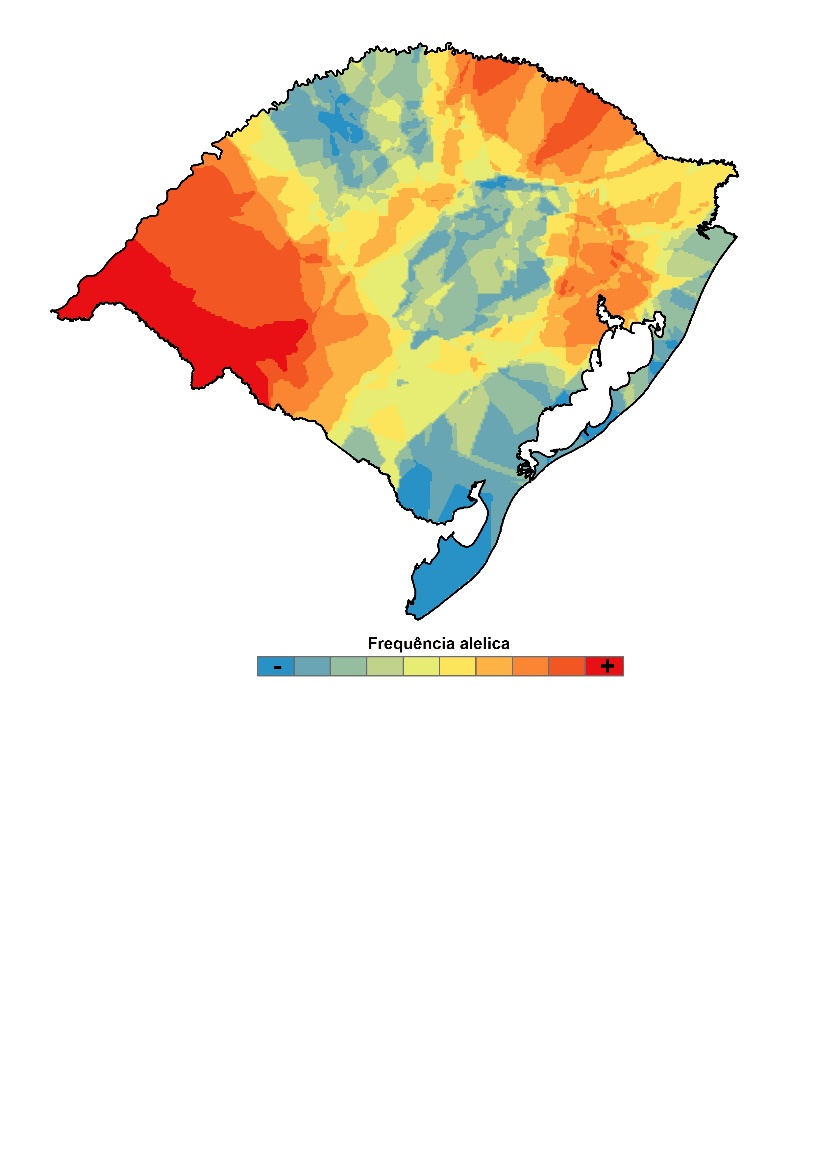


HLA-B*39


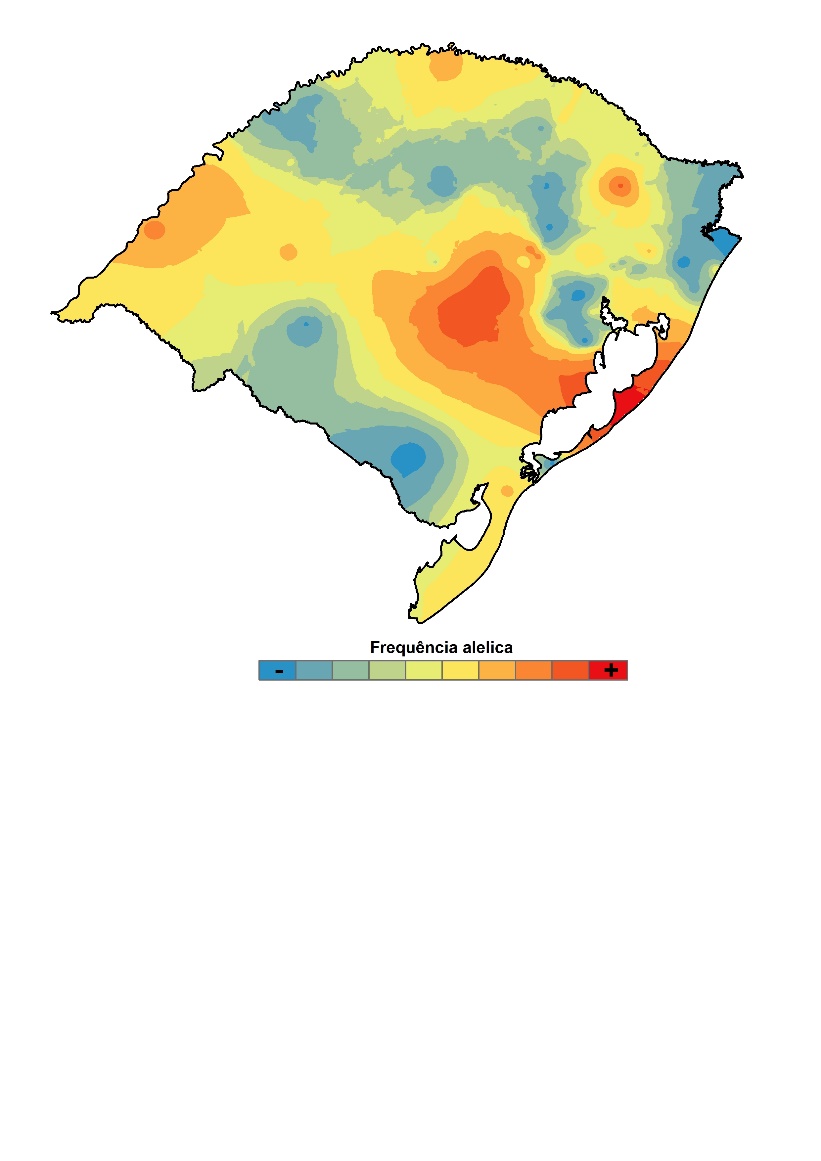


HLA-B*41


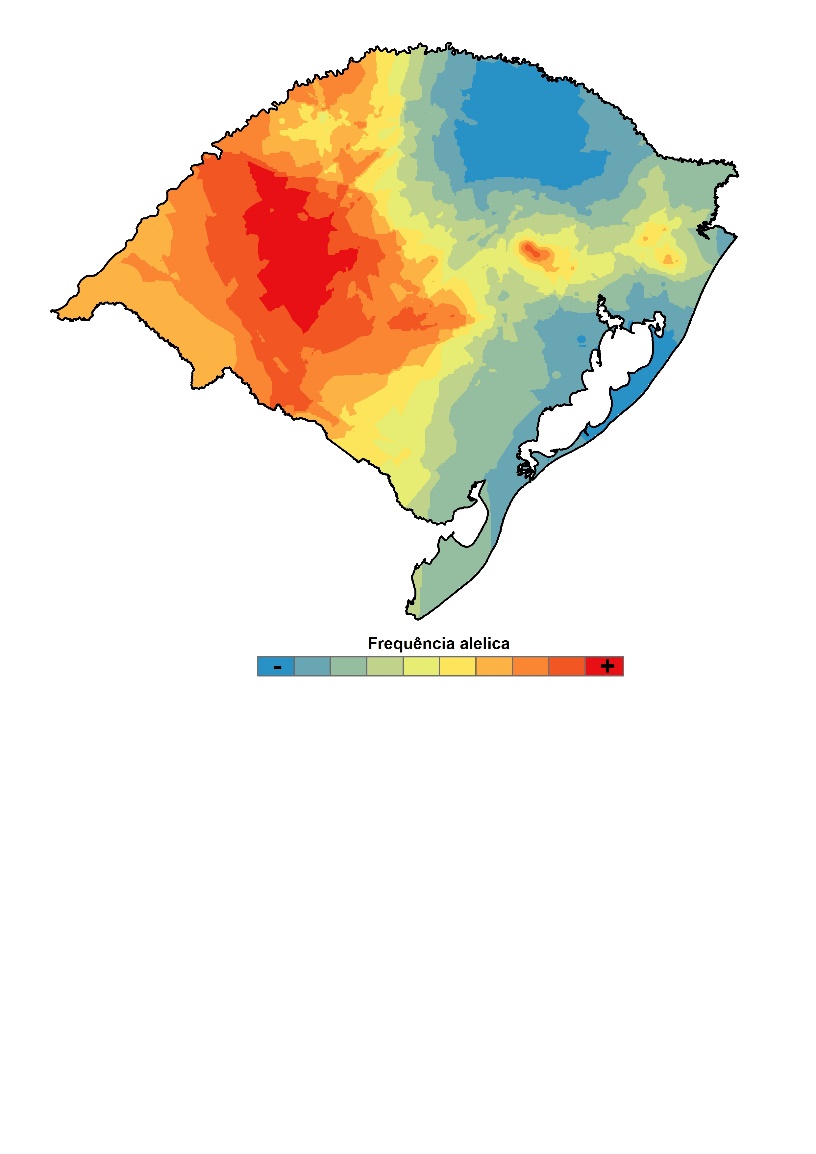


HLA-B*40


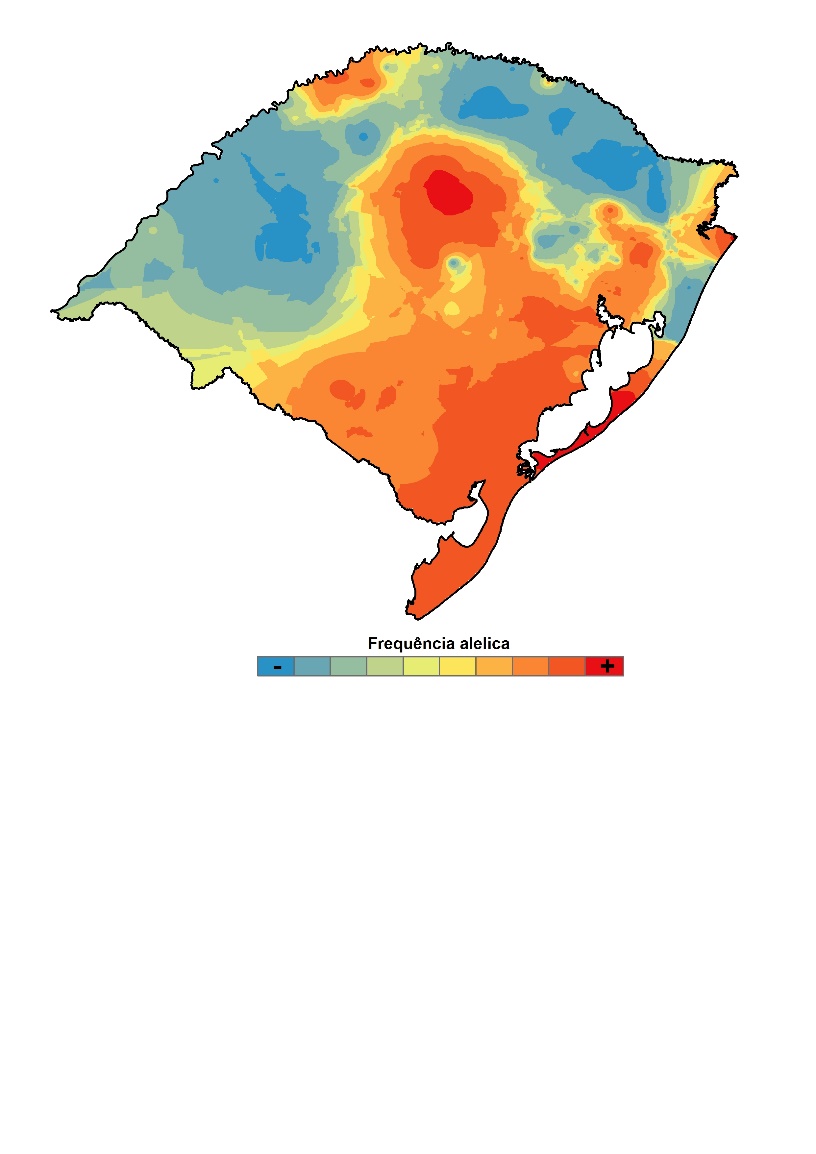


HLA-B*44


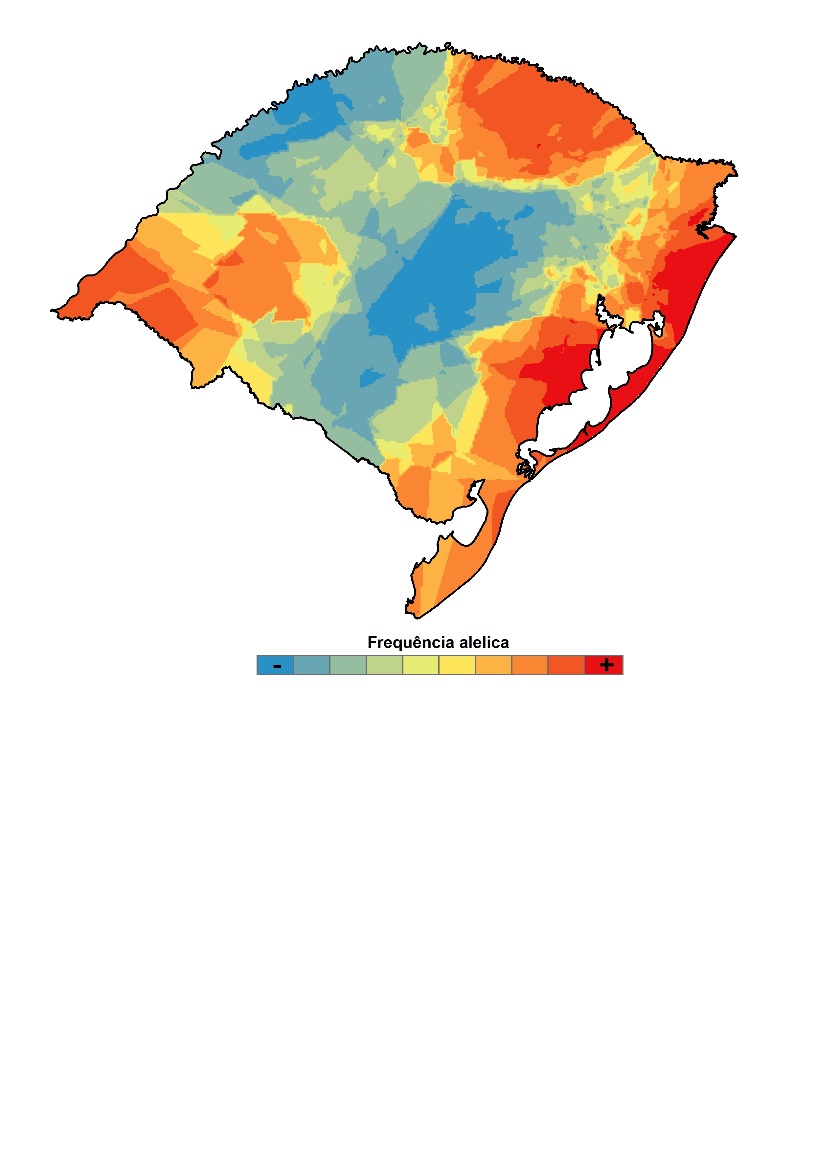


HLA-B*42


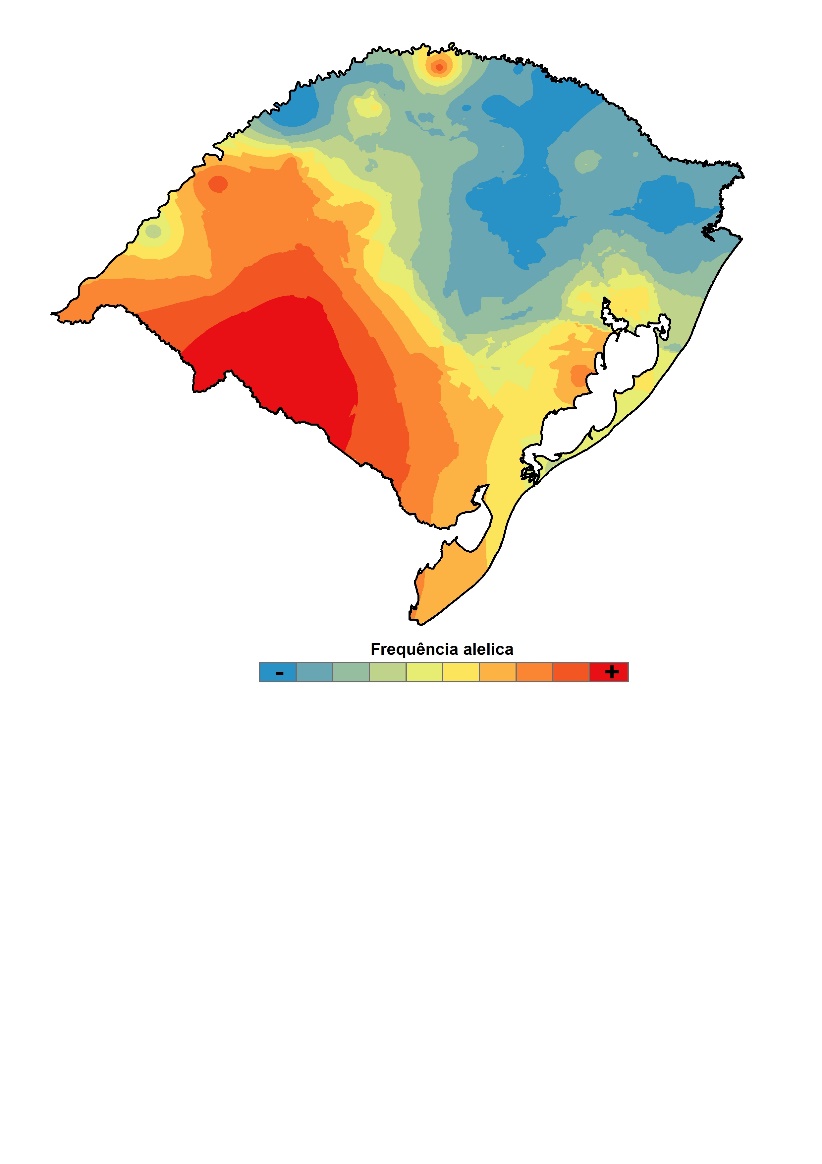


HLA-B*45


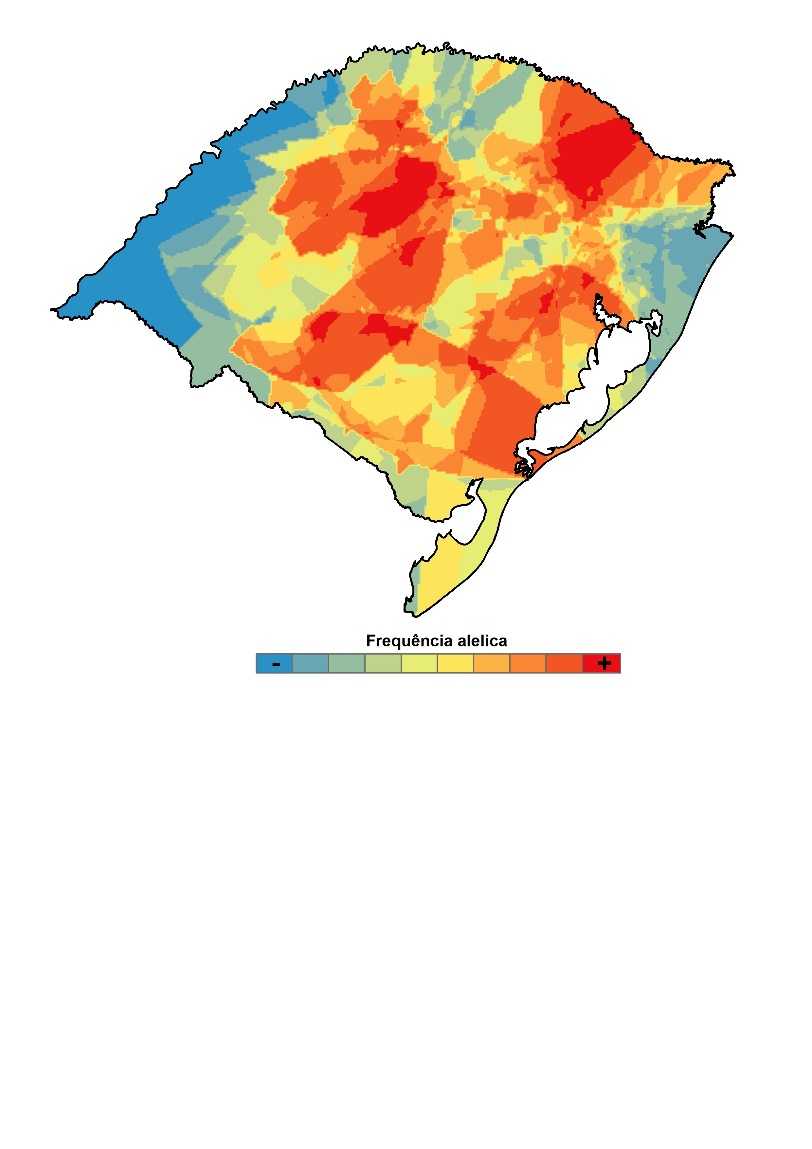


HLA-B*47


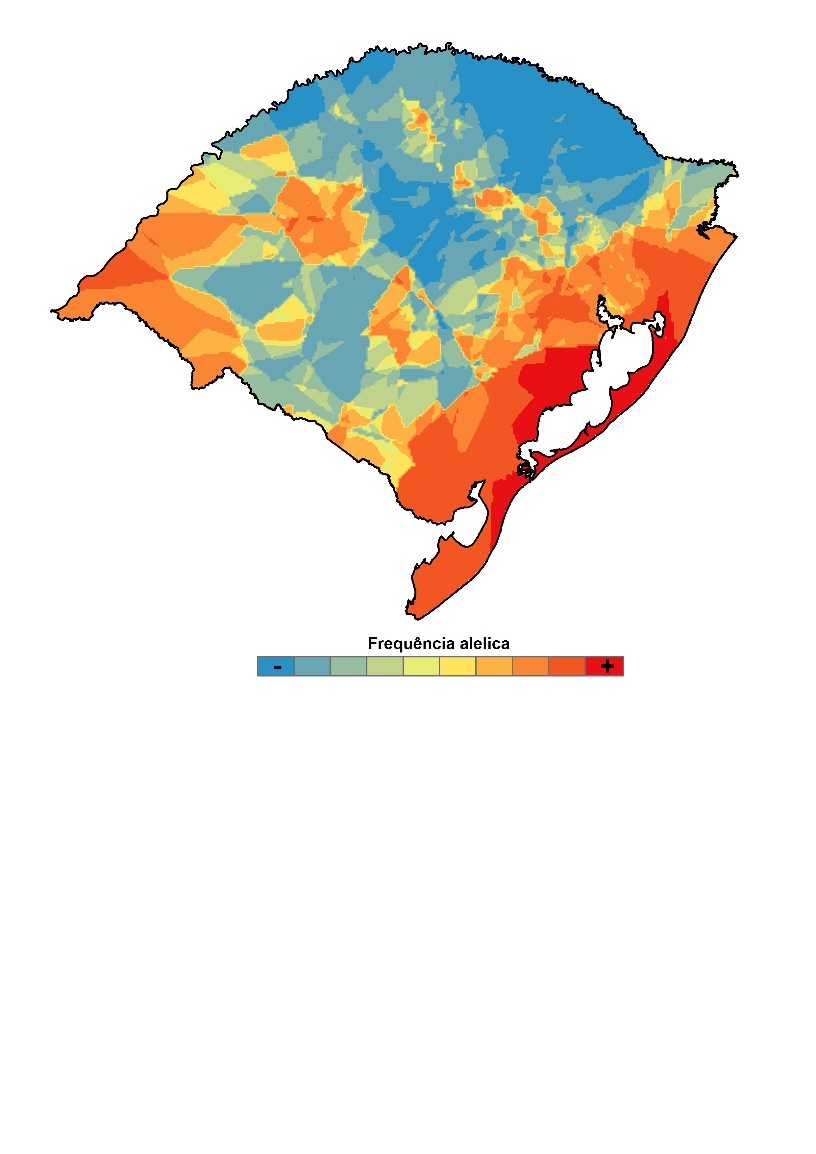


HLA-B*49


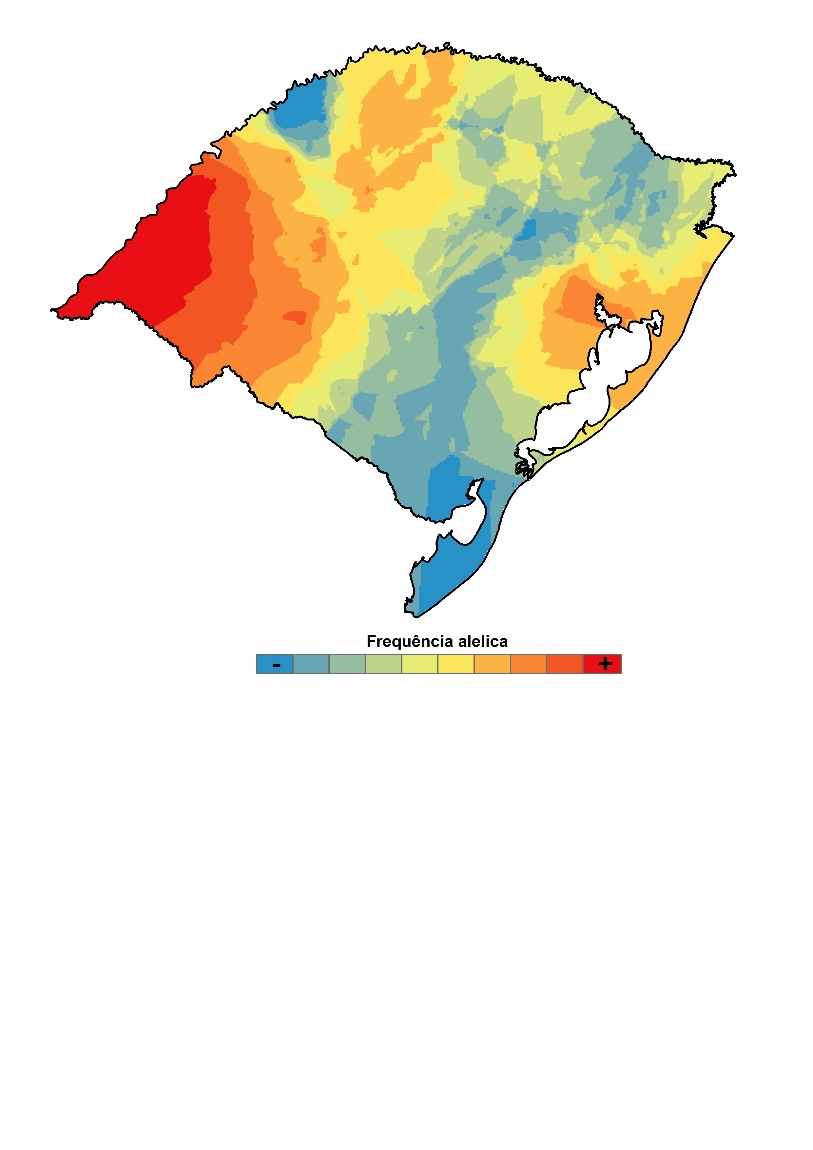


HLA-B*48


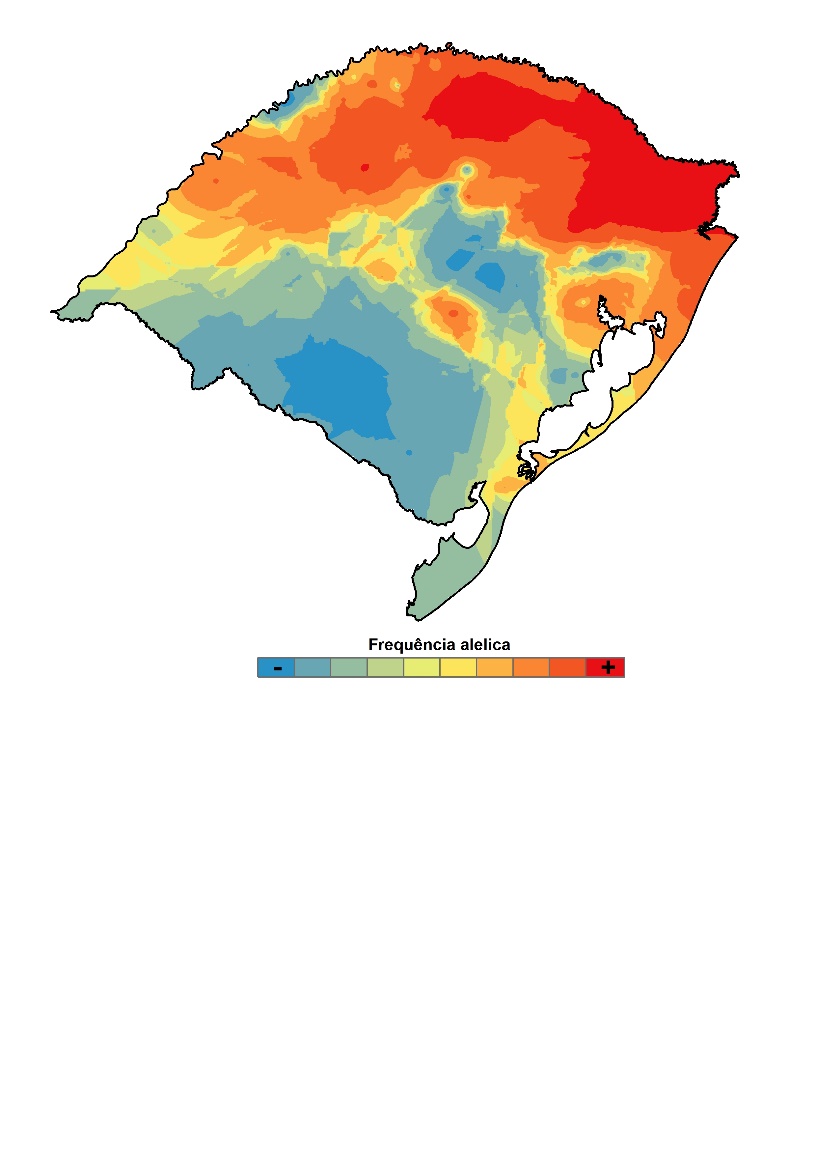


HLA-B*51


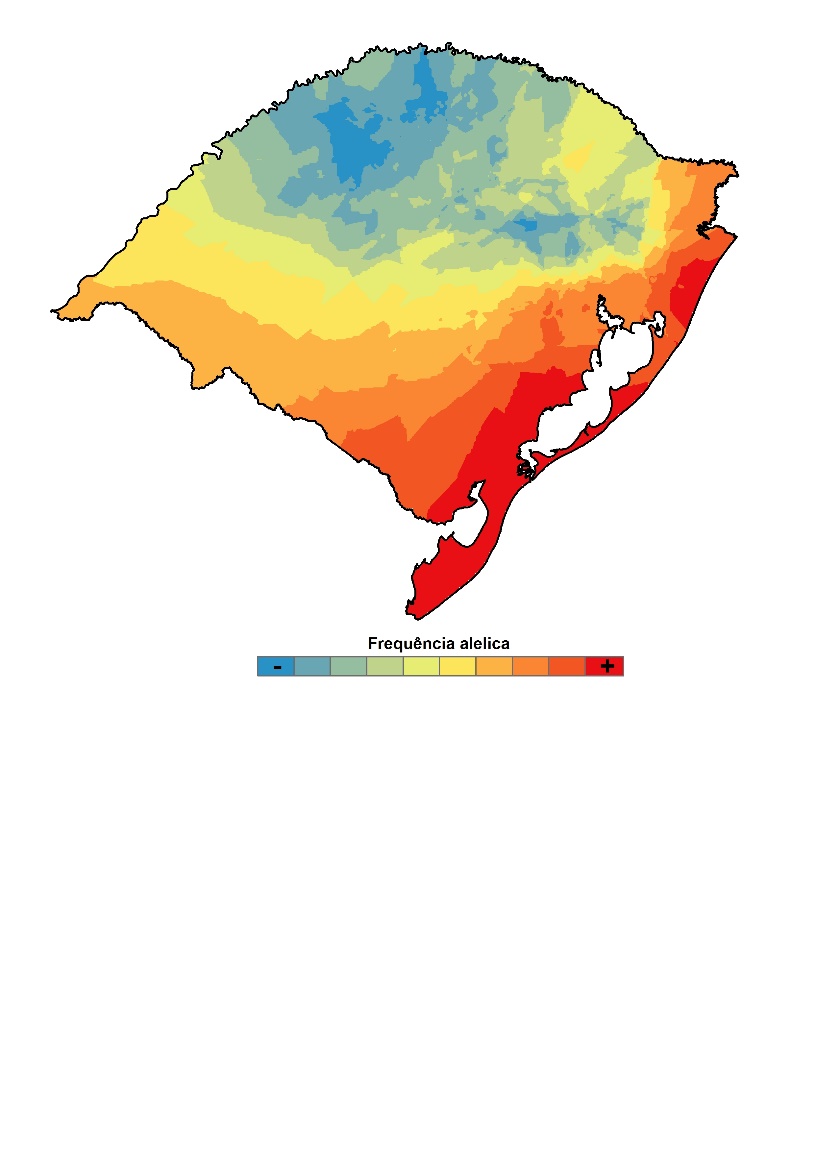


HLA-B*50


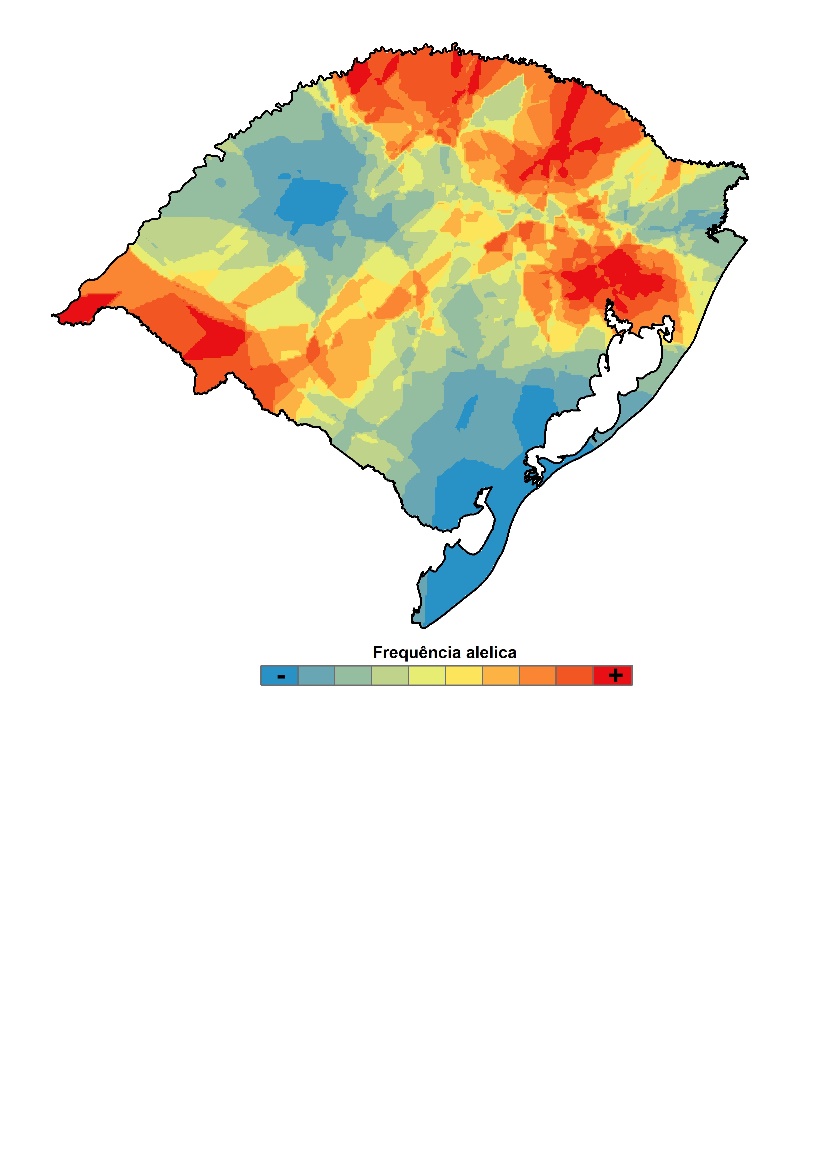


HLA-B*52


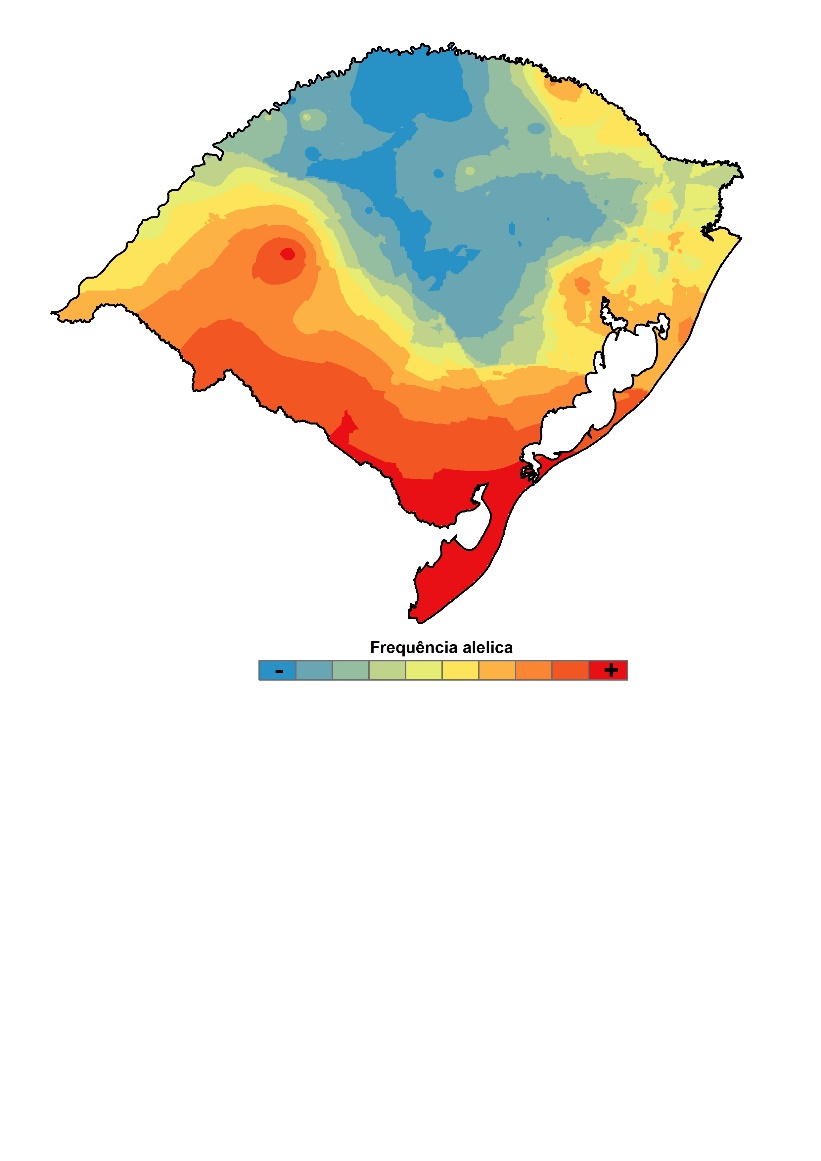


HLA-B*53


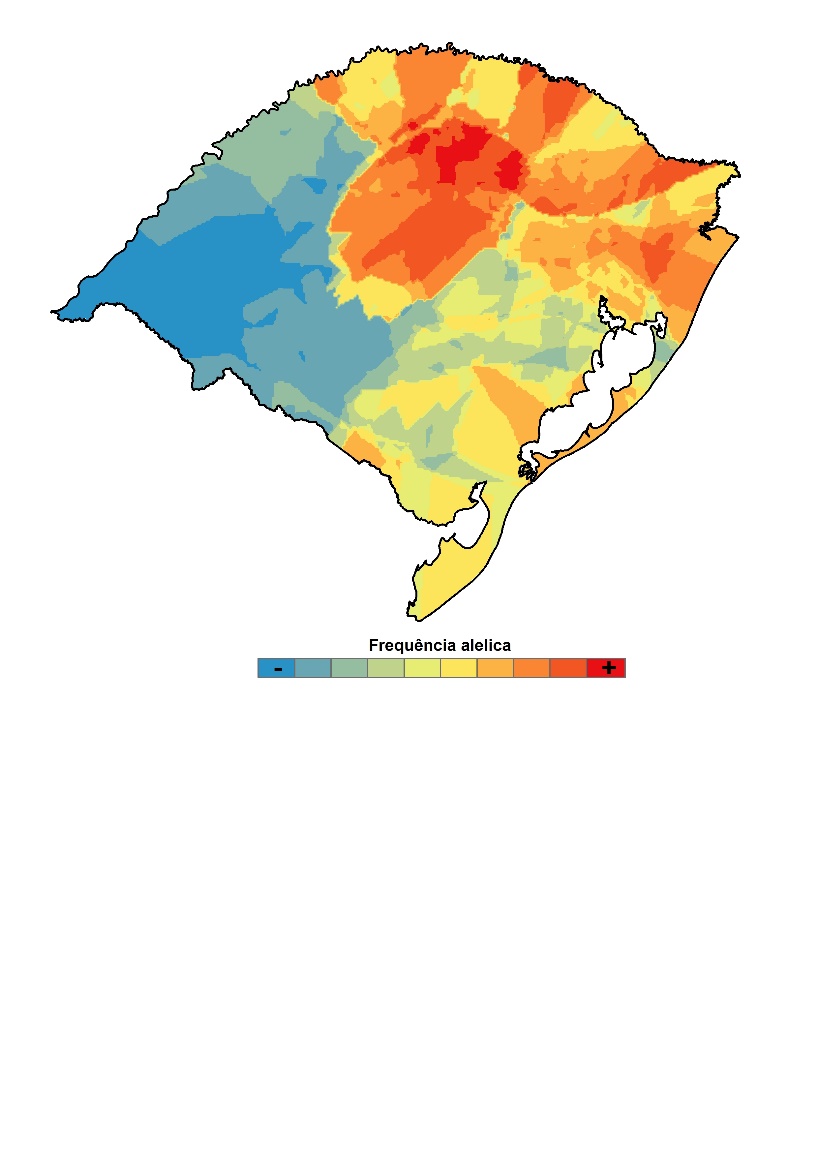


HLA-B*56


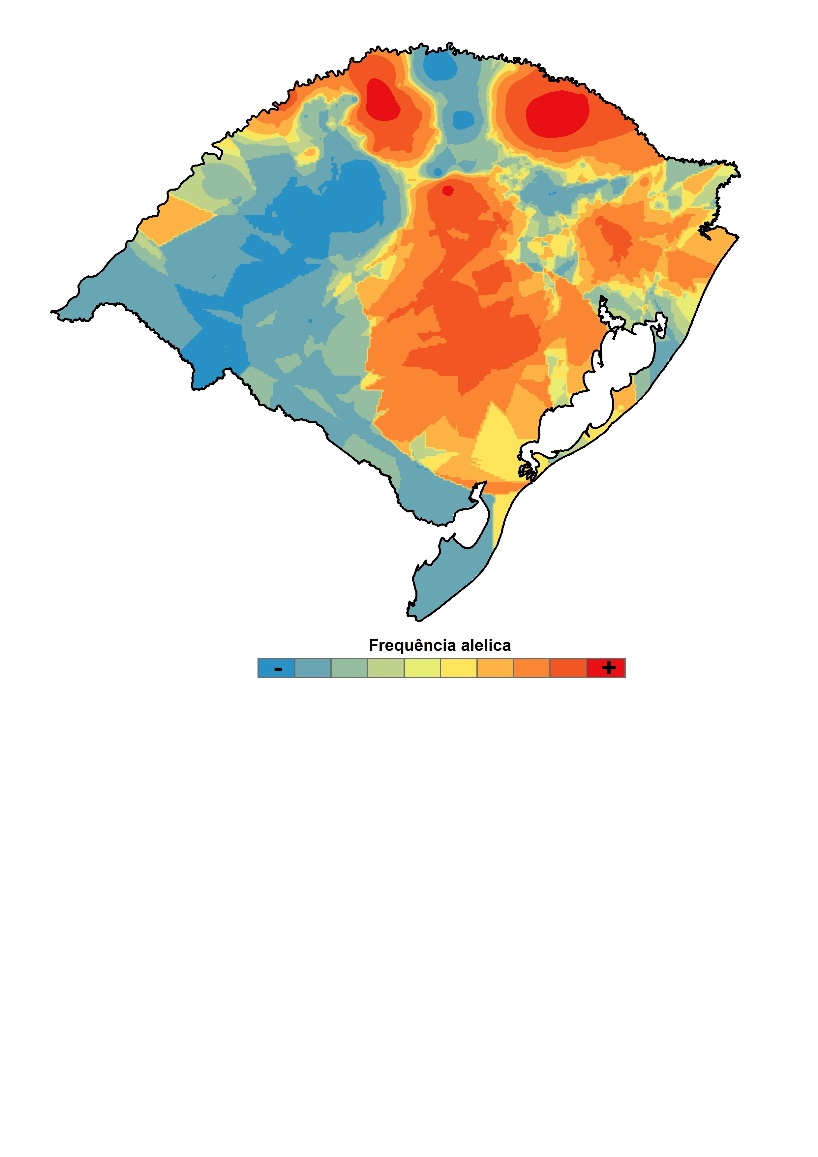


HLA-B*55


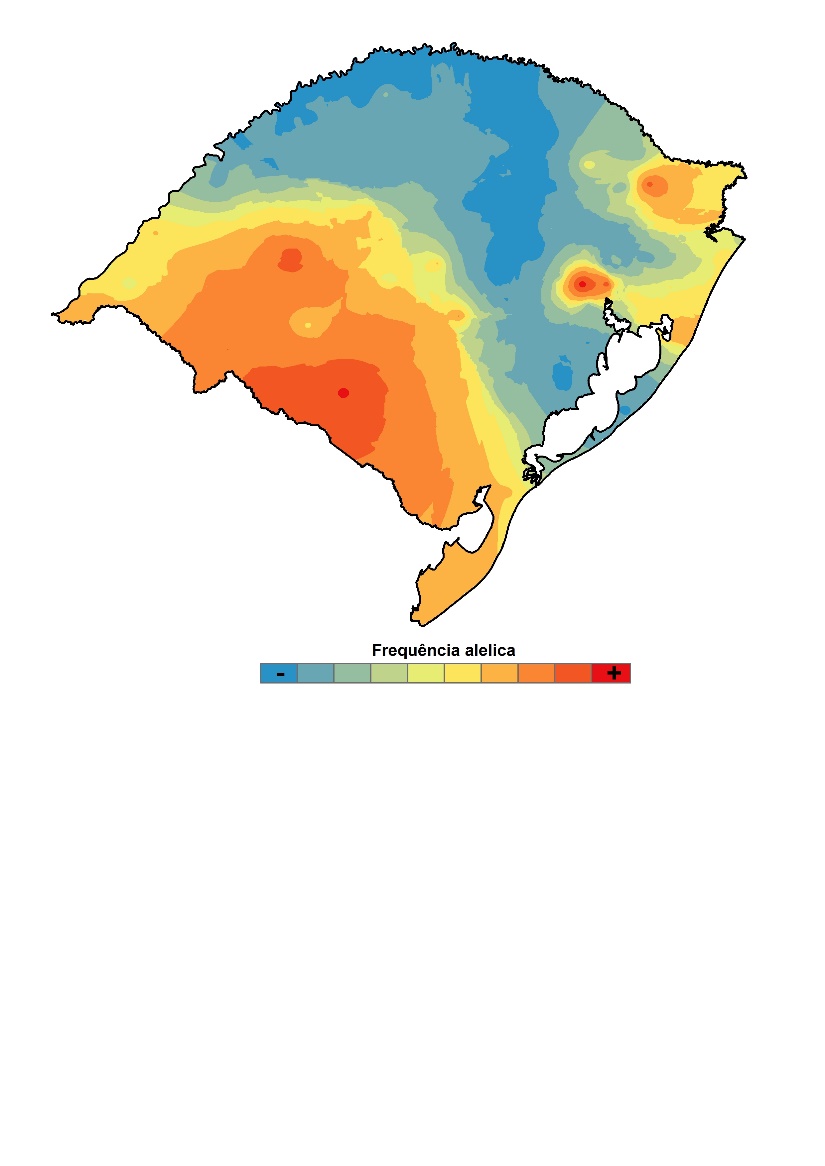


HLA-B*58


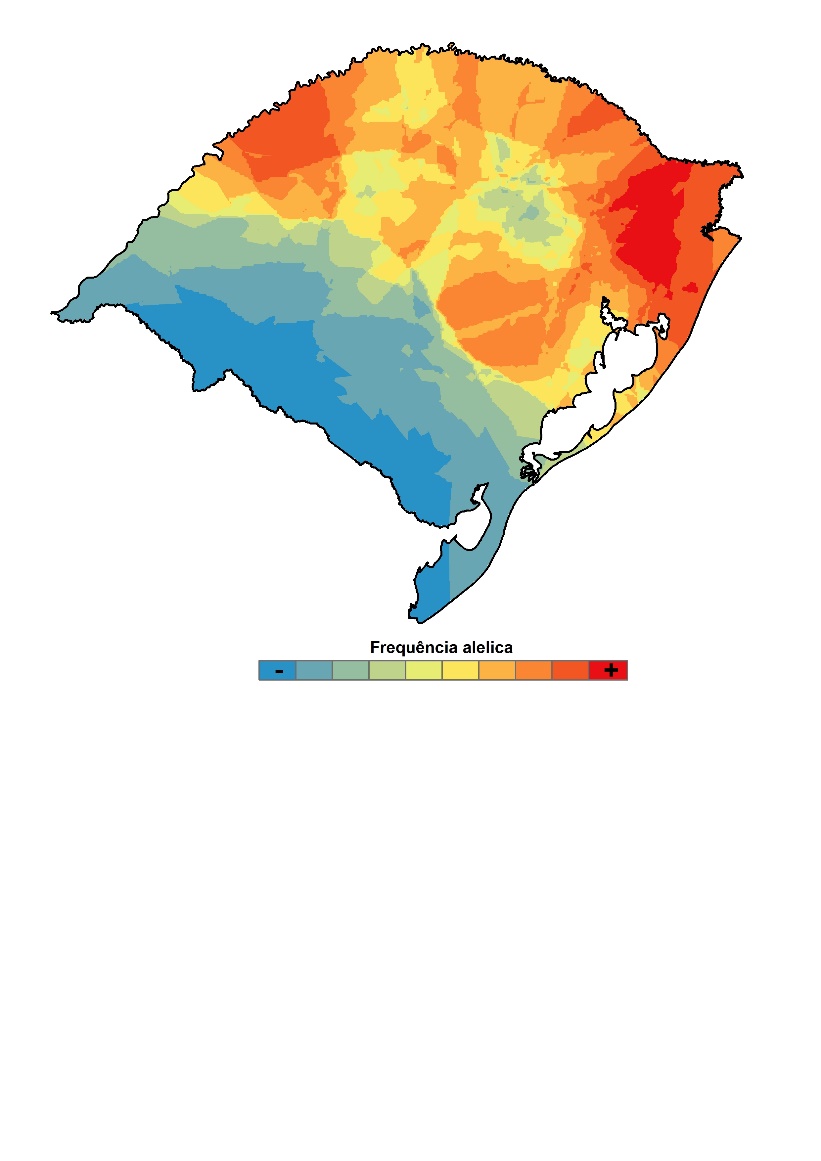


HLA-B*57


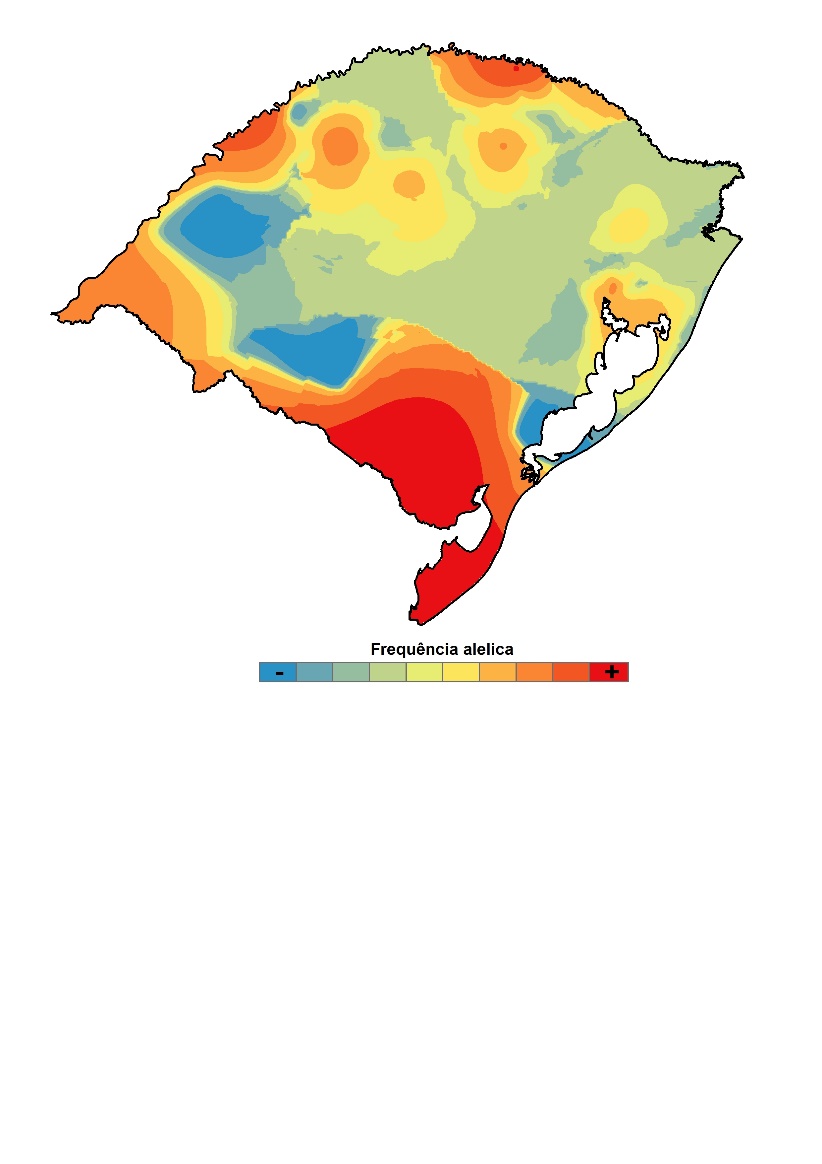


HLA-B*67


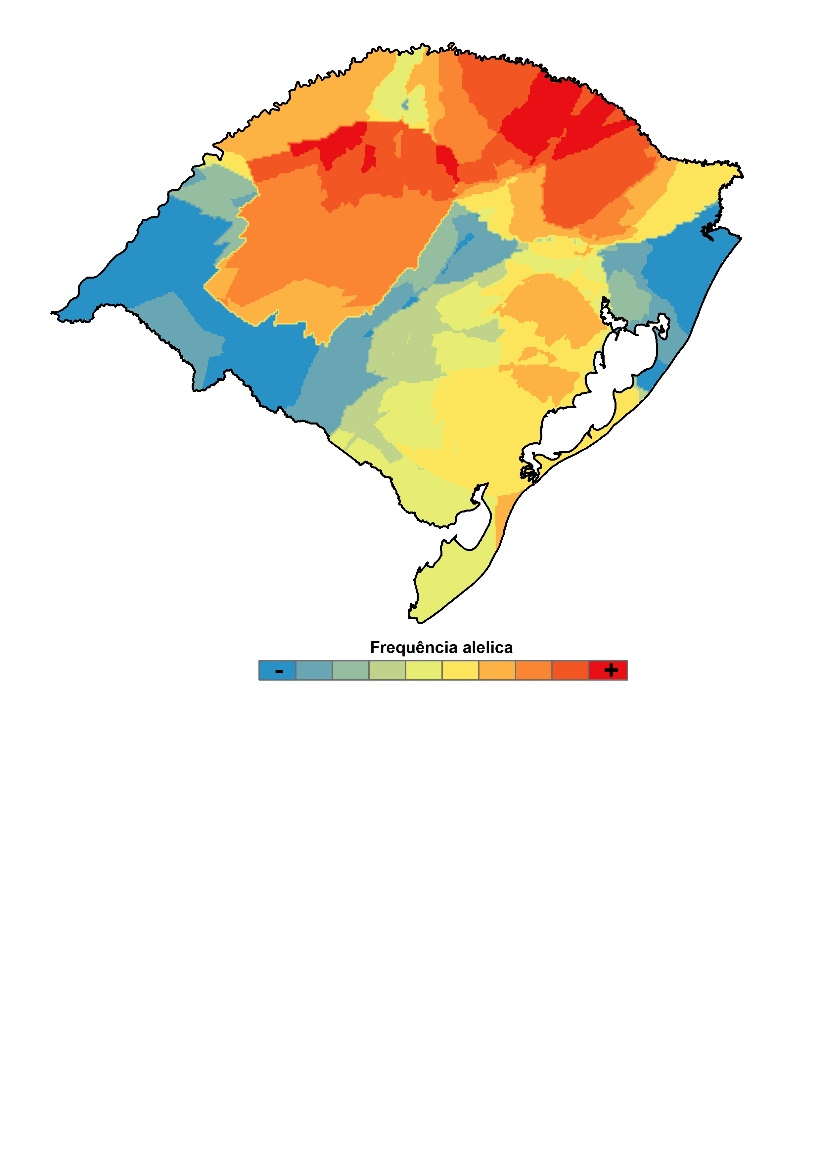


HLA-B*73


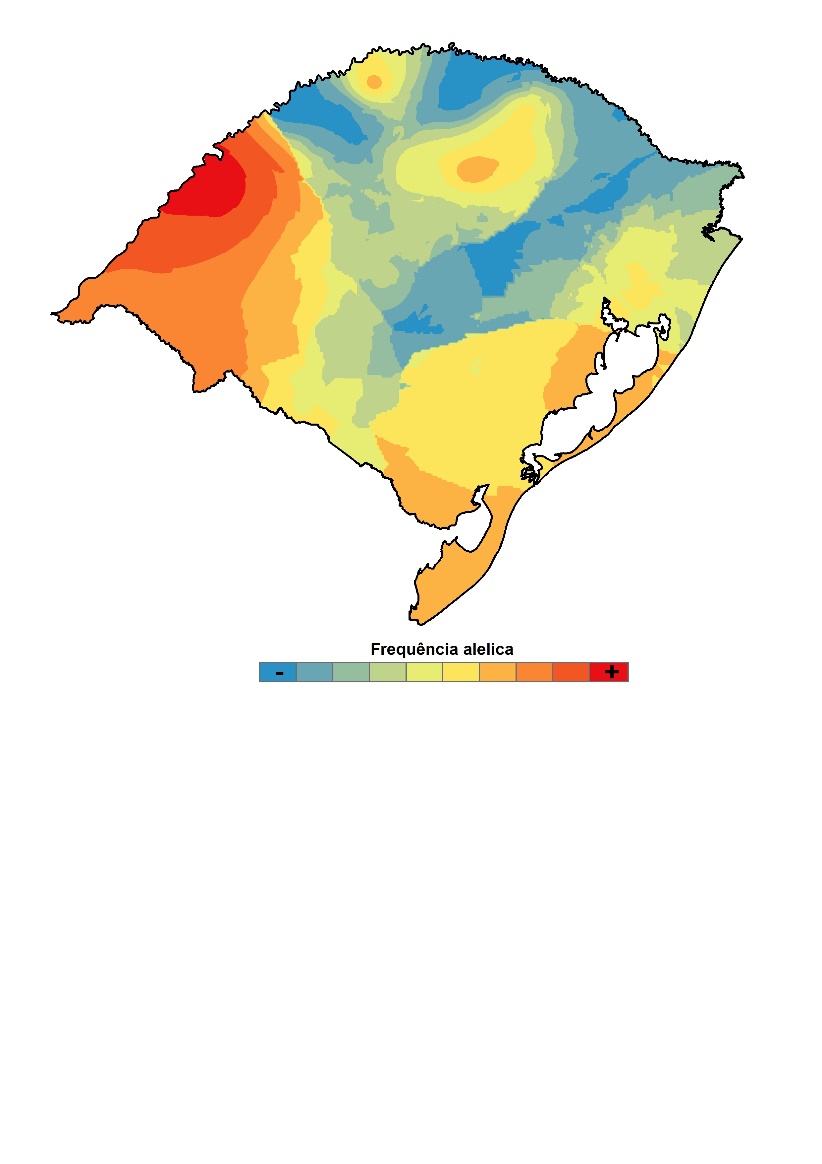


HLA-B*81


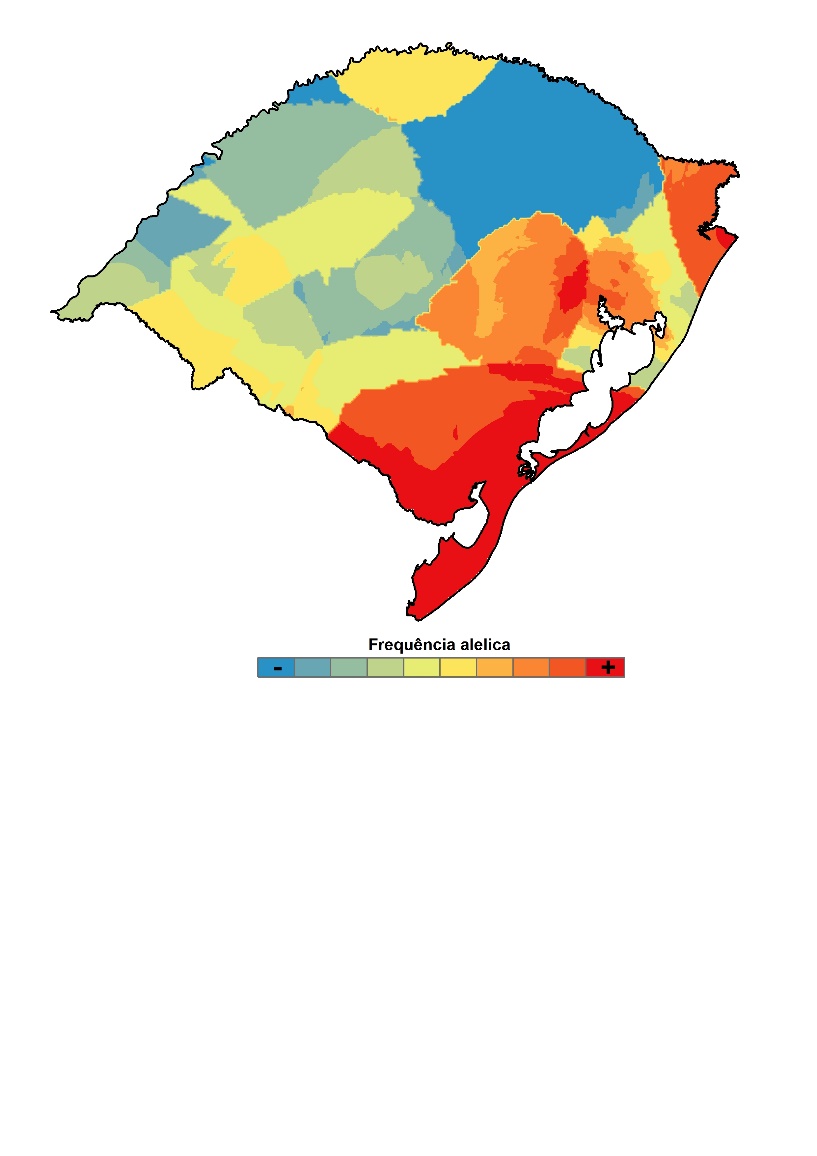


HLA-B*78


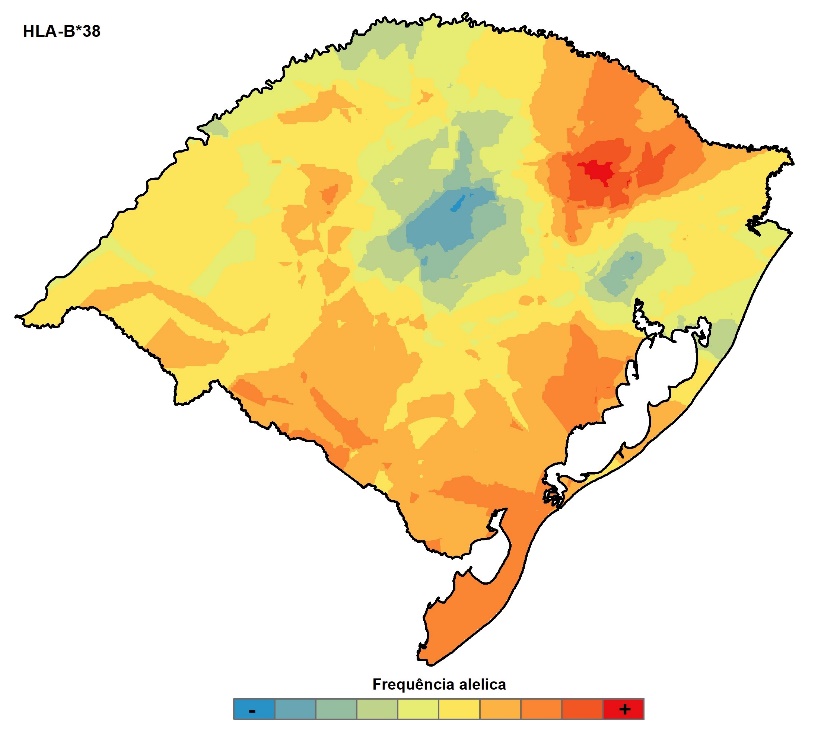


HLA-B*38

**HLA-DRB1**


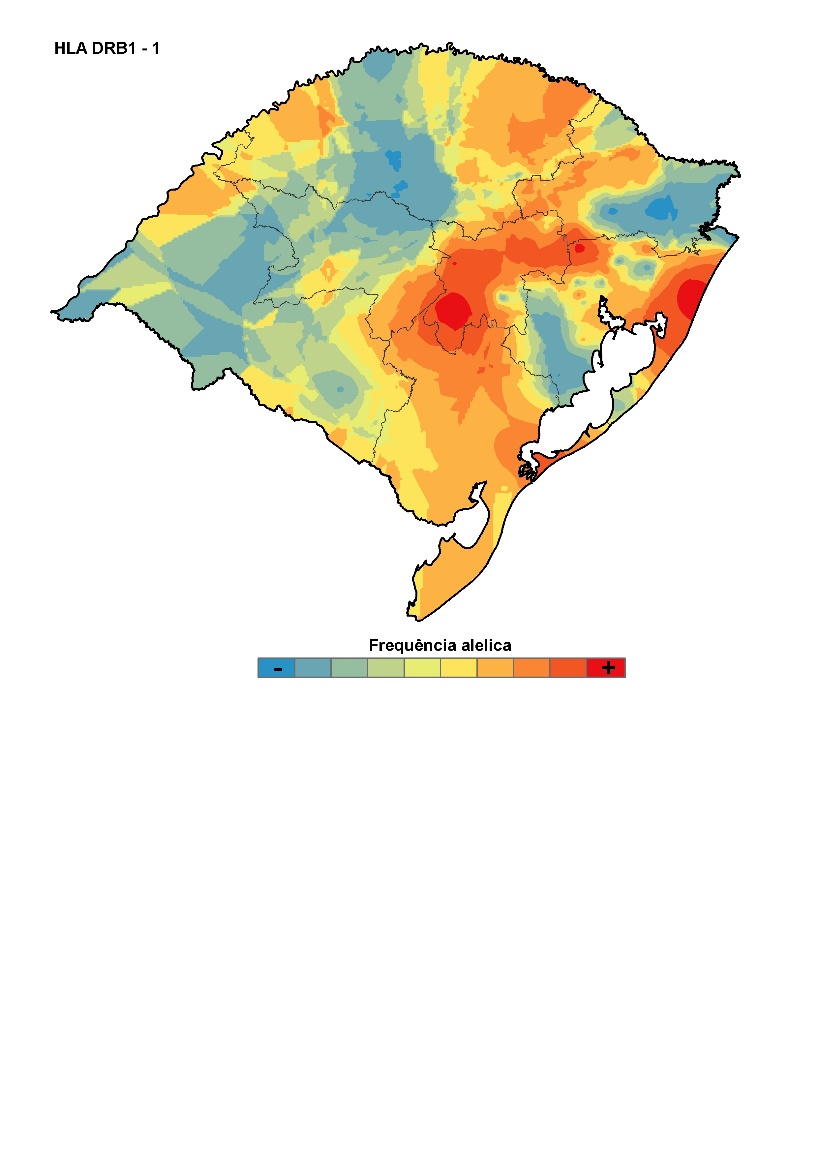


HLA-DRB1*01


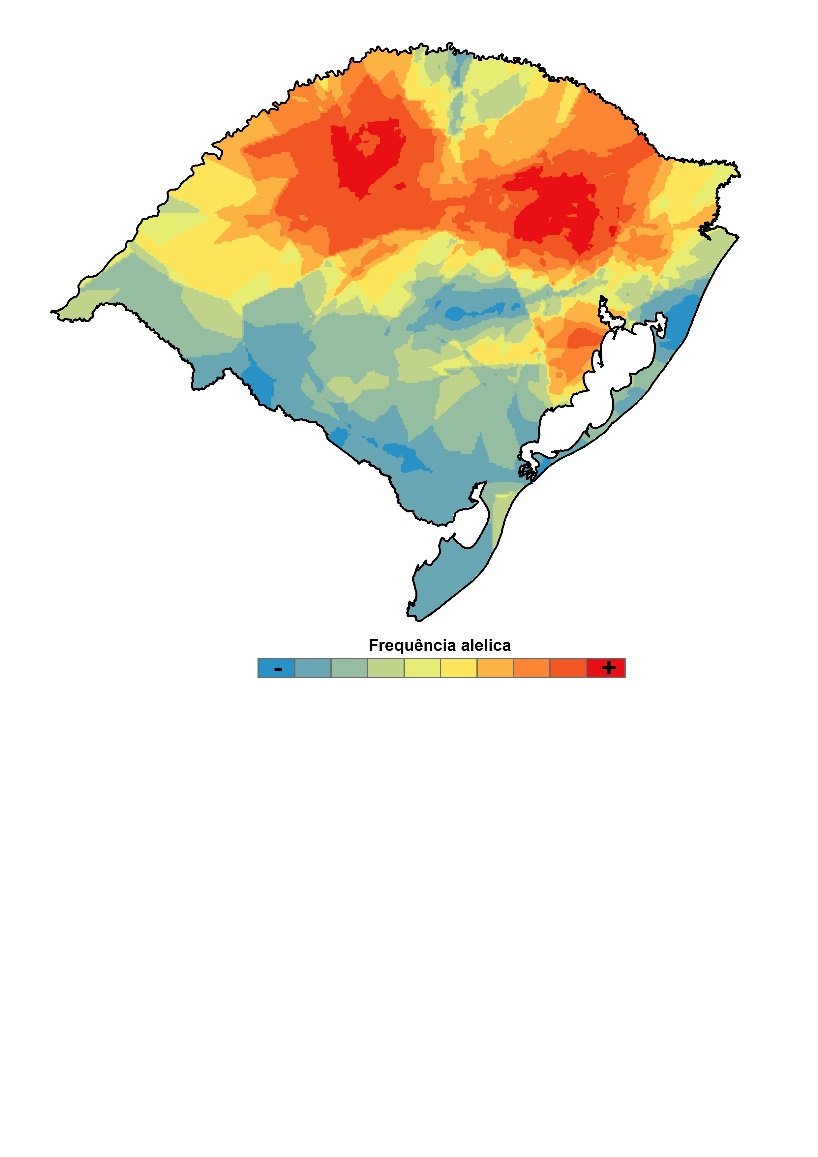


HLA-DRB1*03


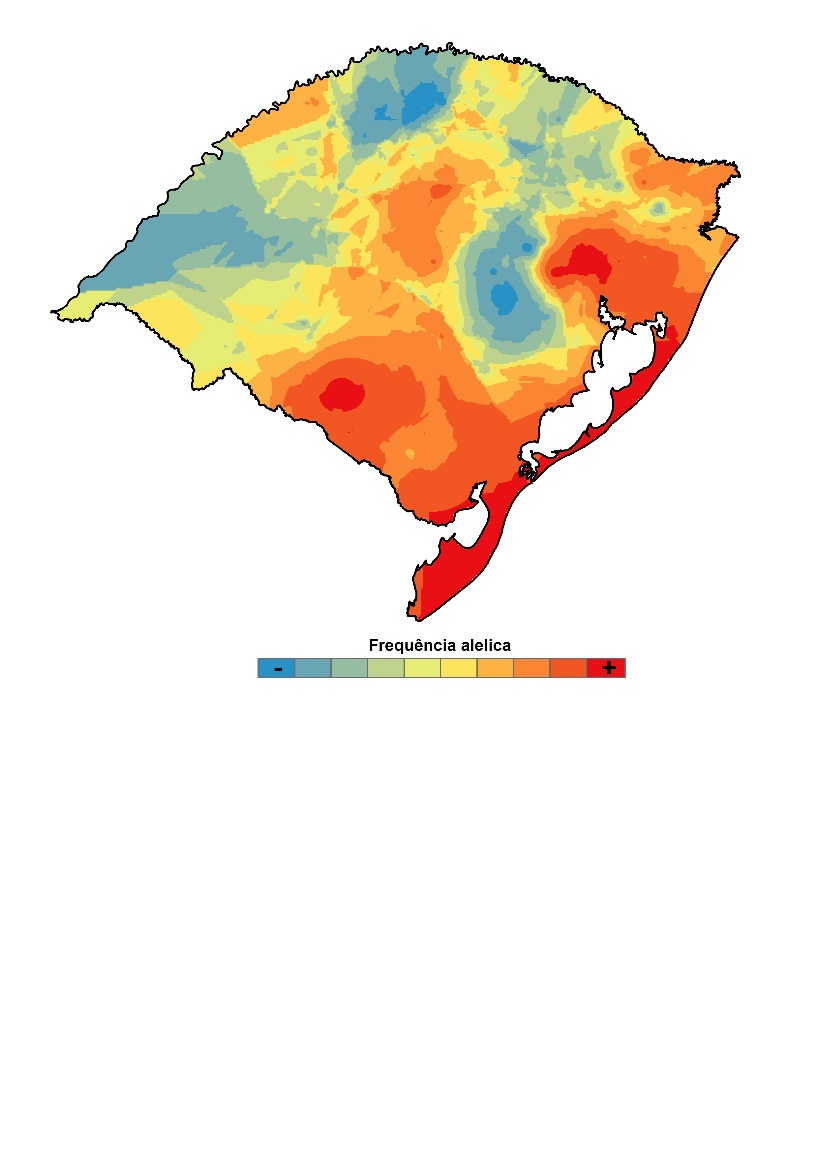


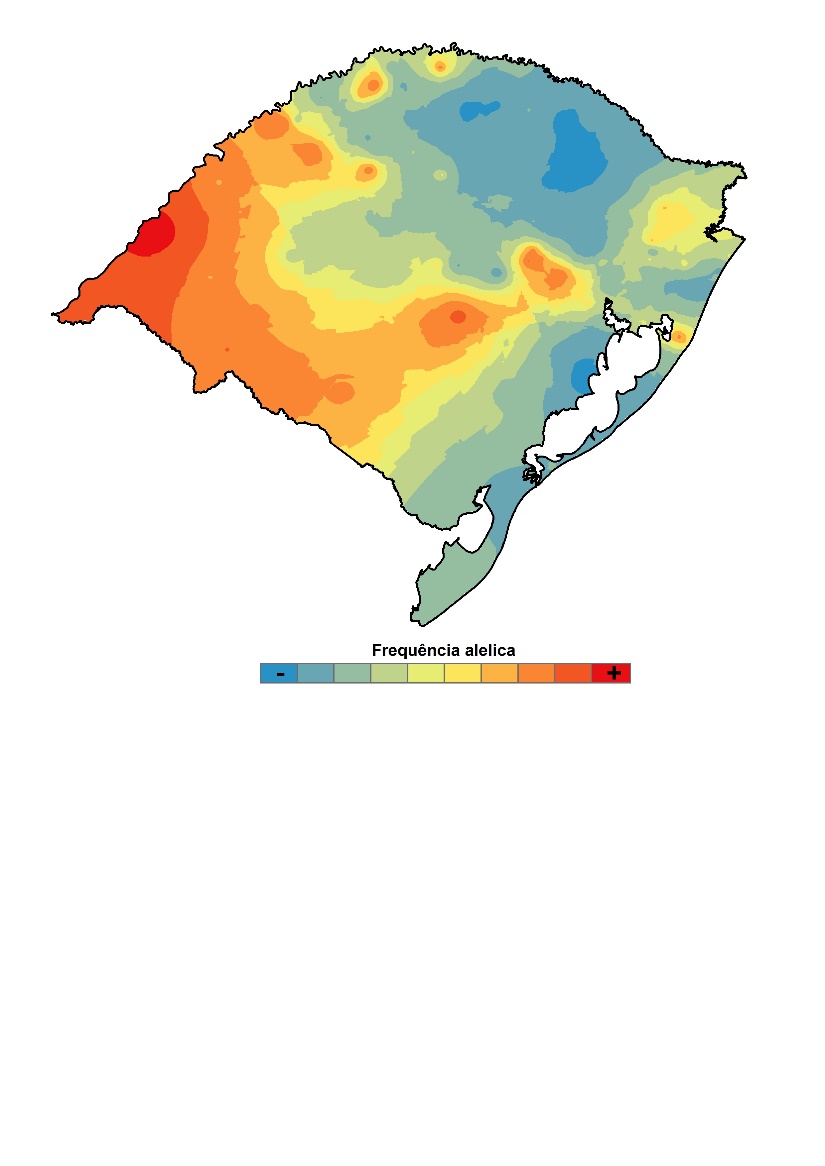


HLA-DRB1*04

HLA-DRB1*07


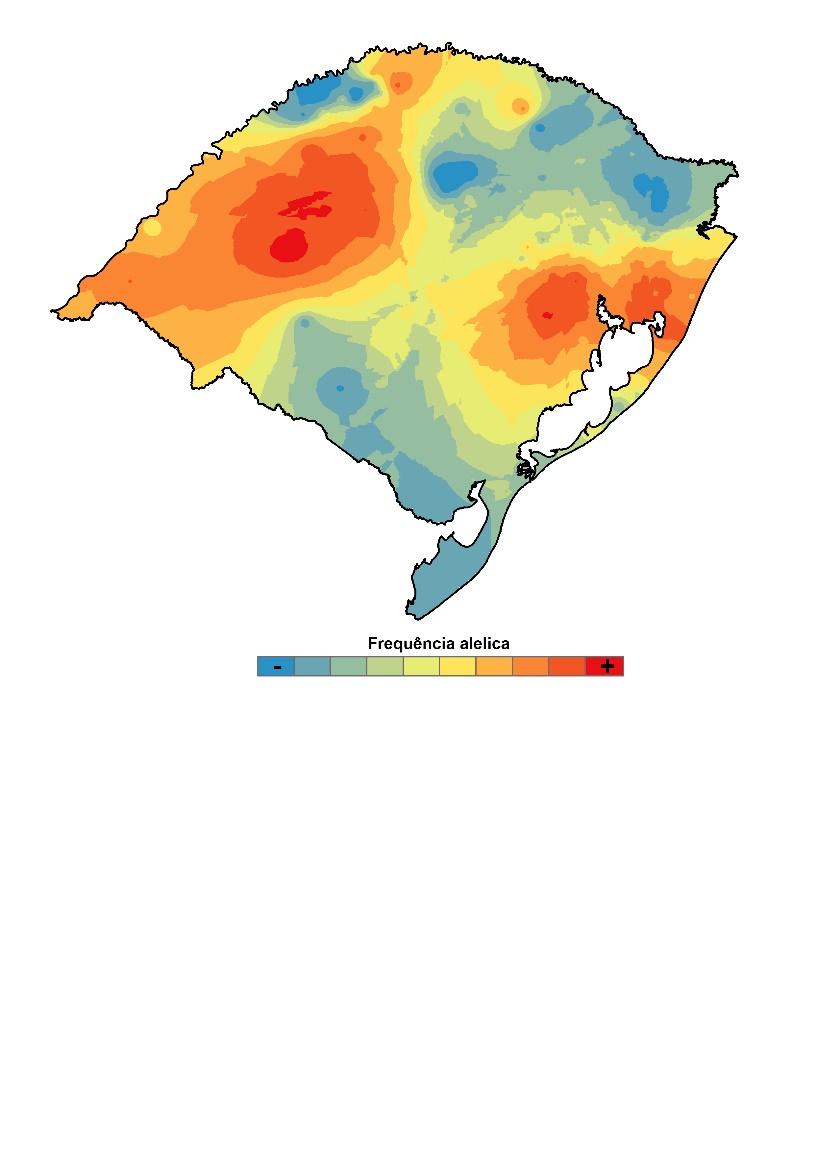


HLA-DRB1*09


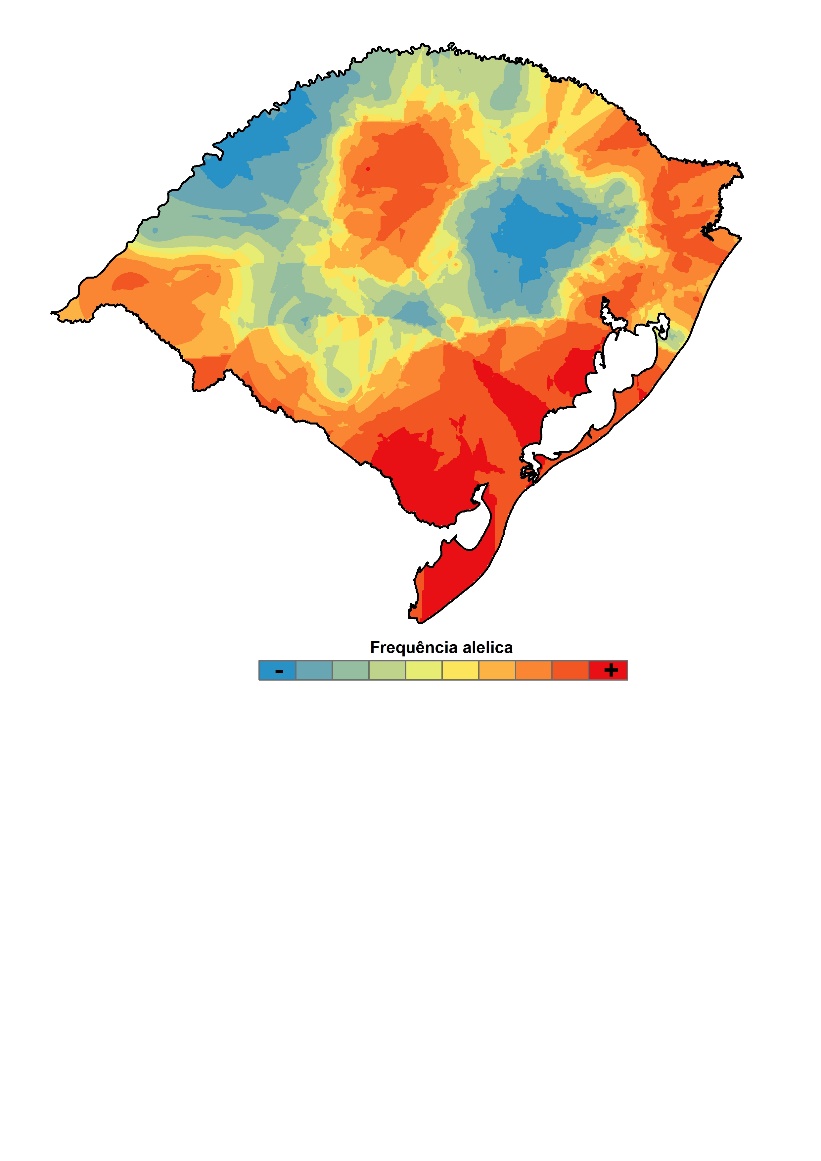


HLA-DRB1*08


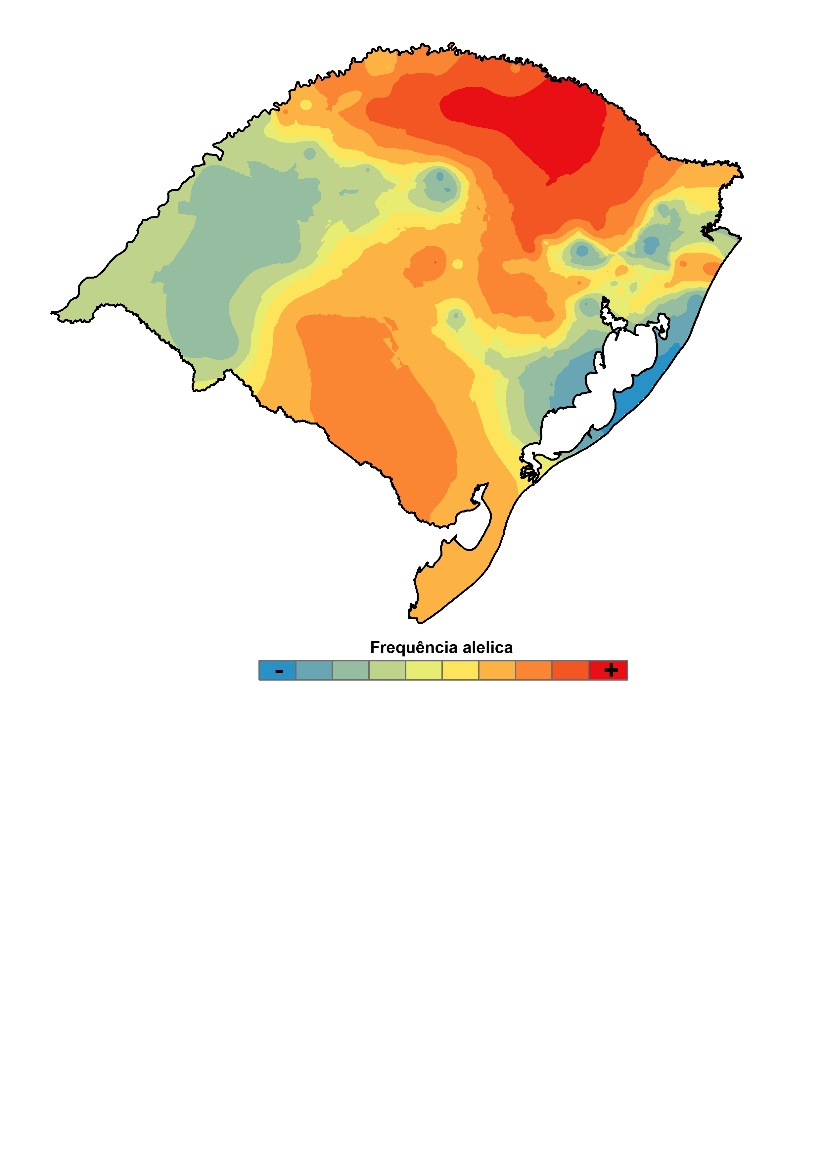


HLA-DRB1*11


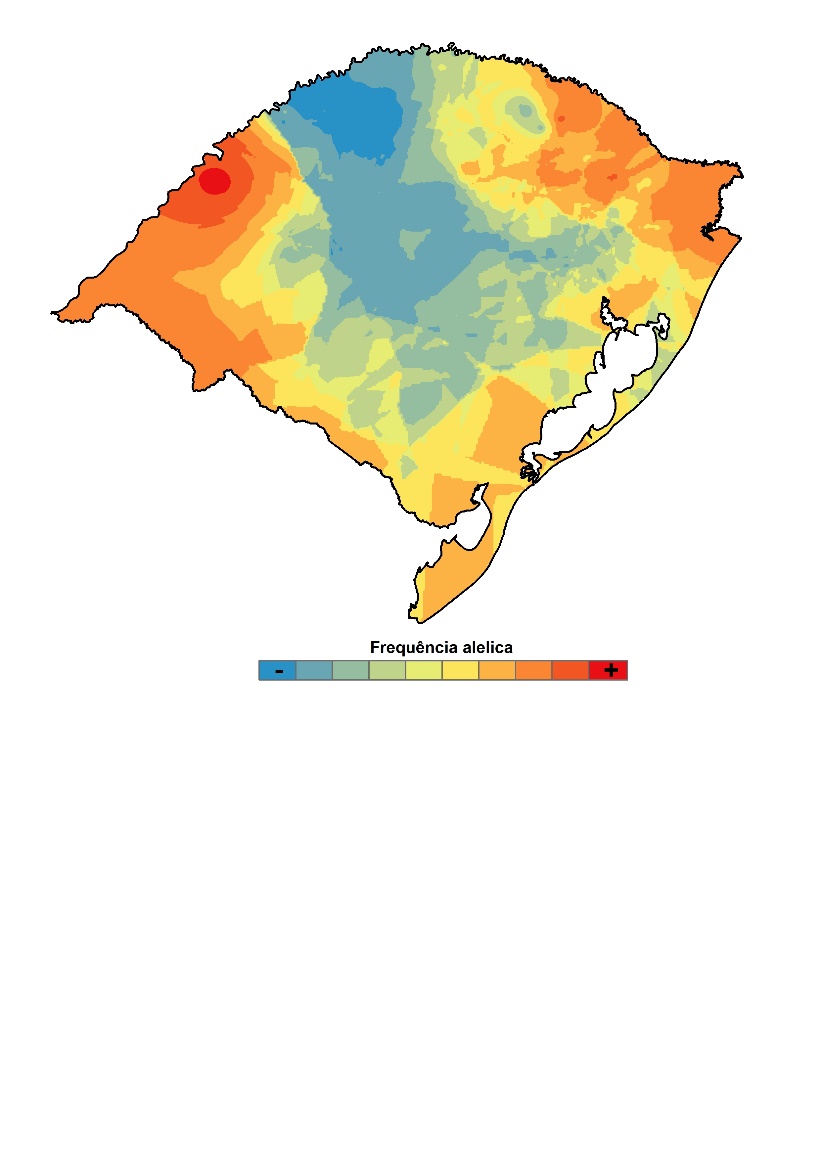


HLA-DRB1*10


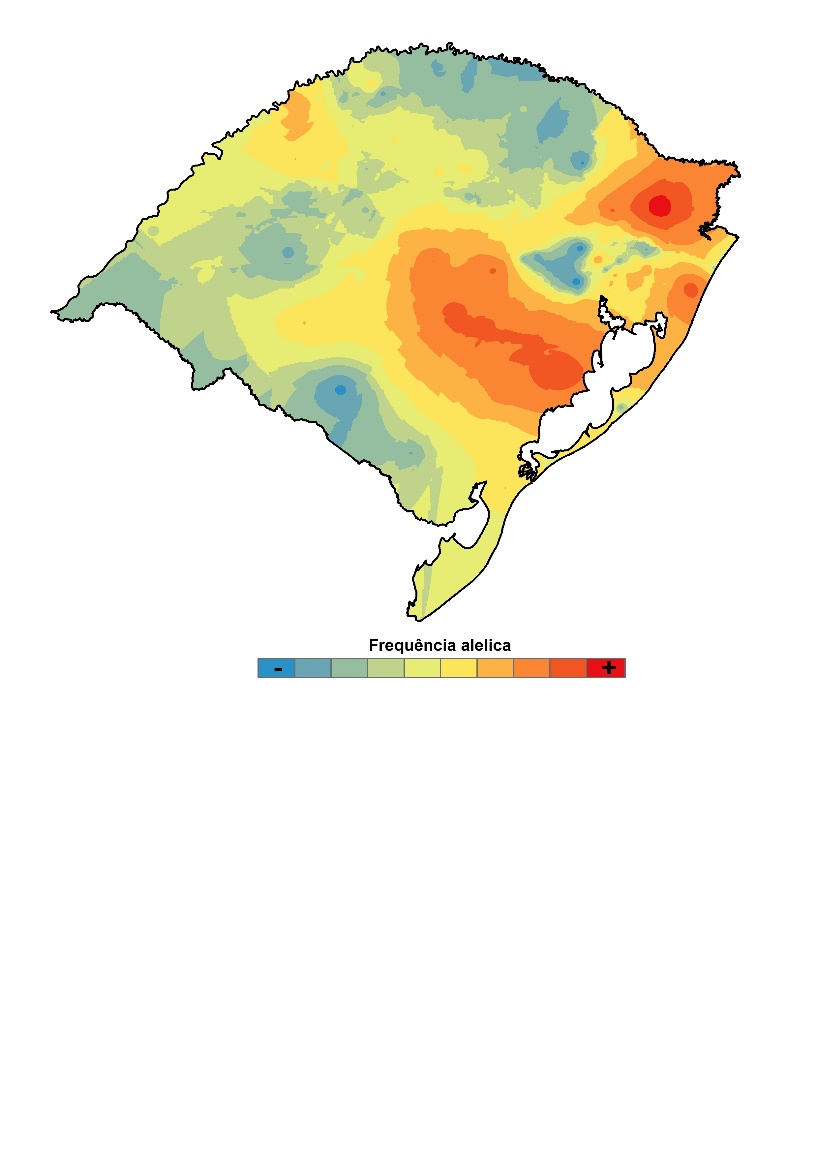


HLA-DRB1*12


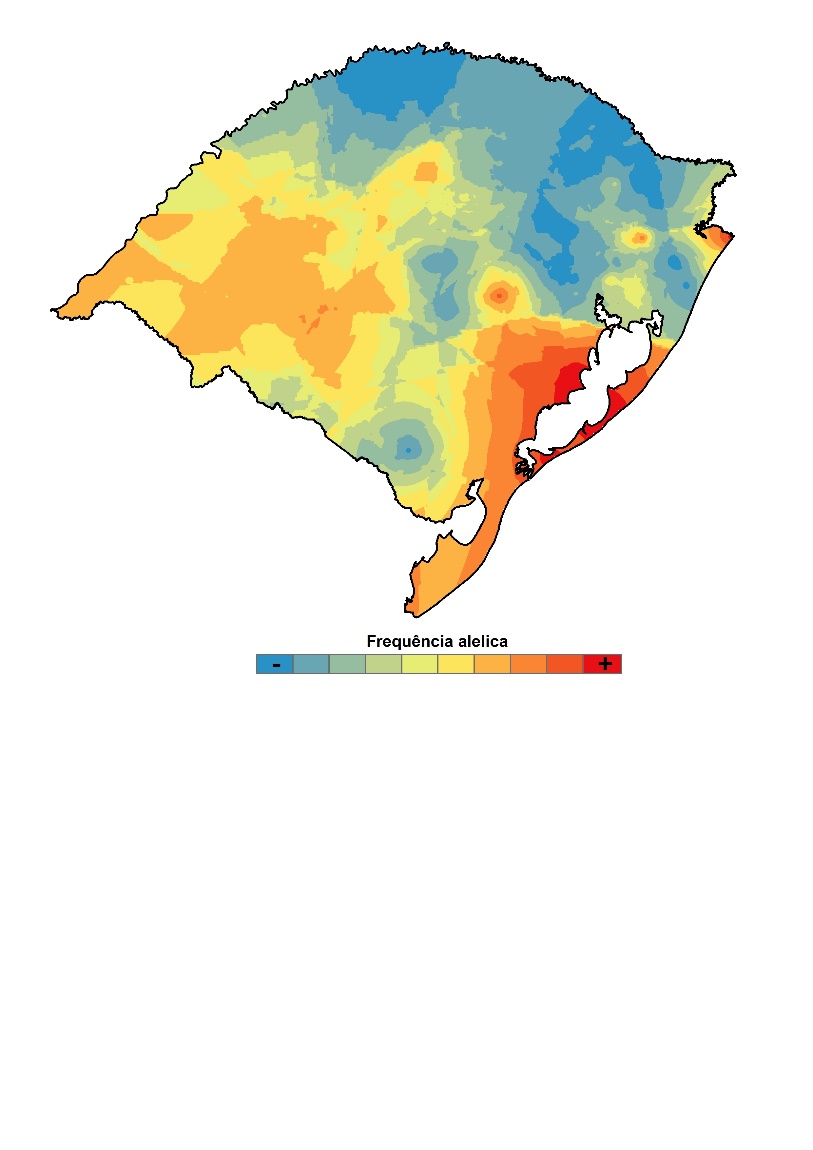


HLA-DRB1*13


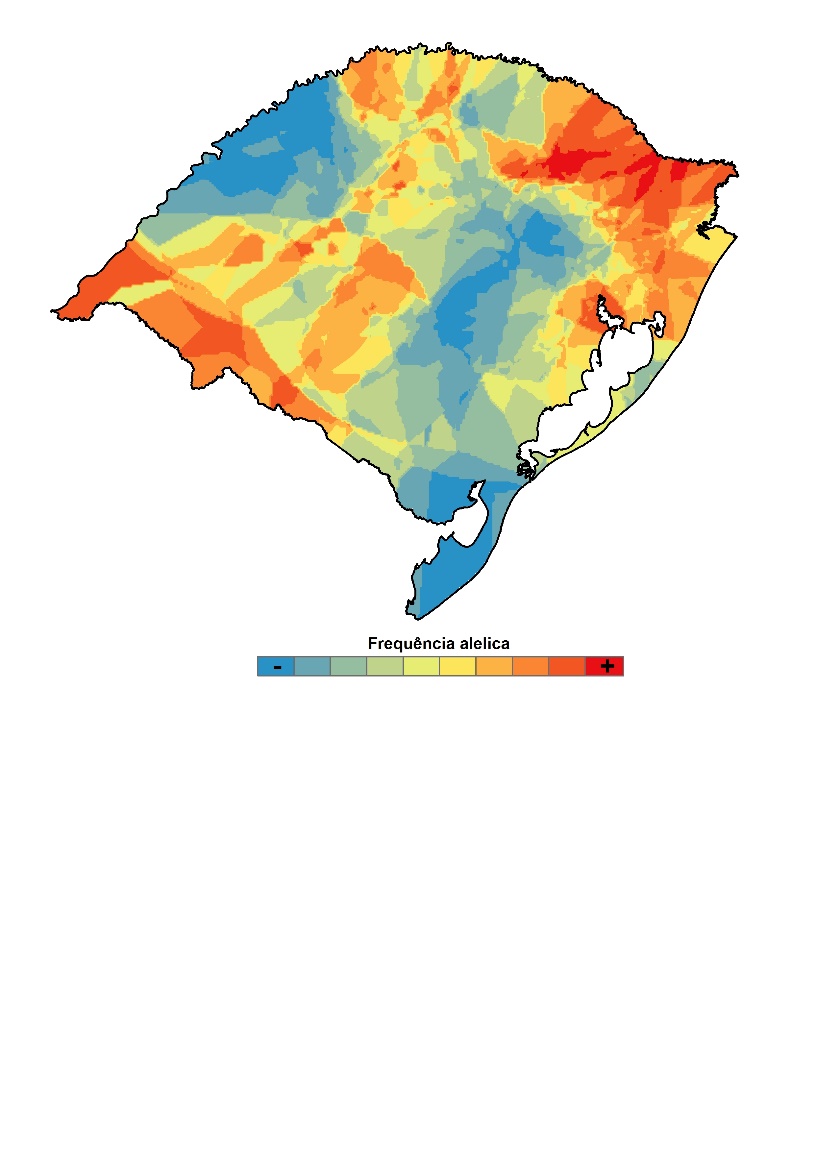


HLA-DRB1*14


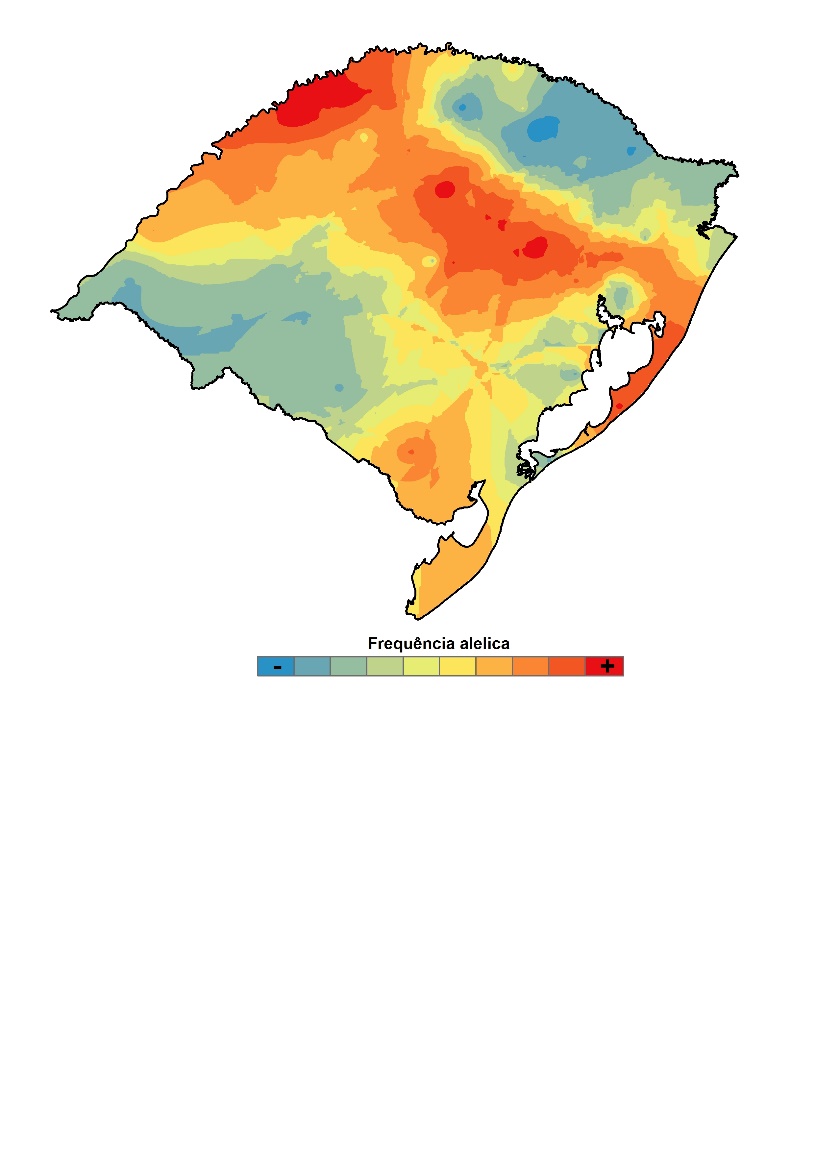


HLA-DRB1*15


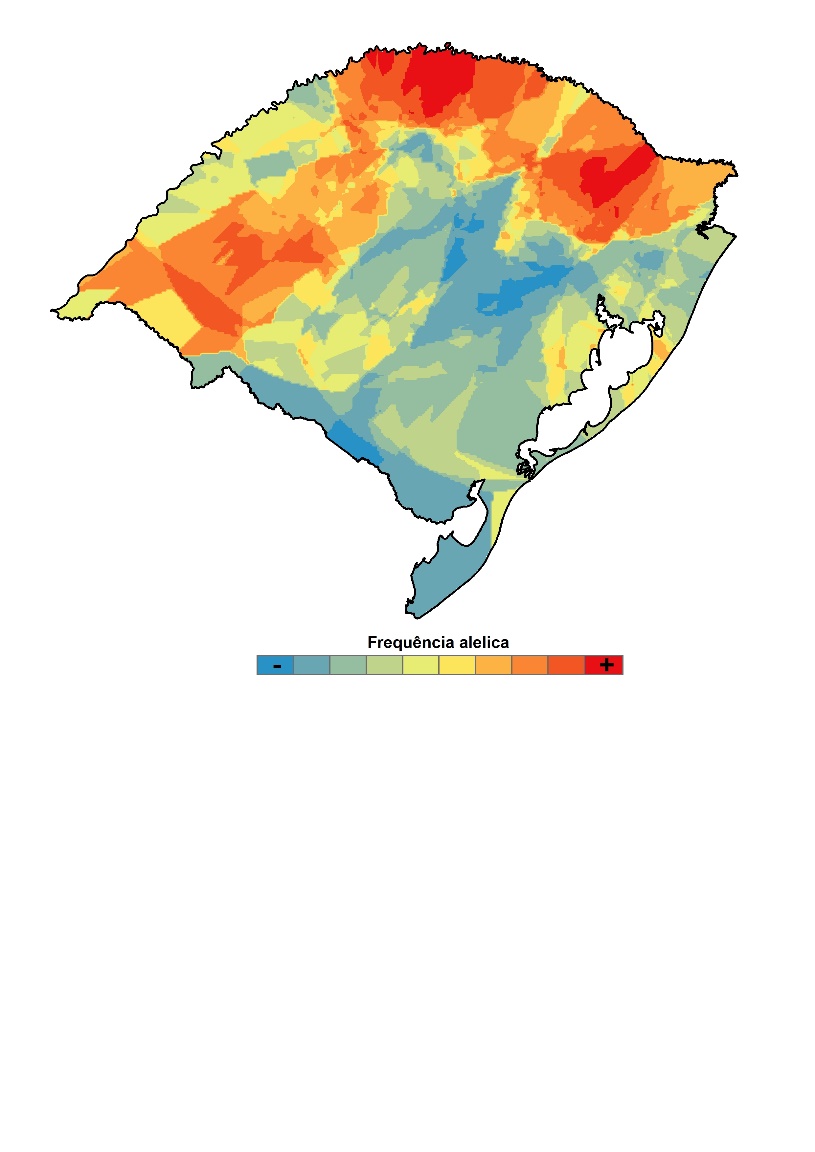


HLA-DRB1*16
