# Supplementary material for: Spatial analyzes of HLA data in Rio Grande do Sul, south Brazil: genetic structure and possible correlation with autoimmune diseases
Source: Int J Health Geogr. 2018 Sep 14;17:34. doi: 10.1186/s12942-018-0154-8 (PMC6137739; doi:10.1186/s12942-018-0154-8)
Supplement: Supplementary file 4 — Additional file 4. Haplotype frequencies. [file 12942_2018_154_MOESM4_ESM.docx]

**Additional file 4 – Haplotype frequencies**

Haplotype frequencies in Rio Grande do Sul cities. Only cities with sample size greater than or equal to 50 were included in the study. Only haplotypes with frequency equal to or greater than 2% in at least one city are listed.

| **Town** | **N** | **A*01~B*08~DRB1*03** | **A*03~B*07~DRB1*15** | **A*29~B*44~DRB1*07** | **A*02~B*07~DRB1*15** | **A*03~B*35~DRB1*01** |
| --- | --- | --- | --- | --- | --- | --- |
| Alegrete | 1259 | 0.026 | 0.010 | 0.017 | 0.011 | 0.006 |
| Alvorada | 841 | 0.033 | 0.011 | 0.022 | 0.010 | 0.012 |
| Aratiba | 130 | 0.053 | 0.015 | 0.023 | 0.015 | 0.027 |
| Arroio Do Sal | 507 | 0.025 | 0.012 | 0.035 | 0.005 | 0.021 |
| Bento Goncalves | 248 | 0.056 | 0.000 | 0.014 | 0.006 | 0.016 |
| Boa Vista Do Burica | 173 | 0.045 | 0.043 | 0.003 | 0.000 | 0.013 |
| Cachoeirinha | 1139 | 0.025 | 0.012 | 0.026 | 0.012 | 0.016 |
| Camaqua | 114 | 0.060 | 0.024 | 0.004 | 0.010 | 0.000 |
| Campo Bom | 612 | 0.033 | 0.017 | 0.014 | 0.015 | 0.014 |
| Campo Novo | 177 | 0.042 | 0.019 | 0.011 | 0.004 | 0.013 |
| Candido Godoi | 172 | 0.061 | 0.031 | 0.003 | 0.011 | 0.017 |
| Canoas | 3820 | 0.032 | 0.014 | 0.020 | 0.011 | 0.011 |
| Capao Da Canoa | 104 | 0.038 | 0.000 | 0.019 | 0.024 | 0.016 |
| Carazinho | 314 | 0.052 | 0.011 | 0.024 | 0.020 | 0.009 |
| Caxias Do Sul | 4959 | 0.034 | 0.014 | 0.016 | 0.011 | 0.012 |
| Charqueadas | 107 | 0.023 | 0.000 | 0.028 | 0.000 | 0.009 |
| Crissiumal | 277 | 0.050 | 0.026 | 0.013 | 0.021 | 0.019 |
| Cruz Alta | 1977 | 0.025 | 0.016 | 0.020 | 0.013 | 0.011 |
| Dois Irmaos | 163 | 0.064 | 0.030 | 0.009 | 0.025 | 0.009 |
| Doutor Mauricio Cardoso | 172 | 0.021 | 0.022 | 0.015 | 0.003 | 0.003 |
| Eldorado Do Sul | 175 | 0.047 | 0.014 | 0.017 | 0.014 | 0.003 |
| Encantado | 214 | 0.062 | 0.013 | 0.020 | 0.003 | 0.013 |
| Erechim | 663 | 0.037 | 0.017 | 0.023 | 0.009 | 0.020 |
| Espumoso | 107 | 0.045 | 0.000 | 0.017 | 0.005 | 0.000 |
| Estancia Velha | 883 | 0.042 | 0.020 | 0.018 | 0.014 | 0.008 |
| Esteio | 654 | 0.030 | 0.011 | 0.018 | 0.006 | 0.021 |
| Estrela | 799 | 0.038 | 0.031 | 0.012 | 0.019 | 0.013 |
| Farroupilha | 2347 | 0.033 | 0.015 | 0.015 | 0.007 | 0.009 |
| Gravatai | 3217 | 0.029 | 0.017 | 0.019 | 0.008 | 0.011 |
| Guaiba | 469 | 0.027 | 0.012 | 0.018 | 0.010 | 0.016 |
| Igrejinha | 279 | 0.045 | 0.023 | 0.014 | 0.021 | 0.016 |
| Ijui | 561 | 0.038 | 0.015 | 0.017 | 0.012 | 0.007 |
| Itaqui | 142 | 0.014 | 0.006 | 0.025 | 0.000 | 0.000 |
| **Town** | **N** | **A*01~B*08~DRB1*03** | **A*03~B*07~DRB1*15** | **A*29~B*44~DRB1*07** | **A*02~B*07~DRB1*15** | **A*03~B*35~DRB1*01** |
| Ivoti | 325 | 0.040 | 0.023 | 0.018 | 0.027 | 0.016 |
| Jacutinga | 154 | 0.042 | 0.013 | 0.026 | 0.021 | 0.000 |
| Lajeado | 1484 | 0.050 | 0.033 | 0.016 | 0.012 | 0.012 |
| Marau | 188 | 0.030 | 0.010 | 0.023 | 0.000 | 0.013 |
| Marcelino Ramos | 113 | 0.026 | 0.013 | 0.018 | 0.009 | 0.000 |
| Montenegro | 504 | 0.036 | 0.019 | 0.013 | 0.010 | 0.007 |
| Nova Hartz | 1566 | 0.034 | 0.016 | 0.017 | 0.022 | 0.011 |
| Nova Santa Rita | 176 | 0.025 | 0.015 | 0.020 | 0.000 | 0.009 |
| Novo Hamburgo | 4997 | 0.035 | 0.016 | 0.015 | 0.012 | 0.012 |
| Palmeira Das Missoes | 1005 | 0.039 | 0.013 | 0.013 | 0.011 | 0.007 |
| Panambi | 353 | 0.051 | 0.026 | 0.013 | 0.022 | 0.011 |
| Parobe | 394 | 0.022 | 0.015 | 0.011 | 0.007 | 0.011 |
| Passo Fundo | 2113 | 0.032 | 0.015 | 0.017 | 0.007 | 0.009 |
| Pelotas | 4143 | 0.031 | 0.012 | 0.018 | 0.010 | 0.014 |
| Portao | 206 | 0.031 | 0.019 | 0.007 | 0.005 | 0.010 |
| Porto Alegre | 21392 | 0.028 | 0.014 | 0.018 | 0.011 | 0.010 |
| Porto Lucena | 192 | 0.059 | 0.008 | 0.018 | 0.014 | 0.008 |
| Porto Xavier | 186 | 0.027 | 0.031 | 0.013 | 0.010 | 0.008 |
| Redentora | 106 | 0.038 | 0.019 | 0.009 | 0.024 | 0.000 |
| Rio Grande | 383 | 0.038 | 0.008 | 0.018 | 0.020 | 0.015 |
| Rolante | 103 | 0.019 | 0.049 | 0.006 | 0.006 | 0.024 |
| Sananduva | 110 | 0.032 | 0.000 | 0.000 | 0.009 | 0.009 |
| Santa Cruz Do Sul | 959 | 0.044 | 0.023 | 0.018 | 0.016 | 0.013 |
| Santa Maria | 1740 | 0.034 | 0.015 | 0.014 | 0.010 | 0.011 |
| Santa Rosa | 1916 | 0.046 | 0.020 | 0.011 | 0.010 | 0.011 |
| Santana Do Livramento | 500 | 0.025 | 0.011 | 0.014 | 0.015 | 0.013 |
| Santo Angelo | 468 | 0.032 | 0.019 | 0.012 | 0.003 | 0.003 |
| Santo Antonio Da Patrulha | 337 | 0.031 | 0.021 | 0.024 | 0.027 | 0.012 |
| Santo Cristo | 144 | 0.030 | 0.040 | 0.017 | 0.026 | 0.000 |
| Sao Jose Do Inhacora | 147 | 0.069 | 0.042 | 0.003 | 0.004 | 0.020 |
| Sao Jose Do Norte | 159 | 0.016 | 0.016 | 0.021 | 0.006 | 0.022 |
| Sao Leopoldo | 1780 | 0.030 | 0.012 | 0.016 | 0.013 | 0.016 |
| Sao Luiz Gonzaga | 291 | 0.048 | 0.011 | 0.028 | 0.023 | 0.020 |
| **Town** | **N** | **A*01~B*08~DRB1*03** | **A*03~B*07~DRB1*15** | **A*29~B*44~DRB1*07** | **A*02~B*07~DRB1*15** | **A*03~B*35~DRB1*01** |
| Sao Paulo Das Missoes | 144 | 0.052 | 0.048 | 0.007 | 0.021 | 0.010 |
| Sapiranga | 1289 | 0.040 | 0.013 | 0.014 | 0.018 | 0.015 |
| Sapucaia Do Sul | 955 | 0.029 | 0.014 | 0.014 | 0.014 | 0.014 |
| Sede Nova | 135 | 0.037 | 0.033 | 0.004 | 0.012 | 0.025 |
| Taquara | 375 | 0.030 | 0.018 | 0.012 | 0.018 | 0.012 |
| Taquari | 101 | 0.030 | 0.015 | 0.015 | 0.000 | 0.005 |
| Tenente Portela | 356 | 0.048 | 0.021 | 0.010 | 0.015 | 0.009 |
| Tres De Maio | 197 | 0.046 | 0.020 | 0.015 | 0.019 | 0.000 |
| Tres Passos | 326 | 0.050 | 0.019 | 0.014 | 0.021 | 0.000 |
| Tuparendi | 243 | 0.040 | 0.020 | 0.012 | 0.002 | 0.008 |
| Uruguaiana | 1346 | 0.026 | 0.010 | 0.021 | 0.010 | 0.005 |
| Vacaria | 614 | 0.030 | 0.014 | 0.017 | 0.011 | 0.021 |
| Viamao | 1327 | 0.029 | 0.014 | 0.014 | 0.010 | 0.011 |
