# Supplementary material for: Spatial analyzes of HLA data in Rio Grande do Sul, south Brazil: genetic structure and possible correlation with autoimmune diseases
Source: Int J Health Geogr. 2018 Sep 14;17:34. doi: 10.1186/s12942-018-0154-8 (PMC6137739; doi:10.1186/s12942-018-0154-8)
Supplement: Supplementary file 5 — Additional file 5. Haplotype frequencies maps. [file 12942_2018_154_MOESM5_ESM.docx]

**Additional file 5 – Haplotype frequencies maps**

Only haplotypes with frequency greater than or equal to 2% in at least one city are shown


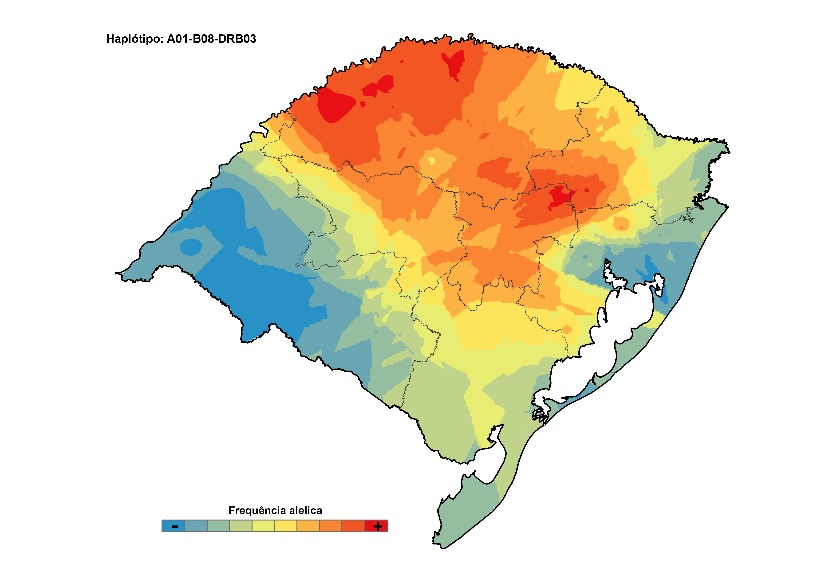


HLA-A*01~B*08~DRB1~03


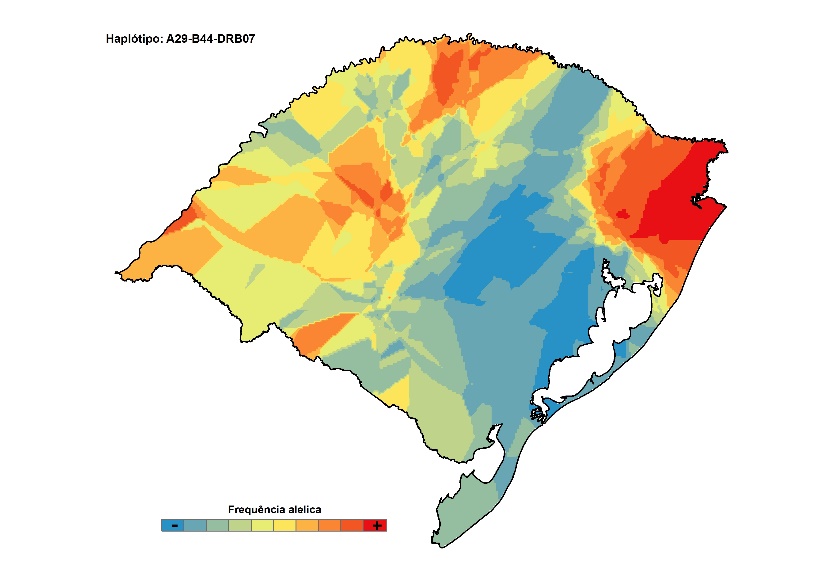


HLA-A*02~B*07~DRB1~15


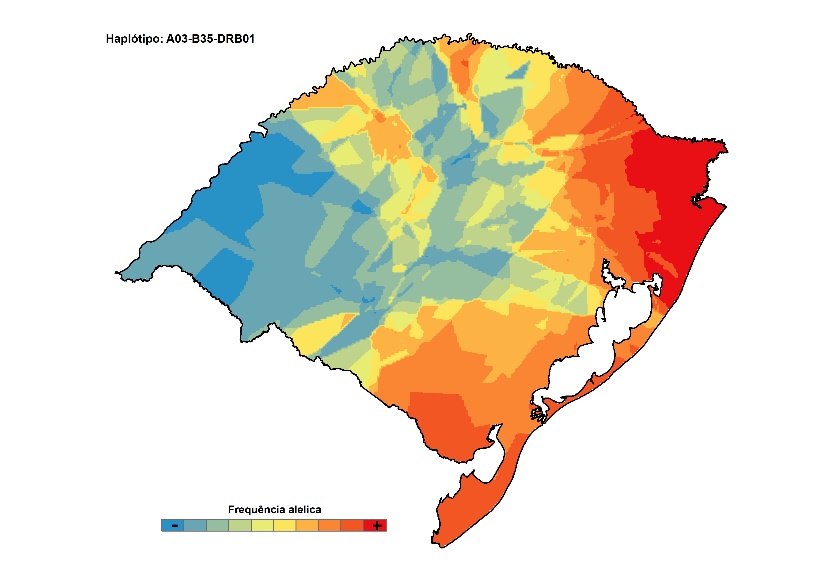


HLA-A*03~B*35~DRB1~01


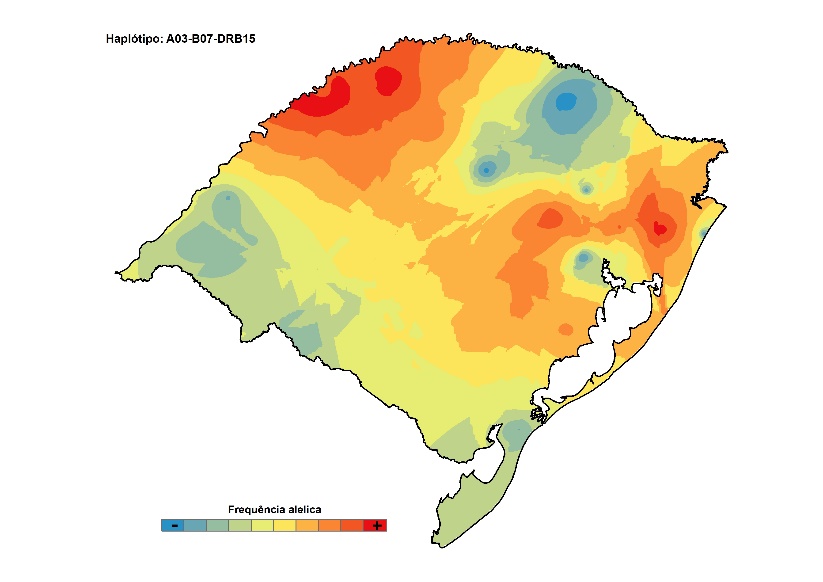


HLA-A*03~B*07~DRB1~15


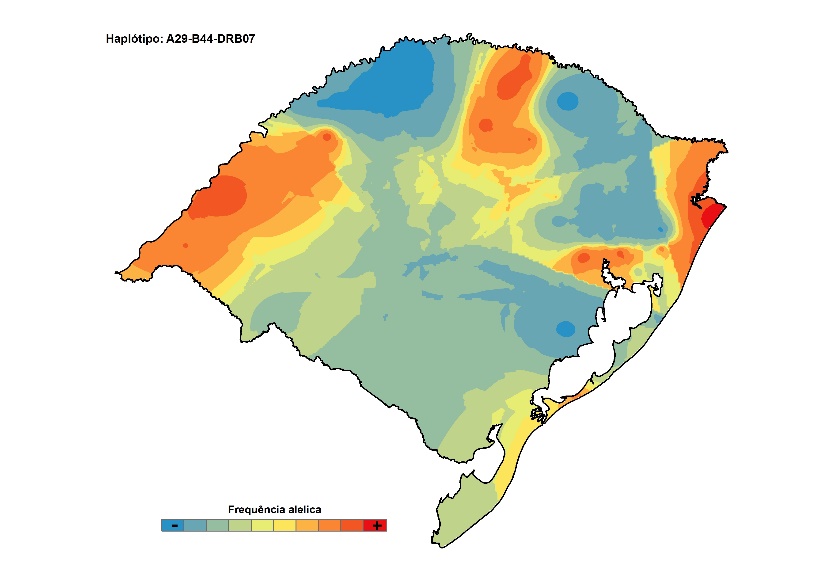


HLA-A*29~B*44~DRB1~07
