# Supplementary material for: Spatial analyzes of HLA data in Rio Grande do Sul, south Brazil: genetic structure and possible correlation with autoimmune diseases
Source: Int J Health Geogr. 2018 Sep 14;17:34. doi: 10.1186/s12942-018-0154-8 (PMC6137739; doi:10.1186/s12942-018-0154-8)
Supplement: Supplementary file 6 — Additional file 6. Prevalence of hospitalizations for each disease in the cities of Rio Grande do Sul. [file 12942_2018_154_MOESM6_ESM.docx]

**Additional file 6 – Prevalence of hospitalizations for each disease in the cities of Rio Grande do Sul**

| **Town** | **RA** | **MS** | **CD** | **Leukemia** |
| --- | --- | --- | --- | --- |
| Aceguá | 1.081 | 0.477 | 1.022 | 0.078 |
| Água Santa | 2.778 | 2.348 | 0.402 | 0.737 |
| Agudo | 2.356 | 2.655 | 0.806 | 1.887 |
| Ajuricaba | 0.770 | 1.157 | 0 | 0.898 |
| Alecrim | 1.190 | 1.489 | 1.275 | 0.584 |
| Alegrete | 1.393 | 1.549 | 0.675 | 0.932 |
| Alegria | 0.715 | 0.975 | 0.348 | 0.159 |
| Almirante Tamandaré Do Sul | 0.676 | 1.015 | 0 | 1.991 |
| Alpestre | 1.810 | 2.657 | 1.119 | 1.196 |
| Alto Alegre | 3.327 | 2.081 | 0 | 3.897 |
| Alto Feliz | 1.054 | 1.438 | 0.513 | 0 |
| Alvorada | 0.430 | 0.390 | 2.609 | 1.272 |
| Amaral Ferrador | 0.528 | 0.660 | 0 | 0.162 |
| Ametista Do Sul | 0.878 | 1.528 | 0 | 1.639 |
| André Da Rocha | 1.609 | 1.150 | 0 | 0 |
| Anta Gorda | 1.242 | 1.324 | 0.740 | 2.089 |
| Antônio Prado | 1.219 | 0.736 | 0.817 | 0.695 |
| Arambaré | 0.757 | 0.379 | 0 | 0.186 |
| Araricá | 0.460 | 0.359 | 0.923 | 1.551 |
| Aratiba | 2.426 | 2.929 | 0.912 | 2.559 |
| Arroio Do Meio | 1.830 | 1.526 | 0.717 | 1.114 |
| Arroio Do Sal | 1.227 | 1.039 | 4.449 | 0.576 |
| Arroio Do Padre | 0.205 | 0 | 0 | 0 |
| Arroio Dos Ratos | 3.676 | 5.062 | 1.100 | 0.907 |
| Arroio Do Tigre | 4.838 | 4.838 | 1.302 | 1.464 |
| Arroio Grande | 0.953 | 0.984 | 0.567 | 0.260 |
| Arvorezinha | 1.886 | 1.505 | 0.732 | 1.107 |
| Augusto Pestana | 0.866 | 1.823 | 0.422 | 0.435 |
| Áurea | 1.754 | 1.622 | 1.225 | 2.526 |
| Bagé | 0.828 | 0.820 | 0.641 | 1.148 |
| Balneário Pinhal | 0.129 | 0.097 | 0 | 0.379 |
| Barão | 6.717 | 1.279 | 0.261 | 0.717 |
| Barão De Cotegipe | 3.424 | 4.445 | 0.229 | 3.256 |
| Barão Do Triunfo | 3.464 | 3.637 | 0.427 | 1.857 |
| Barracão | 1.252 | 1.436 | 0.559 | 0.384 |
| Barra Do Guarita | 0.905 | 0.566 | 1.454 | 1.332 |
| Barra Do Quaraí | 0.139 | 0.174 | 0 | 0.513 |
| Barra Do Ribeiro | 0.756 | 0.723 | 0.595 | 0.546 |
| **Town** | **RA** | **MS** | **CD** | **Leukemia** |
| Barra Do Rio Azul | 1.814 | 3.317 | 0 | 2.568 |
| Barra Funda | 1.062 | 0.886 | 0.632 | 2.173 |
| Barros Cassal | 3.790 | 2.638 | 2.690 | 0.585 |
| Benjamin Constant Do Sul | 0.848 | 0.758 | 0.649 | 3.419 |
| Bento Gonçalves | 1.664 | 1.718 | 0.893 | 0.940 |
| Boa Vista Das Missões | 0.661 | 0.662 | 0 | 0 |
| Boa Vista Do Buricá | 0.850 | 0.745 | 0.911 | 0.052 |
| Boa Vista Do Cadeado | 0.458 | 0.143 | 0 | 0.140 |
| Boa Vista Do Incra | 0.461 | 0.577 | 0 | 0.424 |
| Boa Vista Do Sul | 1.913 | 1.259 | 0.539 | 1.235 |
| Bom Jesus | 1.577 | 1.791 | 1.300 | 3.959 |
| Bom Princípio | 6.091 | 7.919 | 0.762 | 2.269 |
| Bom Progresso | 1.200 | 1.051 | 1.286 | 2.062 |
| Bom Retiro Do Sul | 2.168 | 4.541 | 0.522 | 1.046 |
| Boqueirão Do Leão | 1.493 | 1.276 | 0 | 3.352 |
| Bossoroca | 1.664 | 3.149 | 0.217 | 1.195 |
| Bozano | 0.254 | 0 | 0 | 0.156 |
| Braga | 0.528 | 0.283 | 1.618 | 2.130 |
| Brochier | 1.972 | 0.748 | 6.085 | 0.147 |
| Butiá | 4.478 | 5.260 | 0.807 | 0.622 |
| Caçapava Do Sul | 0.979 | 1.079 | 1.022 | 0.478 |
| Cacequi | 4.801 | 5.752 | 0.109 | 0.602 |
| Cachoeira Do Sul | 0.600 | 0.638 | 0.554 | 0.867 |
| Cachoeirinha | 0.373 | 0.375 | 1.418 | 1.212 |
| Cacique Doble | 0.861 | 1.365 | 1.845 | 1.479 |
| Caibaté | 4.851 | 1.623 | 0 | 0.831 |
| Caiçara | 6.061 | 7.584 | 0.295 | 2.029 |
| Camaquã | 0.913 | 0.813 | 0.286 | 0.863 |
| Camargo | 0.970 | 0.809 | 0 | 2.249 |
| Cambará Do Sul | 0.598 | 0.588 | 0 | 0.262 |
| Campestre Da Serra | 1.721 | 1.508 | 0.922 | 0.211 |
| Campina Das Missões | 1.005 | 0.915 | 1.958 | 1.962 |
| Campinas Do Sul | 1.116 | 1.524 | 0.272 | 0.498 |
| Campo Bom | 0.674 | 0.332 | 2.293 | 1.050 |
| Campo Novo | 0.307 | 0.512 | 0.274 | 0.754 |
| Campos Borges | 2.959 | 5.003 | 0.428 | 0.098 |
| Candelária | 1.065 | 0.521 | 0.794 | 0.375 |
| Cândido Godói | 0.941 | 1.284 | 0.229 | 0.052 |
| Candiota | 0.669 | 0.478 | 0.171 | 1.173 |
| Canela | 2.051 | 2.264 | 0.343 | 0.577 |
| Canguçu | 0.546 | 0.446 | 0.618 | 0.515 |
| **Town** | **RA** | **MS** | **CD** | **Leukemia** |
| Canoas | 0.574 | 0.369 | 0.800 | 0.971 |
| Canudos Do Vale | 1.392 | 0.774 | 0.829 | 0.190 |
| Capão Bonito Do Sul | 0.637 | 0.399 | 0 | 2.151 |
| Capão Da Canoa | 0.671 | 0.665 | 0.962 | 0.799 |
| Capão Do Cipó | 0.360 | 0.451 | 0 | 0.221 |
| Capão Do Leão | 0.402 | 0.259 | 0.616 | 1.143 |
| Capivari Do Sul | 1.006 | 1.258 | 0 | 1.234 |
| Capela De Santana | 0.794 | 0.783 | 0.258 | 1.831 |
| Capitão | 2.332 | 3.183 | 1.136 | 0.390 |
| Carazinho | 1.206 | 1.250 | 1.742 | 0.937 |
| Caraá | 1.834 | 2.008 | 0 | 0.047 |
| Carlos Barbosa | 1.664 | 0.416 | 0.297 | 0.912 |
| Carlos Gomes | 1.217 | 1.088 | 0.932 | 2.134 |
| Casca | 1.227 | 1.495 | 0.173 | 1.110 |
| Caseiros | 1.580 | 1.279 | 1.992 | 0 |
| Catuípe | 0.929 | 0.750 | 0.321 | 0.699 |
| Caxias Do Sul | 0.474 | 0.319 | 0.461 | 0.604 |
| Centenário | 1.131 | 0.943 | 0 | 0.463 |
| Cerrito | 0.742 | 0.710 | 0.234 | 0.857 |
| Cerro Branco | 4.329 | 2.983 | 0 | 2.695 |
| Cerro Grande | 1.387 | 1.736 | 0.619 | 0.567 |
| Cerro Grande Do Sul | 0.708 | 0.443 | 0.146 | 0.434 |
| Cerro Largo | 1.262 | 0.816 | 0.451 | 1.368 |
| Chapada | 1.162 | 1.902 | 1.756 | 1.792 |
| Charqueadas | 1.796 | 1.851 | 0.424 | 1.097 |
| Charrua | 2.496 | 2.418 | 2.157 | 0.790 |
| Chiapetta | 0.691 | 0.519 | 0.370 | 1.018 |
| Chuí | 0.047 | 0 | 0 | 0.174 |
| Chuvisca | 1.187 | 1.273 | 0.908 | 0.069 |
| Cidreira | 0.221 | 0.248 | 0.236 | 0.487 |
| Ciríaco | 2.044 | 1.634 | 1.521 | 0.766 |
| Colinas | 2.194 | 1.878 | 4.331 | 3.259 |
| Colorado | 1.102 | 0.886 | 1.265 | 1.739 |
| Condor | 0.597 | 0.694 | 0 | 0.523 |
| Constantina | 1.146 | 1.291 | 0.921 | 1.231 |
| Coqueiro Baixo | 0.549 | 0.915 | 0.980 | 0 |
| Coqueiros Do Sul | 2.502 | 1.992 | 4.265 | 1.117 |
| Coronel Barros | 0.568 | 1.564 | 0 | 0.558 |
| Coronel Bicaco | 0.325 | 0.496 | 0.193 | 0.885 |
| Coronel Pilar | 1.620 | 0.203 | 0 | 0.398 |
| Cotiporã | 1.641 | 1.607 | 0.382 | 0.263 |
| **Town** | **RA** | **MS** | **CD** | **Leukemia** |
| Coxilha | 1.978 | 2.846 | 4.768 | 0.971 |
| Crissiumal | 0.357 | 0.298 | 0 | 0.682 |
| Cristal | 0.691 | 0.528 | 0.206 | 0.707 |
| Cristal Do Sul | 1.582 | 2.227 | 0 | 0.121 |
| Cruz Alta | 1.103 | 1.030 | 0.620 | 1.261 |
| Cruzaltense | 0 | 0 | 0 | 0.480 |
| Cruzeiro Do Sul | 1.882 | 2.214 | 0.972 | 1.754 |
| David Canabarro | 1.611 | 0.747 | 1.598 | 0.732 |
| Derrubadas | 1.139 | 1.096 | 0.939 | 0.752 |
| Dezesseis De Novembro | 0.682 | 1.342 | 0 | 0.598 |
| Dilermando De Aguiar | 0.091 | 1.712 | 0 | 1.119 |
| Dois Irmãos | 0.507 | 0.507 | 0.326 | 0.547 |
| Dois Irmãos Das Missões | 0.259 | 0.486 | 0.694 | 0.159 |
| Dois Lajeados | 3.751 | 3.093 | 0.913 | 0.209 |
| Dom Feliciano | 0.933 | 1.313 | 0.312 | 0.286 |
| Dom Pedro De Alcântara | 0.438 | 0.274 | 6.458 | 0.672 |
| Dom Pedrito | 0.927 | 1.366 | 0.539 | 0.608 |
| Dona Francisca | 4.765 | 6.785 | 0 | 1.210 |
| Doutor Maurício Cardoso | 2.840 | 2.830 | 1.127 | 0.323 |
| Doutor Ricardo | 1.789 | 2.411 | 0 | 0.169 |
| Eldorado Do Sul | 1.041 | 1.517 | 0.523 | 1.048 |
| Encantado | 0.777 | 0.784 | 0.584 | 0.619 |
| Encruzilhada Do Sul | 0.786 | 0.670 | 0.488 | 1.286 |
| Engenho Velho | 0.915 | 1.374 | 0.980 | 1.347 |
| Entre-Ijuís | 1.845 | 1.252 | 0.335 | 0.691 |
| Entre Rios Do Sul | 0.454 | 0.454 | 0.486 | 1.559 |
| Erebango | 1.035 | 2.001 | 0.504 | 0.346 |
| Erechim | 1.003 | 1.128 | 1.013 | 1.310 |
| Ernestina | 1.357 | 1.132 | 1.939 | 0.999 |
| Herval | 1.034 | 1.191 | 0.443 | 0.152 |
| Erval Grande | 1.082 | 1.016 | 2.320 | 0.797 |
| Erval Seco | 1.454 | 1.820 | 0.570 | 1.349 |
| Esmeralda | 0.617 | 0.662 | 0 | 0.108 |
| Esperança Do Sul | 0.769 | 1.069 | 0.915 | 0.838 |
| Espumoso | 1.302 | 1.170 | 0.491 | 1.350 |
| Estação | 0.790 | 1.047 | 1.245 | 1.883 |
| Estância Velha | 0.354 | 0.279 | 0.422 | 0.757 |
| Esteio | 0.585 | 0.459 | 0.501 | 0.972 |
| Estrela | 1.150 | 1.267 | 1.271 | 1.433 |
| Estrela Velha | 5.391 | 5.108 | 0 | 0.473 |
| Eugênio De Castro | 0.699 | 0.750 | 2.675 | 0.735 |
| **Town** | **RA** | **MS** | **CD** | **Leukemia** |
| Fagundes Varela | 0.975 | 0.813 | 0.581 | 0.399 |
| Farroupilha | 0.667 | 0.577 | 0.118 | 0.577 |
| Faxinal Do Soturno | 2.136 | 2.358 | 6.283 | 1.439 |
| Faxinalzinho | 2.177 | 2.588 | 2.333 | 0.935 |
| Fazenda Vilanova | 1.965 | 3.972 | 0.405 | 0.278 |
| Feliz | 1.379 | 1.386 | 0.485 | 1.471 |
| Flores Da Cunha | 0.371 | 0.296 | 0.276 | 0.455 |
| Floriano Peixoto | 3.739 | 4.158 | 0.742 | 0.850 |
| Fontoura Xavier | 1.408 | 1.533 | 1.117 | 1.280 |
| Formigueiro | 2.550 | 3.140 | 0.213 | 1.564 |
| Forquetinha | 0.225 | 0.141 | 1.208 | 0.415 |
| Fortaleza Dos Valos | 0.855 | 0.917 | 0.327 | 1.649 |
| Frederico Westphalen | 0.804 | 2.109 | 0.208 | 0.785 |
| Garibaldi | 1.211 | 0.422 | 1.024 | 0.346 |
| Garruchos | 0.518 | 0.432 | 0.463 | 0.636 |
| Gaurama | 1.716 | 2.982 | 1.022 | 1.111 |
| General Câmara | 3.506 | 4.056 | 0.354 | 1.055 |
| Gentil | 1.833 | 2.085 | 0.893 | 1.431 |
| Getúlio Vargas | 1.626 | 2.402 | 1.576 | 1.380 |
| Giruá | 0.753 | 0.655 | 0.351 | 0.924 |
| Glorinha | 0.243 | 0.101 | 1.086 | 1.393 |
| Gramado | 1.186 | 0.704 | 0.510 | 0.893 |
| Gramado Dos Loureiros | 4.064 | 4.777 | 0.660 | 0.907 |
| Gramado Xavier | 2.323 | 2.202 | 0.754 | 2.073 |
| Gravataí | 0.392 | 0.398 | 1.277 | 0.936 |
| Guabiju | 3.147 | 3.938 | 1.874 | 3.004 |
| Guaíba | 0.655 | 0.760 | 0.723 | 0.720 |
| Guaporé | 8.978 | 6.452 | 1.444 | 1.097 |
| Guarani Das Missões | 3.271 | 3.318 | 0.922 | 0.718 |
| Harmonia | 0.920 | 1.068 | 1.056 | 0.161 |
| Herveiras | 2.081 | 1.302 | 0 | 0.929 |
| Horizontina | 0.640 | 0.610 | 0.245 | 1.047 |
| Hulha Negra | 1.803 | 1.909 | 0 | 0.227 |
| Humaitá | 0.341 | 0.711 | 0.304 | 2.719 |
| Ibarama | 5.562 | 3.760 | 0.343 | 2.118 |
| Ibiaçá | 1.365 | 1.262 | 0.318 | 0.801 |
| Ibiraiaras | 1.091 | 0.975 | 1.253 | 0.717 |
| Ibirapuitã | 0.894 | 1.636 | 0.737 | 2.702 |
| Ibirubá | 2.431 | 2.933 | 0.310 | 0.852 |
| Igrejinha | 1.112 | 1.027 | 0.567 | 0.975 |
| Ijuí | 0.729 | 0.926 | 1.271 | 0.956 |
| **Town** | **RA** | **MS** | **CD** | **Leukemia** |
| Ilópolis | 3.065 | 2.898 | 1.825 | 0.167 |
| Imbé | 0.285 | 0.139 | 0.508 | 1.223 |
| Imigrante | 2.218 | 2.082 | 3.467 | 0.340 |
| Independência | 0.422 | 0.581 | 0.226 | 1.088 |
| Inhacorá | 0.493 | 0.463 | 0 | 0.605 |
| Ipê | 3.484 | 1.802 | 1.244 | 1.710 |
| Ipiranga Do Sul | 0.719 | 1.619 | 0 | 1.588 |
| Iraí | 0.588 | 0.693 | 0.927 | 0.340 |
| Itaara | 0.223 | 0.140 | 0.299 | 0.411 |
| Itacurubi | 0.487 | 0.305 | 0 | 0.100 |
| Itapuca | 2.980 | 2.983 | 1.277 | 3.657 |
| Itaqui | 0.857 | 1.008 | 0.275 | 0.854 |
| Itati | 0.324 | 0.406 | 0 | 0.664 |
| Itatiba Do Sul | 2.278 | 3.353 | 0.718 | 2.795 |
| Ivorá | 0.648 | 1.622 | 0 | 1.590 |
| Ivoti | 0.591 | 0.387 | 0.527 | 0.759 |
| Jaboticaba | 0.955 | 0.939 | 0.731 | 0.669 |
| Jacuizinho | 1.115 | 0.279 | 1.194 | 0.547 |
| Jacutinga | 1.615 | 1.059 | 1.236 | 1.133 |
| Jaguarão | 0.700 | 0.651 | 0.107 | 1.289 |
| Jaguari | 0.706 | 0.640 | 0 | 1.136 |
| Jaquirana | 1.405 | 0.837 | 3.226 | 0.328 |
| Jari | 2.814 | 2.543 | 0.838 | 3.357 |
| Jóia | 0.302 | 0.420 | 0.359 | 0.782 |
| Júlio De Castilhos | 1.898 | 2.089 | 0.382 | 1.261 |
| Lagoa Bonita Do Sul | 6.718 | 0.788 | 0 | 0 |
| Lagoão | 2.349 | 2.035 | 0 | 0.665 |
| Lagoa Dos Três Cantos | 2.798 | 1.532 | 0.937 | 1.288 |
| Lagoa Vermelha | 0.934 | 1.042 | 1.523 | 0.934 |
| Lajeado | 1.193 | 1.434 | 0.775 | 0.816 |
| Lajeado Do Bugre | 0.449 | 0.984 | 0 | 1.517 |
| Lavras Do Sul | 2.220 | 2.140 | 1.170 | 0.893 |
| Liberato Salzano | 0.290 | 0.302 | 0.518 | 0.297 |
| Lindolfo Collor | 0.481 | 0.468 | 0 | 0.131 |
| Linha Nova | 1.032 | 0.861 | 0 | 0.633 |
| Machadinho | 0.507 | 0.571 | 0.815 | 0.311 |
| Maçambará | 0.472 | 0.590 | 0 | 1.375 |
| Mampituba | 2.698 | 3.493 | 3.490 | 0.685 |
| Manoel Viana | 0.395 | 0.544 | 0.212 | 0.970 |
| Maquiné | 1.700 | 1.924 | 0.434 | 0.993 |
| Maratá | 2.433 | 1.384 | 5.332 | 0.136 |
| **Town** | **RA** | **MS** | **CD** | **Leukemia** |
| Marau | 1.452 | 1.365 | 0.865 | 1.245 |
| Marcelino Ramos | 0.871 | 1.771 | 0.292 | 1.069 |
| Mariana Pimentel | 0.371 | 0.835 | 1.192 | 0.637 |
| Mariano Moro | 1.264 | 1.582 | 0 | 0.621 |
| Marques De Souza | 0.962 | 0.859 | 2.576 | 0.169 |
| Mata | 2.788 | 2.121 | 0 | 0.738 |
| Mato Castelhano | 2.149 | 2.831 | 1.818 | 1.249 |
| Mato Leitão | 1.157 | 1.176 | 0.387 | 0.444 |
| Mato Queimado | 2.330 | 0.194 | 0 | 0.191 |
| Maximiliano De Almeida | 4.097 | 3.417 | 0 | 0.140 |
| Minas Do Leão | 4.870 | 5.910 | 0.392 | 1.168 |
| Miraguaí | 0.921 | 0.504 | 2.159 | 1.413 |
| Montauri | 1.450 | 0.680 | 1.942 | 0 |
| Monte Alegre Dos Campos | 0.991 | 0.564 | 2.413 | 0.774 |
| Monte Belo Do Sul | 1.465 | 1.571 | 0 | 0.514 |
| Montenegro | 1.684 | 1.971 | 0.479 | 0.479 |
| Mormaço | 1.525 | 1.017 | 1.634 | 0.499 |
| Morrinhos Do Sul | 2.634 | 2.527 | 7.057 | 0.216 |
| Morro Redondo | 0.359 | 0.337 | 0.721 | 0.110 |
| Morro Reuter | 0.492 | 0.678 | 0.528 | 2.477 |
| Mostardas | 0.415 | 0.577 | 0.617 | 1.640 |
| Muçum | 2.333 | 2.700 | 4.687 | 1.217 |
| Muitos Capões | 0.187 | 0.585 | 0 | 0.574 |
| Muliterno | 2.774 | 1.928 | 1.652 | 0.378 |
| Não-Me-Toque | 1.455 | 1.667 | 0.658 | 1.270 |
| Nicolau Vergueiro | 1.137 | 1.422 | 0.870 | 1.793 |
| Nonoai | 1.898 | 2.432 | 0.868 | 1.306 |
| Nova Alvorada | 1.317 | 1.319 | 0.470 | 2.371 |
| Nova Araçá | 5.517 | 6.554 | 0 | 0.514 |
| Nova Bassano | 8.882 | 8.939 | 0.508 | 0.349 |
| Nova Boa Vista | 1.141 | 3.568 | 1.528 | 3.499 |
| Nova Bréscia | 5.090 | 6.698 | 2.821 | 0.538 |
| Nova Candelária | 1.117 | 0.763 | 0.544 | 1.371 |
| Nova Esperança Do Sul | 0.538 | 0.299 | 0.962 | 0.587 |
| Nova Hartz | 0.487 | 0.438 | 0.653 | 0.710 |
| Nova Pádua | 0.456 | 0.571 | 0 | 0.980 |
| Nova Palma | 0.617 | 5.568 | 0 | 0.865 |
| Nova Petrópolis | 1.482 | 1.395 | 0.157 | 0.864 |
| Nova Prata | 0.783 | 0.919 | 0.525 | 0.631 |
| Nova Ramada | 1.032 | 0.717 | 0.614 | 0.422 |
| Nova Roma Do Sul | 2.006 | 2.196 | 0 | 0.103 |
| **Town** | **RA** | **MS** | **CD** | **Leukemia** |
| Nova Santa Rita | 0.246 | 0.200 | 0.198 | 0.981 |
| Novo Cabrais | 3.479 | 2.086 | 8.544 | 1.067 |
| Novo Hamburgo | 0.297 | 0.282 | 0.539 | 0.875 |
| Novo Machado | 0.925 | 0.802 | 0 | 0.437 |
| Novo Tiradentes | 0.123 | 1.075 | 0 | 0.602 |
| Novo Xingu | 0.636 | 0.398 | 0.852 | 0.195 |
| Novo Barreiro | 1.054 | 1.494 | 0.376 | 0.259 |
| Osório | 1.510 | 1.393 | 0.549 | 0.637 |
| Paim Filho | 1.120 | 0.742 | 0.353 | 0.647 |
| Palmares Do Sul | 2.420 | 2.741 | 0.273 | 1.063 |
| Palmeira Das Missões | 1.001 | 1.181 | 1.003 | 0.699 |
| Palmitinho | 1.777 | 4.497 | 1.082 | 0.446 |
| Panambi | 0.999 | 0.781 | 0.433 | 0.847 |
| Pantano Grande | 0.480 | 0.424 | 0.303 | 1.455 |
| Paraí | 7.096 | 7.956 | 0.659 | 0.755 |
| Paraíso Do Sul | 3.085 | 3.765 | 0.408 | 0.748 |
| Pareci Novo | 2.069 | 2.191 | 0 | 0.488 |
| Parobé | 0.944 | 0.747 | 0.727 | 0.799 |
| Passa Sete | 5.422 | 2.239 | 0.290 | 0.665 |
| Passo Do Sobrado | 0.651 | 0.640 | 0.249 | 0.399 |
| Passo Fundo | 1.448 | 1.640 | 4.212 | 1.763 |
| Paulo Bento | 0.509 | 2.229 | 0.682 | 0.781 |
| Paverama | 1.042 | 1.130 | 0.931 | 1.108 |
| Pedras Altas | 1.011 | 1.106 | 0.677 | 0.465 |
| Pedro Osório | 0.751 | 0.627 | 0.767 | 0.746 |
| Pejuçara | 1.477 | 1.496 | 0.377 | 0.690 |
| Pelotas | 0.630 | 0.605 | 0.629 | 0.697 |
| Picada Café | 0.971 | 0.540 | 1.156 | 1.456 |
| Pinhal | 1.779 | 1.809 | 1.192 | 2.593 |
| Pinhal Da Serra | 0.525 | 0 | 0 | 0 |
| Pinhal Grande | 1.562 | 1.564 | 0.335 | 0.997 |
| Pinheirinho Do Vale | 1.802 | 2.877 | 0 | 1.373 |
| Pinheiro Machado | 1.574 | 1.970 | 0.351 | 0.376 |
| Pirapó | 2.939 | 2.917 | 0 | 0.498 |
| Piratini | 2.803 | 2.960 | 0.226 | 0.812 |
| Planalto | 0.504 | 0.432 | 0.285 | 0.847 |
| Poço Das Antas | 1.939 | 1.907 | 0.742 | 0.340 |
| Pontão | 1.449 | 0.816 | 2.329 | 1.689 |
| Ponte Preta | 2.714 | 2.198 | 0.855 | 2.351 |
| Portão | 0.560 | 0.373 | 0.678 | 0.843 |
| Porto Alegre | 0.460 | 0.418 | 1.506 | 1.114 |
| **Town** | **RA** | **MS** | **CD** | **Leukemia** |
| Porto Lucena | 0.981 | 0.840 | 0.553 | 0.633 |
| Porto Mauá | 0.550 | 0.413 | 0.589 | 0.405 |
| Porto Vera Cruz | 1.207 | 0.755 | 1.617 | 1.852 |
| Porto Xavier | 1.085 | 1.159 | 0.284 | 0.877 |
| Pouso Novo | 2.533 | 2.984 | 0 | 4.572 |
| Presidente Lucena | 0.787 | 0.704 | 0 | 2.899 |
| Progresso | 9.884 | 7.659 | 0.486 | 0.389 |
| Protásio Alves | 0.559 | 1.224 | 0 | 0.857 |
| Putinga | 1.822 | 0.675 | 6.146 | 0.911 |
| Quaraí | 0.825 | 0.592 | 0.650 | 1.058 |
| Quatro Irmãos | 0.315 | 0.394 | 0 | 0 |
| Quevedos | 0.309 | 0.903 | 0 | 0.253 |
| Quinze De Novembro | 1.912 | 2.297 | 2.049 | 0.845 |
| Redentora | 0.601 | 0.239 | 1.025 | 0.973 |
| Relvado | 4.279 | 4.543 | 2.779 | 0.159 |
| Restinga Seca | 4.055 | 3.949 | 0.378 | 0.627 |
| Rio Dos Índios | 2.086 | 2.514 | 1.242 | 0.569 |
| Rio Grande | 0.808 | 0.800 | 0.858 | 0.840 |
| Rio Pardo | 0.870 | 0.688 | 0.319 | 0.410 |
| Riozinho | 3.743 | 4.280 | 0.692 | 0.713 |
| Roca Sales | 3.913 | 1.870 | 2.620 | 1.134 |
| Rodeio Bonito | 0.973 | 1.887 | 0.521 | 2.030 |
| Rolador | 0.768 | 0.687 | 0.588 | 0.673 |
| Rolante | 2.251 | 2.602 | 0.307 | 0.827 |
| Ronda Alta | 0.683 | 1.197 | 1.025 | 1.845 |
| Rondinha | 1.469 | 1.077 | 0.814 | 2.548 |
| Roque Gonzales | 0.621 | 2.136 | 0.208 | 0.619 |
| Rosário Do Sul | 1.330 | 1.655 | 0.302 | 0.648 |
| Sagrada Família | 1.184 | 1.347 | 0 | 0.661 |
| Saldanha Marinho | 2.240 | 1.584 | 1.044 | 0.478 |
| Salto Do Jacuí | 2.187 | 2.060 | 0.126 | 1.645 |
| Salvador Das Missões | 1.466 | 1.179 | 1.683 | 0.899 |
| Salvador Do Sul | 4.183 | 2.539 | 9.320 | 1.372 |
| Sananduva | 2.854 | 2.274 | 0.876 | 0.848 |
| Santa Bárbara Do Sul | 2.184 | 1.505 | 1.187 | 0.583 |
| Santa Cecília Do Sul | 2.533 | 3.169 | 0.905 | 1.243 |
| Santa Clara Do Sul | 2.060 | 2.823 | 0 | 2.648 |
| Santa Cruz Do Sul | 1.114 | 0.597 | 0.835 | 1.388 |
| Santa Maria | 0.236 | 0.276 | 0.178 | 1.525 |
| Santa Maria Do Herval | 0.785 | 0.578 | 0.247 | 1.360 |
| Santa Margarida Do Sul | 0.594 | 0.595 | 1.910 | 2.624 |
| **Town** | **RA** | **MS** | **CD** | **Leukemia** |
| Santana Da Boa Vista | 1.390 | 1.273 | 0.545 | 0.458 |
| Sant'ana Do Livramento | 1.318 | 1.225 | 1.107 | 0.869 |
| Santa Rosa | 0.990 | 0.617 | 0.764 | 0.520 |
| Santa Tereza | 1.625 | 1.220 | 0.870 | 0.598 |
| Santa Vitória Do Palmar | 0.532 | 0.587 | 0.966 | 1.361 |
| Santiago | 1.048 | 1.304 | 0.549 | 1.495 |
| Santo Ângelo | 1.927 | 1.426 | 0.923 | 0.989 |
| Santo Antônio Do Palma | 2.090 | 2.942 | 0 | 1.122 |
| Santo Antônio Da Patrulha | 1.267 | 1.304 | 0.943 | 1.020 |
| Santo Antônio Das Missões | 0.598 | 0.780 | 0.267 | 2.264 |
| Santo Antônio Do Planalto | 1.547 | 0.880 | 1.507 | 0.690 |
| Santo Augusto | 1.320 | 0.726 | 0.214 | 0.540 |
| Santo Cristo | 0.525 | 0.535 | 0.208 | 0.930 |
| Santo Expedito Do Sul | 2.157 | 1.563 | 0.608 | 2.229 |
| São Borja | 0.725 | 0.771 | 0.243 | 1.168 |
| São Domingos Do Sul | 1.528 | 1.792 | 0 | 1.992 |
| São Francisco De Assis | 1.190 | 1.689 | 0.622 | 1.799 |
| São Francisco De Paula | 4.640 | 2.486 | 1.021 | 0.935 |
| São Gabriel | 2.465 | 2.274 | 3.345 | 0.760 |
| São Jerônimo | 4.646 | 4.913 | 1.150 | 0.310 |
| São João Da Urtiga | 1.892 | 0.888 | 1.267 | 0.508 |
| São João Do Polêsine | 1.485 | 1.194 | 1.136 | 1.041 |
| São Jorge | 3.425 | 3.655 | 0 | 0.989 |
| São José Das Missões | 0.616 | 0.514 | 1.101 | 1.387 |
| São José Do Herval | 5.832 | 6.504 | 0.679 | 0.311 |
| São José Do Hortêncio | 0.819 | 0.342 | 1.097 | 0.838 |
| São José Do Inhacorá | 3.302 | 3.337 | 1.361 | 1.403 |
| São José Do Norte | 0.734 | 1.494 | 0.411 | 1.358 |
| São José Do Ouro | 1.093 | 1.469 | 0.867 | 1.242 |
| São José Do Sul | 1.342 | 0.168 | 6.472 | 0.494 |
| São José Dos Ausentes | 0.934 | 0.850 | 0.455 | 0 |
| São Leopoldo | 0.435 | 0.363 | 0.811 | 0.884 |
| São Lourenço Do Sul | 1.128 | 1.119 | 0.313 | 0.970 |
| São Luiz Gonzaga | 1.019 | 1.376 | 0.390 | 0.824 |
| São Marcos | 2.377 | 1.948 | 0.447 | 0.546 |
| São Martinho | 0.484 | 0.545 | 0.519 | 0.772 |
| São Martinho Da Serra | 0.087 | 0.328 | 0.468 | 2.035 |
| São Miguel Das Missões | 0.527 | 0.754 | 0.202 | 0.693 |
| São Nicolau | 0.390 | 0.488 | 0 | 0.299 |
| São Paulo Das Missões | 3.074 | 3.736 | 0 | 2.209 |
| São Pedro Da Serra | 3.456 | 1.898 | 9.484 | 1.655 |
| **Town** | **RA** | **MS** | **CD** | **Leukemia** |
| São Pedro Das Missões | 0.593 | 0.185 | 0.794 | 1.273 |
| São Pedro Do Butiá | 1.167 | 1.339 | 0.521 | 2.387 |
| São Pedro Do Sul | 0.871 | 3.418 | 0.457 | 1.508 |
| São Sebastião Do Caí | 0.573 | 0.558 | 0.683 | 1.141 |
| São Sepé | 1.033 | 8.991 | 0.315 | 2.666 |
| São Valentim | 0.615 | 0.963 | 1.237 | 1.983 |
| São Valentim Do Sul | 4.511 | 4.677 | 0 | 1.740 |
| São Valério Do Sul | 0.950 | 0.660 | 0.566 | 0.130 |
| São Vendelino | 7.043 | 8.813 | 0.770 | 1.764 |
| São Vicente Do Sul | 1.490 | 1.781 | 0.355 | 1.381 |
| Sapiranga | 0.768 | 0.676 | 1.497 | 1.143 |
| Sapucaia Do Sul | 0.531 | 0.507 | 0.720 | 1.032 |
| Sarandi | 0.604 | 1.117 | 0.563 | 1.192 |
| Seberi | 0.769 | 2.150 | 0 | 1.227 |
| Sede Nova | 0.742 | 0.813 | 0.497 | 0.228 |
| Segredo | 1.601 | 1.417 | 0 | 0.671 |
| Selbach | 1.474 | 1.348 | 0 | 0.696 |
| Senador Salgado Filho | 2.085 | 2.733 | 0 | 1.584 |
| Sentinela Do Sul | 1.183 | 0.807 | 0.288 | 0.858 |
| Serafina Corrêa | 1.451 | 0.932 | 1.155 | 1.588 |
| Sério | 1.715 | 1.380 | 0.656 | 0.150 |
| Sertão | 3.241 | 4.944 | 0.476 | 0.163 |
| Sertão Santana | 0.716 | 0.777 | 0.512 | 0.996 |
| Sete De Setembro | 3.815 | 2.469 | 5.639 | 3.713 |
| Severiano De Almeida | 1.018 | 2.184 | 1.948 | 1.428 |
| Silveira Martins | 1.027 | 1.142 | 0.611 | 2.240 |
| Sinimbu | 1.304 | 0.972 | 0.744 | 1.635 |
| Sobradinho | 5.165 | 2.521 | 0.839 | 1.489 |
| Soledade | 1.804 | 1.653 | 0.897 | 1.484 |
| Tabaí | 1.962 | 2.370 | 0 | 1.660 |
| Tapejara | 1.843 | 1.653 | 1.711 | 0.891 |
| Tapera | 1.391 | 1.640 | 0.287 | 0.952 |
| Tapes | 0.823 | 0.610 | 0.810 | 0.825 |
| Taquara | 1.314 | 1.472 | 0.548 | 0.577 |
| Taquari | 2.185 | 2.117 | 1.148 | 0.644 |
| Taquaruçu Do Sul | 0.377 | 1.532 | 0 | 2.543 |
| Tavares | 0.418 | 0.523 | 1.399 | 0.577 |
| Tenente Portela | 1.935 | 1.835 | 0.982 | 1.600 |
| Terra De Areia | 0.792 | 0.956 | 1.516 | 0.833 |
| Teutônia | 1.281 | 1.231 | 1.372 | 1.584 |
| Tio Hugo | 1.436 | 0.128 | 3.847 | 0.126 |
| **Town** | **RA** | **MS** | **CD** | **Leukemia** |
| Tiradentes Do Sul | 1.341 | 1.299 | 0.232 | 0.265 |
| Toropi | 0.757 | 3.198 | 1.014 | 0.813 |
| Torres | 2.161 | 2.542 | 6.437 | 0.811 |
| Tramandaí | 0.511 | 0.437 | 0.540 | 0.536 |
| Travesseiro | 1.811 | 1.511 | 1.294 | 1.334 |
| Três Arroios | 3.230 | 3.429 | 0 | 4.564 |
| Três Cachoeiras | 1.942 | 2.293 | 6.008 | 0.705 |
| Três Coroas | 0.785 | 0.630 | 0.628 | 0.978 |
| Três De Maio | 0.636 | 0.634 | 0.316 | 0.795 |
| Três Forquilhas | 2.589 | 3.000 | 5.138 | 1.294 |
| Três Palmeiras | 0.574 | 0.559 | 1.709 | 3.209 |
| Três Passos | 1.772 | 2.261 | 0.562 | 1.388 |
| Trindade Do Sul | 0.628 | 0.967 | 0.517 | 0.711 |
| Triunfo | 1.473 | 1.125 | 6.211 | 0.851 |
| Tucunduva | 0.332 | 0.237 | 0 | 0.116 |
| Tunas | 4.896 | 4.137 | 0.341 | 0.936 |
| Tupanci Do Sul | 1.954 | 0.667 | 0 | 1.090 |
| Tupanciretã | 1.417 | 1.758 | 1.949 | 1.616 |
| Tupandi | 3.917 | 5.792 | 2.671 | 0.437 |
| Tuparendi | 0.882 | 0.817 | 0.700 | 0.681 |
| Turuçu | 0.317 | 0.298 | 0.425 | 0.682 |
| Ubiretama | 1.095 | 1.218 | 0.652 | 0.149 |
| União Da Serra | 11.651 | 7.994 | 2.014 | 1.153 |
| Unistalda | 1.369 | 1.284 | 1.222 | 2.099 |
| Uruguaiana | 0.642 | 1.042 | 0.346 | 1.044 |
| Vacaria | 0.651 | 0.564 | 0.561 | 1.073 |
| Vale Verde | 1.546 | 1.612 | 0.920 | 1.054 |
| Vale Do Sol | 4.415 | 1.389 | 1.487 | 0.155 |
| Vale Real | 0.819 | 0.820 | 0.585 | 1.005 |
| Vanini | 2.113 | 1.057 | 1.509 | 0.346 |
| Venâncio Aires | 1.030 | 0.769 | 1.158 | 0.759 |
| Vera Cruz | 1.934 | 2.260 | 0.375 | 1.673 |
| Veranópolis | 0.735 | 0.582 | 0.394 | 0.496 |
| Vespasiano Correa | 2.831 | 2.834 | 6.826 | 0.695 |
| Viadutos | 1.631 | 1.448 | 0.846 | 1.291 |
| Viamão | 0.421 | 0.353 | 1.132 | 0.983 |
| Vicente Dutra | 0.899 | 1.455 | 0 | 2.466 |
| Victor Graeff | 1.473 | 0.806 | 0.493 | 1.242 |
| Vila Flores | 1.133 | 0.545 | 0 | 1.069 |
| Vila Lângaro | 2.986 | 2.762 | 2.783 | 0.956 |
| Vila Maria | 2.383 | 2.402 | 0 | 2.193 |
| **Town** | **RA** | **MS** | **CD** | **Leukemia** |
| Vila Nova Do Sul | 3.376 | 3.313 | 2.483 | 0.487 |
| Vista Alegre | 0.987 | 2.346 | 0.529 | 4.722 |
| Vista Alegre Do Prata | 4.274 | 4.902 | 0.954 | 1.530 |
| Vista Gaúcha | 1.418 | 1.901 | 2.171 | 0.373 |
| Vitória Das Missões | 1.844 | 1.104 | 0.859 | 2.361 |
| Westfalia | 0.800 | 0.626 | 1.072 | 0.491 |
| Xangri-Lá | 0.584 | 0.759 | 0.482 | 0.993 |
