# Supplementary material for: Spatial analyzes of HLA data in Rio Grande do Sul, south Brazil: genetic structure and possible correlation with autoimmune diseases
Source: Int J Health Geogr. 2018 Sep 14;17:34. doi: 10.1186/s12942-018-0154-8 (PMC6137739; doi:10.1186/s12942-018-0154-8)
Supplement: Supplementary file 7 — Additional file 7. Prevalence and cluster maps for each disease. [file 12942_2018_154_MOESM7_ESM.docx]

**Additional file 7 – Prevalence and cluster maps for each disease**

Multiple sclerosis

Rheumatoid arthritis


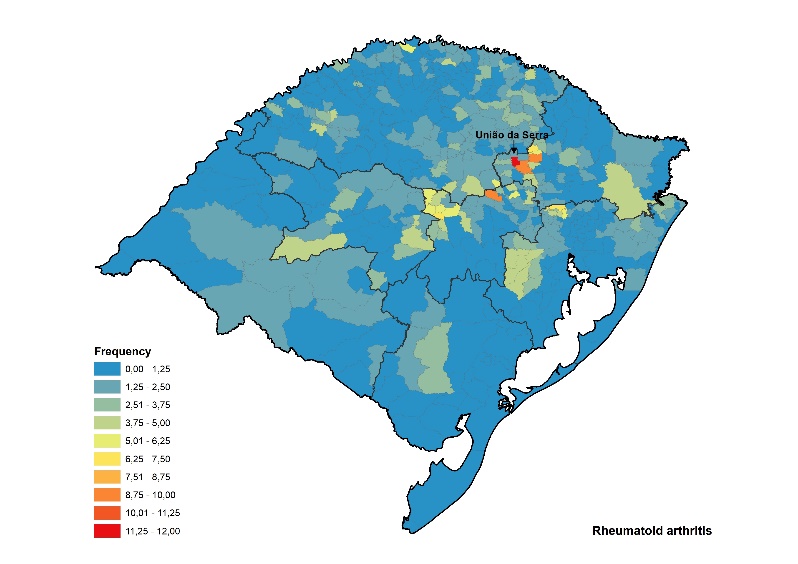

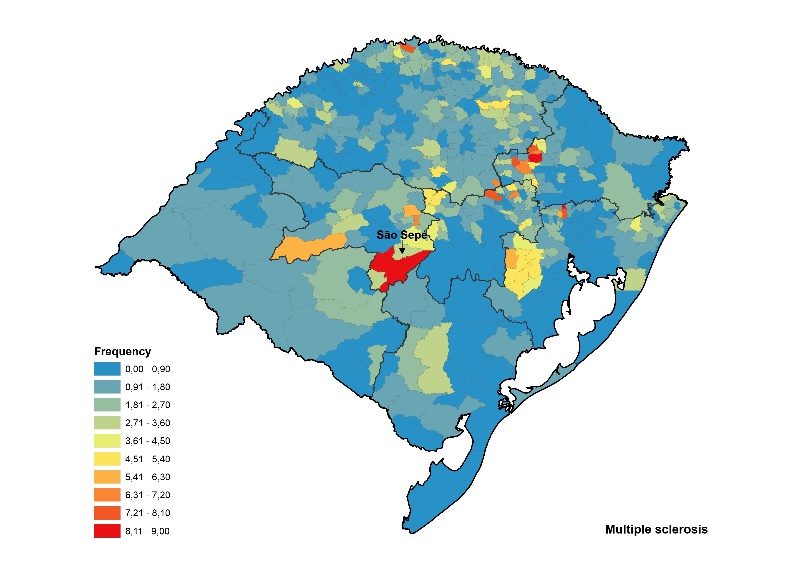


**
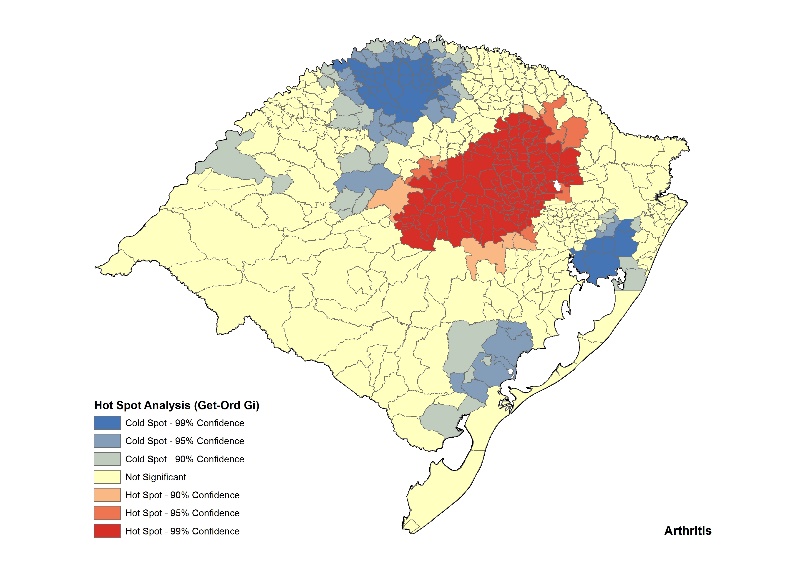

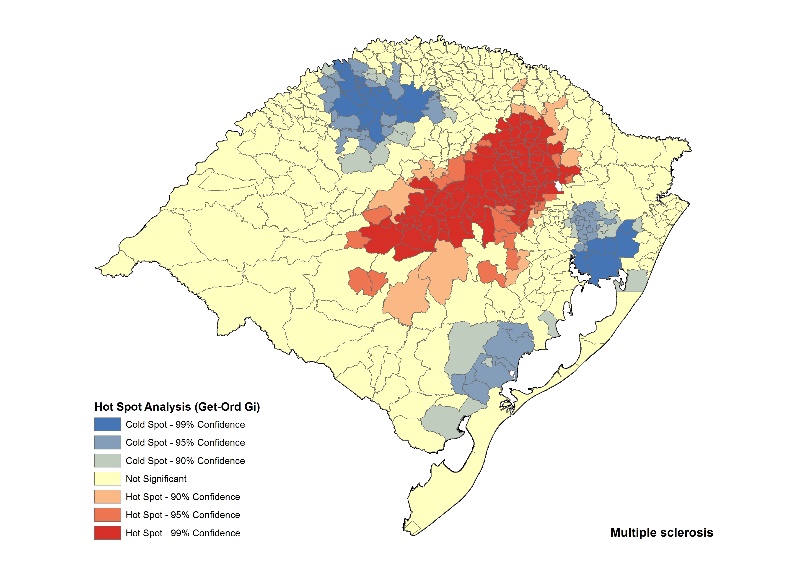
**

Leukemia

Chron’s disease


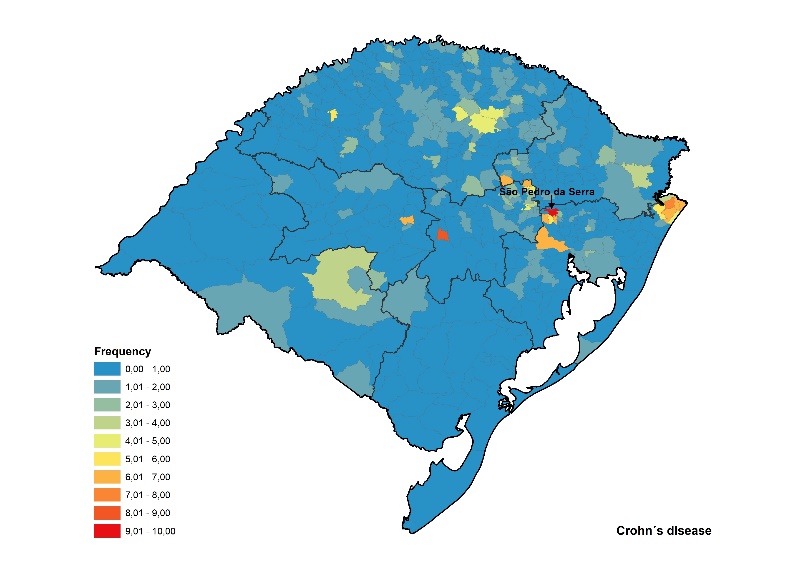

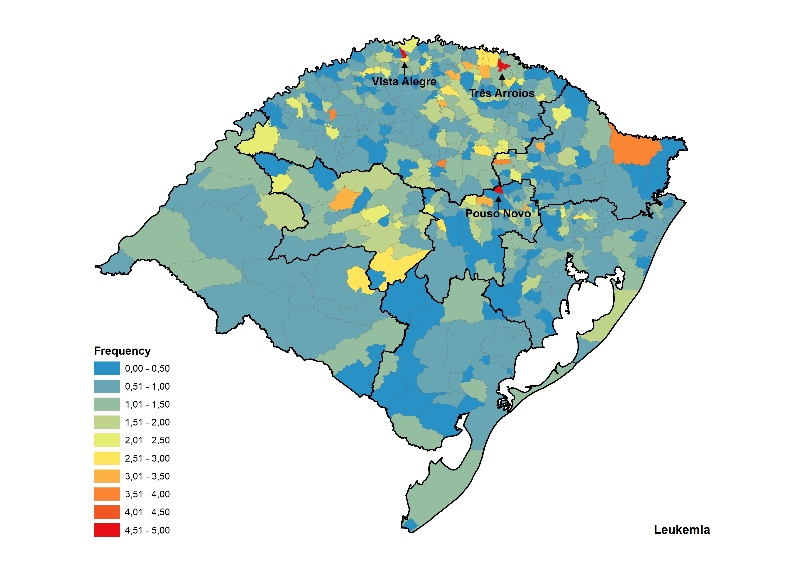


**
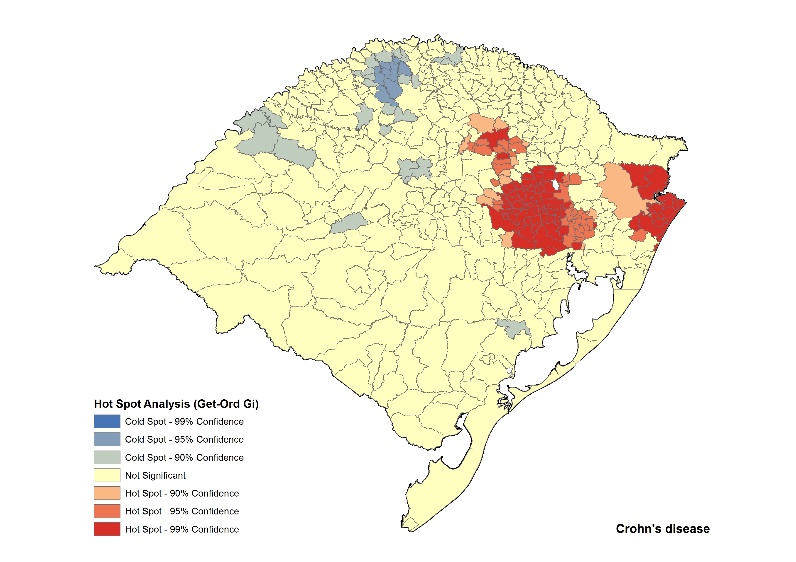

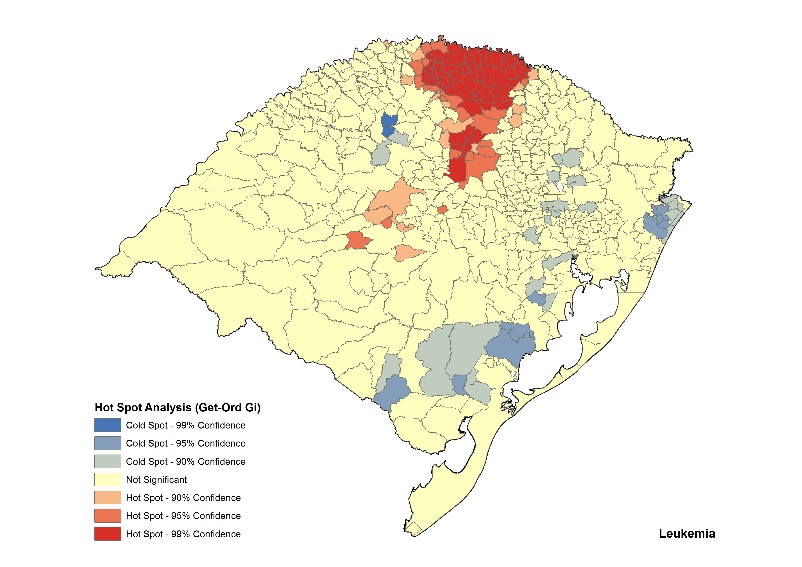
**
