# Supplementary material for: Spatial analyzes of HLA data in Rio Grande do Sul, south Brazil: genetic structure and possible correlation with autoimmune diseases
Source: Int J Health Geogr. 2018 Sep 14;17:34. doi: 10.1186/s12942-018-0154-8 (PMC6137739; doi:10.1186/s12942-018-0154-8)
Supplement: Supplementary file 8 — Additional file 8. Correlation maps between alleles and autoimmune diseases. [file 12942_2018_154_MOESM8_ESM.docx]

**Additional file 8 – Correlation maps between alleles and autoimmune diseases**


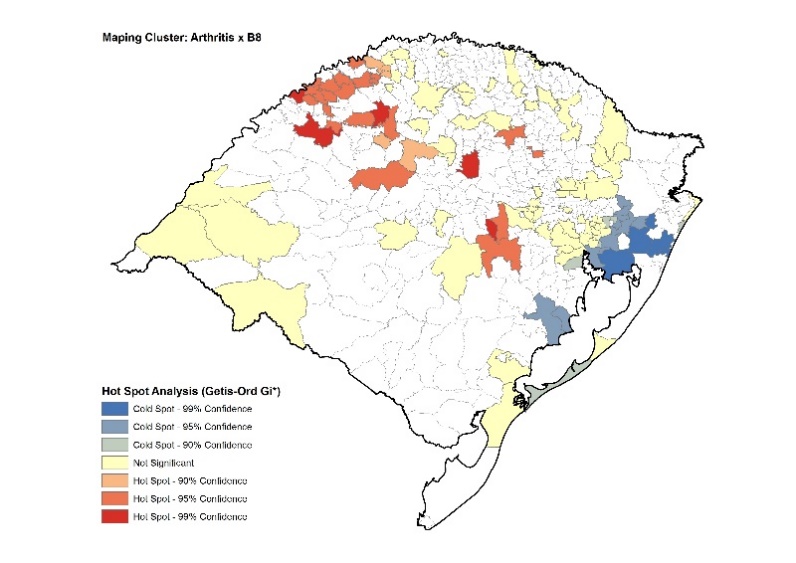

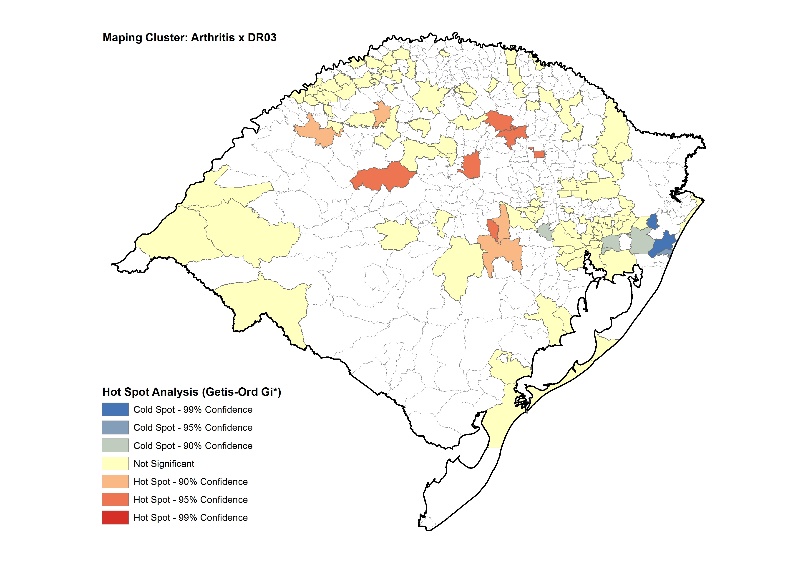


HLA-DRB1*03 x RA

HLA-B*08 x RA


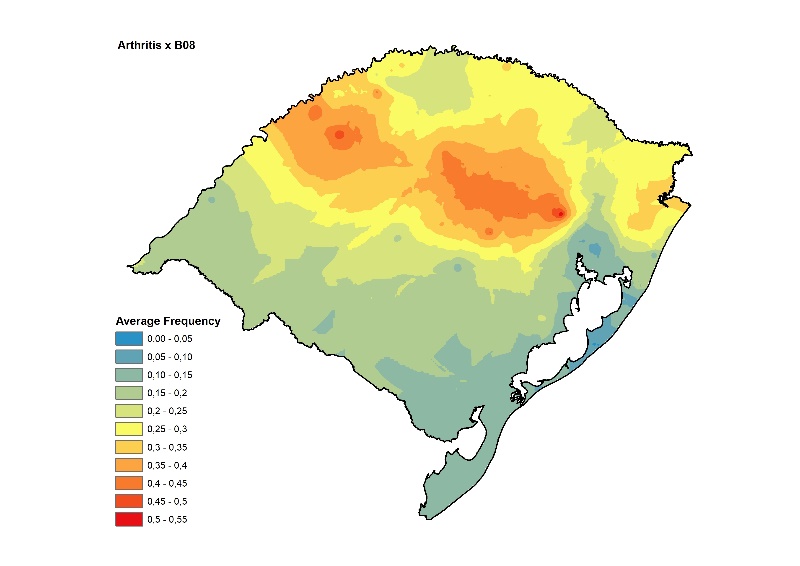

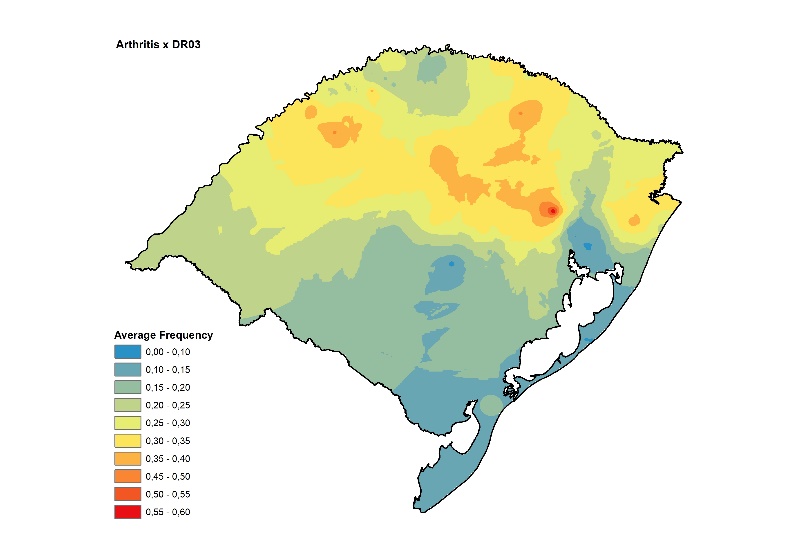


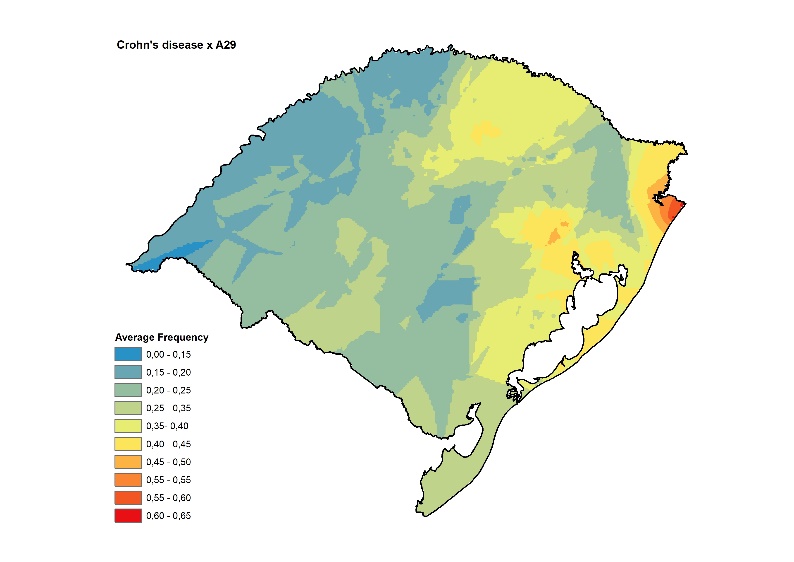

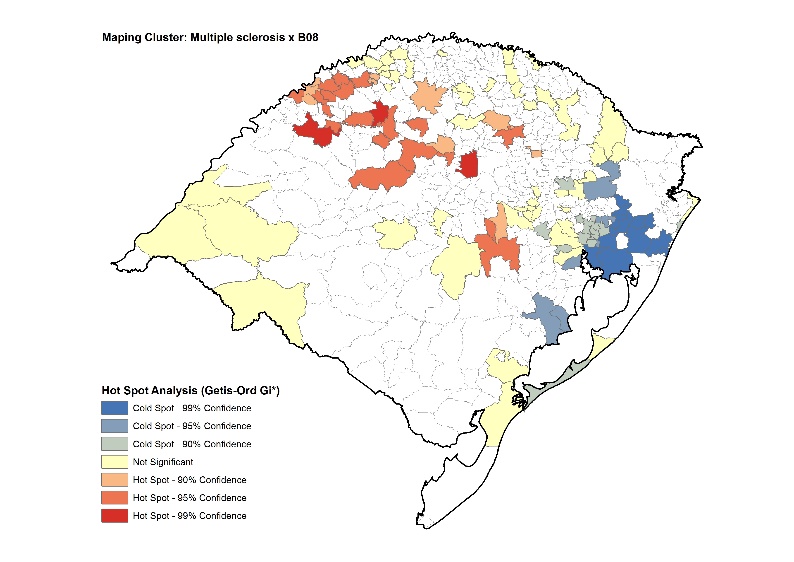

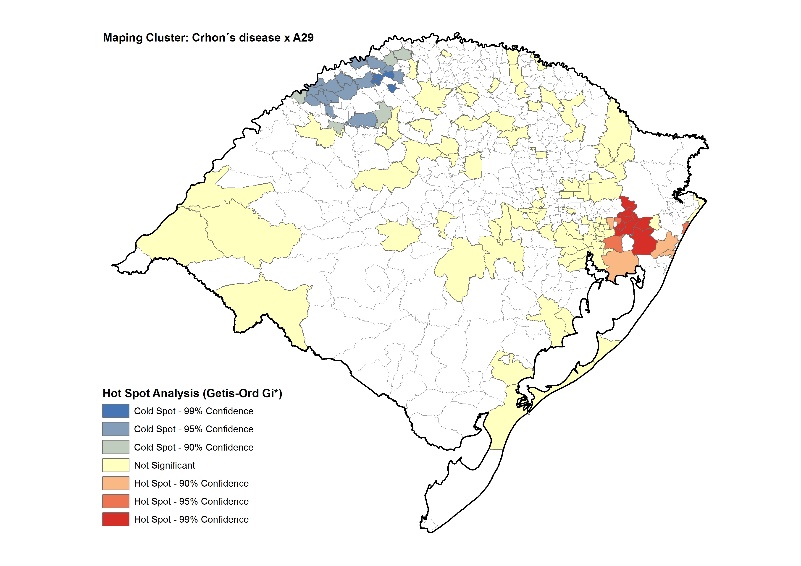

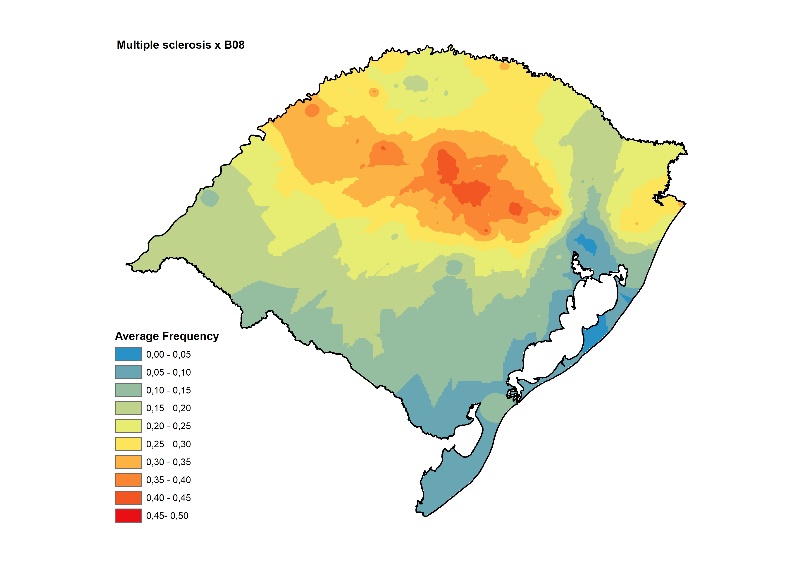


HLA-A*29 x CD

HLA-B*08 x MS


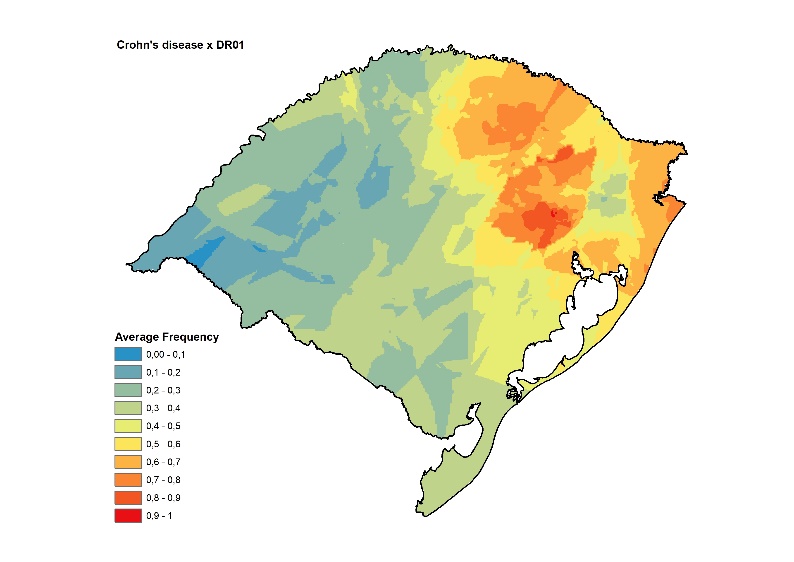

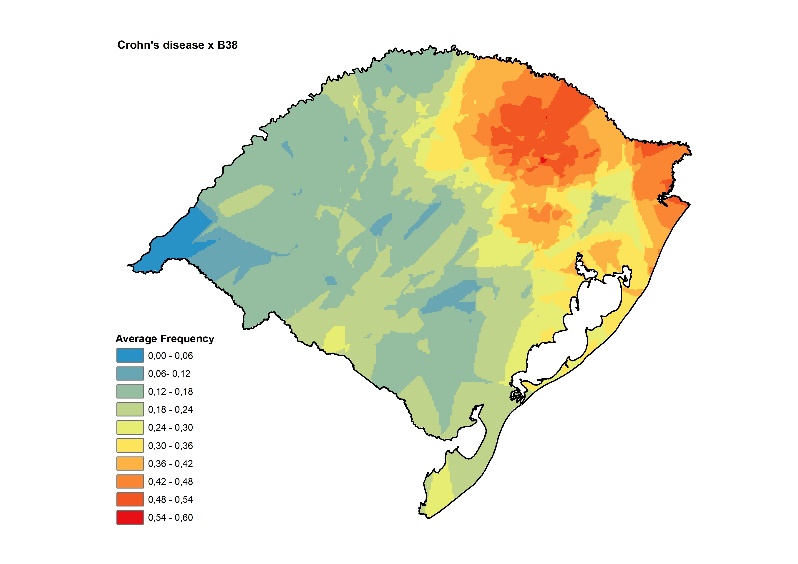

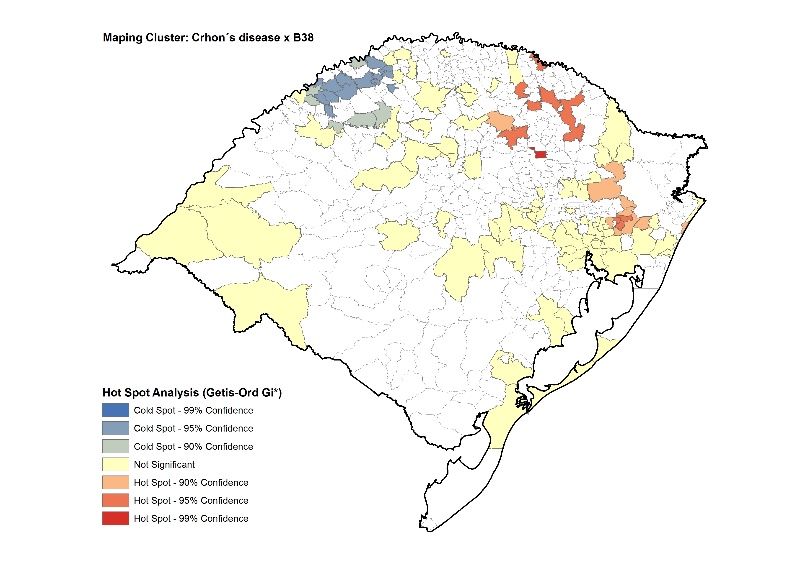

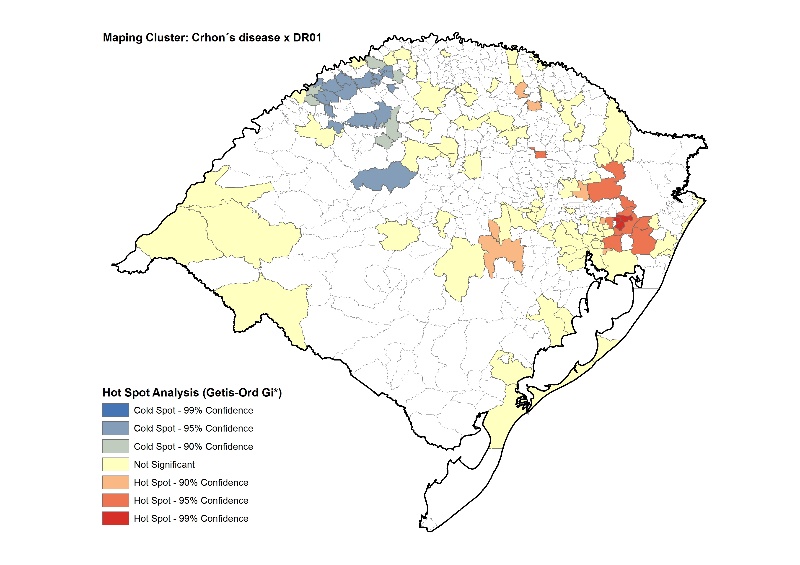


HLA-DRB1*01 x CD

HLA-B*38 x CD
