# Supplementary material for: Spatial analyzes of HLA data in Rio Grande do Sul, south Brazil: genetic structure and possible correlation with autoimmune diseases
Source: Int J Health Geogr. 2018 Sep 14;17:34. doi: 10.1186/s12942-018-0154-8 (PMC6137739; doi:10.1186/s12942-018-0154-8)
Supplement: Supplementary file 9 — Additional file 9. Spearman’s determination coefficient, Spearman’s correlation coefficient and root mean square error for each interpolated map. [file 12942_2018_154_MOESM9_ESM.docx]

**Additional file 9 – Spearman’s determination coefficient, Spearman’s correlation coefficient and root mean square error** **for each interpolated map**

| **Map** | **Figure** | ***ρ*^2^** | ***ρ*** | **RMSE** | **P** | **P*** |
| --- | --- | --- | --- | --- | --- | --- |
| SPC HLA-A | Figure 2 A | 0,9179 | 0,9581 | 0,1160 | 0,0003 | 0,0005 |
| SPC HLA-B | Figure 2 B | 0,9760 | 0,9879 | 0,0668 | 0,0001 | 0,0002 |
| SPC HLA-DRB1 | Figure 2 C | 0,8740 | 0,9349 | 0,1628 | 0,0006 | 0,0006 |
| SPC HLA-A, -B, -DRB1 | Figure 2 D | 0,9882 | 0,9941 | 0,0449 | <0,0001 | 0,0002 |
| HLA-A*01 | SM 2 | 0,9439 | 0,9716 | 0,0079 | 0,0002 | 0,0013 |
| HLA-A*02 | SM 2 | 0,9485 | 0,9739 | 0,0094 | 0,0002 | 0,0013 |
| HLA-A*03 | SM 2 | 0,6918 | 0,8318 | 0,0177 | 0,0017 | 0,0047 |
| HLA-A*11 | SM 2 | 0,3771 | 0,6141 | 0,0137 | 0,0063 | 0,0102 |
| HLA-A*23 | SM 2 | 0,2950 | 0,5431 | 0,0122 | 0,0091 | 0,0134 |
| HLA-A*24 | SM 2 | 0,6369 | 0,7981 | 0,0150 | 0,0022 | 0,0056 |
| HLA-A*25 | SM 2 | 0,1110 | 0,3331 | 0,0082 | 0,0286 | 0,0319 |
| HLA-A*26 | SM 2 | 0,6906 | 0,8310 | 0,0090 | 0,0017 | 0,0047 |
| HLA-A*29 | SM 2 | 0,9653 | 0,9825 | 0,0042 | 0,0001 | 0,0013 |
| HLA-A*30 | SM 2 | 0,9956 | 0,9978 | 0,0012 | <0,0001 | 0,0012 |
| HLA-A*31 | SM 2 | 0,9471 | 0,9732 | 0,0058 | 0,0002 | 0,0013 |
| HLA-A*32 | SM 2 | 0,3694 | 0,6078 | 0,0113 | 0,0065 | 0,0102 |
| HLA-A*33 | SM 2 | 0,5018 | 0,7084 | 0,0086 | 0,0038 | 0,0070 |
| HLA-A*34 | SM 2 | 0,3676 | 0,6063 | 0,0027 | 0,0066 | 0,0102 |
| HLA-A*36 | SM 2 | 0,8810 | 0,9386 | 0,0006 | 0,0005 | 0,0020 |
| HLA-A*66 | SM 2 | 0,5221 | 0,7226 | 0,0038 | 0,0035 | 0,0067 |
| HLA-A*68 | SM 2 | 0,0600 | 0,2448 | 0,0152 | 0,0519 | 0,0538 |
| HLA-A*69 | SM 2 | 0,5835 | 0,7639 | 0,0016 | 0,0028 | 0,0061 |
| HLA-A*74 | SM 2 | 0,3263 | 0,5713 | 0,0039 | 0,0079 | 0,0119 |
| HLA-A*80 | SM 2 | 0,1232 | 0,3510 | 0,0015 | 0,0257 | 0,0306 |
| HLA-B*07 | SM 2 | 0,8480 | 0,9209 | 0,0130 | 0,0007 | 0,0024 |
| HLA-B*08 | SM 2 | 0,8965 | 0,9468 | 0,0097 | 0,0005 | 0,0018 |
| HLA-B*13 | SM 2 | 0,8497 | 0,9218 | 0,0052 | 0,0007 | 0,0024 |
| HLA-B*14 | SM 2 | 0,5751 | 0,7583 | 0,0131 | 0,0029 | 0,0061 |
| HLA-B*15 | SM 2 | 0,9713 | 0,9855 | 0,0050 | 0,0001 | 0,0013 |
| HLA-B*18 | SM 2 | 0,6064 | 0,7787 | 0,0112 | 0,0025 | 0,0057 |
| HLA-B*27 | SM 2 | 0,3927 | 0,6267 | 0,0091 | 0,0059 | 0,0098 |
| HLA-B*35 | SM 2 | 0,9736 | 0,9867 | 0,0055 | 0,0001 | 0,0013 |
| HLA-B*37 | SM 2 | 0,1802 | 0,4245 | 0,0062 | 0,0169 | 0,0213 |
| HLA-B*38 | SM 2 | 0,4666 | 0,6831 | 0,0085 | 0,0044 | 0,0075 |
| HLA-B*39 | SM 2 | 0,1139 | 0,3375 | 0,0116 | 0,0279 | 0,0316 |
| HLA-B*40 | SM 2 | 0,6254 | 0,7908 | 0,0103 | 0,0023 | 0,0056 |
| HLA-B*41 | SM 2 | 0,9548 | 0,9771 | 0,0018 | 0,0002 | 0,0013 |
| HLA-B*42 | SM 2 | 0,2724 | 0,5219 | 0,0031 | 0,0101 | 0,0138 |
| HLA-B*44 | SM 2 | 0,9562 | 0,9778 | 0,0076 | 0,0002 | 0,0013 |
| HLA-B*45 | SM 2 | 0,7135 | 0,8447 | 0,0041 | 0,0016 | 0,0047 |
| HLA-B*47 | SM 2 | 0,1085 | 0,3294 | 0,0024 | 0,0293 | 0,0321 |
| **Map** | **Figure** | ***ρ*^2^** | ***ρ*** | **RMSE** | **P** | **P*** |
| HLA-B*48 | SM 2 | 0,5416 | 0,7359 | 0,0041 | 0,0033 | 0,0064 |
| HLA-B*49 | SM 2 | 0,1329 | 0,3646 | 0,0113 | 0,0237 | 0,0288 |
| HLA-B*50 | SM 2 | 0,4903 | 0,7002 | 0,0073 | 0,0040 | 0,0072 |
| HLA-B*51 | SM 2 | 0,9029 | 0,9502 | 0,0099 | 0,0004 | 0,0018 |
| HLA-B*52 | SM 2 | 0,1811 | 0,4256 | 0,0068 | 0,0168 | 0,0213 |
| HLA-B*53 | SM 2 | 0,6895 | 0,8304 | 0,0050 | 0,0017 | 0,0047 |
| HLA-B*55 | SM 2 | 0,5565 | 0,7460 | 0,0064 | 0,0031 | 0,0064 |
| HLA-B*56 | SM 2 | 0,0284 | 0,1686 | 0,0065 | 0,0963 | 0,0978 |
| HLA-B*57 | SM 2 | 0,2398 | 0,4896 | 0,0115 | 0,0119 | 0,0159 |
| HLA-B*58 | SM 2 | 0,9585 | 0,9790 | 0,0025 | 0,0002 | 0,0013 |
| HLA-B*67 | SM 2 | 0,2818 | 0,5309 | 0,0001 | 0,0097 | 0,0137 |
| HLA-B*73 | SM 2 | 0,0726 | 0,2694 | 0,0019 | 0,0435 | 0,0462 |
| HLA-B*78 | SM 2 | 0,0914 | 0,3023 | 0,0009 | 0,0348 | 0,0376 |
| HLA-B*81 | SM 2 | 0,2728 | 0,5223 | 0,0020 | 0,0101 | 0,0138 |
| HLA-DRB1*01 | SM 2 | 0,5417 | 0,7360 | 0,0144 | 0,0033 | 0,0064 |
| HLA-DRB1*03 | SM 2 | 0,2827 | 0,5317 | 0,0174 | 0,0096 | 0,0137 |
| HLA-DRB1*04 | SM 2 | 0,9817 | 0,9908 | 0,0058 | 0,0001 | 0,0013 |
| HLA-DRB1*07 | SM 2 | 0,6901 | 0,8307 | 0,0150 | 0,0017 | 0,0047 |
| HLA-DRB1*08 | SM 2 | 0,6843 | 0,8272 | 0,0113 | 0,0018 | 0,0047 |
| HLA-DRB1*09 | SM 2 | 0,9002 | 0,9488 | 0,0037 | 0,0004 | 0,0018 |
| HLA-DRB1*10 | SM 2 | 0,4716 | 0,6867 | 0,0062 | 0,0043 | 0,0075 |
| HLA-DRB1*11 | SM 2 | 0,9783 | 0,9891 | 0,0052 | 0,0001 | 0,0013 |
| HLA-DRB1*12 | SM 2 | 0,9170 | 0,9576 | 0,0032 | 0,0004 | 0,0018 |
| HLA-DRB1*13 | SM 2 | 0,6144 | 0,7838 | 0,0177 | 0,0024 | 0,0057 |
| HLA-DRB1*14 | SM 2 | 0,1186 | 0,3443 | 0,0149 | 0,0267 | 0,0311 |
| HLA-DRB1*15 | SM 2 | 0,8957 | 0,9464 | 0,0105 | 0,0005 | 0,0018 |
| HLA-DRB1*16 | SM 2 | 0,1411 | 0,3757 | 0,0135 | 0,0222 | 0,0274 |
| HLA-A*01~B*08~DRB1*03 | SM 5 | 0,7909 | 0,8893 | 0,0065 | 0,0010 | 0,0017 |
| HLA-A*03~B*07~DRB1*15 | SM 5 | 0,8950 | 0,9460 | 0,0031 | 0,0005 | 0,0011 |
| HLA-A*29~B*44~DRB1*07 | SM 5 | 0,9758 | 0,9878 | 0,0012 | 0,0001 | 0,0005 |
| HLA-A*02~B*07~DRB1*15 | SM 5 | 0,0968 | 0,3111 | 0,0068 | 0,0329 | 0,0329 |
| HLA-A*02~B*35~DRB1*01 | SM 5 | 0,1570 | 0,3962 | 0,0059 | 0,0197 | 0,0247 |
| HLA-B*08 x RA | SM 8 | 0,9211 | 0,9598 | 0,0335 | 0,0003 | 0,0010 |
| HLA-DRB1*03 x RA | SM 8 | 0,9142 | 0,9561 | 0,0341 | 0,0004 | 0,0010 |
| HLA-B*08 x MS | SM 8 | 0,8822 | 0,9393 | 0,0445 | 0,0005 | 0,0010 |
| HLA-A*29 x CD | SM 8 | 0,2041 | 0,4517 | 0,0886 | 0,0146 | 0,0146 |
| HLA-B*38 x CD | SM 8 | 0,3353 | 0,5791 | 0,0892 | 0,0076 | 0,0114 |
| HLA-DRB1*01 x CD | SM 8 | 0,2393 | 0,4892 | 0,1040 | 0,0120 | 0,0144 |
| *ρ^2^* = Spearman’s determination coefficient; *ρ* = Spearman’s correlation coefficient; RMSD = root mean square error; P = unadjusted; P* = P-value after false discovery rate (FDR) correction. | | | | | | |
