# Supplementary material for: Spatial analyzes of HLA data in Rio Grande do Sul, south Brazil: genetic structure and possible correlation with autoimmune diseases
Source: Int J Health Geogr. 2018 Sep 14;17:34. doi: 10.1186/s12942-018-0154-8 (PMC6137739; doi:10.1186/s12942-018-0154-8)

**Additional file 10 – Scatterplot and coefficient of determination (R^2^) for each interpolated map**

Allelic Frequency – HLA-A (Supplementary Material 3)


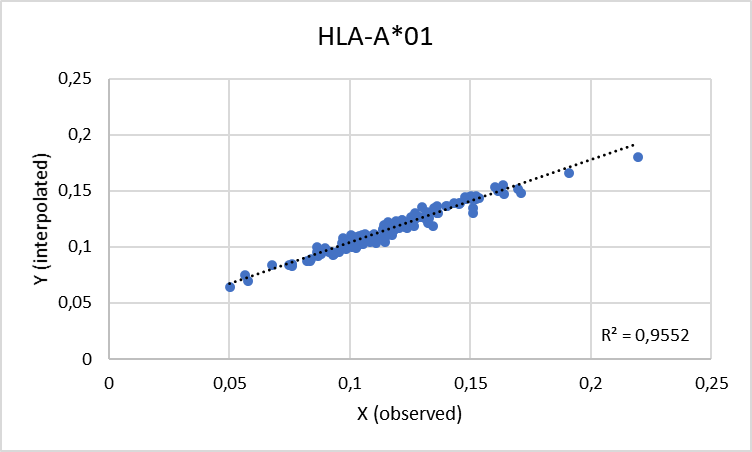

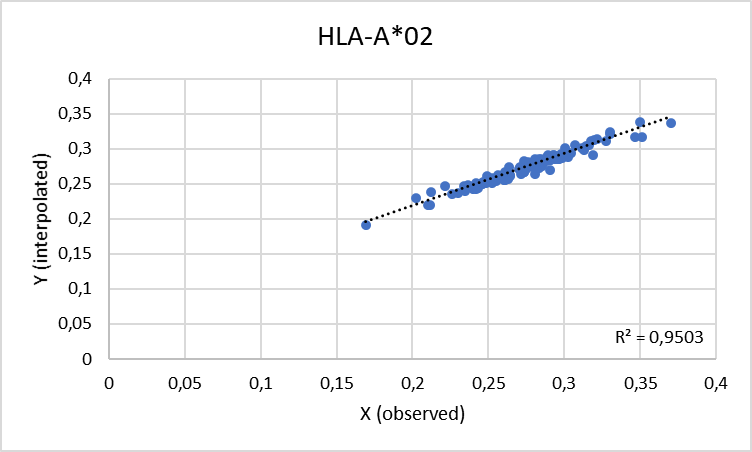


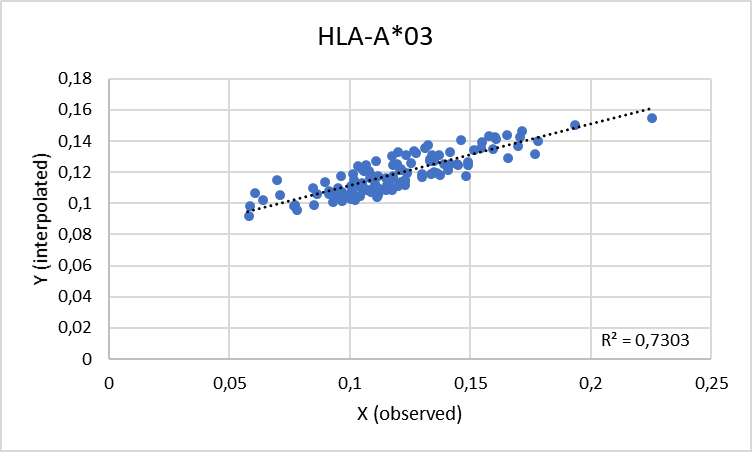

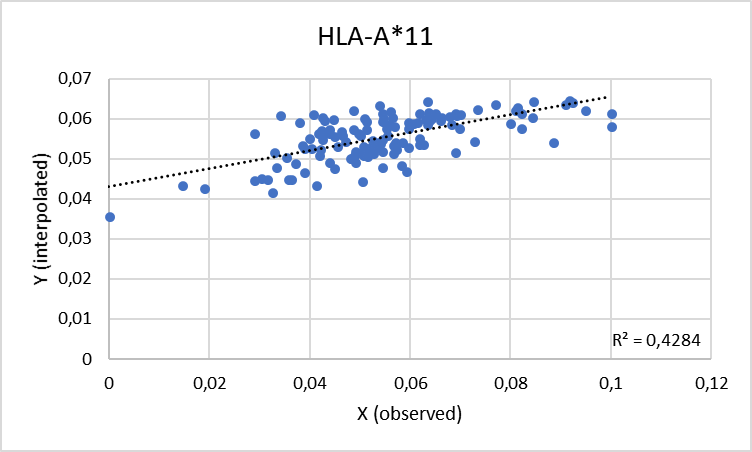


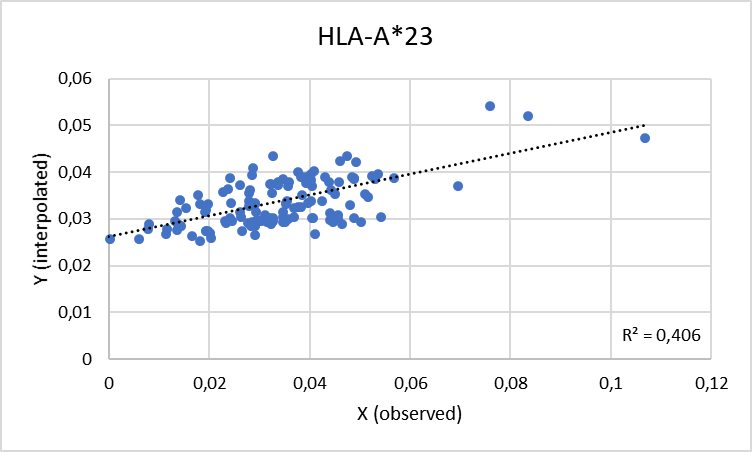

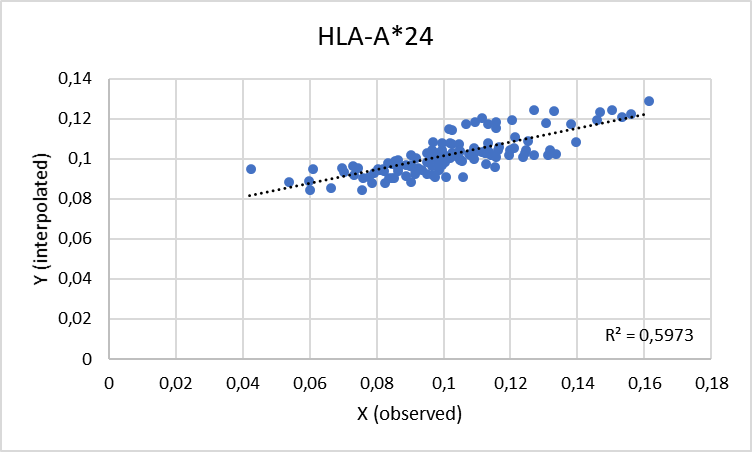


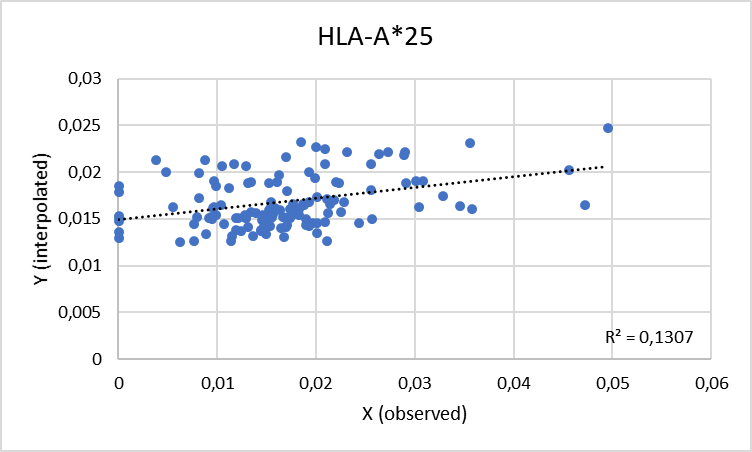

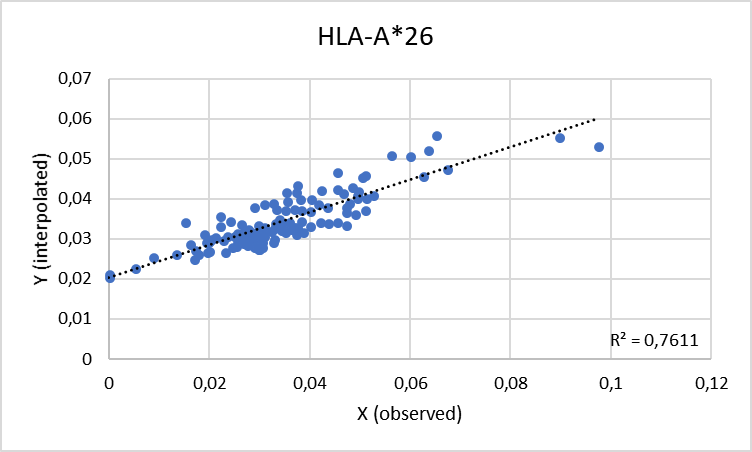


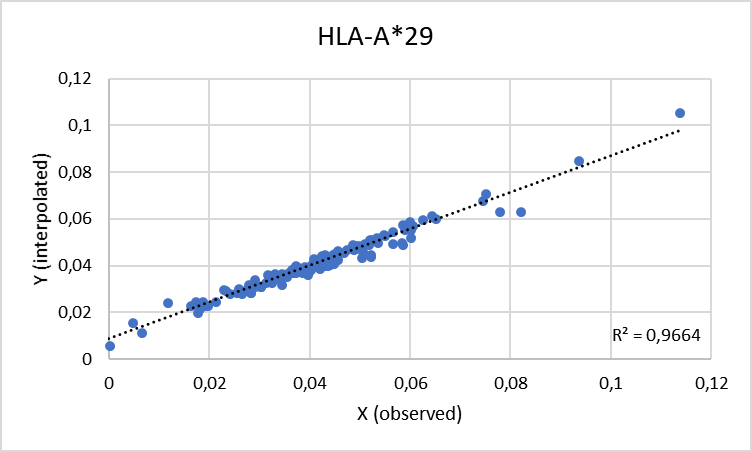

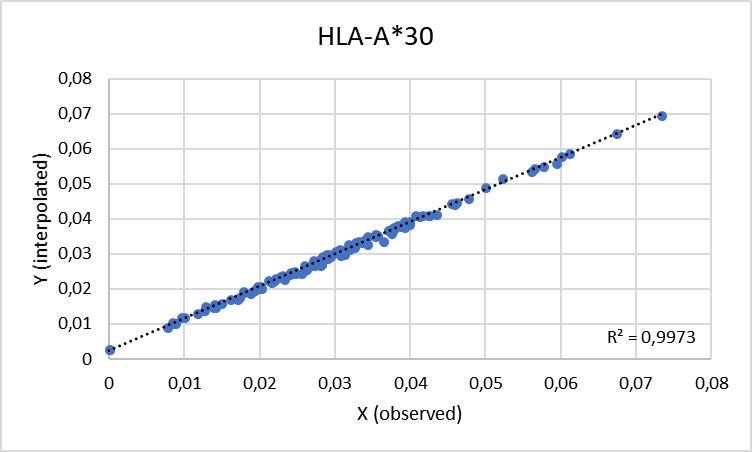


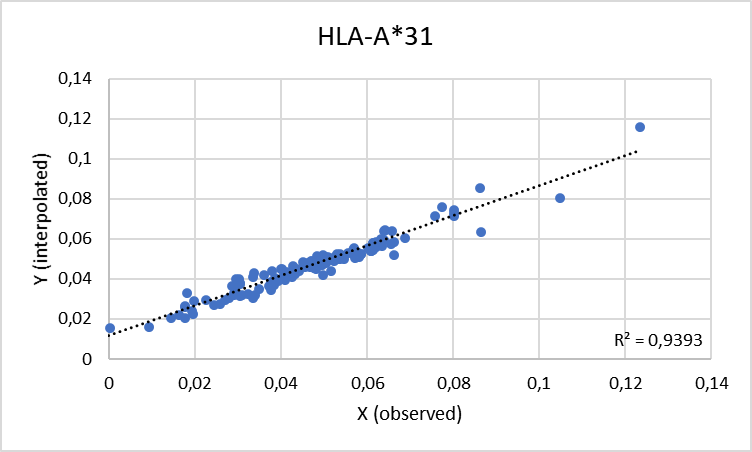

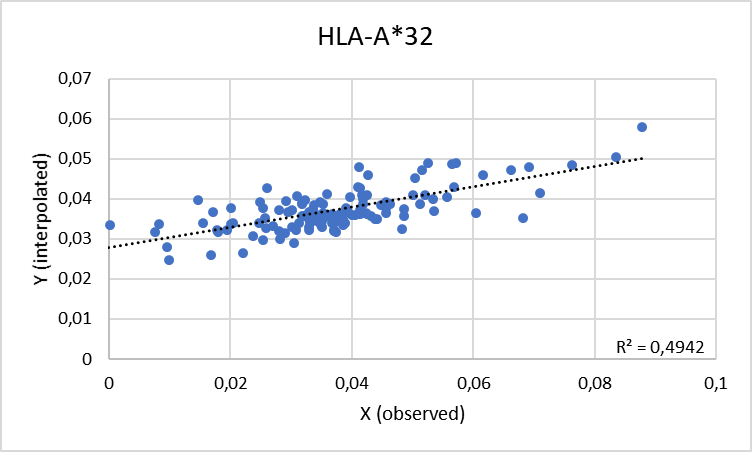


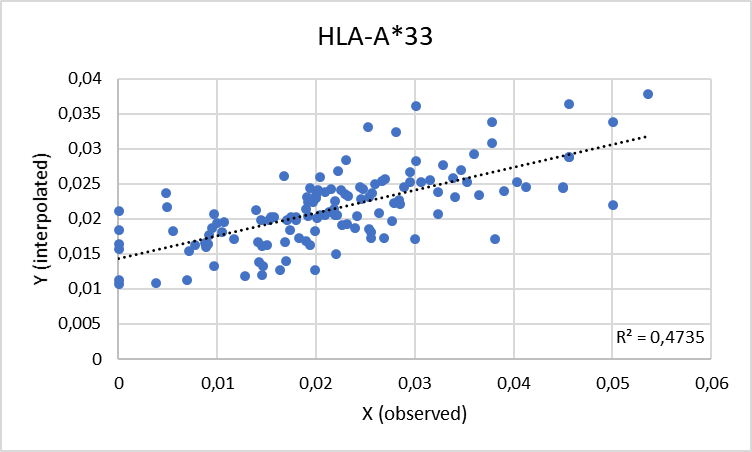

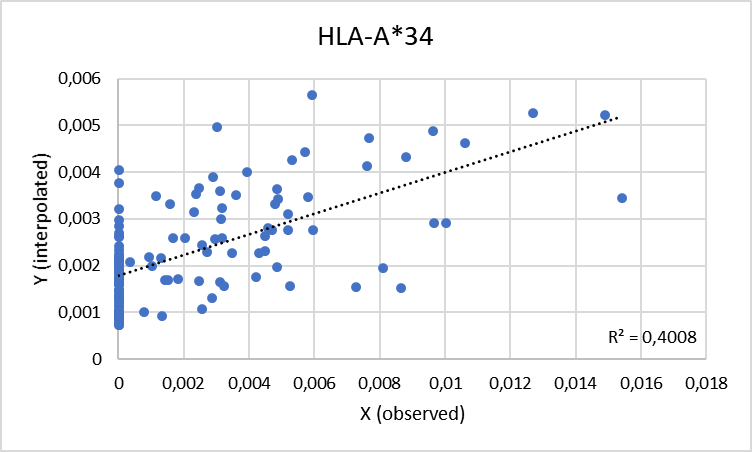


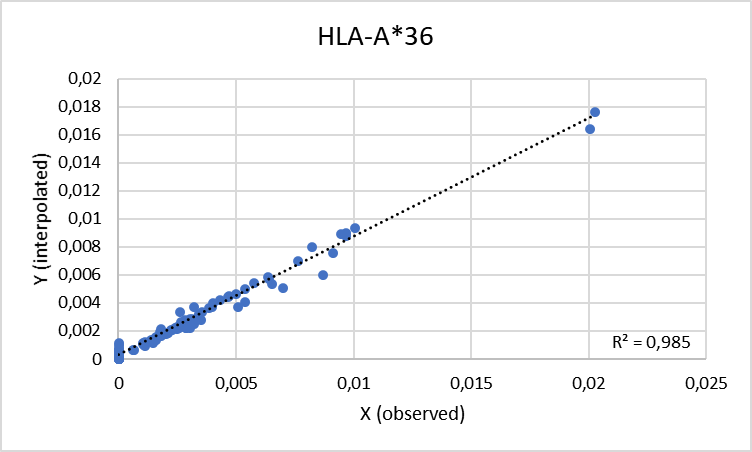

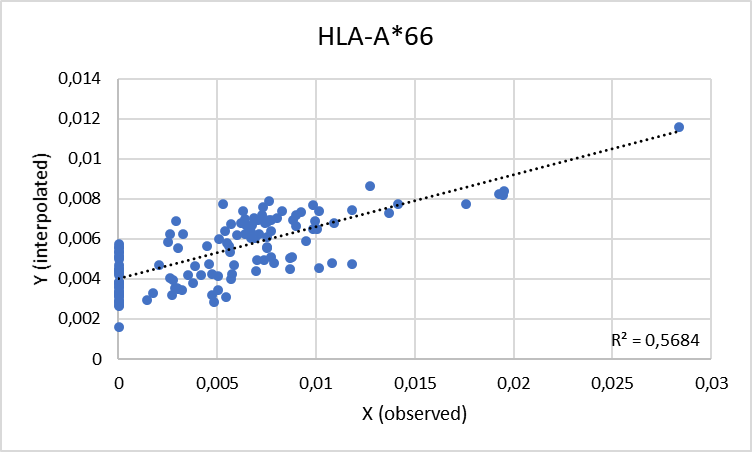


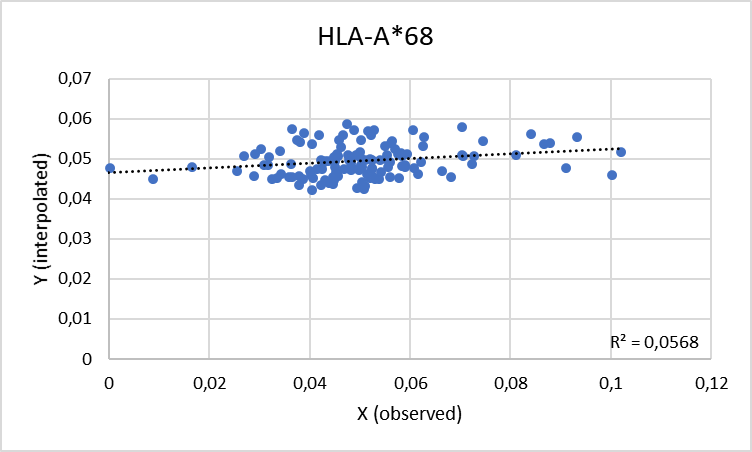

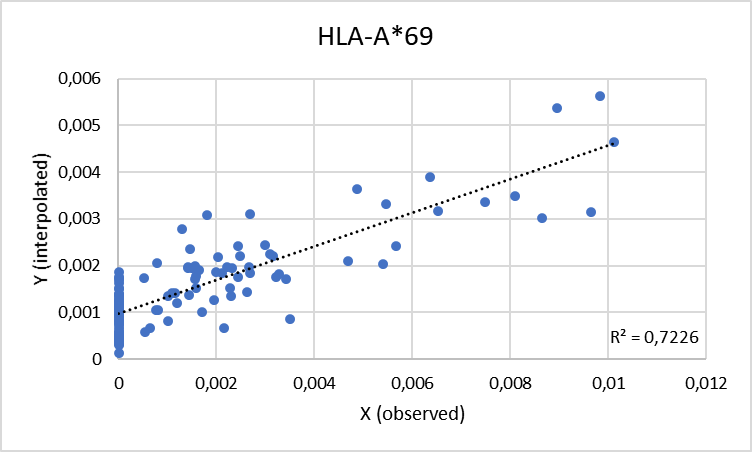


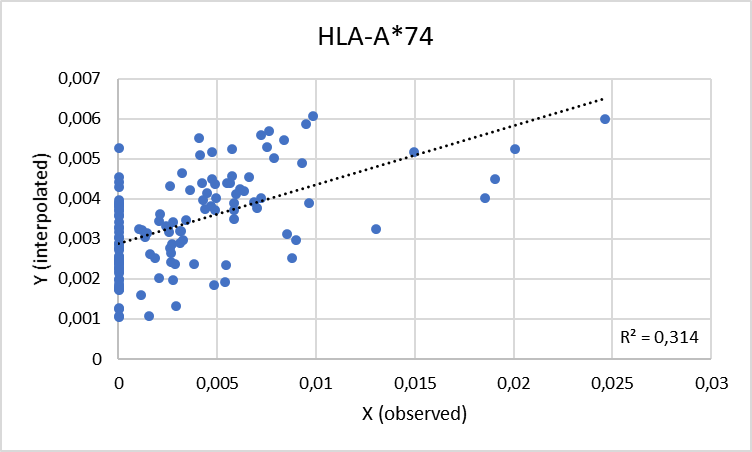

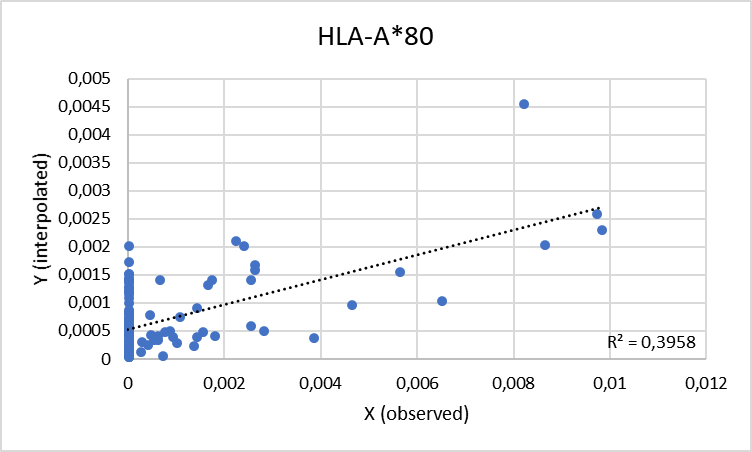


Allelic Frequency – HLA-B (Supplementary Material 3)


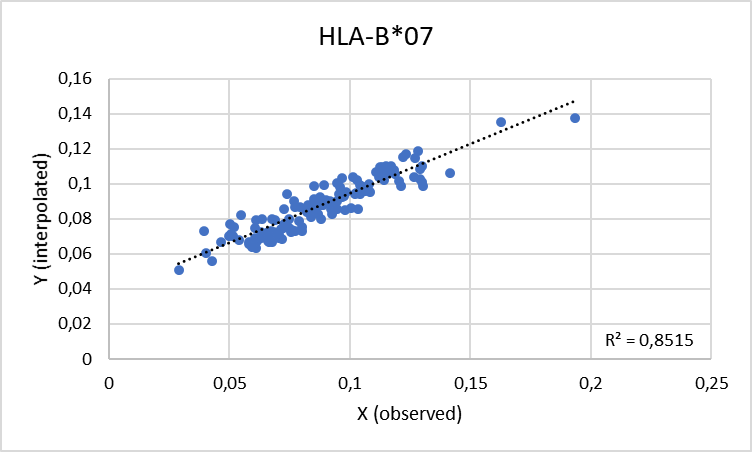

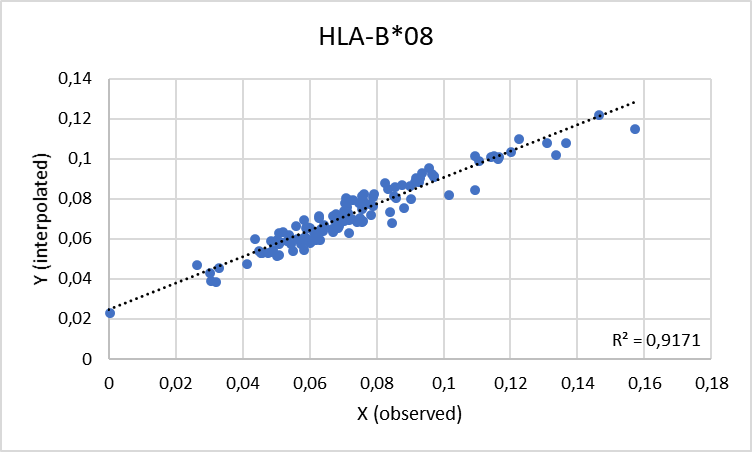


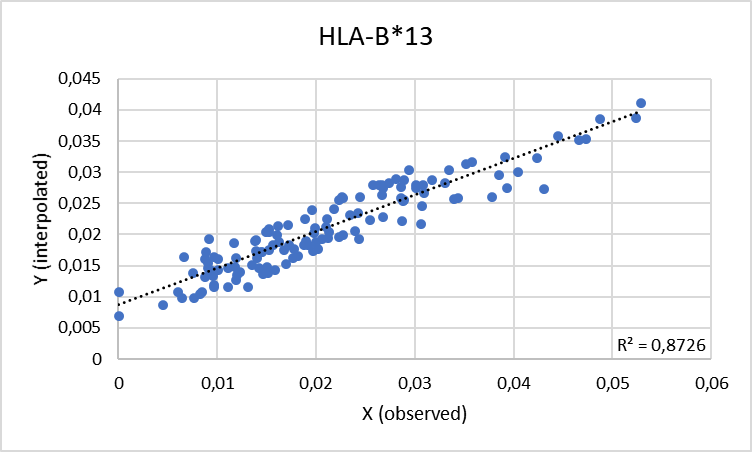

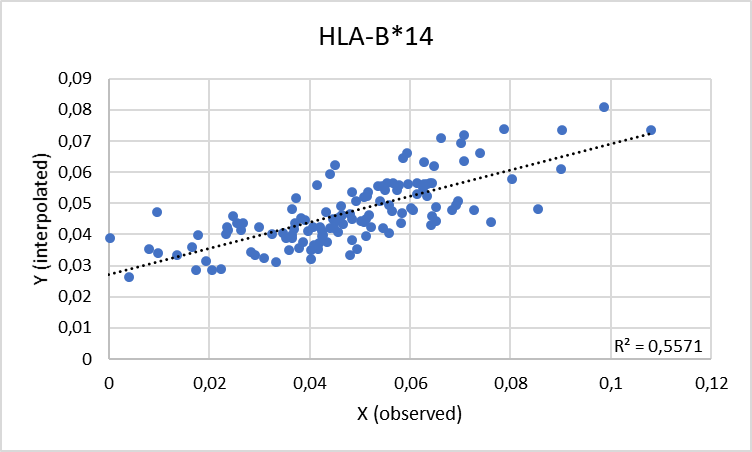


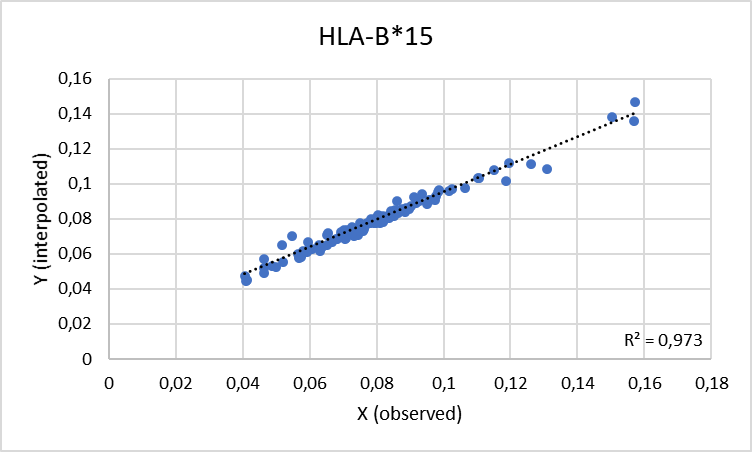

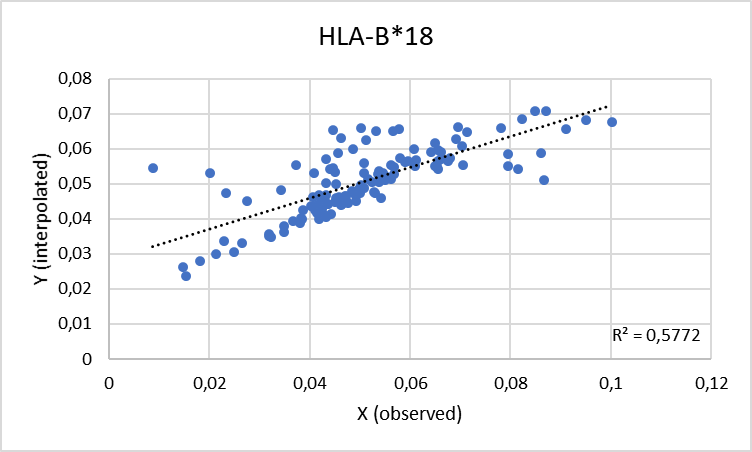


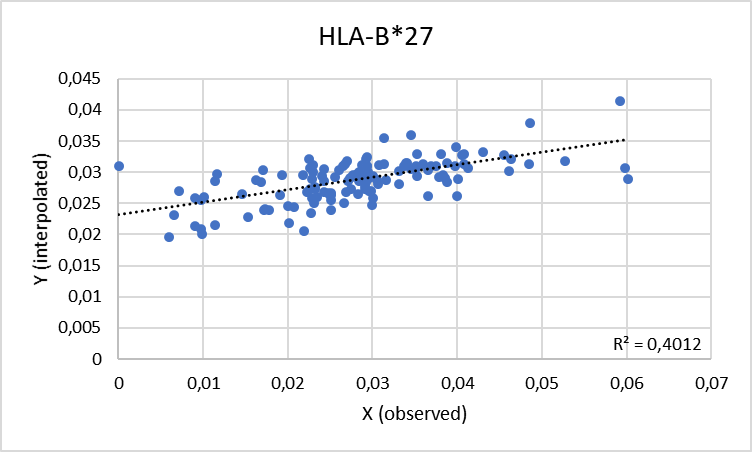

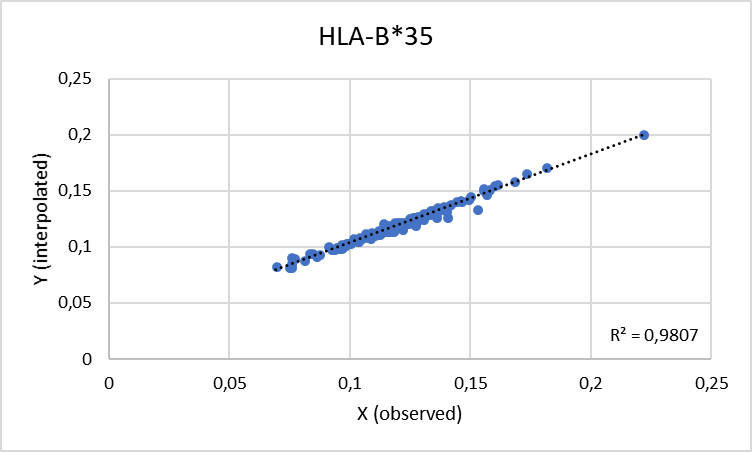


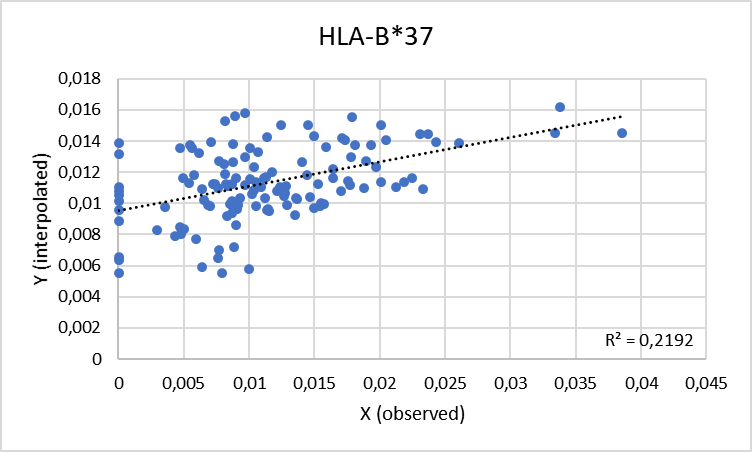

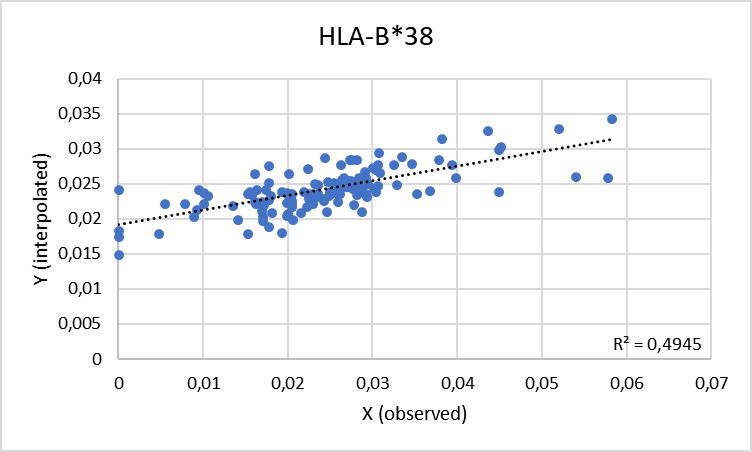


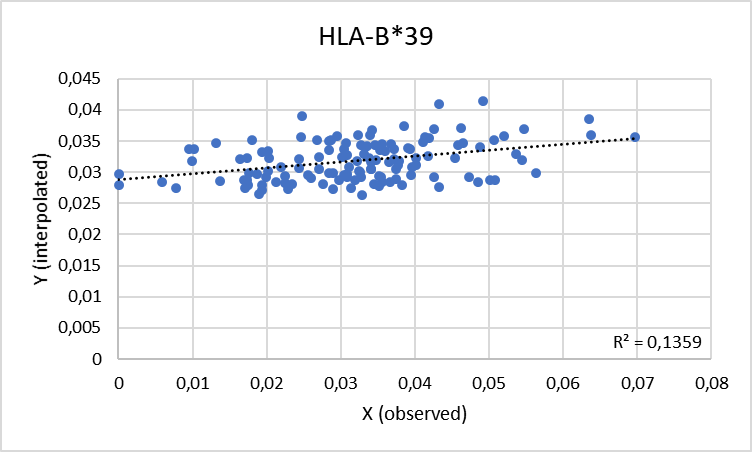

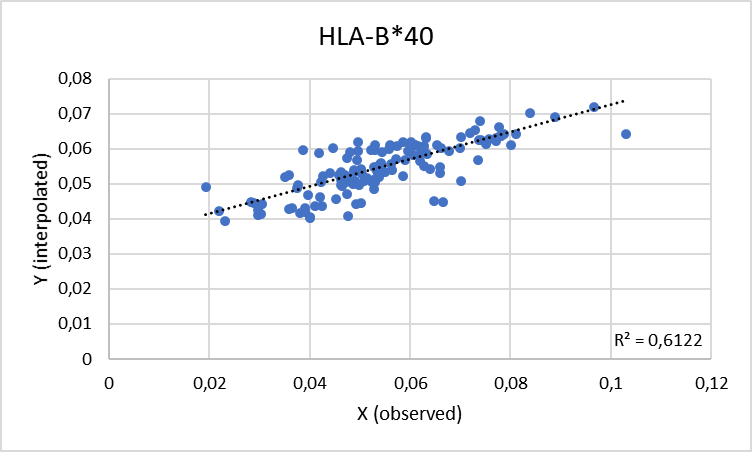


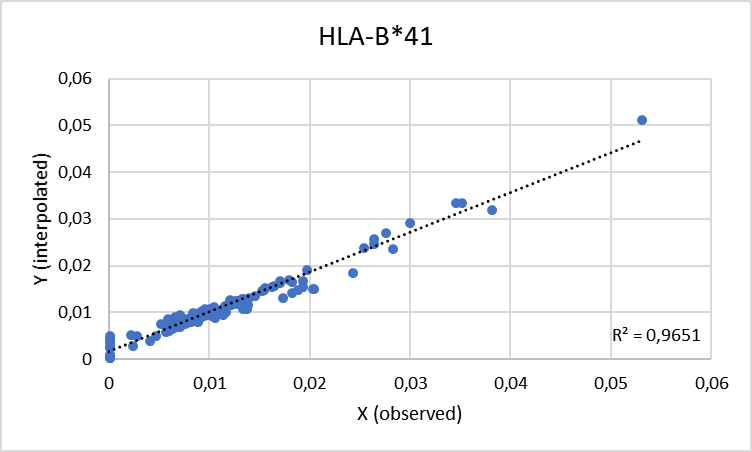

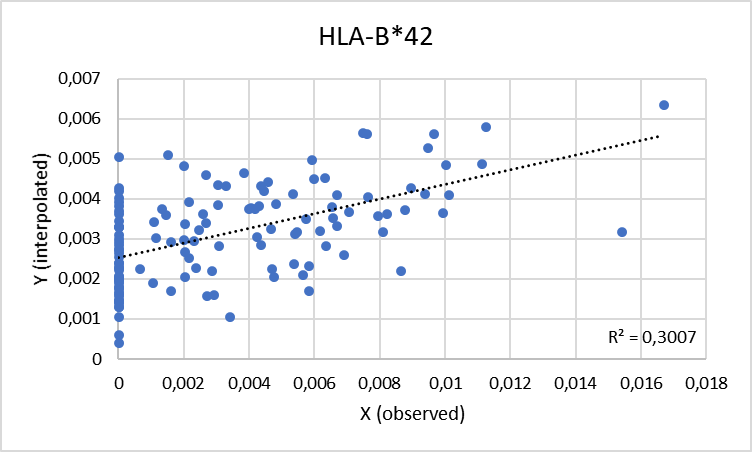


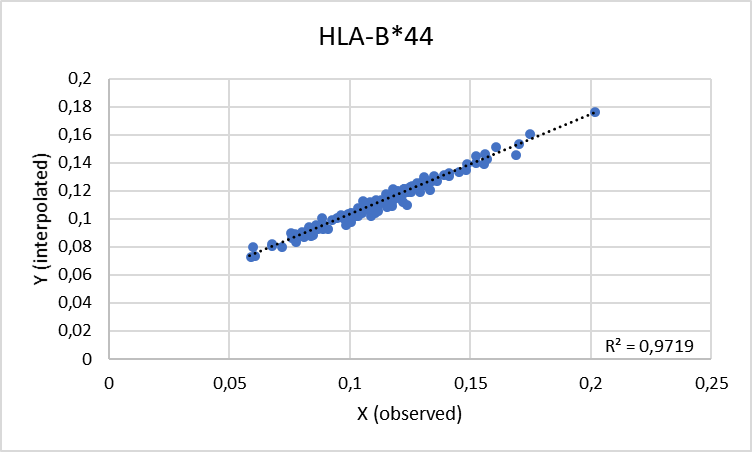

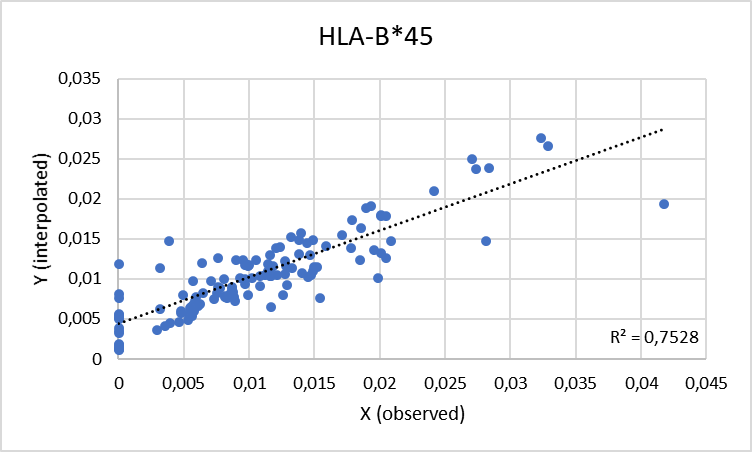


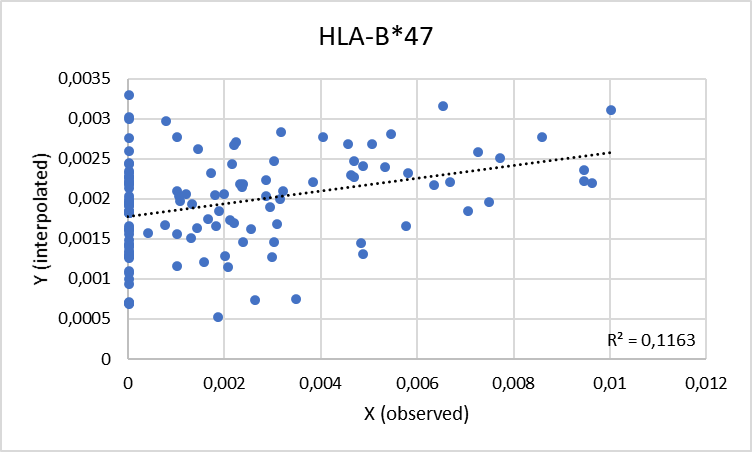

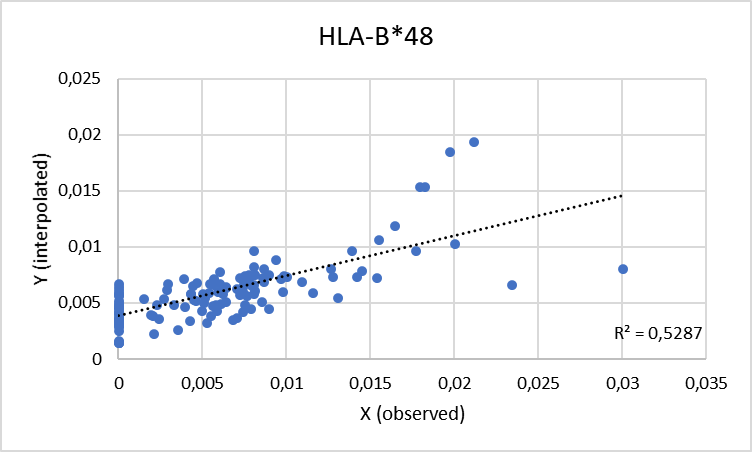


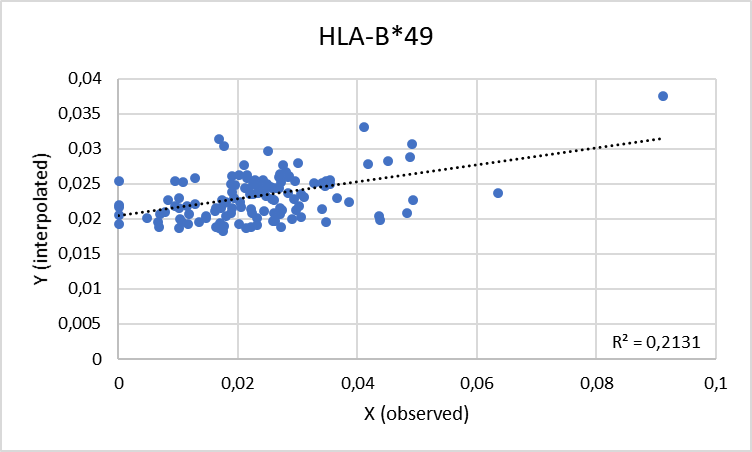

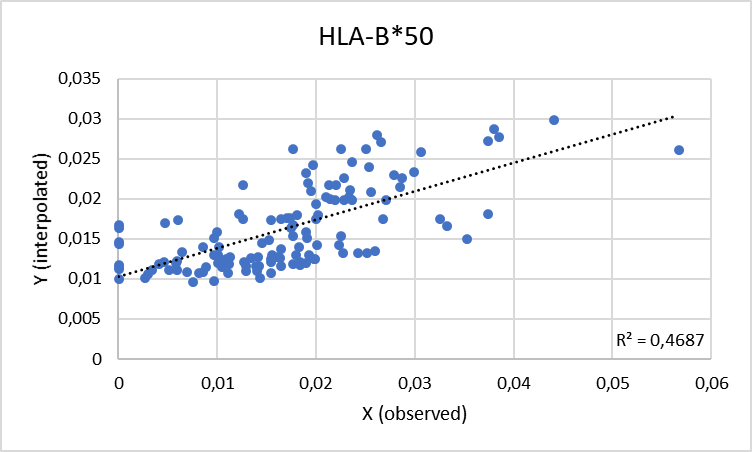


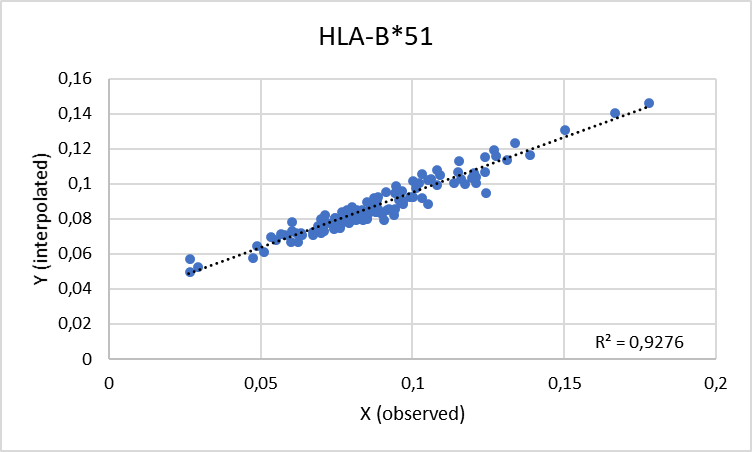

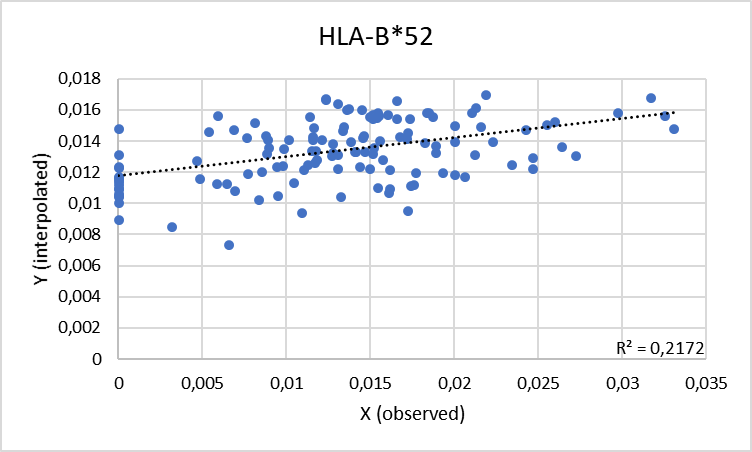


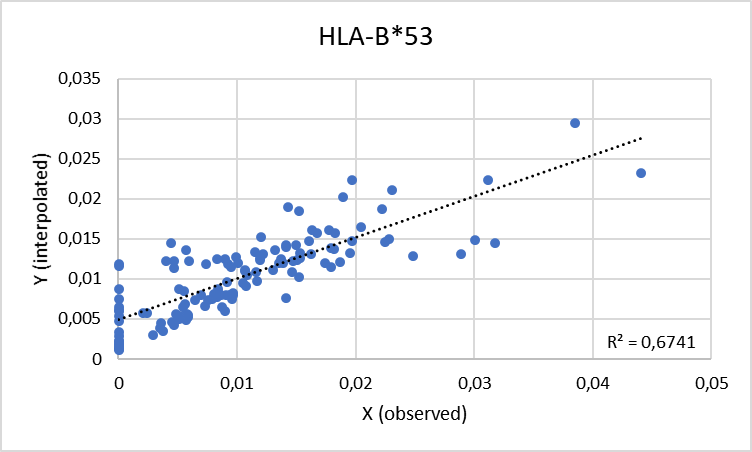

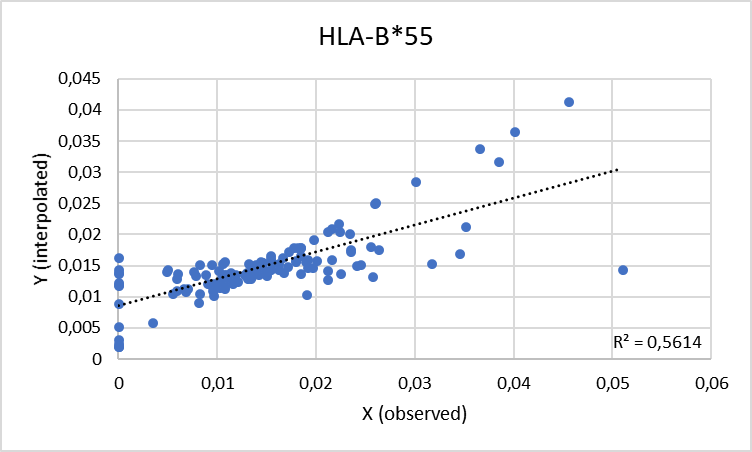


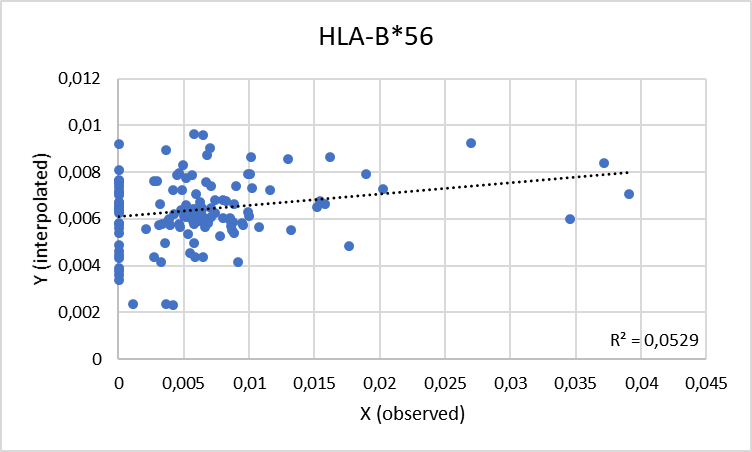

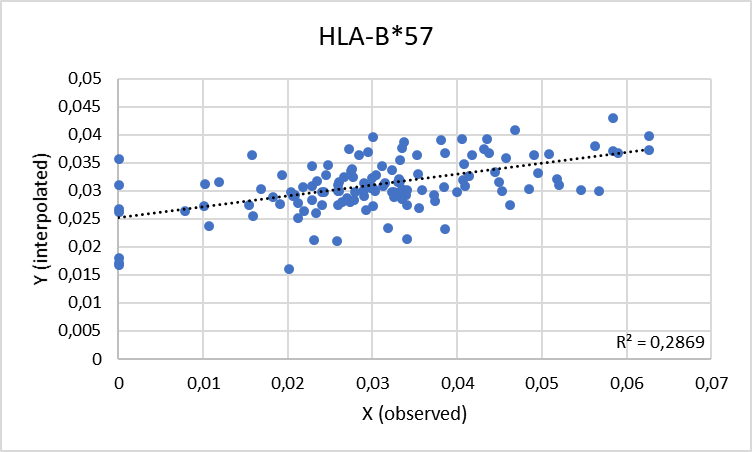


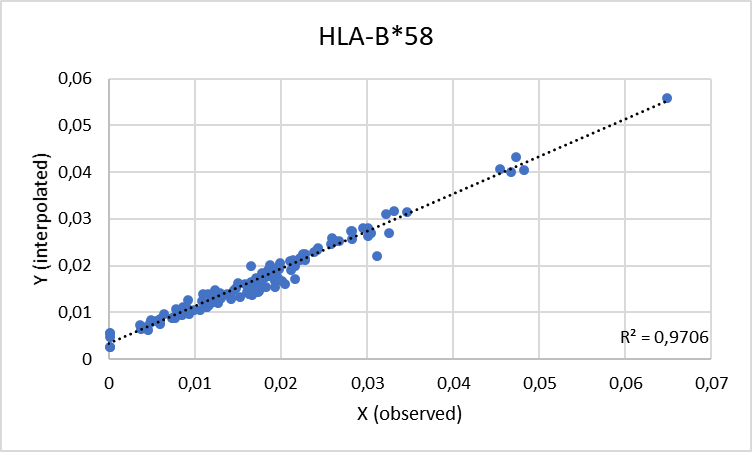

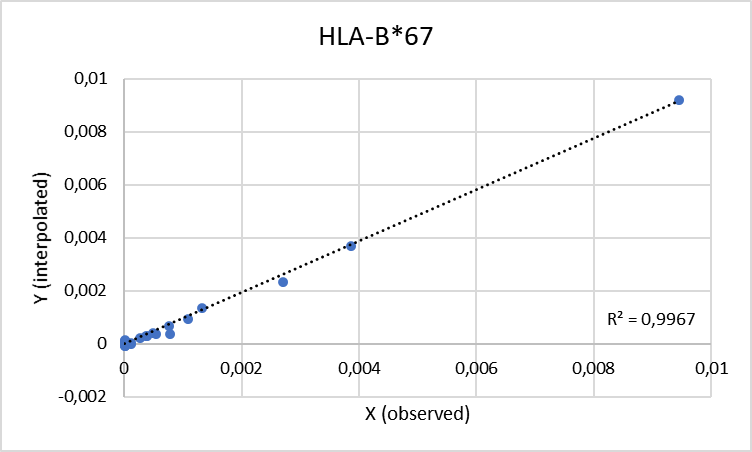


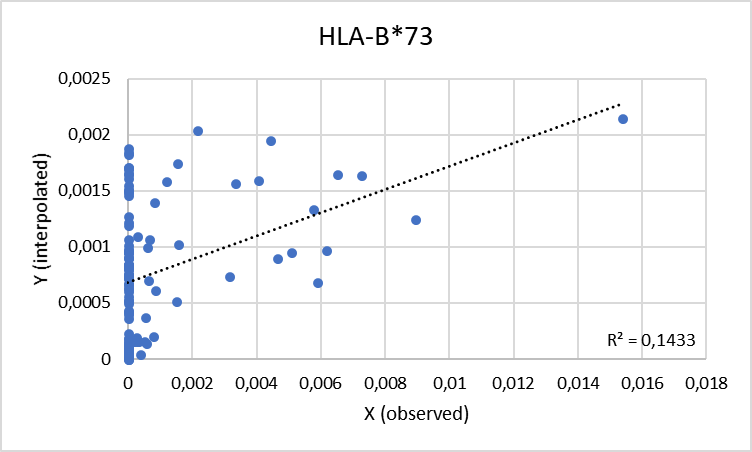

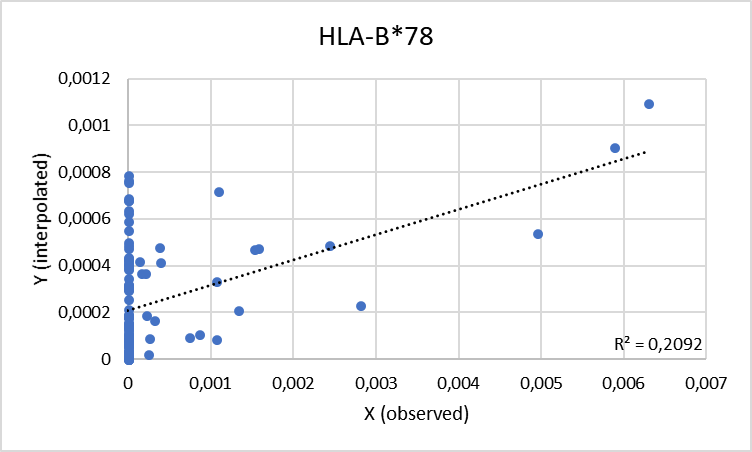


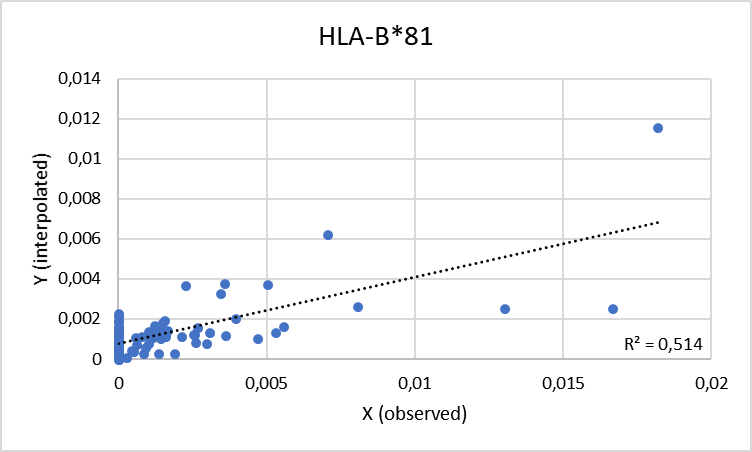


Allelic Frequency – HLA-DRB1 (Supplementary Material 3)


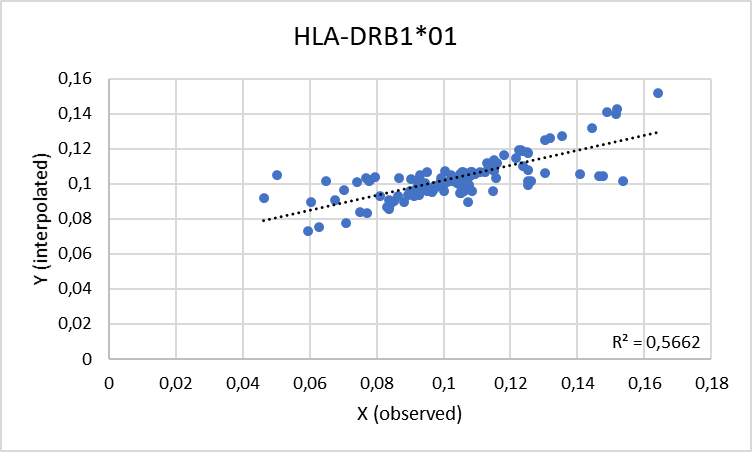

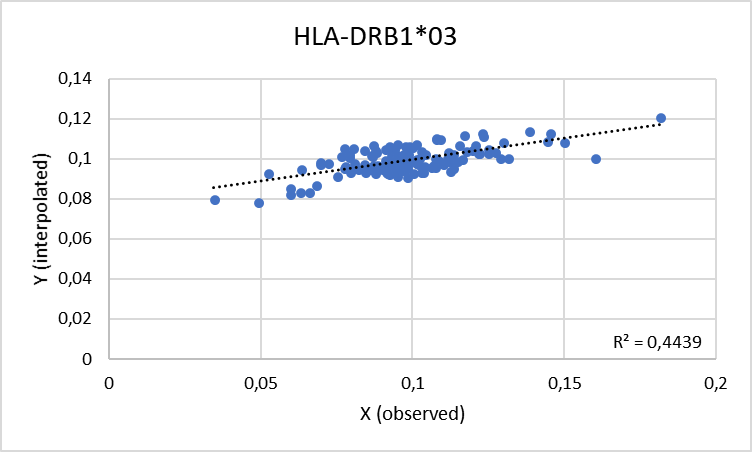


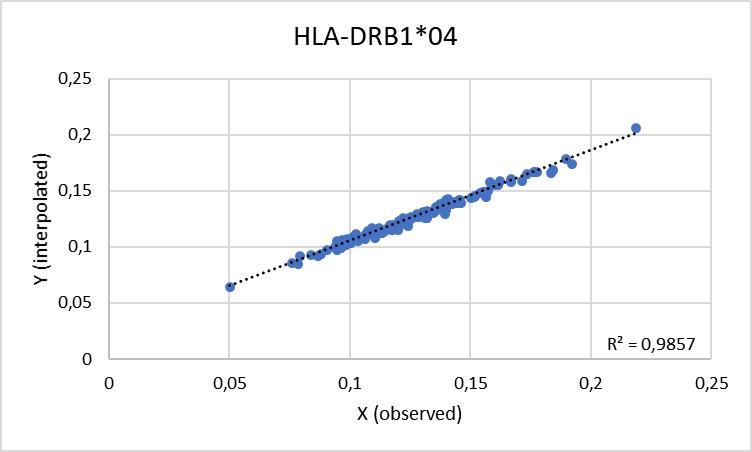

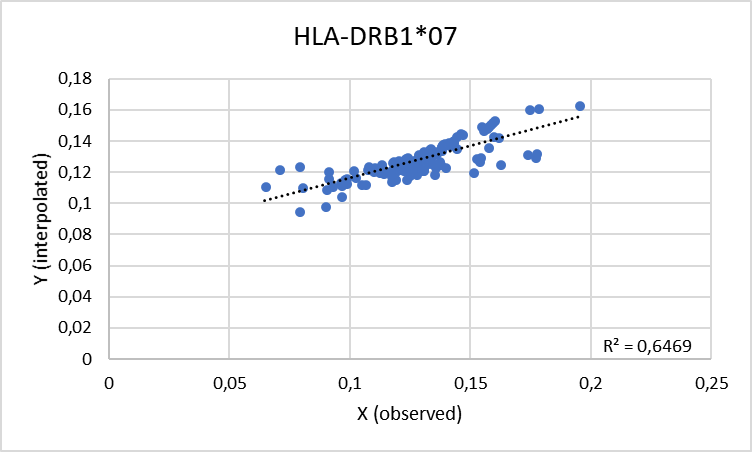


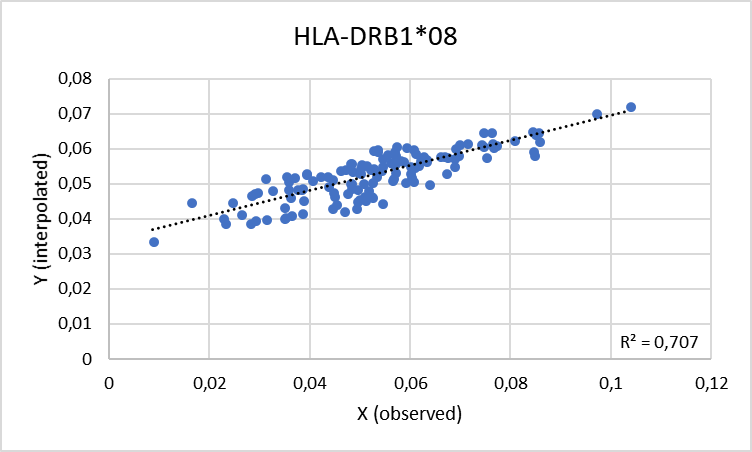

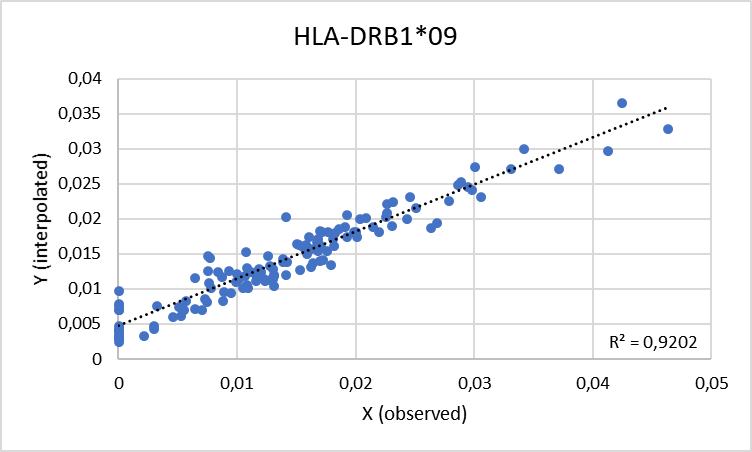


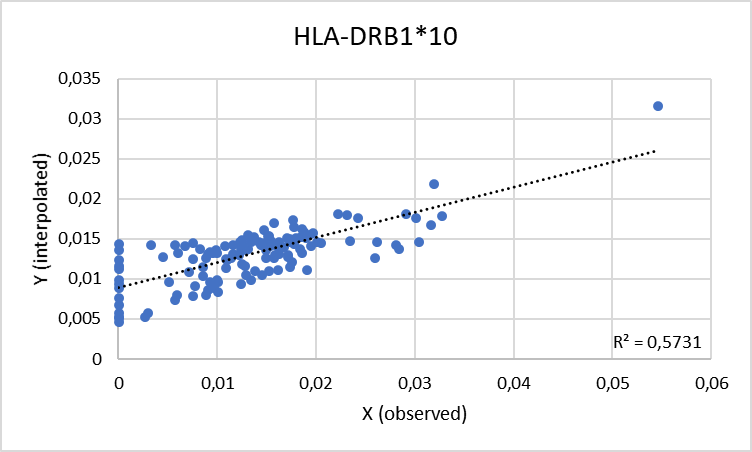

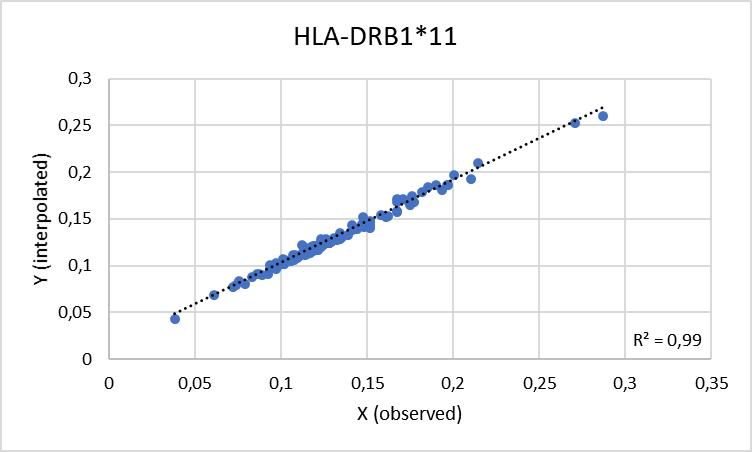


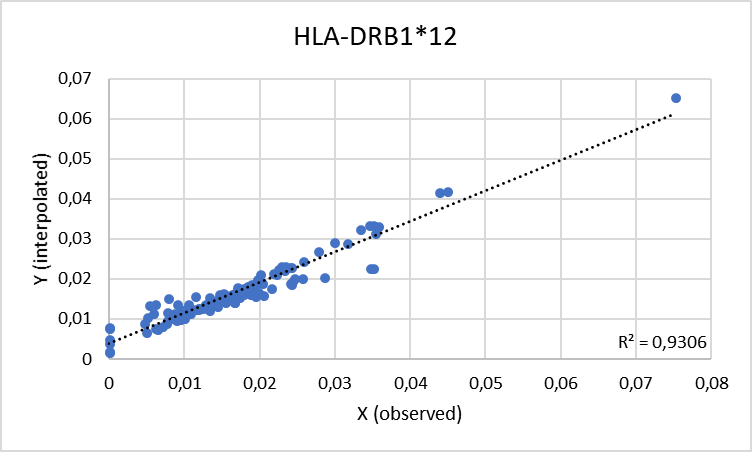

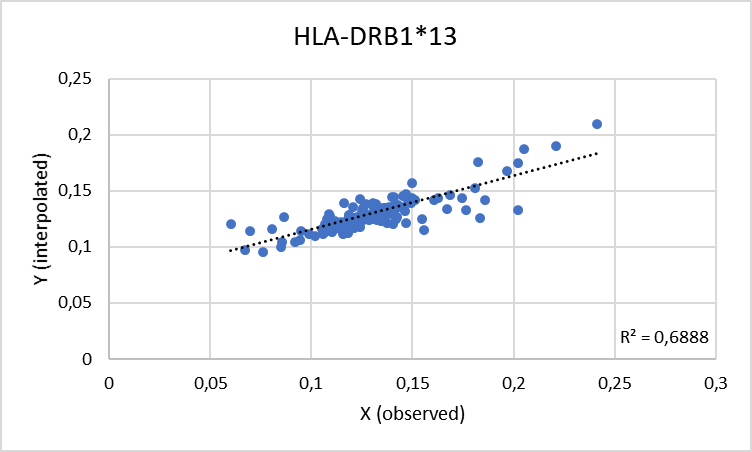


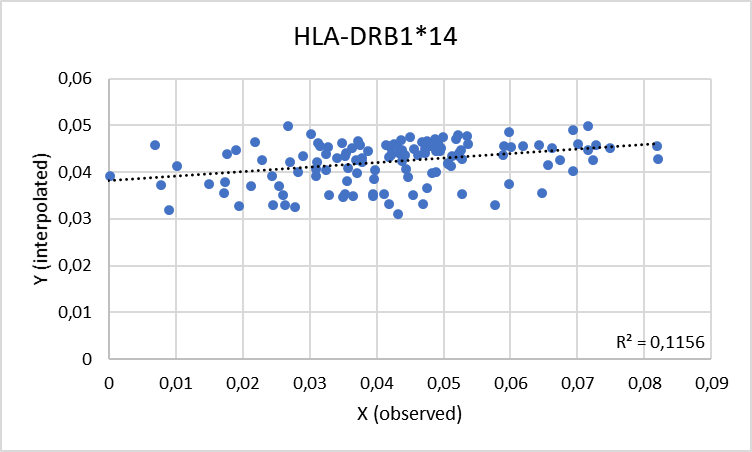

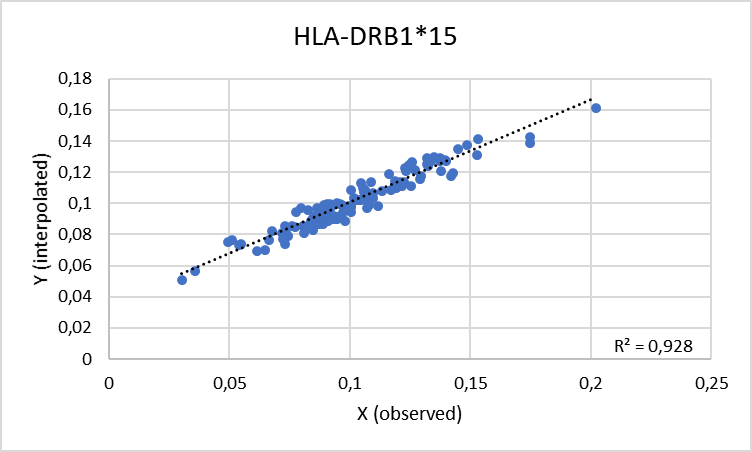


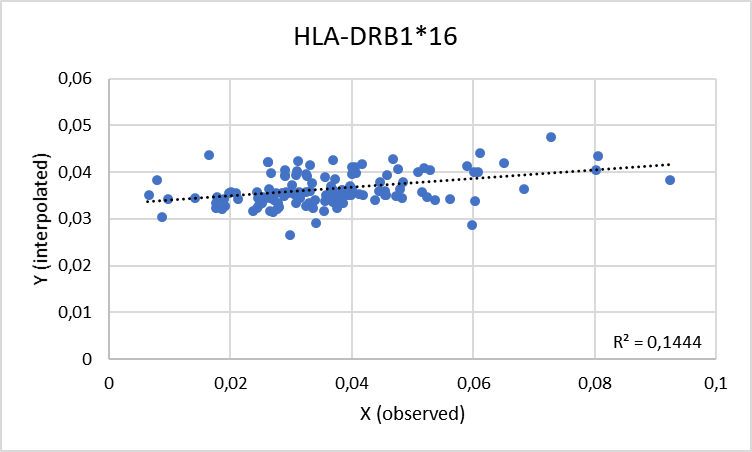


Genetic structure estimated by SPC (Figure 2)


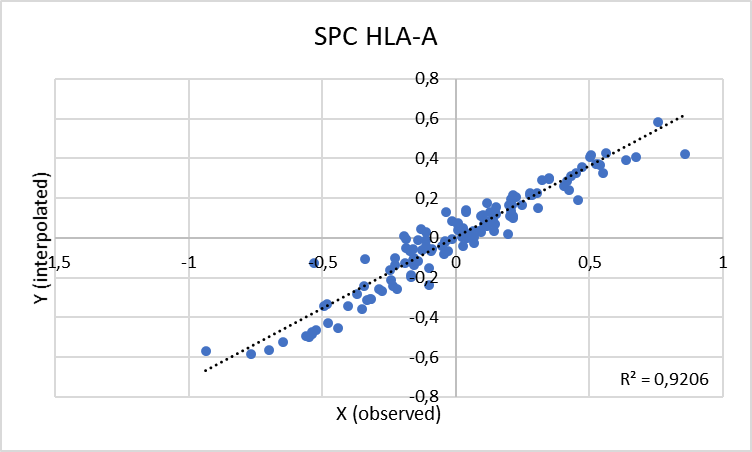

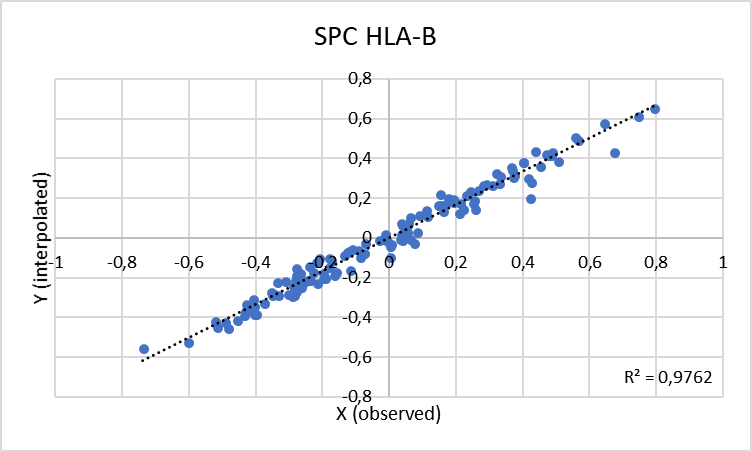


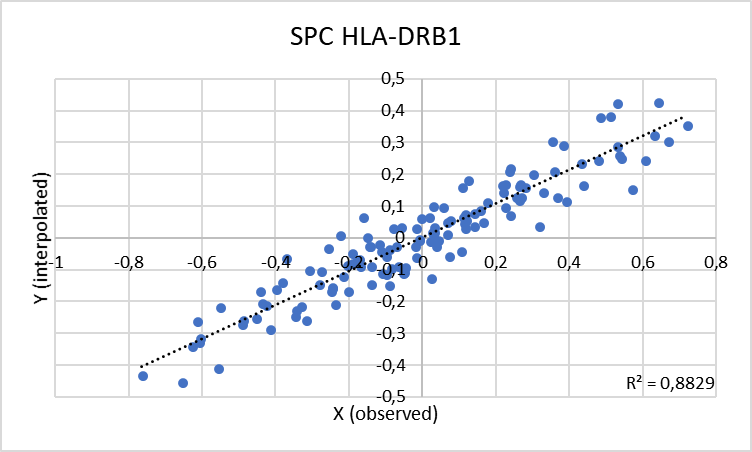

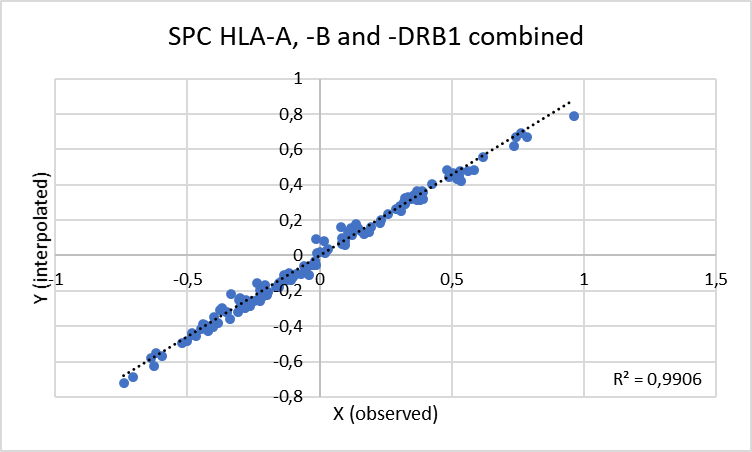


Haplotype Frequencies (Supplementary Material 5)


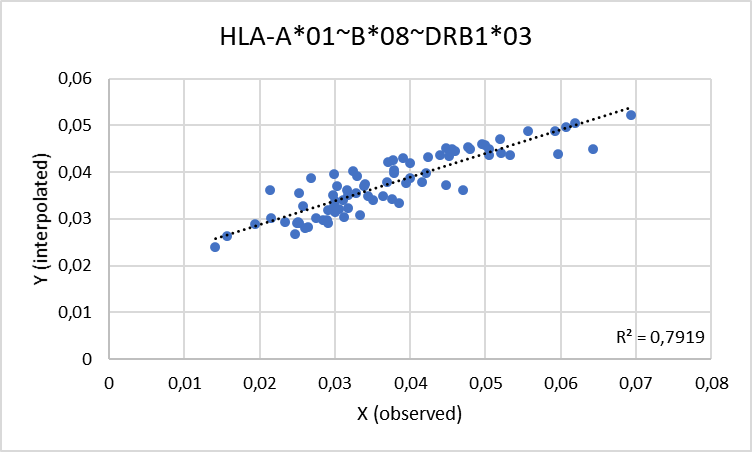

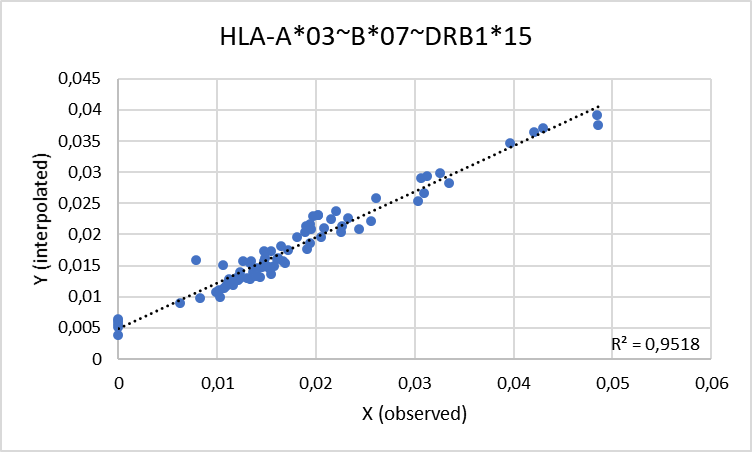


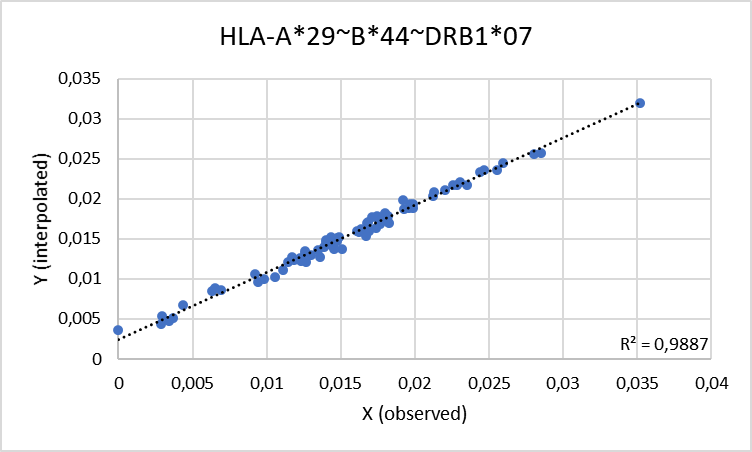

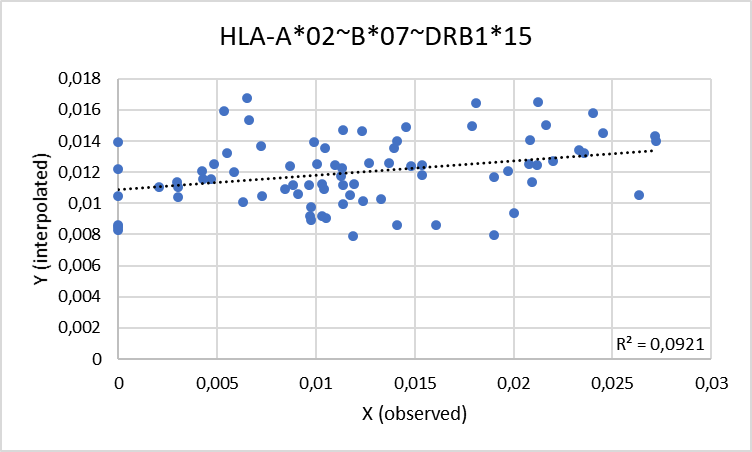


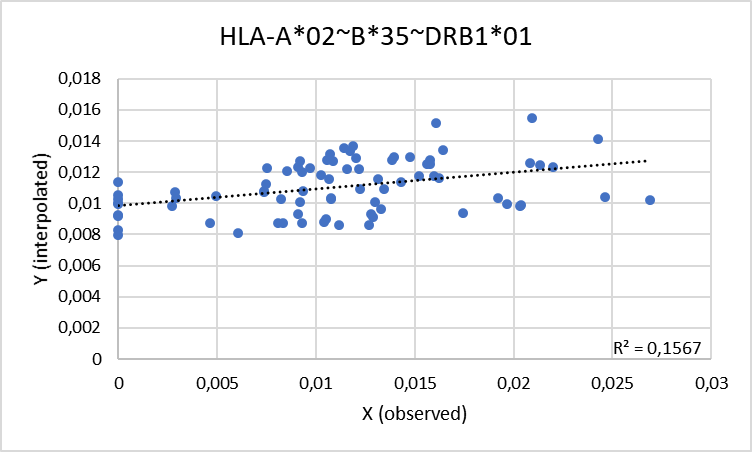


Correlation between alleles and autoimmune diseases (Supplementary Material 8)


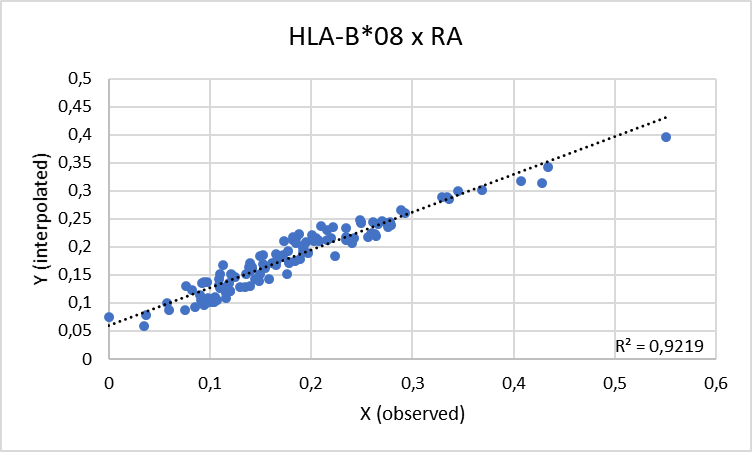

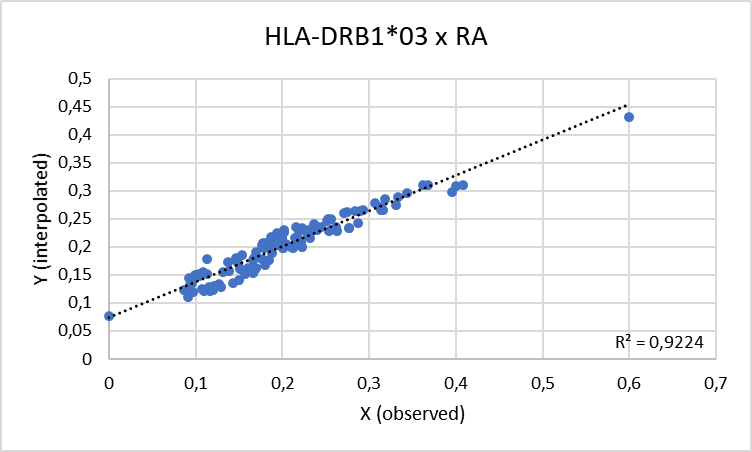


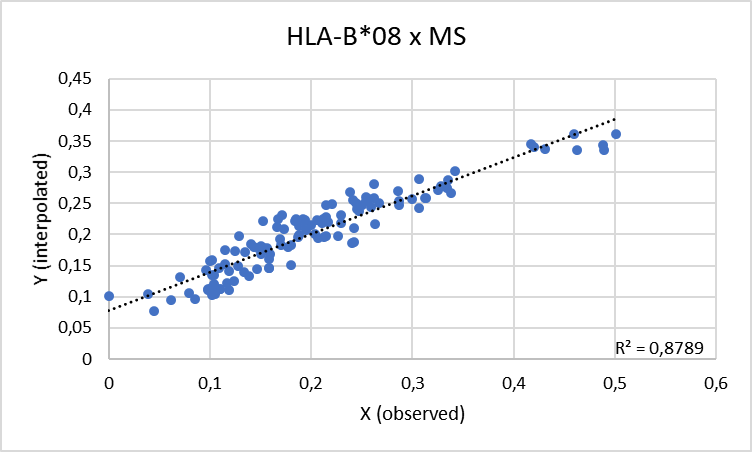

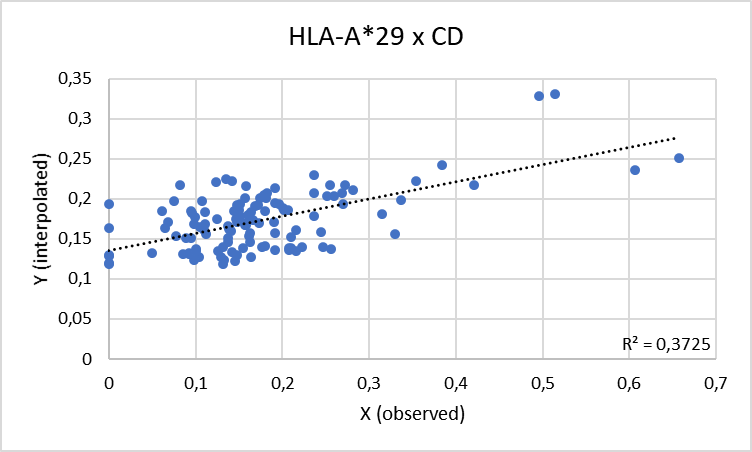


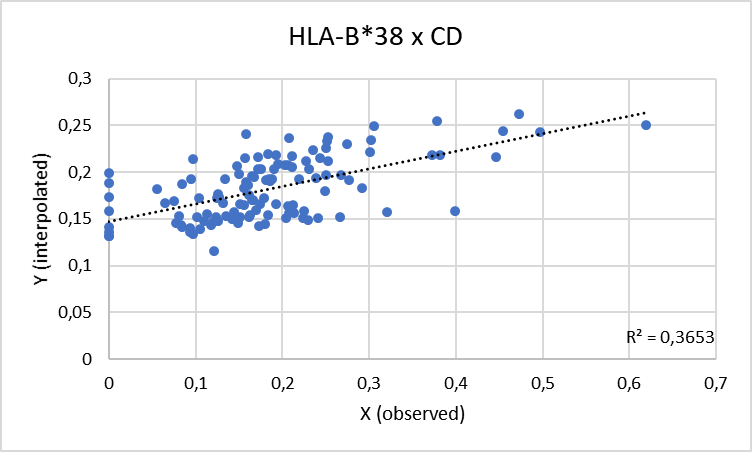

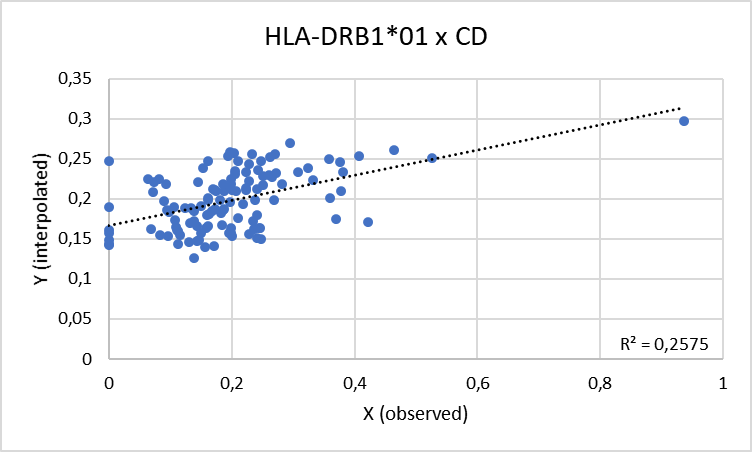

Supplement: Supplementary file 10 — Additional file 10. Scatterplot and coefficient of determination (R2) for each interpolated map. [file 12942_2018_154_MOESM10_ESM.docx]
