# Supplementary material for: Spatial analyzes of HLA data in Rio Grande do Sul, south Brazil: genetic structure and possible correlation with autoimmune diseases
Source: Int J Health Geogr. 2018 Sep 14;17:34. doi: 10.1186/s12942-018-0154-8 (PMC6137739; doi:10.1186/s12942-018-0154-8)
Supplement: Supplementary file 11 — Additional file 11. Meso-regions of Rio Grande do Sul and its colonization regions. [file 12942_2018_154_MOESM11_ESM.docx]

**Additional file 11 – Meso-regions of Rio Grande do Sul and its colonization regions**


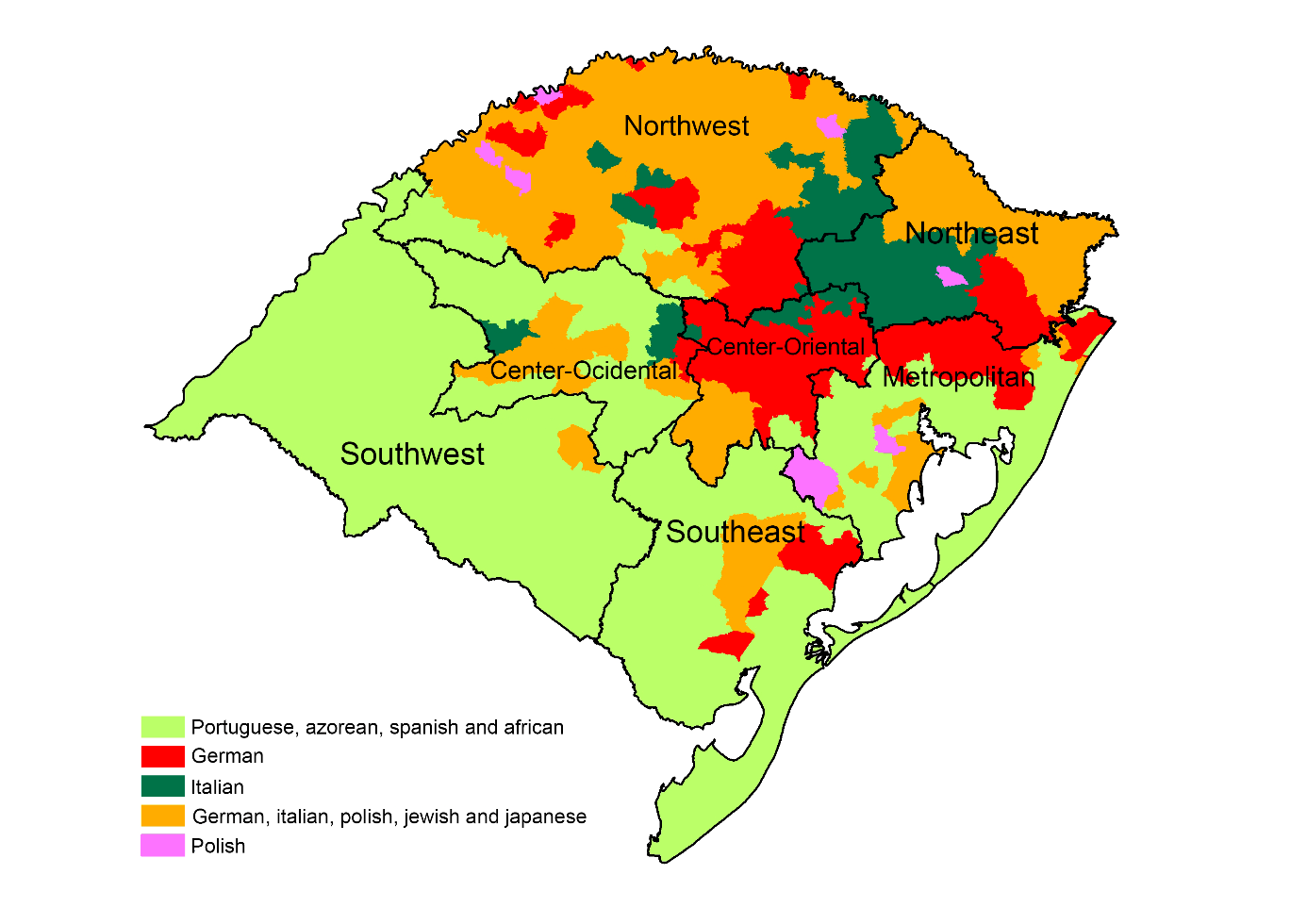


Meso-regions of Rio Grande do Sul and its colonization regions (adapted from Neto and Bezzi, 2008).
